# Growth by Insertion: The Family of Bacterial DDxP Proteins

**DOI:** 10.3390/ijms21239184

**Published:** 2020-12-02

**Authors:** Pierpaolo Di Nocera, Eliana De Gregorio

**Affiliations:** Dipartimento di Medicina Molecolare e Biotecnologie Mediche, Università degli Studi di Napoli Federico II, via S. Pansini 5, 80131 Napoli, Italy; dinocera@unina.it

**Keywords:** RTX toxins, bacterial adhesins, Ca^2+^-binding sites, type I secretion systems, modular proteins, site-specific endonucleases, target site duplications, Asp-rich motifs, HGT, horizontal gene transfer

## Abstract

We have identified a variety of proteins in species of the Legionella, Aeromonas, Pseudomonas, Vibrio, Nitrosomonas, Nitrosospira, Variovorax, Halomonas, and Rhizobia genera, which feature repetitive modules of different length and composition, invariably ending at the COOH side with Asp–Asp–x–Pro (DDxP) motifs. DDxP proteins range in size from 900 to 6200 aa (amino acids), and contain 1 to 5 different module types, present in one or multiple copies. We hypothesize that DDxP proteins were modeled by the action of specific endonucleases inserting DNA segments into genes encoding DDxP motifs. Target site duplications (TSDs) formed upon repair of staggered ends generated by endonuclease cleavage would explain the DDxP motifs at repeat ends. TSDs acted eventually as targets for the insertion of more modules of the same or different types. Repeat clusters plausibly resulted from amplification of both repeat and flanking TSDs. The proposed growth shown by the insertion model is supported by the identification of homologous proteins lacking repeats in Pseudomonas and Rhizobium. The 85 DDxP repeats identified in this work vary in length, and can be sorted into short (136–215 aa) and long (243–304 aa) types. Conserved Asp–Gly–Asp–Gly–Asp motifs are located 11–19 aa from the terminal DDxP motifs in all repeats, and far upstream in most long repeats.

## 1. Introduction

In most Gram-negative bacteria, large modular proteins, featuring sequence repeats ranging in size from 20 to 200 amino acids, are translocated outside the cell via the action of different secretion systems [1,2]. These proteins vary significantly in size (1000 to 6000 aa) and exert a variety of functions. Some are secreted in the milieu and target eukaryotic cells, such as the *Bordetella pertussis* CyaA and the *Escherichia coli* HlyA cytolysins [3], while others, in contrast, are retained on the cell surface and may either enhance biofilm formation [4,5,6,7,8] or stimulate adhesion to eukaryotic cells [9,10], or inhibit growth of non-akin neighboring bacteria [11,12]. Regardless of their function, many secreted proteins share a common architecture, as they carry in the distal end of the COOH region adjacent nonapeptide glycine- and aspartate-rich repeats, fitting the X-(L/I/F)-X-G-G-X-G-(N/D)-D consensus, and are hence referred to as repeats-in-toxins (RTX) proteins [13,14,15]. The number of RTX repeats varies among proteins from <10 to >40. RTX proteins are brought outside the cell by type 1 secretion systems (T1SSs), tripartite protein complexes in which an inner membrane (HlyB), a fusion membrane (HlyD), and an outer membrane (TolC) protein interact to form a channel through which proteins are transferred from the cytoplasm to the extracellular environment. The RTX repeats are a peculiar class of calcium-binding domains, which undergo Ca^2+^-triggered folding once extruded from the cells and form structures known as β-roll assemblies, which act as ratchets accelerating protein translocation through the T1SS channel [16,17].

Larger sequence repeats found in bacterial surface proteins also bind Ca^2+^ ions. The giant *Salmonella enterica* adhesin SiiE lacks RTX repeats, but carries 53 bacterial immunoglobulin (BIg)-like repeats, measuring 95 to 100 aa, in which conserved aspartate (D) or glutamate (E) residues form binding sites for Ca^2+^ ions [10]. Type I sites are critical for efficient secretion of SiiE, while type II sites are critical for adhesion and invasion of eukaryotic cells [18].

In *Legionella pneumophila*, a microorganism causing severe human respiratory disease, RTX proteins represent a major pathogenic factor that mediate the invasion of human cells [19]. *L. pneumophila* RTX proteins are highly variable, as they carry BIg-like repeats, which differ in number, length, and sequence content among isolates. Changes in the repeat composition of RTX proteins have been hypothesized to play a role in the degree of *L. pneumophila* pathogenicity [20].

This intriguing observation stimulated us to shed light on the process of intra-species variation of *L. pneumophila* RTX proteins and to investigate whether mosaicism of the repeat region might be a feature common to RTX-like proteins present in other species.

## 2. Results

### 2.1. Multiple Repeat Types in Legionella Repeats-In-Toxins (RTX) Proteins

**Figure 1:**
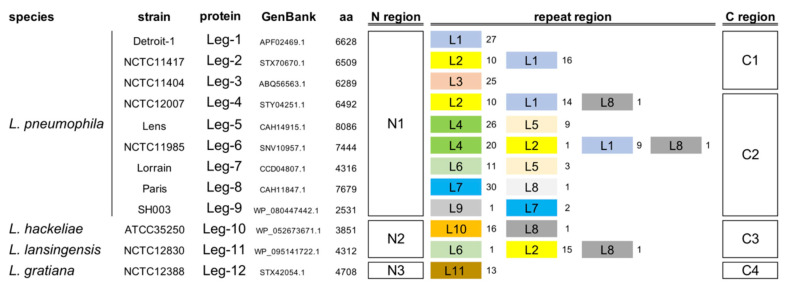
Modularity of Legionella repeats-in-toxins (RTX) proteins. The organization of the RTX proteins encoded by *L. pneumophila* and other Legionella species is diagrammed. N regions, C regions, and L repeats exhibiting >70% sequence identity have the same number. Repeat types are distinguished by color, with their copy numbers shown to the right. Protein modules were not drawn to scale.

The heterogeneity of *L. pneumophila* RTX proteins [20] was analyzed in a large set of samples. *L. pneumophila* RTX proteins share the same N region and feature two types of C regions, but differ in length and composition, resulting in changes in the number and type of repeat modules in the central region (Figure 1).

The repeats vary in length from 152 to 185 aa and show poor homology (35–40% similarity) to each other, but all feature DDxP motifs, with x standing for either G, T, or V residues, at the right-end side. Importantly, a DDGP motif is present in all proteins at the border between the N and the repeat regions. RTX proteins identified in other Legionella species displayed a similar organization (Figure 1).

**Figure 2:**
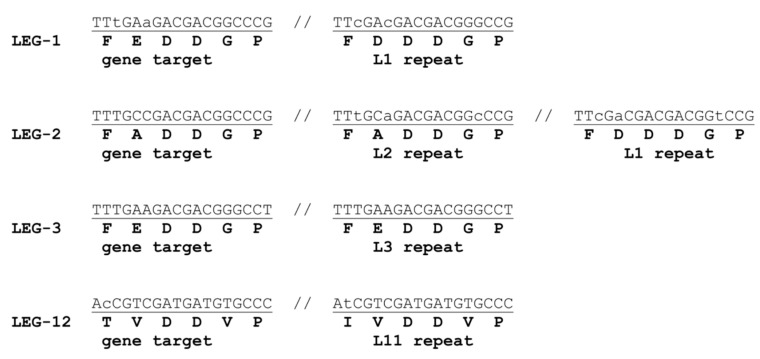
Target site duplications (TSDs) in Legionella *ddxp* genes. DNA duplications flanking DNA modules encoding L1, L2, L3, and L11 repeats in the Legionella DDxP proteins LEG-1, LEG-2, and LEG-12, respectively, are underlined. The corresponding amino acids residues in each protein are shown. Nucleotide changes between TSDs are denoted by lower case letters.

The heterogeneity of Legionella *rtx* genes results from the insertion of DNA tracts mediated by an endonuclease, which recognizes as the target the “GACGACGGCCCG” dodecamer encoding the DDGP motif into the *rtx* gene and which cleaves DNA both at the right end of the dodecamer and 6 bp upstream, making a staggered cut that produces ends with a single-stranded overhang. Repair synthesis led to the formation of 18 base pairs target site duplications (TSDs), which explains the presence of DDxP motifs at repeat ends (Figure 2).

Eventually, TSDs targeted the insertion of different DNA segments, determining the mosaicism of Legionella RTX proteins (Figure 1). Sequence arrays plausibly arose from duplication events involving both repeats and flanking TSDs. The sequences of Legionella repeats are reported in Figure S1.

#### 2.1.1. Search for DDxP Proteins

We set out to find proteins featuring the same organization of Legionella RTX proteins. To this end, Legionella RTX repeats were used as probes in BLASTp analyses carried out against the NCBI non-redundant (nr) protein sequence database. In turn, the N and C regions of the selected proteins were used as probes to identify homologous proteins carrying similar or different repeats. The pool of proteins fished out by homology searches was pruned by eliminating all of the sequences annotated as partial and proteins annotated as complete in Genbank, but truncated because initiating within DDxP repeats. Complex proteins found in Pseudomonas in which rearranged DDxP repeats are intermingled with other repeated sequences were not analyzed.

**Figure 3:**
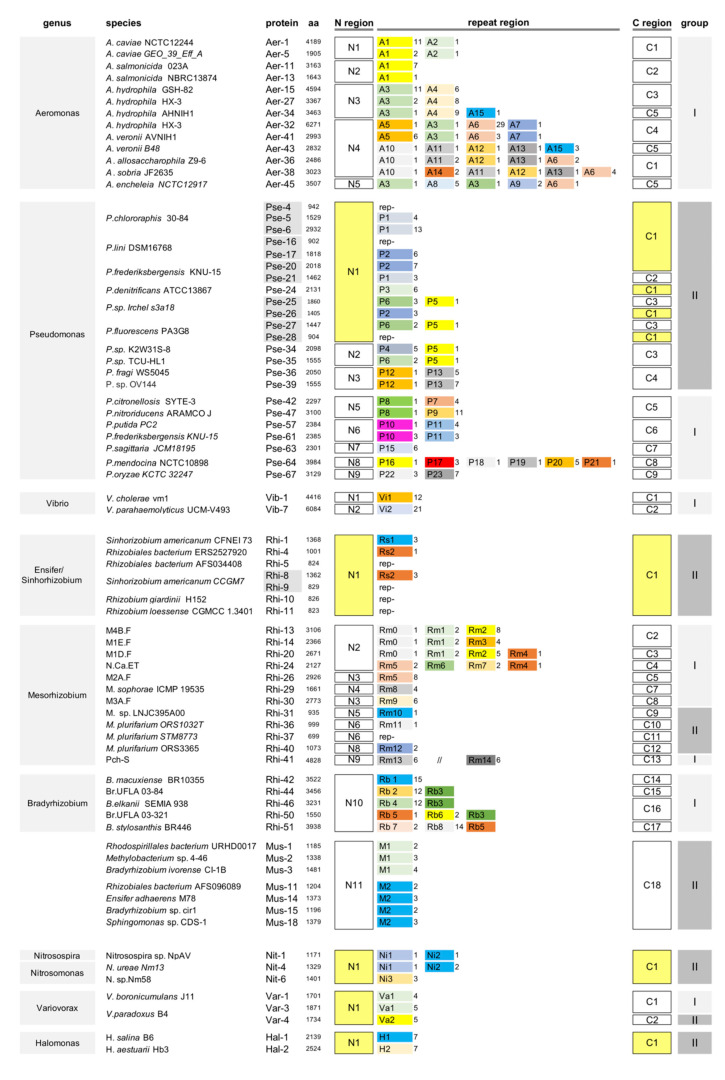
DDxP proteins. All proteins are labeled by a three-letter code denoting the genus. N and C regions are numbered, and repeats are colored in a genus-specific manner. Proteins lacking repeats are denoted as rep-. Proteins carrying or lacking RTX repeats are denoted as gr I or II, respectively (see light and dark grey bars to the right). Proteins encoded by adjacent genes are highlighted. Protein IDs, sources, and places of isolation of the producer strains are reported in Figure S2.

Hundreds of proteins carrying DDxP repeats, accordingly named DDxP proteins, were identified in Aeromonas, Pseudomonas, Vibrio, Nitrosomonas, Nitrosospira, Variovorax, Halomonas, and Rhizobia genera. The organization of >200 proteins was analyzed in detail. Proteins differ extensively in their modular structure and repeat organization, but can be assigned to two main groups, according to the presence (group-I) or absence (group-II) of canonical RTX repeats in the distal end of the COOH region. Information for each analyzed DDxP protein (species, strain, and place of isolation; GenBank ID; size, type, and number of repeat modules) is given in Figure S2. Representative DDxP proteins from different species were selected for further studies. Their organization is shown in Figure 3, while their sequences are reported in Figure S3.

The sequences of the DDxP repeats identified in this study are reported in Figure S1. Repeats are marked with one or two letters (i.e., L, Legionella; A, Aeromonas; Va, Variovorax; Vi, Vibrio) to denote the genus.

#### 2.1.2. Gamma-Proteobacteria DDxP Proteins

Aeromonas species are predominantly found in aquatic environments, such as rivers, ponds, and estuaries, but also in wastewater [21]. Aeromonas DDxP proteins are modular RTX proteins resulting from the combination of a few different NH_2_ and COOH regions and 16 repeat types. However, for a 107 aa duplication in N2 and N3, the NH_2_ regions are closely related (65–70% similarity). The COOH regions all contain von Willebrand factor A-like (vWFA) domains, but significant homologies are restricted to the RTX repeats region. *Aeromonas salmonicida* proteins feature only A1 repeats. In contrast, the repeat regions of other Aeromonas proteins result from the association of 2 to 5 different DDxP modules, many of which are found as single units. The repeat thread is largely species-specific, although is often altered by recombination events bringing in new modules (see Figure S2). The *Aeromonas hydrophila* HX-3 strain has two DDxP proteins, encoded by unlinked genes. Aer-27 features canonical A3 and A4 modules, while Aer-32 is similar to the *Aeromonas veronii* Aer-41 protein, plausibly because it is encoded by a sequence imported from *A. veronii* cells (Figure 3 and Figure S2).

Pseudomonas species are broadly sorted into the two major *Pseudomonas aeruginosa* and *Pseudomonas fluorescens* groups [22]. Most DDxP proteins were found in species of the *P. fluorescens* group, but many were also found in unclassified species from different habitats (see Figure S2). Pseudomonas group-II proteins have a limited repertoire of N and C regions, as well as repeat types. Most are encoded by adjacent genes, some of which encode homologous proteins lacking repeats (rep- proteins in Figure 3), a finding directly supporting the hypothesis that DDxP protein genes were shaped by the acquisition of DNA modules. Sequence alignments of filled and empty Pseudomonas proteins are reported in Figure S4. Group-I proteins carry different N, C, and repeat regions. In most, the repeat region is constituted by 1–2 sequence types. In contrast, Pse-64, a representative of extra-large proteins identified in *Pseudomonas mendocina* isolates from different habitats (Figure S2), features a patchy repeat region formed via the assembly of 5 repeat types. A similar repeat mosaic was found in Aeromonas proteins, while the adjacent P18, P19, and P20 repeats in Pse-64 and the A12, A13, and A6 repeats in Aer-36 and Aer-38 show 60–72% identity. The relatedness of P5, P10, P22, and P23 repeats to A15, A1, A9, and A8 repeats confirms that multiple exchanges of sequences encoding DDxP repeats occurred between Pseudomonas and Aeromonas genomes, respectively.

Group-I DDxP proteins were found in *Vibrio cholerae* and *Vibrio parahaemolyticus*. In both species, the proteins feature long repeat regions made from single repeat types, but are fully unrelated. Vi2 modules are highly heterogeneous and are assigned to at least two major a and b subtypes (see Figure S2).

#### 2.1.3. Alpha-Proteobacteria DDxP Proteins

Several DDxP proteins were identified in the alpha-proteobacteria division (Figure 3). Proteins found in species of the order Rhizobiales were labeled as RHI, while their repeats were denoted according to the genus, with Sinorhizobium (Ensifer) denoted as Rs, Mesorhizobium denoted as Rm, and Bradyrhizobium denoted as Rb. Proteins found both in Rhizobia and in other orders of the alpha-proteobacteria division, such as Rhodospirillales and Sphyngomonadales, were alternatively marked as multiple genera species (Mgs), while their repeats were denoted as M.

The few DDxP proteins identified in the Sinorhizobium (Ensifer) genus are variants of the same sequence type and differ primarily by the presence or absence of Rs1 or Rs2 repeats. DDxP proteins within the Mesorhizobium genus vary extensively, both in length and sequence composition. Some DDxP proteins feature 1–2 repeats of the same type, others feature mosaic repetitive regions resulting from the joining of multiple sequence types, while others lack repeats. The high number of proteins identified in the genus reflects the phylogenetic diversity of Mesorhizobium populations sampled in different geographic areas [23]. However, it is important to note that similar DDxP proteins (Rhi-14, Rhi-19; Rhi-21, Rh1-22; Rhi-27, Rhi-28; Rhi-31, Rhi-32) are produced by Rhizobia isolated from different geographic areas. The same holds true for the repeat-minus Rhi-37 to Rhi-39 proteins (see Figure S2). The large Rhi-41 (4828 aa), a protein which uniquely features two unlinked clusters of DDxP modules, is encoded by Mesorhizobium sp. Pch-S, a bacterium curiously isolated not from plants, but from an amoeboid organism of the genus Paulinella.

Proteins from the Bradyrhizobium genus have the same N region, but different C regions. Except Rhi-50, all Bradyrhizobium are greater than 3000 aa, each featuring a long cluster of a specific repeat type.

Mus proteins are a heterogeneous set of proteins that are highly similar throughout (>80% identity, see Figure S5A), and vary mostly by their number (2–4) of the unrelated M1 and M2 repeats present. Mus proteins owe their appellation to the fact that they have been identified in species belonging to multiple genera of the Rhizobium order and to genera of different bacterial orders, such as Rhodospirillales (Mus-1) and Sphingomonadales (Mus-18).

#### 2.1.4. Additional DDxP Proteins

The spread of *ddxp* genes among different species by horizontal gene transfer (HGT) is further documented by the identification of DDxP proteins in Nitrosospira, Nitrosomonas, and Variovorax genera (Figure 3). Nitrosospira and Nitrosomonas spp. are ammonia-oxidizing bacteria found in soil, such as in aquatic environments [24], while Variovorax are plant growth-promoting rhizobacteria [25,26]. A shown in Figure S5B, proteins found in species of the three genera are closely related to Pseudomonas group-II proteins. Nit-1 to Nit-6 are homologous to Pseudomonas proteins of the N1-C1 type. Moreover, repeats Ni3, Ni2, and Ni1 are 60–70% identical to Pseudomonas P3, P2, and P5, respectively. The similarity of Variovorax proteins to Pseudomonas proteins is modular. The N and C regions of Var-1 and Var-3 are homologous to the type-1 N regions found in Pse-16 and Pse-28, and to the type-4 C region found in Pse-36, respectively (see alignments Figure S5B). Var-4 has a Pseudomonas N1-like region, but differs completely from any Pseudomonas protein type in the C region. Pseudomonas sequences acquired by Variovorax genomes have been modified by mutations and acquisition of novel sequences, such as the cluster of RTX repeats found in the sub-terminal C regions of Var-1 and Var-3 (Figure S5B).

Halomonas are halophilic bacteria found in saline environments. Hal-1 and Hal-2, two DDxP proteins respectively identified in *Halomonas salina* and *Halomonas aestuarii,* are comparable to group-II Sinorhizobium (Ensifer) proteins. The finding that H1 (Hal-1) and Rs1 (Rhi-1) repeats show 72% identity reinforces the hypothesis that Rhi-1-like sequences moved from Sinorhizobium to Halomonas DNA. Sequence alignments indicate that all of the abovementioned proteins, as well as Mesorhizobium group-II proteins carrying different N and C regions, such as Rhi 31 (N5-C9), Rhi-36 (N6-C10), Rhi-37 to Rhi-39 (N7-C11), and Rhi-40 (N8-C12), feature large regions of homology at multiple sites and may plausibly be evolutionarily related (Figure S5B).

#### 2.1.5. DDxP Proteins and T1SSs

RTX proteins are translocated outside the cell by the T1SS machinery, while *rtx* and T1SS genes are often located within a single locus [13]. Most DDxP protein genes are associated with, or at close distance from, T1SS genes (Figure S6). Some T1SSs are involved in the so-called two-step secretion mechanism, and cooperate with periplasmic proteases called bacterial transglutaminase-like cysteine proteinases (BTLCP), which in particular conditions cleave the retention module anchoring a protein to the cell surface, determining its release in the extracellular environment [27,28]. Aeromonas, Bradyrhizobium, and some Mesorhizobium genes are associated with large T1SS clusters, including BTLCP- and LapD-like genes (in *P. fluorescens*, the BTLCP LapG is held in check by LapD; see [27,28]). The absence or presence of BTLCP-like genes allows one to hypothesize that proteins encoded by the associated *ddxp* genes might be translocated outside the cell by a one- or two-step secretion mechanism, respectively. Some *ddxp* genes are not associated with T1SS genes, and the mechanism involved in the secretion of their gene products is very unpredictable. In this context, is worth noting that Legionella RTX toxins are translocated outside the cell by a two-step secretion mechanism, but *rtx* genes, as well as the *hlyB–hlyD* and *tolC*–*lapG*–*lapD* gene clusters involved in the process, are unlinked [29,30,31].

The sequences, locations, and orientations of the genes encoding T1SS proteins involved in the secretion of the related DDxP proteins found in Pseudomonas and Nitrosospira, as in Sinorhizobium and Halomonas, all vary. This suggests that *ddxp* genes move among species as independent units and not as components of genomic islands together with T1SS genes.

#### 2.1.6. DDxP Repeats

The 85 repeats analyzed in this work are listed in Table 1.

The terminal DDxP signature is conserved in all but Rb4, Rm10, and Rm11 repeats, in which the second aspartic residue is changed to glycine, threonine, and valine, respectively. Most repeat types show limited (<5%) sequence variation. In contrast, repeats marked as heterogeneous (het) vary significantly in sequence (15–20%) within the same protein, as they do among different proteins (see Figure S3). Solo units represent about 20% of the DDxP modules. Solo units are neither degenerated versions of flanking repeats nor unrelated amino acid tracts located next to repeat clusters, as they all have the typical signatures of canonical DDxP repeats, and many are conserved in different species.

**Figure 4:**
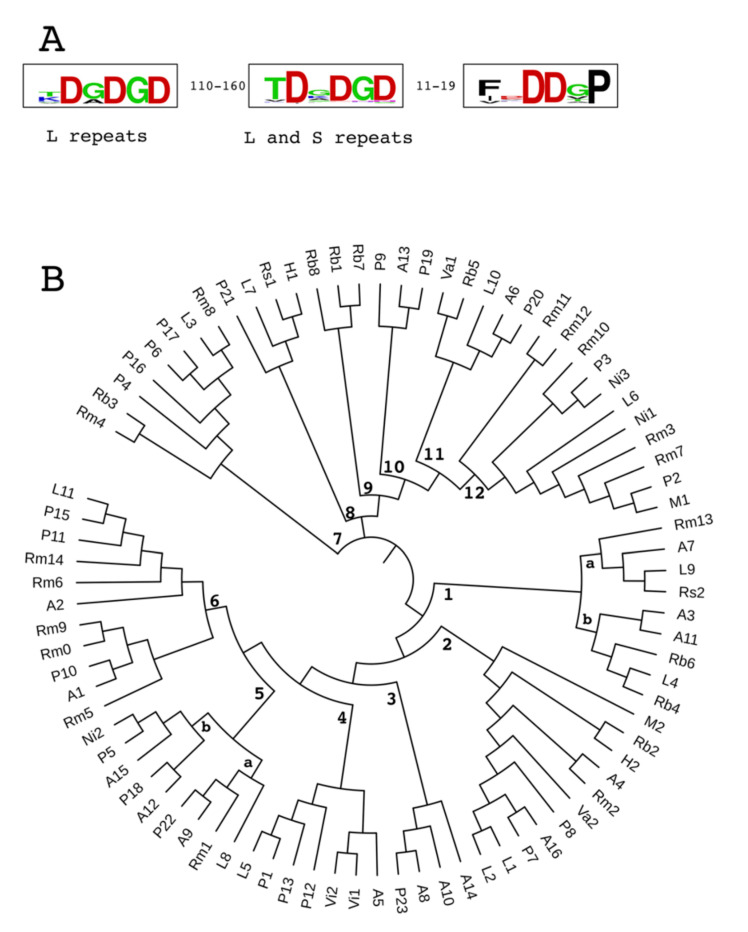
DDxP repeats. (**A**) Signatures of repeats. Conserved sequence motifs in short (S) and long (L) repeats are highlighted by WebLogo alignments. The number of amino acids between repeat motifs is shown. (**B**) Phylogenetic tree. The 85 DDxP repeats described in this work were aligned in a circular tree at the Interactive Tree Of Life (iTOL) site (https://itol.embl.de).

According to their size, DDxP modules were grouped as short (S, 136–215 aa) or long (L, 243–309 aa) types. S and L modules feature distinctive signatures. Conserved DGDGD motifs are found at close distance from the DDxP motifs in S and L modules, and also far upstream in 14/17 L modules (Figure 4A).

DDxP repeats were aligned in a circular phylogenetic tree (Figure 4B). The alignments of repeats within each branch of the tree are reported in Figure S7. Most repeats are related, and most branches of the tree in Figure 4B include repeats from multiple species, suggesting extensive inter-species mobilization of *ddxp* sequences. Certainly, it is worth noting that aside from *hal* and *nit ddxp* genes, the acquisition of repeats and hosting *ddxp* genes is uncoupled and might have been driven by homologous recombination between unintegrated resident and donor *ddxp* genes. Searches carried out by using the DNA sequences of a few representative repeats (L1, L2, A1, A3, P1, P6) as queries failed to find significant homologies outside the known *ddxp* hosting genes. The existing data rule out the idea that DDxP repeats are encoded by mobile DNA segments fortuitously inserted into cellular genes, a conclusion reinforced by the lack of direct or inverted terminal repeats, hallmarks of mobile DNA sequences, in DDxP repeat DNAs.

Many DDxP repeats are annotated in GenBank as T1SS-143 modules. T1SS-143 is a model domain of 137–143 amino acids derived predominantly from the analysis of proteins from *V. parahaemolyticus* hypothetically secreted by T1SSs [32], which were successfully exploited to find DDxP proteins in the genus Vibrio. The T1SS-143 domain is highly homologous to Vibrio Vi1 and Vi2 repeats, but matches to other DDxP repeats are restricted to the DDxP motifs and a few more amino acids stretches (see Figure S8). The presence or absence of a few residues identical to the domain is crucial for DDxP repeats to be recognized as T1SS-143. This point is paradigmatically illustrated by the Genbank annotations of Legionella proteins Leg-5 (GenBank CAH14915.1), featuring 26 L4 repeats plus 9 L5 repeats, and Leg-7 (GenBank CCD04807.1), featuring 11 L6 repeats plus 3 L5 repeats, respectively. In either protein, L4 or L6 is unseen, and only L5 is partly marked as a T1SS-143 module. Unfaithful annotations based on homologies to T1SS-143 fail to detect repeats in many DDxP proteins and also miss repeat size changes, such as the thread of repeat clusters.

## 3. Discussion

The modular proteins described in this paper have been apparently shaped by the action of specific endonucleases, recognizing dodecamers encoding DDxP motifs as target DNA. Sequence alignments suggest that recipient genes were cleaved both at the right end of the target dodecamer and 6 bp upstream. Repair of the staggered ends generated by the asymmetric endonucleolytic cleavage formed 18 bp direct duplications (Figure 2), accounting for the DDxP signatures at the ends of protein repeats. Eventually, TSD sequences functioned as targets for the insertion of more repeats of the same or different types. Head-to-tail sequence clusters likely arose from amplification events duplicating both repeat and flanking TSDs. Alignment of the targets of the genes encoding the proteins shown in Figure 1 and Figure 3 narrowed down the endonuclease recognition site to the 18 bp consensus sequence DTYnnnGAYGAYGKnCCV. Residues 1–3 are TTC or TTT in most targets, explaining the predominance of phenylalanine at the corresponding place in protein repeats (Figure 4A). Careful inspection of literature data failed to find a similar mechanism behind the shaping of the repeat regions in known modular bacterial proteins [3,4,5,6,7,8,9,10]. The hypothetical growth shown by the insertion model is directly supported by the identification of homologous empty proteins lacking repeats in Pseudomonas and Rhizobium cells.

Independently from the repeat content, DDxP proteins can be sorted into two mains sets, which differ based on the presence (group-I proteins) or absence (group-II proteins) of RTX repeats in the COOH region. Except for the RTX repeats and Legionella proteins, which are involved in host tissue invasion [19,20], the roles of all the other described DDxP proteins are unknown. The actin-binding RTX toxins described in *V. cholerae* [33] and *A. hydrophila* [34] are unrelated to the DDxP proteins found in either species.

Group-I proteins are a heterogeneous set of sequences. Group-II proteins, in contrast, are related in sequence, and most of them may plausibly be viewed as genus-specific products of a few archetypal sequence types spread by HGT and variably modified in different species by mutations and the insertion of different repeat types. Proteins of either group are likely translocated outside the cell by type I secretion systems, a hypothesis substantiated by the contiguity of most DDxP protein genes to T1SSs gene clusters. Taking into account the idea that T1SSs are engaged in one- or two-step translocation strategies [29,30,31], the presence or absence of protease genes in T1SS gene clusters could be indicative of the mechanism by which different DDxP proteins may be secreted. In some instances, secretion strategies may be unpredictable, since *ddxp* and *T1SS* genes may be unlinked, as in Legionella [29,30,31]. Aside from Legionella, Aeromonas, Vibrio, and group-I Pseudomomas proteins, DDxP proteins lack computer-recognized retention modules (RM) at the left-hand side of the NH_2_ region. RM are crucial for holding secreted proteins on the cell surface. RM-minus proteins may be secreted immediately into the external milieu, may use different RM-like plugs [18], or may adhere to the outer membrane independently from RM sequences, such as *S. enterica* SiiE adhesin [10]. It is plausible that at least the rhizobial proteins Rhi-13 to Rhi-30 may be retained on the cell surface prior to secretion, as their genes are flanked by T1SSs clusters, including protease genes (see Figure S6).

**Figure 5:**
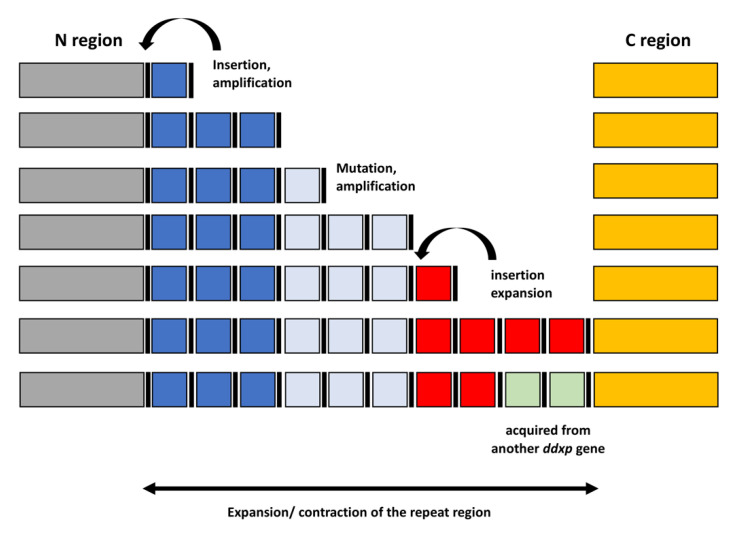
Step-wise modification of *ddxp* genes. Insertion, mutation, and amplification of DNA modules coding DDxP repeats are sketched. Black bars denote TSDs.

The repeat regions of DDxP proteins are highly variable. A few proteins feature one module, while most feature two or more. Steps in the formation and modification of the repeat regions in *ddxp* genes are summarized in Figure 5.

Upon insertion, a DNA module may go through rounds of amplification, mutate, or be amplified again, as indicated by the step-wise growth of many DDxP arrays (e.g., changes in Leg-3, Leg-6, Leg-10, Pse-63, Rhi-44, and Var-3 repeat arrays in Figure S3). Insertion and amplification of additional modules (only one is shown in Figure 5) may generate complex mosaic structures reshaped by homologous recombination, and DDxP proteins among isolates of the same species vary significantly in length because of dynamic expansion or retraction of the repeat region. New modules may also be introduced in the repeat region by homologous recombination between resident and foreign genes (Figure 5).

Changes in the number of DDxP repeats in the same protein may be functionally irrelevant [15]. However, the entry of *S. enterica* into polarized epithelial cells, which is mediated by the SiiE adhesin, is gradually reduced by progressive deletion of SiiE repeats [35]. Nitrosospira and Sinorhizobia DDxP proteins feature short repeat regions (Figure 3). A limited number of repeats may underly short-distance interactions between proteins and their targets. Interactions between the enteropathogenic *E. coli* surface protein intimin and a cognate receptor injected into host cells are indeed dictated by a protruding rod made by only two intimin Ig-like repeats [36].

The origin of DDxP repeats is unknown. The multitude of repeat types identified in several species adds complexity to the issue, as the corollary that the endonucleases mediating their chromosomal insertion are active, or functioned in the past, in species containing DDxP proteins. Repeats vary in length from short (S, 136–215 aa) to long (L, 243–309 aa) units. Conserved DGDGD motifs are at close distance from the COOH terminus in S and L repeats and are far upstream in most L repeats (Figure 4A). S and L modules are randomly distributed among DDxP proteins, and likely play the same functional role. Repetitive Ig-like domains stabilized by Ca^2+^ ions are a common feature of cell surface proteins, conferring them rigid rod-like structures that extend over the LPS layer. Aspartic acid residues are crucial for Ca^2+^ binding [16], with Asp-rich motifs present at repeats ends likely binding Ca^2+^, instructing the proper folding of DDxP proteins outside the cell. Most DDxP modules are related in sequence (Figure 4B and Figure S8) and represent variants of a relatively few sequence types exchanged and modified among organisms living in common habitats.

Several issues raised by our work call for experimental assays. The notion that homologous proteins may be empty or enriched by repeats is unprecedented and calls for experiments aimed at evaluating functional differences between the two protein formats. In several species, the same DDxP proteins vary in length according to the repeat number, as in the sequence for the presence of multiple repeat types. Whether changes are functionally irrelevant or may affect protein activity needs to be assessed. This point is crucial, in light of the finding that *L. pneumophila* DDxP proteins, exhibiting both types of variation, are involved in pathogenicity [20].

Answers to these questions raised will be provided by comparative analyses testing in different species of the ability of isogenic cells expressing size and sequence variants of DDxP proteins to assemble biofilm structures or adhere to eukaryotic cells, the two gold-standard assays routinely exploited to monitor the functional activity of bacterial surface proteins. Antibodies raised against empty proteins may be used to compare the relative abundance of empty and filled proteins located on the cell surface or extracellularly secreted.

Finally, the occurrence of DDxP proteins makes the assumption that other protein genes might have been shaped by DNA insertions dictated by the action of site-specific endonucleases plausible. Answers to this issue may be provided by careful inspection of the periodicity patterns of different classes of repeat protein sequences.

## 4. Materials and Methods

DDxP proteins were identified by BLASTP analyses in the NCBI non-redundant protein sequence (nr) database. Homology searches were driven using *L. pneumophila* RTX sequences as queries. Proteins identified in this way were in turn used to fish out more members of the DDxP protein superfamily. The relationships between selected sequences were analyzed by the bioinformatic software programs MUSCLE and ClustalW, provided by the Phylogeny.fr web service (http://www.phylogeny.fr/index.cgi) [37]. Repeat alignments shown in supplementary Figures S3 and S5 were obtained using the MultAlin software [38]. Conserved sequence motifs shown in Figure 4 were generated with the WebLogo software [39]. The phylogenetic tree of DDxP repeats shown in Figure 4 was generated by the online tool (https://itol.embl.de) iTOL v5 [40]. Conserved protein motifs were searched in the NCBI’s conserved domain database [32].

## Figures and Tables

**Table 1:** All DDxP repeats analyzed in this work.

Genus	Repeat	aa	Features				Genus	Repeat	aa	Features			
Aeromonas	**A1**	254	**L**			DDVP	Vibrio	**Vi1**	144	**S**		het	DDKP
	**A2**	260	**L**			DDAP		**Vi2**	142	**S**		het	DDxP
	**A3**	178	**S**			DDGP							
	**A4**	171	**S**			DDVP	Nitrosomonas	**N1**	161	**S**		het	DDGP
	**A5**	166	**S**			DDVP		**N2**	137	**S**			DDTP
	**A6**	158	**S**			DDGP		**N3**	172	**S**			DDGP
	**A7**	169	**S**	solo		DDGP							
	**A8**	204	**S**			DDGP	Variovorax	**Va1**	167	**S**			DDGP
	**A9**	267	**L**			DDMP		**Va2**	177	**S**			DDGP
	**A10**	205	**S**			DDGP							
	**A11**	176	**S**			DDGP	Bradyrhizobium	**Rb1**	181	**S**			DDGP
	**A12**	144	**S**	solo		DDTP		**Rb2**	200	**S**			DDGP
	**A13**	304	**L**	solo		DDGP		**Rb3**	215	**S**	solo		DDGP
	**A14**	219	**S**			DDGP		**Rb4**	186	**S**			DSGP
	**A15**	138	**S**	solo		DDTP		**Rb5**	172	**S**			DDGP
	**A16**	183	**S**	solo		DDGP		**Rb6**	185	**S**			DDGP
								**Rb7**	181	**S**			DDGP
Legionella	**L1**	179	**S**			DDGP		**Rb8**	177	**S**			DDGP
	**L2**	185	**S**			DDGP							
	**L3**	180	**S**			DDGP	Mesorhizobium	**Rm0**	267	**L**	solo		DDVP
	**L4**	174	**S**			DDGP		**Rm1**	269	**L**			DDAP
	**L5**	153	**S**			DDTP		**Rm2**	172	**S**			DDIP
	**L6**	168	**S**			DDGP		**Rm3**	153	**S**			DDGP
	**L7**	183	**S**			DDGP		**Rm4**	195	**S**			DDGP
	**L8**	153	**S**	solo		DDVP		**Rm5**	243	**L**			DDVP
	**L9**	174	**S**	solo		DDGP		**Rm6**	245	**L**			DDVP
	**L10**	155	**S**			DDGP		**Rm7**	157	**S**			DDGP
	**L11**	247	**L**			DDVP		**Rm8**	189	**S**			DDGP
								**Rm9**	269	**L**			DDVP
Pseudomonas	**P1**	153	**S**			DDTP		**Rm10**	162	**S**	solo		DTGP
	**P2**	154	**S**			DDGP		**Rm11**	160	**S**			DVGP
	**P3**	182	**S**			DDGP		**Rm12**	157	**S**			DDGP
	**P4**	185	**S**			DDGP		**Rm13**	180	**S**			DDGP
	**P5**	136	**S**	solo		DDTP		**Rm14**	244	**L**			DDVP
	**P6**	196	**S**			DDGP							
	**P7**	180	**S**			DDGP	Sinorhizobium	**Rs1**	183	**S**			DDGP
	**P8**	179	**S**	solo		DDGP		**Rs2**	177	**S**			DDGP
	**P9**	159	**S**		het	DDGP							
	**P10**	245	**L**			DDVP	Multispecies	**M1**	154	**S**			DDGP
	**P11**	244	**L**			DDVP		**M2**	168	**S**			DDTP
	**P12**	178	**S**			DDTP							
	**P13**	170	**S**			DDTP	Halomonas	**H1**	184	**S**			DDGP
	**P15**	248	**L**			DDVP		**H2**	201	**S**			DDGP
	**P16**	190	**S**	solo		DDAP							
	**P17**	202	**S**		het	DDGP							
	**P18**	144	**S**	solo		DDTP							
	**P19**	309	**L**	solo		DDAP							
	**P20**	158	**S**		het	DDAP							
	**P21**	183	**S**	solo		DDGP							
	**P22**	268	**L**		het	DDMP							
	**P23**	204	**S**			DDGP							

## Supplementary Materials

Supplementary Materials can be found at https://www.mdpi.com/1422-0067/21/23/9184/s1.

## Abbreviations

aa	amino acids
RTX	repeats-in-toxins
T1SSs	type 1 secretion systems
TSDs	target site duplications
BIg-like	bacterial immunoglobulin-like
