# Supplementary material for: Growth by Insertion: The Family of Bacterial DDxP Proteins
Source: Int J Mol Sci. 2020 Dec 2;21(23):9184. doi: 10.3390/ijms21239184 (PMC7730722; doi:10.3390/ijms21239184)
Supplement: Supplementary file 1 [file ijms-21-09184-s001.pdf]

Figure S1. Sequences of the DDxP repeats analyzed.

>A1
TAVSDVNLTVASENNLVLNGNVITNDVQGADGAAVTAGTLSGTYGSLVLNANGTYTYTLNPNDGDFKALTGGGVGSEVFTYTLTDADGDVSTATLTLTIK
NDDDGVTITNLTPKGEGGDAKVYEDDLLATRGENESAGSDSSKESTTVTGDFTISAPDGVKTLSVGGINIVVNGVVAGFPLSGVTTPLGNTLTITGYNPA
TGLVSYSYTLNDNEAHLTANGNNSLFEDFAVSLVDSDGDSASNTLSVQIVDDVP
>A2
TANDDADSATEGVFTPVTGNVITGAGTIGGTAGAGVDVMGADGAAISQVVSSNGNSDSNPAGGFTVTGLYGTLTMGLDGEYSYTLTAASVPVGASEVFTY
TLKDGDGDTDPATLTINIAQDTRVPVLTIGNATVDEEGLPEGTAASGNSEIFNGSFTINTQGENLTTLTIGGQTYNLTSGGSQTLINNTEGMLTVTGVSG
PTAGVYTVNYTYTLKDNVLTHNVQGNSDTANGPSFVVSATDASGDSGTGNLQVVISDDAP
>A3
RAGLAVEAPSLGASVDESLVSLGGAGSDGVASATLSAANVQAQFNPAFGADGAGSIGYSLALTGSNVASGLYAVDPAAANGQGAAIVLNQVGNVITGSAG
GVDYFTLTINPSTGEVTLALLDNVWHGDTTNADDSVALTLGQGVLTLVQTVTDADGDSASAAVDLGANGVFRFEDDGP
>A4
SVTINAVADGGITLTTQDAQTIGSASDTATGSFAAAFLAAAVPSYGADGPGTTTVSGYSLSVTDSNSGLTSNGLAITLTKVGSDIVGSTTAGEVFRISVA
SNGTVTLTQSAELDHLPEDVDNSNDNNLISLANGKVLLSATVTVVDGDNDTATGTVSADLGGNISFEDDVP
>A5
VLAGGEDERPQVGGQVQEDALTSGNSEGTGQSASASGAAGTLNNLVNFGADGAGSFGLGSDVSSLTAQGLTSGGVALSYSVVGNVLTASAGSVPVFTLTV
GVDGSYSFTLSGPLDHPVADGDDSEQLAGVGIDFSGVLTATDGDGDPLTGSFPAGSFAIRVEDDVP

>A6
SISAGSAANDSLQVDESNLSLNASTSFASLFTSSFGADGAGTLVYSLNVSAAGADSGLNDTATGQDILLYLESGSVVGRVGGSSGSVAFTLSVNSSGEVT
LDQERAIAHTPNSGPDQESSLLSANLVKLVGTVTDKDGDSQSASVDLGSAISFKDDGP
>A7
SASAKAAASAGATLDETGGFDAVDITSSAISGLFNTPVYGADGSGSVQYSLSATAGAGTGLWLEGQSGSASEIKLVAVANGYEGWTGGSTSGTKAFSVLM
DGNGKVTVTQYAALEHGVDGSSASDHDDSVSLAAAAAIKVVQTVTDRDGDSTTATSASGLNIAFKDDGP
>A8
TLTVEANGEAVAALAVTLDETTGAADHYANGEGADGYVNDDAPGALARVTTQVVGGLVSLFNVGGSYGSDGAGTLSGTMSFTGVPLGGLSTNLSATDGGA
ITLVAVGSGTLNGVDGDGNIVFSIQIVDVGGGVLQLQTTQFEALAHGSDGLFDEALSLLLNQGSLALQYEVIRTDADGDPVRVSDSIVLADGERSVFSFD
DDGP
>A9
IATDEAAQSVAEGTTKLGAFDFVPGADGAGVSHINGIALTFGGDGYSQVVDVGNGVLKVKADGSYSYTADASVDTTNPVTDNLTFTVTDGDGDAVTKAAS
FNITDANVPTGGSTTAAVDDDGLTGGNPASTTGDLVVAPDPDTNEATFSGTLTFGFGPDGAGSVNFAAMHGTSGTVGQETVNYSWNGANNTLTATGPRGP
LFSVVVNPSTGAYTVTLLDNVQHATLDGQPGDNTENDAVTALTYTVKDADNSTTTGTLTITFDDDMP

>A10
TQSVSVAEEANVGGLSVSLDETVGEGDIYNGSDVDDGYVSDDVTGALAQATTAITGGLQSLFTSSGSYGADGEGSTTGSFSFVGIGEAGVATNLSATDGG
AITLNAVSATELQGIDSDGNVVFSIKIVAVDGVSQLQTTQFEALSHGNIDLFDESLPLLLSGQNTLGLQYSVTRVDGDGDRVTDSATVSLVAGERSVFSF
DDDGP
>A11
SSSVQAEASLKAAVVDESLATLGGAQGDGVAQSYLDASSIEGLFNYTYGADGAGSRVFSLELAGRDVGSGLFALGSDGAKGSEIVLNQGLDGVVTGSVGS
VNYFTLAMDGDGKVTFTLLDNVWHSNTGSNDDASTLNVAAGQLNIVQTVTDGDLDSVQSRFDVGSSGVFKVEDDGP

>A12
KASNVEAPLVLDDEGLAGGINGGPSDVAGANTVTSGNLGYQAGADALKSLELSGPISLGTESVTSTWDAQSGTLSISSTRGVLMTVTITDPSTGAYSVKL
LQPLMHSEVDSEDNITLNVGYKVTDGDGDSATGSLAVTINDDTP

>A13
TIQVGELDLNSSVTFLGSNAGYSNSYGYYIKGDDGTPVSGQVIWANVHNQSSGNQVDISALDPAHTGFFVIPNGGANPSLGNNTVVTFQLVDGIWQAFMG
STPLSGADGANIVFSDASLNPGGSHLQDTGSAGNQNWEDMTANSDYDYNDVSTSVTWGGAVQLQVDESNLNHDAIADFSGVFNVQPGADGLGSQNYSLNI
QNVNSGLIDTATGEQIRLVMNDSTIEGRTATTGELVFSLTVDGSGKVTLDQLRAVEHPTSDPDEATFLGSGHVSLTLTVTDKDGDHASGVLDIGKVISFK
DDGP

>A14
TLSVSVEVSKEELAALQANLDESVGEDRAASGEVADGNGDDAGPGLAQMYSSVSGGLASLFAQSGVSYGSDGAGSTTSSLSFVGVPVGGLATNLSSTAGG
AITLFWEEGDLVGRDANGGDPVFTIKIVNVADAGDPPVYQLETTLYEALDHGDNARIDEAVNLLLSQDGAVQLQYSVTLVDGDGDSITRSATMDLISRST
DEQEVVSQSSVFSFDDDGP

>A15
SISANAVVQLDDDALAGGIAGGTGDDPNAVNVSGILGHSFGADGAGSVQWLTTGAPAGFTYETNPAGALLVKQGATTVLTVTLNSATGAYSVTQNSPIQH
ANADQENNQAFTLNYKVNDGDGDTATGTLAINVDDDTP

>A16
SVTITVTDDNSVVLNTQDAQTIGELTDSDGPVSFASAFVASATSFGADGMGSDSWSYGLTLLAASGADSGLDSNGSTVYLYKVGDVIVGSTAASGVGLTT
SSSSVVFTLSVNGGGGVTLNQMQEIDHAGPGVTSGYGNQEAILGSGLVGLTGTVVRTDGDGDTATDSKTLDLGGNVTFDDDGP

>P1
TAxGTAxAGTVDEDGLANGIAGGVGDVPGEATTASGSVTGIFQSGADVPLTYSVSSDTSGLPALSSGGVALVYSVAGDTLTAKAGAVDVFTFSLNAAGDY
SFTLLQPLDHPAGNDENDITLNLGAMLQATDKDGDTVTAAAEKLVITVDDDTP

>P2
SIxTTGTEPTLTVDETVLTTDATQSFAANFSSAFGADGAGTLTYALGVVAGxSGLTDTATGEAVNLSLNGTVVEGRTATTNLPVFTVSVAANGDVTLDQQ
RAVVHPDATNPDDSTSLTSDNLVTLTATITDKDGDSAxATLNIGQNLVFKDDGP

>P3
SITPSEASVPTLTTDDTHIPDSDGPTSFADLFNPDFGKDGFKDADDDNxQDADAxTYALGVSADNGVDSGLVDTLSGDKIYLFLENGSVVGRVGTAAGQA
DPTGDIALTIAVDANTGAVTLTQNNSVVHDDPqDPAETStSAAGLAAANLVTLTATITDGDGDTASTSRDIGDAFKFEDDGP
>P4
TAQIARTADNAVLDESLGVDANDANAEDDDVPPATDPFAGSYGAPIGAVAGVNLADTTTATGTDVVGATTAVTLSIVNGNGSDSGLDTTDGTQINLWKEA
NGDVTGRAGNASATVIFAIRIDNDGKVAVAQYDSIKHPDFPNNYDEELDLTGKLDAVVTVTDGDGDVATHNIGIGDGINFQDDGP

>P5
TVSANNTVLLDDDALTGGNPGGTXDDADSANXSGTLGHAFGQDGAGTVAYLTSGAPTGFTYVADGTSLLVKQGTTTVLTLT$VAATGAYTVAQNNPILHA
AGLTENNQAFTINYRVTDGDGDTADGTLSINVDDDTP

>P6
SADVIDGGGAVTIDETAGNQDNDSDAAAVLALFDTEVVNKGTDLNPAEFAISTTPLATTLLSSFGQDQEGATKVLSLAIVGGDGTDSLLNTTDGKNILLF
KEGDLIVGRYDVANGVVTVADPAAFAIALLQDGNVAVAQYVSLTHPTAGSTAAAHDDRIDLSGLINAVVTVTDGDGDTSTDSVGIGDDINFDDDGP

>P7
SIxIgLSGKVPVLLHTQDADTIGSNFDTATTSFAAAFAITSQSAGADTPAAvSWSYALKLDVAEGTQSGLNSAGQKIYLYEQNGQIIGSTASSEGGVNSS
NTIFNLKVDGSGNVTLTQYAEIDHALPGATSNYAAQQAFLGTGLVSLDATATITDYDGDKATSTASLDLGNKVAFDDDGP

>P8
QAGLSADNGVDVLLQTHDANTAGAAFDTSTADYSSAFQVTANYGGDGAGSTQLTYSLNLVGGNGANSGLTSGGATIYLYSVGGAIVASTSASAAGITGEN
TVFSLSVNGSGVVTLTQYSAIDHALPGSESGYASQTAVLDSGLVQLQGNLTVTDRDGDSVSSSSSIDLGGRVSFTDDGP

>P9
SIXPXXXXDIXLTVDETXLXXDATSXXXXXVAXLFTAHFGADGAGSITYKVETXDGTDSGLKDTATGXXIFLFNTASGVEGRVGGXNGXVAFRVTLXXDGX
ITLDQVRALVHPDTXDXNDPLSLXXGKIXLTATITDXDGDXXSASIDLGSKLTFLDDGP

>P10
TAVADTNASVATEAATVLTGNVLTNDVQGADRVTGPITAGTFTGTYGTLVLAADGSYTYTLNPSDPDFYNLHGGGTGTETFTYTLSDADGDTSTANLVLN
IKNLNDPVTLNGLDAXGGELSVYEKNLSDGSSPNAAALTQSGTFTVTAPDGLQTLSVGGINVISGGVAATFPQSTTTPLGNTLTVTGYNPATGVVSYSYT
LLDNEAHPTGGGINNLTESFTVTATDTDGSTSSGSLDVNIVDDVP

>P11
KAHXDXASVXEGGTVSGNVLXNDXXGADXXGDGQYVVGVRAGSDTSTSAIGQLGSQVMGQYGYLTLDAQGNATYHANPDSVXPAGATDVFVYTIRDADGD
ESTTTITINVXDCSLVAXPDXGVTVYEKALDLVKDGNDLAPGTVTGSDPHSTGETASGSLAGSVSGGXGALTXTLVGNAVGQYGQIQLNADGSYTYTLTS
APKSPGNVNDGANSLTESFTYXVKDSLGNSTTSTIVITIVDDVP

>P12
AAAGLPVTALVDEDSLAGGIDGGVGDAGLLVPASASGTVATVFTSGTDAPASYSLSNDTSGVQVFDSAATAVALASKGETVKYDVI
GNTLWGYVGAAAEYVAATDRAVFKLELTNTSDGSYTFTLLDQLDHPDTVGGDNSENELLLQLGSVLKVTDKDGDSVTATA

QKLVITVDDDTP
>P13
IATLNQLTGTVDEDGVLEGAANAGPGDGIAGGTGDVAGEIVLASGNISTLFQSGADEALNYSLTTNGMAALGLTSGGVAVTYAVGFDAGSSTWLLTASAGA
GNTAFTFSLHATTGAYAFTLVDQLDHASGLNENDLSIALGTAINATDFDGDTVTAAANGLVITVNDDTP

>P15
TAQADLNSVVEGAAVSGNVLSDGTADVFGADGPAAGGGVVGVRAAGSDTSTAVTSGVGTTISGLYGTLTLAANGSYTYTSTPNAVPLAGASDVFVYTIKD
GDGDLSTTTLTINLSDGGLSAPADQDVRVYENALDLVKDGLDLAPGTVTGSLPGSTGETDADNQLDAVGVVPLSYVLVGEAAGTYGTIQINADGSYTYTL
TSPYTSXGDANDGANVEAGVDSFTYQVTDGYGNTLTGTIRIDIVDDVP

>P16
SAGLEFSREGGEVVLDESAGLQPGSHDVAGPLAVFNGVANPSSDMRAYAQGTAPAVVANYNFGADEGGATAVYSLALVGSGEGEQGPQVQAVGLTTTSGS
PITLSVENGLVVGRDAGGNAAFAISIDPDTGIISVVQYQSLHHPDASDANDSIDLSGLIRAVLTVTDGDGDQAVASADIGQLIRFVDDAP

>P17
TAAIERVEGVRVTHDESAGLQNGIATPTPGGDANDNDTNAGSVVGLFSSVSNTGTDLSSPGYATSNGAVVDISGSSTGEDNEGATTVLSLSIVGGDGSDS
GLTTTDGQTIRLYLENGLIVGRVDGGETSPGAAAFAIAIGQDGSISVAQYISLHHPDTGSADDAVSLAGKVRAVVTVTDGDGDVDTAQVGIGGAIRFEDD
GP

>P18
TATKVTATNVLDDEGLNGGINGGPGDVSGAHTSTSGNLGYDAGADGLKSIELSGPSTLGSENVTSTWDPDTNTLTISSVRGDLMTVVLTDLASGAYTVNL
LKPLMHTVDGTEDNITLNVGYKVTDGDGDTADGSLTVTINDDTP

>P19
TLQVGALDLNSSVTFHGTDAGYSNSYGYYIKGEDGTPLSGKVIWANVHDQSSGDTFDLGDLDPASTGFFIIPNGGGNAGLLNGADVTFQLVGGKWQAFVG
GTPLIGADGANVLFSDATLNPGGSHLQDTGNAGNQNWEDKTDTSDYDYNDVSTSVTWGSTLQLQVDETHLEQGNSATATADFSGLFNVQPGADGLGSLDY
ELQVTNPASGLVDTETGEDVVLFLEGDQIVGRVGDANGEAVFTLTVSADGKITLEQLRAIEHPTADPDEAAFLGSGHVELKLTVTDGDGDSVSGGLDIGK
VISFRDDAP

>P20
TISAGQAGVASLQVDETTLAINATTNFSGAFTSNYGADGAGSISYVLDVKSQGVDSGLKDSASGSDIKLYLEGGEVVGRVGDQNGAIAFKVTVDASGNVS
LDQVRAIVHSPNTGPDQATGLSAADLVKLVATITDKDGDSNSASLNLGNAISFRDDAP

>P21
KITGFELQGGGRVYVDESVGTAGSSRDENGYVAPNDEQGHGNVLGYARIQGSDLFNLATNGGSDGLDASRTQFALSLVSEGVDSGLDATAGGNILLYTDS
NGNVLGKVGLDTIFKISVNTVDGSITLEQYKAIAHGNTASHDELALIREGLVKLGVTVYDKDGDSDSASLDLGKVVGFEDDGP

>P22
SAxXEPQRXLxEGXTXXGVFDFVAGADGAXVTHINGQALxFGQDGYSQXIDTGHGLIKVKADGSYSFTADASVISXEXVVDNLTFTVTDGDGDAVTXNAS
FSITDANAPTAGXTXAAVDDDGLXGGNPASTXGDLVVXPDPDLNEATFSGTLPHDFGXDGPGXISFAAMDGKTXTVGTETVTYSWXADXNTLTAIGPRGX
LFTVXVXPLTGXYTVTLLQNVLHXSLXGQXGDNTENDATADLIYYTVSDADXSXAQGTLSITFDDDMP

>P23
TQTVTANAGGTAAVTVSLDETTGPSDHYAAGEAADSYVNDDVPGALARATTALSGGLLGLFTVSGSYGSDGAGSLSTAVSFQGVPAGGLATNLVATDGGA
ITLFATGAGVLEGKDATNDTVFKIEIVNVGGALQLQTTLYEALDNGTDSLFDEAVSLLLEPGSTLSLQLQVTRTDGDGDTIVASDRVVLANNTTSAFSFD
DDGP

>Ni1
SINTTGXEPXLTVDETVLATNDTKNFAANFTSAFGXDGAGTLXFALGISASGXDSXLVDTAXXNHVFLFLXXGVVQGRXGTDAXXAAGGDIVFTVXXXXG
NVTLDQQRAVVHPDATNPDDAKXLLADXLVTLTGTIXDXDGDXXSATLNIGXNLVFEDDGP

>Ni2
TVXANSLVKLDDDALAGNPGGTGDDIDAENVSGTLAHSFGADGAGSIAYLTTGAPAGFSYVADGDNLLVQQGTTTVLTLTLNTATGAYLVTQNAPIXHAA
CGLDENNQAFTINYRVTDGDGDTADGTLAIDVDDDTP

>Ni3
TIAANTEPTPTLTTDDTEIVDTAGPVSFAGLFTSAFGNDXFKDADDDDVQDTDAITYTLGVSGANAVSGLTDTLTGDGVVLNKVGNDIVGTAGAAGPEVL
RITVDASGNVALSQSRAVVHDDPLDPSESSSPATLLTADLVTLAATITDGDGDTATATRNIGDAFKFEDDGP

>Va1
SIXVADTAPPDALTVDETALATNASASFADNFSSTPXYGADGAGTVXSAYALSVKSAGVASGLVDTLTNQSVVLTLNAGGVVEGRTAISGXLVFTVSVDA
AGLVTLDQQRAIVHPDXTNPDDSKTLAAADLIVLTRTDTITDXDGDQNTGSDFLNIGQALNFEDDGP

>Va2
SVVLAAPIDTALVNTQDADTIGAATDSATQDFSTAFGVASSSYGADGAGTTVSSFALGVATQGGDSGLKSDGATIYLYLVSGNVIGSTAATEAGITAGNT
IFDLAVSATGSVTLQQFAEIDHALPGDSSNYADQEAT$ADTLITLTNTVTVTDGDGDTASDSKVLNIGANIKFDDDGP

>L1
SVTMTVSDNNVITLNTQDAdTIGaaSDSDSASFAAAFAVTPNYGADGAGTTVTTYALSVSAQGVDSGLDNNGNnIYLYnIagSVVGSTSATQAGITTGNT
IFSLGVNSSSGVVTLTQHQEVDHGLPGASSNYAAQEAILNTGLVFLNATAVTTDgDGDTATASASLDLGGNVKFDDDGP

>L2
SVAVNVGNEAGILLTTQDADTIGTATDAAVTSANFGNVFSNTPNYGADGAGSTVMSYSLGLIAASGADSGLDSNGSNIYLYDISGTIVGSTSATQAGVNA
GNTVFDLSVDGAGVVTLRQYAEIDHPIADDPSASGAPFDDQLAVLGNNLVSLTATALTTDGDGDTATASERIDLGGNVRFADDGP

>L3
VAQIAATGVKVTHDETAGVNAGSDDVASPLALFAGVSQKSTDMAGFAQSSGAVVSSAGSLVGQDNEGATIKFSLAIANAASGLQTTDGDAITLTLESGFV
VGRDAGGDAVFAIAIDADTGVLSLAQYESIKHPSGGASYDEAVDLSGKINAVVTVTDGDGDVATQAIGIGDAIVFEDDGP

>L4
SIDGSKVLSADVLTVDETNLGVNATASFADNFAQAIDFGEDGAGSVSYALVLNGNNVGSGLYAIDNLDVSTADGDGIGRGGEIVLNQNGNVVTGSLNGTD
YFTITIDAASGTVTFDQLASVWHANTANPDDQSALQALANSLVVRATVVDADGDKAAYDLDVSQGVFQVKDDGP

>L5
VASATKLTGVVDEDGLAGGIAGGTGDVAGQAVAASGNVAALFQSGADAPLSYSLNSSTSGLPALSSGGVALSYAVSGDTLTASANGTDVFTFTLGSNGNY
TFTLLAKLDHPAGADENDIAINLGSVIRATDSDGDTVVAAADGLVITVDDDTP

>L6
SIVVSGATQTLTVDESVLTTNDTQSFAGLFTPSFGADGAAAANALSYSLGVSANGAASGVLDTASGNQVFLFLENGIVVGREGSDALDAATGDVVFNISV
DGSGNVTLDQVRAVVHDNPLDPDESTGPTQLSAANLVTLTAIATDKDGDSASATANIGLSFNFEDDGP

>L7
VVDMAVKAGAALTLDETKGVKAGDANANDEAASADANDIGYAKLVGSDLFTLTKDAGSDGEQSTLFKLLVSAPASGLVDTATNQAIVLSANAGGTEVLGK
NTNGDVVFKVLLTASDGDVEVFQYRAIKHENASDHDESGAGGIIERIQAGSLKLEVTLTDKDGDSAKGELDLGQMMRFEDDGP

>L8
VAGTISLVADEDNLPRGNNDTASGDAAQSNLTGTLPVNFGADGAGSIDFQGMHGLSAVIGNDNITYNWNASTNTLTAYQTGGALGVNDVFKIVVNPTTGQ
YTFTLLAAINHHAVADNTEGLVDPFVNLNYRVIDGDGDTAIGTLKVTIDDDIP

>L9
KADAKAGAVAGAVLDESPAEGGLNNPVTIAASAISGLFETPDYGADGLGSVAYSLSGTDGAGTGLWLTGQSGAANEIRLVKVSDTEYEGRVGGAAGTLAF
EVTIDSNTGAVTVTQFATLEHTTDGSSAAAHDDALSLGGAAGIFVVQTVTDADGDTDAATSANALNISFEDDGP

>L10
SINASAAADSLTVDETTLGNDASANFADNFTSSFGEDGAGQIDYSLSVHEGVTGLVDTATNEAVVLSINNDGVVEGRTANSNDLVFTVTVDSAGTVTLDQ
LRAVVHSPNTGADQPTSLNAADLIQLTATITDKDGDSASAHLDLGQALNFKDDGP

>L11
TARTDVASITEGNLLTVTAAGGVLHNDTAGADNYAAGGSVVGVRAAGSDTTSAVSTGVNTAINGTYGTLIVQANGSYTYQANPNTDGQSDVFVYTIKDGD
GDLSTTTLTINLTDSGLSAPADNDVLVEERALDPAQDGADLAAGTVTGSLPNSTAETDASNQLNATGSTGPYTYSLVGAANGTYGVIQINADGTYTYTLT
KAYTTAPDANNGANTEDNRDSFTYQVTDAKGNTTTGTITVDIIDDVP

>Rs1
AIDLTSLAPTIAVDESVGTGGSTKDEPGGAAANDEAAAGAPAGAIGYAVTAAATLFSETTEPGSDGEDGTAYSLVLTAGATGLTDIATGSAVVLSLVGGV

VEGRSATGNHLVFTIAVASTTGNVTTTLYRALSHGADSNDHDSAVSMASGLVELQATLTDGDGDTGSDKIELGSLIDFEDDGP

>Rs2
DAVAKNVAGPTATLDESPLPTSGDGVNSVTIAATTIAALFETPDFGEDGQGSVSYTLSGTAGAQTGLWLTGESGAANEILLVKVSDTQWEGRKAGAGGTL
AFTVSINGTTGAVTVTRSAATLEHTVDGSSAAAHDDALTMAATANLFVVQHVTDGDGDTDSATATNPLTIKFEDDGP

>Rm0
TAVADTDAVAAGSFAPVAGNVMDGSGSDGNAAGADTKGADDATVVGVAAGNTNTDVDNAATLNTPIQGEYGKLTLHADGSYTYVRDAGTPGGVNDVFTYT
IKDGDGDTSHTTLTISIGNSTPEISDLTPEANGGDVIVNENDLLASRGPDESAGSDTSKESTTQGGTFTINSPDGIASLAIDGHTFITNGTFTGGSFTTA
LGNTLTVTGYDAGTGVVSYTYTLNDNEAHATAQDTNSLFENFTVTLTDEDGQSANGTLSVNIVDDVP
>Rm1
TASAEGSQNVAEGATLTGTLDFVAGADGATVTHIDGTLLVFGQDGYSQAIDIGDGSIKVKADGSYSFTADSPVNGTGAASATFTVTDGDGDTATAGISFA
VTDANTPTSGTAAASVDDDGLAGGNAASTTGDIAVPNTDGDNNEATFSGTLGGSLGGDGAGANGFSFATLNGTTGTVGQENVAYSWDAGTHTLTATTTSG
DRIGTALFTVHVTNPATGAYTVTLLDNVMHTGGPNNENNATVDLGYVITDADGSTAPGTLTITFNDDAP

>Rm2
SLTVGTVNESAITLVTQDAQTIGPASDTASASFAAAFLAAVTPSYGADGAGSTVISGYTLNVTNSASGLTSQGEAITLAKVGNDIVGSTASHGEIFRIAV
DANGTVTLTQSQQIDHLPESLDTTNNNAHIDLGSGLVTLSATATVTDGDHDQATSTVSTDLGGNIGFDDDIP

>Rm3
TISASGASASLTVDETVLLTNDTKAFASAFTSSYGADGAGAITYALGFNAGSTGLVDTLSGQAVVLSLEAGQVVGRAGAGGAIVFTVSTDASGNVTLDQQ
RAVVHPTSDPNEPVSLTADNLVTLTATITDKDGDSSAATLNIGQNLTFLDDGP

>Rm4
SVTVTAAPATKVALDETATSSSVATIDTGAIVKGDDPDVSGSGYISTATSVGALVTGNAAFGADGPAASGNTTYALTVTNTNSGLTLTDGSAIVLQQLAN
GTVVGVVSGGAFDGQAAFAMTINAATGAVMVEQYLSLDHPAEATAANSFNSYDETLTLASGSLGVTVTVTDGDHDMATSNTADVSNQITFDDDGP

>Rm5
TAHNDSGTQASENAAVTVNVFANDVPGADGVNIADPTKVSLVANSLTGSGTVVYHNDGTFTYTPAAGEQGTVTFQYQIVDGDGDPSVATVTINLLNDSTP
TIAIAGGSDTSVNEAGLPARGGEPAGSGEIADNNGTNNSDPSETATGSLNITTGGDTIGHLYVTDKNNVQVDVTNAAGGILVHGQYGDLTITGTPATGYT
YSYTLLDNTSGNTTHDDFAVQVVDSDGDPASTTLSISIVDDVP

>Rm6
TAHADTDAVQSGETVTGNVETGTSTHGTGAADTAGADGIASIAWTGIAGTTVTGTYGVLTVDANGGYSYHANPSASGVDIFNYTITDGDGDTSPSTLTIT
VSDGTPQPVAATVKVDEAALDTSTTGDDIDHGTATGTNPSSAAETVTGTLTLGDPDSPHVTNIAGATSSAVGGIPVTVQGTYGVLQIDQDGHYTYTLMTP
FTTAPATDNGAVTEPGKDVFTYTVTDSFGNSSTSTISVDIIDDVP

>Rm7
SVTASATQPPLTVDESNLGQNATASFASVFTPVFGADGAAAANSVTYALGFNAGATGLVDTATGQAVVLSLNGTVVEGRTAVSNELVFTVATDASGNVTL
DQQRAVVHPNAADPNDAKSLSADNLITLTATVTDKDGDTATATANIGQNLTFLDDGP

>Rm8
TAGIVQGAPTVAHDETAGIQADADDTTAPAVVALFAGVSNVSSDLSPTGYAQDPGAVVSSTGSSFGADQEGGTTAFSLAVSAAGVDSGLDTTAGTSIFLF
KEGDLVVGRIGGAAGAAAFAVAINSTTGVVSVAQYASIHHPTPGASHDETVNITDSALLAVVTVTDGDGDTATSSTGIGDAVQFQDDGP

>Rm9
TANLDTNSIASGQFGPVSGNVLANDVIGADGPATTSPAGGIVGVVAGTTAGGEDANVGTVIQGTYGKLTLNADGSYSYTRDAGTPGGHDDVFTYTIKDGD
GDLSHTTLTISIGNSTPTIGGLTPQAQGGDTTVNEKGLPASSGSEGSGELAAPGADGDPSEHNTGTFTISSPDGIAKLTINGTDISAAALANSGTTHVDV
TTPDGNTLTITGYDAGTGQVSYTYTLLDNETHSGAGIDSIFDNMTVTVTDTDGDASSGTLSVQIVDDVP

>Rm10
SIVPSGNDVPTLVVGDSDFVTDDSTSFAALFTPNFGVDGPAATDDVTYTLDVLGGGSNVASGVFDTLSGLEVLLNVVGGDIVGTAGTDEVFRISVDADGN
VTLDQSRAVVHGDPTDPVEADTPVILAANLVSLTATATDGDGDSAHTSVDIGGAFTFEDTGP

>Rm11
TISASTTNEPTLTVSEATLGTPSSGAFGGQFTVDYKADGPAASNPTTYALSTPGGDSGLIDTLTGQHVQLSKVGSQIVGTINNGTTTVFTVSVDGTGQVT
LTQSRAVVQATGSNPDTGEGIGLTGSNLVVLTATATDGDGDSASTPLDLTPQLQFTDVGP

>Rm12
SITASAINAPPLTVDETVLATDATGTFAAQFTPTFGADGQGATPVSYALSTPGGASGLTDTATGESVVLSLVNGQILGKTATTGLTVFVVSVDASGQVTL
DQQRAIVHANPNDPNESRGLTGSNLVVLTATATDGDGDHASAPLDLTPLLAFKDDGP

>Rm13
TAVLALAGKASIVVDESLGQNGAPETEPVGTLGQVTVLGTSLFDTTGSLAGQDEEGATTAFSLEVAAGGHTGLFLTGQSGAANEILLVDNGGVIEGRVGG
AGGTLAFTVSIDPSSGNVTLTQYESLNHPTSGASYDEALFLATGILKAVYTVTDGDKDVASASVDLGGRAGVIAFEDDGP

>Rm14
TAHADADAGIEGALIGGNVLTGIGTTGGVSGADVFGADGPTVAGGGVTGVATGSNTASPVTGNVGTPIVTALGTLTLNGDGSYTYQAKPNVVPAGGATDT
FVYTITDGDGDTSTVTLTINLSDSGITASNDAIVNEAGLSTGSDASSNSEIFATGQITASSAVPIVSYAITSVGGGTGSYGTLSLNPTTGAYTYTLTSRF
DTSPDADNATNTEQNRDVFTYTATDANGNTVTGTIEVDIIDDVP

>Rb1
SIALTGNVGSLNTFEAYLSAATNAGVNGSTPDAVPTQGHALDTESFAGAFTVVTGADGATTAYTLSIAGNGTATNLIDSASGLGVVLDQTGNTISGYVTG
HEGDAAWLVFTLAVNTATGDVTLTQDRAVHELTASSPDTGEGISLTGGLVTLTATVTDKDGDSAAQNLDLSSHVTFHDDGP

>Rb2
QTPTVTLAAFTAPVLLTFDGGLAGGNFTGTQDSHDTNASATIATEDFSGAFTIGNTSLYGADGAGATTINYTLGFHAGVADGVDSGLTHAGSTIYLYDVG
GVITGSTSTTAGGINAGNTAFTLSVDLTTGIVTLTQLESIDHSQTDTYNGSYLSDIKQIAANLIDLTASAFTTDSEGDKTVTASASVDVGHQIEFGDDGP

>Rb3
KAPTLTVSAATVGVDETPGVQTVGGASDVLGSTAITFNGAAATVAGLFATVANAGTDTDVSSAVLDNGALSFASTGAASILTASGGSYGADGAGTTVYAL
SVLNAASGLTLTDGTAITLSLDASGRIIGTVGADAANPSLTGKVAFAIAIDPATGQLYVAEYLSLHQNDTTNPNDVLTMAAGKVGA
TVTVTDSDGDHATSSADIGTHISFLDDGP

>Rb4
TAGTVTGPTDTLVLDETRPVGTDTAGGTAPTGLDTVTADFSNNFTPGSYGSDGPGHTTYTLNLVGANVASGLYALDATDTTTADGDGIGQGAQILLNQVG
NTITGSAGGTDYFTITIDPSTGIVTFTQLNNIWQSDPTNPDDAATLTLAGANLLQVVQTIVDADGDSVTTPLDVGTGVFTIQDSGP
>Rb5
AITMTATAPADALVVDETNLAINASANFADNFTVTSSYGADGAGTTASAYTLGIKSAGVESGLIDVATGQHVYLYVNGNVVEGRVGAGGAADPAGVIDFT
VSVSAAGVVTLDQVHALQHPNAANPDDVVTLSATDLITLTRTDTITDRDGDTTSGSASINIGQALSFHDDGP
>Rb6
SLGTPTAAVAGQLTLDETNLTGAPISTSANFAGNFDGGTPHYGTDGPGHVDYALALSANGTGSGLYALDPADVTTADNDGIGQGSQILLYQLADGSIVGK
VGAAADGSGGTLYFTIGVAANGDVTFTQDKNIWHSNTGNNDELASLTTAANTLLLNQTVTDADGDHVTKGIDLGSGVFAIHDDGP
>Rb7
SIHVVLDGEGEGQVLTNLVVDESNLTAVTNGIEGSIHNGINSAIGWFGGIFQANSGADGVADSEHPISYALSIGPSGTSGLVDAQTGEAVVLLMNGTTVE
GHTAASNLLVFTLSVDSSGFVTFTVDRSVKEADGTNPDTNEGISLSDGAVTLTATITDNDGDTATAGIDIGRQVTFLDDGP
>Rb8
TAHISLNESPTLIVDETDGVGAPGEVDPVGGNLGTNTILGATLFNNTSVFGSDGAASTTPTVYSLSLLSTVSGLTDTATGNAITLFLNGGVIEGHAGTLS
TDPLVFTLSINSSTGDTTLTQFRAVVHGTETDPDTSEAATLADNAVGVTVTVTDKDGDSSHDTANLNGVVKFLDDGP

>Vi1
YFTNVQGLYVHEDDLPQGSDTDKEPVTVNGQFQLVQGADTVASFALDSSVNPVQGLTSNGVAVTLSAPVDDGNGNLTYTAMAGSVTVFTLTLNXDGTYSF
TLAAPVDHALNSNDLTLNFKVIATDFDGDSDSIVLPVKINDDKP
>Vi2
TITNVDAIXVDEDDLXGIGSDQDGXSIDGKFTTNQGSDRVVSYQLDXSXXPVXGLTSXGXXVTLVETANADGSFTYTATANGNPVFTLVVNXDGSYNFTL
EGPIDHASNSDELTLNFPITATDFDGDTSSAVIPVTIVDDQP

>H1
AIALTSLAPTIAVDESLGTAGSSKDEPGNAAPDDETNANAPAGAIGYAVTTAATLFTETADAGTDGEASKVYALVLNAGASGLTDTASGQAVVLSDNSGVIEGRTDD
ANADLVFSIAVDATTGDVTTTQYRALDHGADSNDHDSAVSMAAGLVELEATLTDGDTDTASSKIELGSLIGFEDDGP

>H2
ADPTVALSGAEPVGLTFDGGLTDGNFTGTESGDDTSTSTPLVAVVDFSTAFTIGIQDDFGADGGDSTVVSYALQLATGVAEGDPSGLTSGGDPIRLYINDDGTLVTGSTDTSEGAGDGFTVFTLALNATTGVLTQTQSAVIDHGT
TDEYEGAYIVDVRELANGLVELVASAETTDSEGDTSGVNSEALDLGGNVRFGDDGP

>M1
SISTTGTEPTLTVDETVLATDDSKSFAANFSSAFGADGAGTLTYALSVVAGPSGLVDTATNQAVNLSLNGSVVEGRTAVSNDLVFTVSVDASGNVTLDQXRAVVHADXXDXDDAATLAADNLVKLTATVT
DKDGDHQAATLNIGQNLXFXDDGP

>M2
TLSITAAPVVGAAEVVEASGAGGQSQATITPPTFTAGAADGFTTNVTYALALAGASATGLLTTAGNHPITLVVDSANQISGKYDSDGNTTLDATAFTVTLSGTTVTLRSLVALEHSNAPQGVGEDNTLDLNGLINVVAT
VTVTDGDNDVVSSQSTSAGLSLTFDDTDP

Figure S2. List of all DDxP proteins analyzed. Proteins were labeled by a three-letter code denoting the genus. For each, Genbank ID, size in amino acids, name, place and source of isolation of the corresponding isolate are provided. Identical proteins are underlined. Boxed proteins are encoded by adjacent genes. Proteins reported in Figure 3 are marked by asterisks. Repeat highlighting and numbering of N and C regions is genus-specific. Proteins are classified as groups denote protein carrying (group-I) or lacking (group-II) RTX repeats) in the C region.

strain	source and place of isolation			protein	Genbank	aa	NH2	repeats																	COOH	group
Aeromonas																										
A. caviae NCTC12244	unknown		*	Aer-1	WP_167418739.1	4189	N1	A1	A1	A1	A1	A1	A1	A1	A1	A1	A1	A1	A2						C1	I
A. caviae GSH8M-1	wastewater treatment plant	Japan		Aer-2	WP_125117303.1	3683		A1	A1	A1	A1	A1	A1	A1	A1	A1	A2									
A. caviae R25-6	sludge from bioreactor	China		Aer-3	WP_113070082.1	3411		A1	A1	A1	A1	A1	A1	A1	A1	A2										
A. caviae YL12				Aer-4	WP_050498298.1	2665		A1	A1	A1	A1	A1	A2													
A. caviae GEO_39_Eff_A	water	USA	*	Aer-5	WP_128296818.1	1905		A1	A1	A2																
A. caviae GEO_47_Up_A	water	USA		Aer-6	WP_128271663.1	1886		A1	A1	A2																
A. caviae 429865	urine	Mexico		Aer-7	KOG94313.1	1395		A2																		
A. sp ASNIH1	waste water	USA		Aer-8	WP_104453085.1	2919		A1	A1	A1	A1	A1	A1	A2												
A. sp ASNIH2	waste water	USA		Aer-9	WP_104454347.1	4191		A1	A1	A1	A1	A1	A1	A1	A1	A1	A1	A1	A2							
A. sp ASNIH5	waste water	USA		Aer-10	WP_103242944.1	1652		A1	A2																	
A. salmonicida 023A	rock biofilm	Poland	*	Aer-11	ARW81648.1	3163	N2	A1	A1	A1	A1	A1	A1	A1											C2	
A. salmonicida J409	sablefish			Aer-12	WP_159368383.1	1896		A1	A1																	
A. salmonicida NBRC 13784	blood fish	Japan	*	Aer-13	GAJ50574.1	1643		A1																		
A. salmonicida AS03	crucian carp	South Korea		Aer-14	WP_152524362.1	1390		A1*																		
A. hydrophila GSH8-2	wastewater treatment plant	Japan	*	Aer-15	BBG84527.1	4594	N3	A3	A3	A3	A3	A3	A3	A3	A3	A3	A3	A3	A4	A4	A4	A4	A4	A4	C3	
A. hydrophila WCX23	diarrheal snake	China		Aer-16	WP_135325457.1	4592		A3	A3	A3	A3	A3	A3	A3	A3	A3	A3	A3	A4	A4	A4	A4	A4	A4		
A. hydrophila NCTC8049	unknown			Aer-17	WP_115586419.1	4267		A3	A3	A3	A3	A3	A3	A3	A3	A4	A4	A4	A4	A4	A4	A4				
A. hydrophila ATCC 7966	contaminated milk			Aer-18	WP_011706509.1	4266		A3	A3	A3	A3	A3	A3	A3	A4	A4	A4	A4	A4	A4	A4	A4				
A. hydrophila ZYAH75	human infection	China		Aer-19	WP_118880919.1	3702		A3	A4	A4	A4	A4	A4	A4	A4	A4	A4	A4	A4							
A. hydrophila D4	fish	China		Aer-20	WP_081051731.1	3391		A3	A3	A3	A3	A3	A4	A4	A4	A4	A4									
A. hydrophila JBN2301	crucian carp	China		Aer-21	WP_082189002.1	3391		A3	A3	A3	A3	A3	A4	A4	A4	A4	A4									
A. hydrophila ZYAH72	crucian carp	China		Aer-22	WP_118836200.1	3925		A3	A3	A3	A3	A3	A3	A3	A4	A4	A4	A4	A4							
A. hydrophila Ae34	Koi carp			Aer-23	WP_135354512.1	2885		A3	A3	A3	A3	A3	A3	A4												
A. hydrophila Arkansas 2010	catfish	USA		Aer-24	WP_141694518.1	2173		A3	A3	A4																
A. hydrophila AL09-79	catfish	USA		Aer-25	WP_155729455.1	2173		A3	A3	A4																
A. hydrophila GYK1	Siniperca chuatsi	China		Aer-26	WP_155770770.1	2173		A3	A3	A4																
A. hydrophila HX-3	Larimichthys crocea	China	*	Aer-27	WP_158197039.1	3367		A3	A3	A4	A4	A4	A4	A4	A4	A4	A4									
A. hydrophila MX16A	water	China		Aer-28	WP_123785028.1	4059		A3	A3	A3	A3	A3	A3	A3	A4	A4	A4	A4	A4	A4	A4	A4	A4		C1	
A. sp. CA23	lake water	Switzerland		Aer-29	WP_098983680.1	4073		A3	A3	A3	A3	A3	A3	A3	A3	A3	A3	A4	A4	A4	A4	A4	A4			
A. dhakensis KN-Mc-6U21	Myocastor coypus	Korea		Aer-30	WP_095592473.1	3897	N3	A3	A3	A3	A3	A3	A4	A4	A4	A4	A4	A4	A4	A4					C3	
A. dhakensis KOR1	Kandelia obovata	mangrove plant		Aer-31	WP_122975430.1	3796		A3	A3	A3	A3	A3	A3	A3	A3	A3	A3	A3	A3	A4						
A. hydrophila HX-3	Larimichthys crocea	China	*	Aer-32	WP_158198304.1	6271	N4	A5	A3	26 A6 repeats												A7			C4	
A. hydrophila 4AK4	unknown			Aer-33	WP_148297625.1	3369		A3	A3	A3	A3	A3	A3	A5	462 aa	A2	A2									
A. hydrophila AHNIH1	perirectal swab	homo sapiens	*	Aer-34	WP_147822853.1	3463		A3	A4	A4	A4	A4	A4	A4	A4	A4	A4	A15							C5	
A. hydrophila PAQ091014-9	*Oncorhynchus mykiss*	unknown		Aer-35	WP_139393764.1	2073		A3	A16									A15								
A. allosaccharophila Z9-6	chicken meat	China	*	Aer-36	WP_162626916.1	2486	N4	A10	A11				A11	A12	A13	A6	A6								C1	
A. allosaccharophila TTU2014-159ASC	unknown			Aer-37	WP_152986422.1	2801		A10	A11				A11	A12	A13	A6	A6	A6	A6							
A. sobria JF2635	Perca fluvialis	Switzerland	*	Aer-38	WP_157830651.1	3023		A10			A14	A14	A11	A12	A13	A6	A6	A6	A6							
A. sobria TM18	*Salvelinus fontinalis*			Aer-39	WP_157830800.1	2955		A10	A10	A10	A14	A14	A11	A12	A13	A6										
A. sobria TM19, TM12	*Salvelinus fontinalis*			Aer-40	WP_101346926.1	2750		A10	A10		A14	A14	A11	A12	A13	A6										
A. veronii AVNIH1	human-associated habitat	USA	*	Aer-41	WP_158512870.1	2993	N4	A5	A5	A5	A5	A5	A5	A3	A6	A6	A6	A7							C4	
A. veronii AVNIH 2	human-associated habitat	USA		Aer-42	KZW94267.1	2608		A10	A11				A11	A12	A13			A15							C5	
A. veronii B48	fish	Brazil	*	Aer-43	WP_158110690.1	2832		A5	A5				A11	A12	A13	A15	A15	A15								
A. veronii AK236	lake	France		Aer-44	WP_139731964.1	1553		A10	A7																C4	
A. encheleia NCTC12917	european eel	Spain	*	Aer-45	WP_126623349.1	3507	N5	A3	A8	A8	A8	A8	A8	A3	A9	A9	A6								C5	
Pseudomonas group-2																										
P. chlororaphis PCL1606	avocado rizhosphere	Spain		Pse-1	PCL1606_25380	2628		P1	P1	P1	P1	P1	P1	P1	P1	P1	P1	P1								
P. chlororaphis PA23	soybean root nodule	Canada		Pse-2	EY04_18075	1530		P1	P1	P1	P1															

P. chlororaphis PA23	soybean root nodule	Canada		Pse-3	EY04_18080	2165		P1	P1	P1	P1	P1	P1	P1	P1					
			*	Pse-4	WP_009044311.1	942		Rep-													
P.chlororaphis 30-84	wheat rizhosphere	USA	*	Pse-5	WP_009044312.1	1529		P1	P1	P1	P1										
			*	Pse-6	WP_152032113.1	2901		P1	P1	P1	P1	P1	P1	P1	P1	P1	P1	P1	P1	P1	
				Pse-7	JM49_12945	1554		P1	P1	P1	P1										C1
P.chlororaphis JD37	plant root	China		Pse-8	JM49_12940	1554		P1	P1	P1	P1										
				Pse-9	JM49_12935	2932		P1	P1	P1	P1	P1	P1	P1	P1	P1	P1	P1	P1	P1	
P.sp. MRSN12121	homo sapiens urine	USA		Pse-10	TO66_18930	2170		P1	P1	P1	P1	P1	P1	P1	P1						
P.sp.UW4				Pse-11	PputUW4_0419	2036		P2	P2	P2	P2	P2	P2								
P.nitroreducens DF05	soil	USA		Pse-12	WP_088417046.1	2001		P2	P2	P2	P2	P2	P2								
				Pse-13	WP_105344698.1	909		Rep-													
P.frederiksbergensis 11-D3	soil	China		Pse-14	WP_105344781.1	1977		P2	P2	P2	P2	P2	P2								
				Pse-15	WP_105344701.1	1361		P1	P1	P1											C2
P.lini DSM16768	rhizospheric soil	France	*	Pse-16	KMM95656.1	902		Rep-													
			*	Pse-17	KMM95655.1	1818	N1	P2	P2	P2	P2	P2	P2								
P.thivervalensis PITR2	wheat rizhosphere	Italy		Pse-18	WP_053185211.1	1391		P2	P2	P2											C1
P.fluorescens C8	soil	Denmark		Pse-19	KJZ43480.1	1376		P2	P2	P2											
P.frederiksbergensis KNU-15	farming soil	South Korea	*	Pse-20	WP_096481634.1	2018		P2	P2	P2	P2	P2	P2	P2							
			*	Pse-21	WP_096480329.1	1462		P1	P1	P1											C3	II
				Pse-22	WP_026331617.1	2018		P2	P2	P2	P2	P2	P2	P2							C1
P.mandelii 36MFCvi1.1	missing	missing		Pse-23	WP_033051309.1	1462		P1	P1	P1											C3
P.denitrificans ATCC 13867	missing	missing	*	Pse-24	WP_015477833.1	2131		P3	P3	P3	P3	P3	P3								C1
P.sp. Irchel s3a18	environmental	Zurich		Pse-25	WP_095149306.1	1860		P6	P6	P6	P5										C3
				Pse-26	WP_095149308.1	1405		P2	P2	P2											C1
P.fluorescens PA3G8	potato rhizosphere	France	*	Pse-27	WP_052248660.1	1447		P6	P6	P5											C2
			*	Pse-28	WP_038983706.1	904		Rep-													C1
P.lini BS3782	rhizospheric soil	France		Pse-29	WP_053078269.1	1447		P6	P6	P5											C2
				Pse-30	WP_048393634.1	902		Rep-													C1
				Pse-31	RON35734.1	1431		P6	P6	P5											C2
P.frederiksbergensis 94G2	soil	USA		Pse-32	RON35735.1	913		Rep-													C1
				Pse-33	RON35736.1	1982		P2	P2	P2	P2	P2	P2	P2							
P.sp. K2W31S-8	water (karst cave)	missing	*	Pse-34	WP_119892223.1	2098	N2	P4	P4	P4	P4	P4	P5								C3
P.sp. TCU-HL1	soil	Taiwan	*	Pse-35	WP_069085698.1	1555		P6	P6	P5											
P. fragi WS5045			*	Pse-36	WP_169910027.1	2050		P12	P13	P13	P13	P13	P13								
P. fragi NMC25				Pse-37	ARQ74978.1	1911		P12	P13	P13	P13	P13									
P. fragi NBRC 3458				Pse-38	WP_016781149.1	1710	N3	P12	P13	P13	P13										C4
Pseudomonas sp. OV144			*	Pse-39	TDU99816.1	2421		P12	P13	P13	P13	P13	P13	P13	P13						
Pseudomonas sp. FSL R10-0056				Pse-40	WP_178114911.1	1710		P12	P13	P13	P13										
Pseudomonas group-1																					
P.humi CCA1	leaf soil	Japan		Pse-41	WP_069864245.1	2477		P8	P7	P7	P7	P7	P7	P7							
P.citronellosis SYTE-3	sludge	China	*	Pse-42	WP_064582715.1	2297		P8	P7	P7	P7	P7	P7								
P.citronellosis P3B5	basil phyllosphere	Switzerland		Pse-43	WP_061562108.1	1939		P8	P7	P7	P7										
P.delhiensis 7361	fly ash dumping site	missing		Pse-44	WP_089394631.1	2121		P8	P7	P7	P7	P7									
P.sp. EGD-AKN5	soil	India		Pse-45	WP_058490182.1	2117		P8	P7	P7	P7	P7									
MULTIISPECIES *	river sediment	The Netherlands		Pse-46	WP_024128636.1	2117	N5	P8	P7	P7	P7	P7									C5
P.nitroriducens Aramco J	oil contaminated soil	Arabia	*	Pse-47	WP_052239091.1	3100		P8	P9	P9	P9	P9	P9	P9	P9	P9	P9	P9	P9		
P.sp. NBRC 111135	pharyngeal swab	Japan		Pse-48	WP_054908207.1	3100		P8	P9	P9	P9	P9	P9	P9	P9	P9	P9	P9	P9		
P.sp. 21	soil	USA		Pse-49	WP_082085846.1	3210		P8	P9	P9	P9	P9	P9	P9	P9	P9	P9	P9	P9		
P.sp. EaCA-3 231	Eisenia andrei symbiont	missing		Pse-50	PJI49538.1	3044		P8	P9	P9	P9	P9	P9	P9	P9	P9	P9	P9			
P.sp. AU11447	sputum	USA		Pse-51	WP_064979587.1	2943		P8	P9	P9	P9	P9	P9	P9	P9	P9	P9	P9			
P.denitrificans ATCC 13867	missing	missing		Pse-52	WP_162140827.1	3046		P8	P9	P9	P9	P9	P9	P9	P9	P9	P9	P9			
P. entomophila 2014	soil	China		Pse-53	WP_125465063.1	2371		P11	P11	P11	P11	P11									
P.parafulva CRS01-1	rice rhizosphere	China		Pse-54	WP_039583081.1	2405		P10	P11	P11	P11	P11									
P.mosselii Gil3	gills of channel catfish	USA		Pse-55	WP_028691107.1	2123		P10	P11	P11	P11										
P.soli SJ10	wastewater	South Korea		Pse-56	WP_038706594.1	2611		P10	P11	P11	P11	P11	P11								
P.putida PC2	*Pistacia chinensis*	*China*	*	Pse-57	WP_063543780.1	2384		P10	P11	P11	P11	P11									
P.putida W5	Medicago sativa	Canada		Pse-58	WP_159411598.1	2621	N6	P10	P11	P11	P11	P11	P11								C6
P.putida AA7	Zea mais	USA		Pse-59	WP_079229229.1	3116		P10	P10	P10	P11	P11	P11	P11	P11						
P.putida CBF10-2	soil	USA		Pse-60	WP_081263106.1	3338		P10	P10	P10	P10	P10	P10	P10	P10	P11					
P.frederiksbergensis KNU-15	farming soil	South Korea	*	Pse-61	WP_096479694.1	2385		P10	P10	P10	P11	P11	P11								
P.mandelii 36MFCvi1.1	missing	missing		Pse-62	WP_019580760.1	2379		P10	P10	P10	P11	P11	P11									I

P.sagittaria JCM 18195	oil contaminated soil	type st	*	Pse-63	SFP88769.1	2301	N7	P15 P15 P15 P15 P15 P15	C7	
Pseudomonas mendocina NCTC10898	freshwater	unknown	*	Pse-64	WP_147285553.1	3984	N8	P16 P17 P17 P17 P18 P19 P20 P20 P20 P20 P20 P20 P21	C8	
Pseudomonas mendocina S5.2	rhizosphere soil	France		Pse-65	WP_026042120.1	3984		P16 P17 P17 P17 P18 P19 P20 P20 P20 P20 P20 P20 P21		
Pseudomonas mendocina NK-01	farmland soil	China		Pse-66	WP_013716144.1	3977		P16 P17 P17 P17 P18 P19 P20 P20 P20 P20 P20 P20 P21		
P.oryzae KCTC 32247	paddy soil	China	*	Pse-67	WP_157719523.1	3129	N9	P22 P22 P22 P23 P23 P23 P23 P23 P23 P23	C9	
Vibrio										
V.cholerae vm1	India	homo sapiens	*	Vib-1	WP_119299450.1	4416	N1	Vi1 Vi1 Vi1 Vi1 Vi1 Vi1 Vi1 Vi1 Vi1 Vi1 Vi1 Vi1	C1	
V.cholerae 2011EL-1271	nd	nd		Vib-2	WP_123161736.1	4127		Vi1 Vi1 Vi1 Vi1 Vi1 Vi1 Vi1 Vi1 Vi1 Vi1		
V.cholerae Vn-2923	Germany	coastal water		Vib-3	WP_118133744.1	3839		Vi1 Vi1 Vi1 Vi1 Vi1 Vi1 Vi1 Vi1		
V.cholerae 981-75	India	homo sapiens	*	Vib-4	WP_032473928.1	3263		Vi1 Vi1 Vi1 Vi1		
Vibrio metoecus OYP8G09	oyster pond	USA		Vib-5	WP_095463043.1	4126		Vi1 Vi1 Vi1 Vi1 Vi1 Vi1 Vi1 Vi1 Vi1 Vi1		
Vibrio mimicus NCTC11435	ear homo sapiens	USA		Vib-6	WP_115236305.1	4125		Vi1 Vi1 Vi1 Vi1 Vi1 Vi1 Vi1 Vi1 Vi1 Vi1		
V. parahaemolyticus UCM-V493	sediment sample	Spain	*	Vib-7	WP_025442669.1	6084	N2	Vi2 Vi2 Vi2 Vi2 Vi2 8 more Vi2 Vi2 Vi2 Vi2b Vi2b Vi2 Vi2b Vi2b Vi2	C2	
V. parahaemolyticus SNUVpS-1	sea food	South Korea		Vib-8	WP_017448122.1	6084		Vi2 Vi2 Vi2 Vi2 Vi2 8 more Vi2 Vi2 Vi2 Vi2b Vi2b Vi2 Vi2b Vi2b Vi2		
V. parahaemolyticus B2_7	fish	China	*	Vib-9	WP_140188058.1	5227		Vi2 Vi2 Vi2 Vi2 Vi2 Vi2 Vi2 Vi2 Vi2 Vi2 Vi2 Vi2 Vi2 Vi2 Vi2		
V. parahaemolyticus 2013V-1244	oral swab	USA		Vib-10	WP_089183625.1	5227		Vi2 Vi2 Vi2 Vi2 Vi2 Vi2 Vi2 Vi2 Vi2 Vi2 Vi2 Vi2 Vi2 Vi2 Vi2		
Sinhorizobium										
Sinhorizobium americanum CFNEI 73	Vachellia farnesiana	Mexico	*	Rhi-1	WP_064253643.1	1368	N1	Rs1 Rs1 Rs1	C1	II
Sinhorizobium sp. NFACC03	unknown			Rhi-2	WP_093233261.1	1373		Rs1 Rs1 Rs1		
Rhizobiales bacterium ERS2527895	corn			Rhi-3	WP_113512315.1	1001		Rs2		
Rhizobiales bacterium ERS2527920	corn		*	Rhi-4	WP_113478518.1	1001		Rs2		
Rhizobiales bacterium AFS034408	unknown		*	Rhi-5	WP_113174696.1	824		Rep-		
Ensifer adhaerens M78	purple siltstone	China		Rhi-6	WP_065375910.1	1357		Rs2 Rs2 Rs2		
Sinhorizobium fredii NXT3 (plasmid pSfreNXT3a)	Phaseoulus vulgaris	Mexico		Rhi-7	WP_104840299.1	1355		Rs2 Rs2 Rs2		
Sinhorizobium americanum CCGM7	Phaseoulus vulgaris	Mexico	*	Rhi-8	WP_072597184.1	1362		Rs2 Rs2 Rs2		
			*	Rhi-9	WP_072597182.1	829		Rep-		
Rhizobium giardinii H152	Phaseolus vulgaris	France		Rhi-10	WP_018325016.1	826		Rep-		
Rhizobium loessense CGMCC 1.3401	unknown	unknown		Rhi-11	WP_092586626.1	823		Rep-		
Ensifer sp. MPMI2T	pigeon pea	india		Rhi-12	WP_136508763.1	822		Rep-		
Mesorhizobium										
Mesorhizobium M4B.F.Ca.ET.058.02.1.1	chickpea	Amhara, Ethiopia	*	Rhi-13	WP_126056483.1	3106	N2	Rm 0 Rm 1 Rm 1 Rm2 Rm2 Rm2 Rm2 Rm2 Rm2 Rm2 Rm2	C2	I
Mesorhizobium M1E.F.Ca.ET.045.02.1.1	chickpea	Amhara, Ethiopia	*	Rhi-14	WP_126092540.1	2366		Rm 0 Rm 1 Rm 1 Rm3 Rm3 Rm3 Rm3		
Mesorhizobium N.Ca.ET.122.02.1	chickpea	Amhara, Ethiopia		Rhi-15	RWB55351	1897		Rm 0 Rm 1 Rm 1 Rm3		
Mesorhizobium N.Ca.ET.039.01.1	chickpea	Amhara, Ethiopia		Rhi-16	RWD73564	1924		Rm 0 Rm 1 Rm 1 Rm3		
Mesorhizobium N.Ca.ET.113.01.1	chickpea	Amhara, Ethiopia		Rhi-17	RWB21180.1	2136		Rm 0 Rm 1 Rm 1 Rm4 Rm4		
Mesorhizobium tamadyense DSM28320	unknown	Spain		Rhi-18	WP_124997996.1	2285		Rm 0 Rm 1 Rm 1 Rm2 Rm2 Rm4		
Mesorhizobium amorphae CCNWGS0123	Robinia pesudoacacia	China		Rhi-19	WP_065518171.1	2802		Rm 0 Rm 1 Rm 1 Rm3 Rm3 Rm3 Rm3 Rm3 Rm3 Rm3		
Mesorhizobium M1D.F.Ca.ET.043.01.1.1	chickpea	Amhara, Ethiopia	*	Rhi-20	WP_126085826.1	2671		Rm 0 Rm 1 Rm 1 Rm2 Rm2 Rm2 Rm2 Rm2 Rm4	C3	
Mesorhizobium N.Ca.ET.109.02.1	chickpea	Tigray, Ethiopia		Rhi-21	RWA89033.1	1983		Rm 0 Rm 1 Rm 1 Rm2 Rm4		
Mesorhizobium N.Ca.IN.005.06.1	chickpea	Varanasi India		Rhi-22	RWK34869.1	1978		Rm 0 Rm 1 Rm 1 Rm2 Rm4		
Mesorhizobium USDA-HM6	chickpea	-		Rhi-23	RUU11243.1	1794		Rm 0 Rm 1 Rm2 Rm4	C4	
Mesorhizobium N.Ca.ET.018.01.1	chickpea	Oronia, Ethiopia	*	Rhi-24	RWC29680.1	2127		Rm5 Rm5 Rm6 Rm7 Rm7 Rm4		
Mesorhizobium N.Ca.ET.013.01.1	chickpea	Oronia, Ethiopia		Rhi-25	TIX28404.1	1878		Rm5 — Rm6 Rm7 Rm7 Rm4		
Mesorhizobium M2A.F.Ca.ET.043.05.1.1	chickpea	Amhara, Ethiopia	*	Rhi-26	WP_126062539.1	2926	N3	Rm5 Rm5 Rm5 Rm5 Rm5 Rm5 Rm5 Rm5	C5	
Mesorhizobium waimense ICMP19557	Sophora longicarinata	New Zealand		Rhi-27	WP_120013116.1	1013	N4	Rm8	C6	
Mesorhizobium tamadayense DSM 28320	root nodule	Spain		Rhi-28	WP_125001042.1	1200		Rm8 Rm8		
Mesorhizobium sophorae ICMP 19535	Sophora microphylla	New Zealand	*	Rhi-29	WP_095084448.1	1661		Rm8 Rm8 Rm8 Rm8	C7	
Mesorhizobium M3A.F.Ca.ET.080.04.2.1	chickpea	Wollo, Ethiopia	*	Rhi-30	WP_129553402.1	2773	N3	Rm 9 Rm 9 Rm 9 Rm 9 Rm 9 Rm 9	C8	I
Mesorhizobium sp. LNJC395A00	*chile lotus*	USA	*	Rhi-31	ESY26103.1	935	N5	Rm10	C9	II
Mesorhizobium sp. M00.F.Ca.ET.186.01.1.1	chickpea	Oronia, Ethiopia		Rhi-32	TGV40215.1	934		Rm10		
Mesorhizobium sp.	chickpea	Jharkhand, India		Rhi-33	RWH69293.1	1002	N6	Rm11	C10	
Mesorhizobium sp.	chickpea	Jharkhand, India		Rhi-34	RWL45713.1	972		Rm11		
Mesorhizobium sp.	chickpea	Jharkhand, India		Rhi-35	RWL58406.1	1162		Rm11 Rm11		
Mesorhizobium plurifarium ORS1032T	Senegalia senegal	Senegal	*	Rhi-36	WP_073986818.1	999		Rm11		

Mesorhizobium plurifarium STM8773	Acacia senegal	Senegal	*	Rhi-37	WP_040987538.1	699		Rep-		
Mesorhizobium sp. WSM1497	Biserrula pelecinus	Greece		Rhi-38	WP_064994388.1	754	N7	Rep-	C11	
Mesorhizobium unclassified multispecies	chickpea	India		Rhi-39	WP_127239580.1	750		Rep-		
Mesorhizobium plurifarium ORS3365	Vachelllia seyal	Senegal	*	Rhi-40	WP_052468903.1	1073	N8	Rm12 Rm12	C12	
Mesorhizobium sp. Pch-S	Paulinella (micro alga)	South Korea	*	Rhi-41	WP_129411922.1	4828	N9	Rm13 Rm13 Rm13 Rm13 Rm13 Rm13 457 aa Rm14 Rm14 Rm14 Rm14 Rm14 Rm14	C13	
Bradyrhizobium										
Bradyrhizobium macuxiense BR10355	unknown		*	Rhi-42	WP_146992040.1	3522		Rb1 Rb1 Rb1 Rb1 Rb1 Rb1 Rb1 Rb1 Rb1 Rb1 Rb1 Rb1 Rb1 Rb1 Rb1	C14	
Bradyrhizobium tropiciagri SEMIA 6148	perennial soybean	Africa		Rhi-43	WP_050424423.1	3328		Rb1 Rb1 Rb1 Rb1 Rb1 Rb1 Rb1 Rb1 Rb1 Rb1 Rb1 Rb1 Rb1 Rb1		
Bradyrhizobium sp. UFLA03-84	black eyed pea	Brasil	*	Rhi-44	WP_095733983.1	3456		Rb2 Rb2 Rb2 Rb2 Rb2 Rb2 Rb2 Rb2 Rb2 Rb2 Rb2 Rb2 Rb3	C15	
Bradyrhizobium sp. th.b2	plant root			Rhi-45	WP_029080584.1	2427		Rb2 Rb2 Rb2 Rb2 Rb2 Rb2 Rb2 Rb3		
Bradyrhizobium elkanii SEMIA 938	Lupinus albus	Brasil	*	Rhi-46	WP_137476231.1	3231		Rb4 Rb4 Rb4 Rb4 Rb4 Rb4 Rb4 Rb4 Rb4 Rb4 Rb4 Rb4 Rb3		
B. sp. R5	unknown			Rhi-47	WP_092089788.1	3222	N10	Rb4 Rb4 Rb4 Rb4 Rb4 Rb4 Rb4 Rb4 Rb4 Rb4 Rb4 Rb4 Rb3	C16	I
Bradyrhizobium elkanii BLY3-8	soybean	Myanmar		Rhi-48	WP_069277524.1	3240		Rb4 Rb4 Rb4 Rb4 Rb4 Rb4 Rb4 Rb4 Rb4 Rb4 Rb4 Rb4 Rb3		
B. pachyrhizi BR3262	cowpea	Brasil		Rhi-49	WP_057017072.1	3222		Rb4 Rb4 Rb4 Rb4 Rb4 Rb4 Rb4 Rb4 Rb4 Rb4 Rb4 Rb4 Rb3		
B. sp. UFLA 03-321	black eyed pea	Brasil	*	Rhi-50	OMI11765.1	1550		Rb5 Rb6 Rb6 Rb3		
Bradyrhizobium stylosanthis BR 446	S. guianensis	Brasil	*	Rhi-51	WP_063694591.1	3938		Rb7 Rb7 Rb8 Rb8 Rb8 Rb8 Rb8 Rb8 Rb8 Rb8 Rb8 Rb8 Rb8 Rb8 Rb8 Rb8 Rb5	C17	
Bradyrhizobium stylosanthis BR 510	S. guianensis	Brasil		Rhi-52	WP_145662683.1	3936		Rb7 Rb7 Rb8 Rb8 Rb8 Rb8 Rb8 Rb8 Rb8 Rb8 Rb8 Rb8 Rb8 Rb8 Rb8 Rb8 Rb5		
Multispecies										
Rhodospirillales bacterium URHD0017	nd	nd	*	Mus-1	WP_092826367.1	1185		Mu1 Mu1		
Methylobacterium sp. 4-46	Lotononis bainesii legume		*	Mus-2	WP_012332371.1	1338		Mu1 Mu1 Mu1		
Bradyrhizobium ivorense CI-1B	pigeonpea root nodule	Cote d'Ivoire	*	Mus-3	WP_175607160.1	1481		Mu1 Mu1 Mu1 Mu1		
Rhizobiales bacterium AFS031577	corn	nd		Mus-10	WP_113133853.1	1059		M2		
Rhizobiales bacterium AFS096089	corn	nd	*	Mus-11	WP_112948475.1	1204		M2 M2		
Rhizobiales bacterium AFS054759	corn	nd		Mus-12	WP_113478464.1	1223		M2 M2		
Ensifer sp. AP48	nd	nd		Mus-13	WP_110881766.1	1372		M2 M2 M2		
Ensifer adhaerens M78	purple siltstone	China	*	Mus-14	WP_065377507.1	1373	N11	M2 M2 M2	C18	II
Bradyrhizobium sp. cir1	nd	nd	*	Mus-15	WP_183245945.1	1196		M2 M2		
Sphingobium barthaii KK22 24_6	nd	nd		Mus-16	WP_133654628.1	1204		M2 M2		
Sphingomonas sediminicola KACC 15039	freshwater sediment	Sout Korea		Mus-17	WP_187708106.1	1294		M2 M2		
Sphingomonas sp. CDS-1		China	*	Mus-18	WP_087575419.1	1379		M2 M2 M2		
Sphingobium sp. TKS	soil	Japan		Mus-19	WP_145907593.1	1379		M2 M2 M2		
Sphingobium sp. TB-6	soil	Sout Korea		Mus-20	WP_169576360.1	1379		M2 M2 M2		
Sphingobium sp. 15-1	soil	Sout Korea		Mus-21	WP_176595181.1	1379		M2 M2 M2		
Nitrosospira, Nitrosomonas										
Nitrosospira sp. NpAV	soil	USA	*	Nit-1	WP_084226822.1	1171		N1 N2		
Nitrosospira sp. Nsp13	unknown	unknown		Nit-2	SCY49192.1	1189		N1 N2		
Nitrosospira multiformis Nl4	unknown	unknown		Nit-3	WP_081350755.1	1215		N1 N1		
Nitrosomonas ureae Nm13	unknown	unknown	*	Nit-4	SEF55012.1	1329	N1	N1 N2 N2	C1	
Nitrosospira sp. Nsp44	unknown	unknown		Nit-5	WP_121084721.1	1336		N1 N1 N2		
Nitrosomonas sp.Nm58	unknown	unknown	*	Nit-6	WP_090575218.1	1401		N3 N3 N3		
Nitrosomonas sp. Nm132	unknown	unknown		Nit-7	WP_090540268.1	1211		N3 N3		
Variovorax										II
Variovorax boronicumulans J1	soil	China	*	Var-1	WP_095744550.1	1701		Va1 Va1 Va1 Va1		
Acidovorax cavernicola 1011MAR4D40.2	cave wall	Spain		Var-2	RIX75831.1	1508	N1	Va1 Va1 Va1	C1	
			*	Var-3	AGU49274.1	1871		Va1 Va1 Va1 Va1 Va1		
Variovorax paradoxus B4	soil	Germany	*	Var-4	AGU49286.1	1734	N2	Va2 Va2 Va2 Va2 Va2	C2	
Variovorax sp. 502	maize root	China		Var-5	WP_125964858.1	1218		Va2 Va2		
Halomonas										
Halomonas salina B6	human gut	USA		Hal-1	WP_040184700.1	2139	N1	H1 H1 H1 H1 H1 H1 H1 H1	C1	
Halomonas aestuarii Hb3	salt filed	South Korea		Hal-2	BOX17_03680	2524		H2 H2 H2 H2 H2 H2 H2 H2 H2		

Figure S3. Sequences of the DDxP proteins shown in Figures 1 and 3. N, C and repeat regions are in blu, red and black, respectively. Multiple DDxP repeats within each protein were aligned to highlight sequence variations.

Leg-1 APF02469.1

```
MLAESVIGIVRAVNGLLEKVNVQGQASLVKSGARLEEGDVLTLLSGEAYIQFINGFPEALALEKPVKLDGVSPTLQYGAEDLKEQMVQEAIAKGIDPSVI
LDVLGSAAAGAEAVGSGGDAFIMDPLFGFGHVTAGYPTGPISFAYEADTQQLFWFVPEETGAIAESELTQEPESIPQIPQFTTNQAVLTVFEDALPSGIA
DSAGQARIASSSLSSLLTSSADVAASFAFNSNLSALPTLKSGGIDLSYELSADKRTLTLRESSTQGPGAEVMKLELTADGQLTQTLMNSIDHPAADSDG
EWMRFDLSPLIDVTFTRTSNGTVLESRTLPANAVVAGIQDDVPIARAQLTNNEILLDETIGIKVGDADAANDDFNPTTTVDPFNNIYGTPLGLVQNANLL
DASTSEMGGDYKNATMTHLLKITDAVSGLQTTDGTPINLFLESNGDITGRAGDVGAPAVFAIRMNPNTGAITVAQYGSIKQFDTSSYDEAVDLTGRISAV
VTAKDSDGDVSNVEIPIGQLIIFEDDGP
```

L1 repeats

```
SVTMTVSDNNAITLNTQDADTIGGLSDSDSASFAAAFAVTPNYGADGAGTTVTTYALSVSAQGVDSGLDNNGNNIYLYNIAGSVVGSTSATQAGITTGNT
SVMMTVSDNNVITLNTQDADTIGAASDSDSASFAAAFAVTPNYGADGAGTTVTTYALSVSAQGVDSGLDNNGNDIYLYKIGDSVVGSTSATQAGIVTGNT
SVTMTVSDNNVITLNTQDADTIGGLSDSDSASFATAFAVTPNYGADGAGTTVTTYALSVSAQGVDSGLDNNGNDIYLYKIGDSVVGSTSATQAGVVAGNT
SVTMTVSDNNVITLNTQDADTIGGLSDSDSASFATAFAVTPNYGADGAGTTVTTYALSVSAQGVDSGLDNNGNNIYLYNIAGSVVGSTSATQAGITTGNT
SVTMTVSDTNAITLNTQDADTIGAASDSDSVSFAAAFAVTPNYGADGAGTTVTTYALSVSAQGVDSGLDNNGNNIYLYNIAGSVVGSTSATQAGITTGNT
SVTMTVSDTNAITLNTQDADTIGAASDSDSVSFAAAFAVTPNYGADGAGTTVTTYALSVSAQGVDSGLDNNGNNIYLYNIAGSVVGSTSATQAGITTGNT
SVTMTVSDNNVITLNTQDADTIGGLSDSDSASFAAAFAVTPNYGADGAGTTVTTYALSVSAQGVDSGLDNNGNDIYLYKIGDSVVGSTSATQAGITTGNT
SVTMTVSDNNVITLNTQDADTIGAASDSDSVSFAAAFAVTPNYGADGAGTTVTTYALSVSAQGVDSGLDNNGNNIYLYNIAGSVVGSTSATQAGITTGNT
SVTMTVSDNNVITLNTQDADTIGAASDSDSVSFAAAFAVTPNYGADGAGTTVTTYALSVSAQGVDSGLDNNGNNIYLYNIAGSVVGSTSATQAGITTGNT
SVTMTVSDNNVITLNTQDADTIGAASDSDSVSFAAAFAVTPNYGADGAGTTVTTYALSVSAQGVDSGLDNNGNNIYLYNIAGSVVGSTSATQAGITTGNT
SVMMTVSDNNVITLNTQDADTIGAASDSDSASFAAAFAVTPNYGADGAGTTVTTYALSVSAQGVDSGLDNNGNNIYLYNIAGSVVGSTSATQAGITTGNT
SVTMTVSDNNVITLNTQDADTIGAASDSDSASFAAAFAVTPNYGADGAGTTVTTYALSVSAQGVDSGLDNNGNNIYLYNIAGSVVGSTSATQAGITTGNT
SVTMTVSDNNVITLNTQDADTIGAASDSDSVSFAAAFAVTPNYGADGAGTTVTTYALSVSAQGVDSGLDNNGNNIYLYNIAGSVVGSTSATQAGITTGNT
SVTMTVSDNNVITLNTQDADTIGGLSDSDSASFAAAFAVTPNYGADGAGTTVTTYALSVSAQGVDSGLDNNGNDIYLYKIGDSVVGSTSATQAGVVAGNT
SVTMTVSDNNVITLNTQDADTIGAASDSDSVSFAAAFAVTPNYGADGAGTTVTTYALSVSAQGVDSGLDNNGNNIYLYNIAGSVVGSTSATQAGITTGNT
SVTMTVSDTNAITLNTQDADTIGAASDSDSVSFAAAFAVTPNYGADGAGTTVTTYALSVSAQGVDSGLDNNGNNIYLYNIAGSVVGSTSATQAGITTGNT
SVTMTVSDNNVITLNTQDADTIGGLSDSDSASFAAAFAVTPNYGADGAGTTVTTYALSVSAQGVDSGLDNNGNDIYLYKIGDSVVGSTSATQAGITTGNT
SVTMTVSDNNVITLNTQDADTIGAASDSDSVSFAAAFAVTPNYGADGAGTTVTTYALSVSAQGVDSGLDNNGNNIYLYNIAGSVVGSTSATQAGITTGNT
SVTMTVSDNNVITLNTQDADTIGAASDSDSVSFAAAFAVTPNYGADGAGTTVTTYALSVSAQGVDSGLDNNGNNIYLYNIAGSVVGSTSATQAGITTGNT
SVTMTVSDNNVITLNTQDADTIGAASDSDSVSFAAAFAVTPNYGADGAGTTVTTYALSVSAQGVDSGLDNNGNNIYLYNIAGSVVGSTSATQAGITTGNT
SVMMTVSDNNVITLNTQDADTIGAASDSDSASFAAAFAVTPNYGADGAGTTVTTYALSVSAQGVDSGLDNNGNNIYLYNIAGSVVGSTSATQAGITTGNT
SVTMTVSDNNVITLNTQDADTIGAASDSDSASFAAAFAVTPNYGADGAGTTVTTYALSVSAQGVDSGLDNNGNNIYLYNIAGSVVGSTSATQAGITTGNT
SVTMTVSDNNVITLNTQDADTIGAASDSDSASFAAAFAVTPNYGADGAGTTVTTYALSVSAQGVDSGLDNNGNNIYLYNIAGSVVGSTSATQAGITTGNT
SVTMTVSDTNAITLNTQDADTIGAASDSDSASFAAAFAVTPNYGADGAGTTVTTYALSVSAQGVDSGLDNNGNNIYLYNIAGSVVGSTSATQAGITTGNT
SVTMTVSDNNVITLNTQDADTIGAASDSDSASFAAAFAVTPNYGADGAGTTVTTYALSVSAQGVDSGLDNNGNNIYLYNIAGSVVGSTSATQAGITTGNT
SVTMTVSDNNVITLNTQDADTIGAASDSDSASFAAAFAVTPNYGADGAGTTVTTYALSVSAQGVDSGLDNNGNNIYLYNIAGSVVGSTSATQAGITTGNT
SVTMTVSDNNVITLNTQDADTIGAASDSDSVSFAAAFAVTPNYGADGAGTTVTTYALSVSAQGVDSGLDNNGNNIYLYNIAGSVVGSTSATQAGITTGNT

IFSLGVNSSSGVVTLTQHQEVDHGLPGASSNYAAQEAILNTGLVFLNATAVTTDRDGDTATASASLDLGGNVKFDDDGP
IFSLGVNSSNAMLTLTQHQEVDHGLPGAGSNYAAQEAILNTGLVFLNATAVTTDRDGDTATASASLDLGGNVKFDDDGP
IFSLGVNSSNAMLTLTQHQEVDHGLPGAGSNYAAQEAILNTGLVFLNATAVTTDGDGDTATASASLDLGGNVKFDDDGP
IFSLGVNSSSGVVTLTQHQEVDHGLPGASSNYAAQEAILNTGLVFLNATAVTTDGDGDTATASASLDLGGNVKFDDDGP
IFSLGVNSSSGVVTLTQHQEVDHGLPGASSNYAAQEAILNTGLVFLNATAVTTDGDGDTATASASLDLGGNVKFDDDGP
IFSLGVNSSSGVVTLTQHQEVDHGLPGASSNYAAQEAILNTGLVFLNATAVTTDGDGDTATASASLDLGGNVKFDDDGP
IFSLGVNSSSGVVTLTQHQEVDHGLPGASSNYAAQEAILNTGLVFLNATAVTTDRDGDTATASASLDLGGNVKFDDDGP
IFSLGVNSSSGVVTLTQHQEVDHGLPGASSNYAAQEAILNTGLVFLNATAVTTDGDGDTATASASLDLGGNVKFDDDGP
IFSLGVNSSSGVVTLTQHQEVDHGLPGASSNYAAQEAILNTGLVFLNATAVTTDGDGDTATASASLDLGGNVKFDDDGP
IFSLGVNSSSGVVTLTQHQEVDHGLPGASSNYAAQEAILNTGLVFLNATAVTTDGDGDTATASASLDLGGNVKFDDDGP
IFSLGVNSSSGVVTLTQHQEVDHGLPGASSNYAAQEAILNTGLVFLNATAVTTDGDGDTATASASLDLGGNVKFDDDGP
IFSLGVNSSSGVVTLTQHQEVDHGLPGASSNYAAQEAILNTGLVFLNATAVTTDGDGDTATASASLDLGGNVKFDDDGP
IFSLGVNSSSGVVTLTQHQEVDHGLPGASSNYAAQEAILNTGLVFLNATAVTTDGDGDTATASASLDLGGNVKFDDDGP
IFSLGVNSSSGVVTLTQHQEVDHGLPGASSNYAAQEAILNTGLVFLNATAVTTDGDGDTATASASLDLGGNVKFDDDGP
IFSLGVNSSNAMLTLTQHQEVDHGLPGAGSNYAAQEAILNTGLVFLNATAVTTDRDGDTATASASLDLGGNVKFDDDGP
IFSLGVNSSSGVVTLTQHQEVDHGLPGASSNYAAQEAILNTGLVFLNATAVTTDGDGDTATASASLDLGGNVKFDDDGP
IFSLGVNSSSGVVTLTQHQEVDHGLPGASSNYAAQEAILNTGLVFLNATAVTTDGDGDTATASASLDLGGNVKFDDDGP
IFSLGVNSSSGVVTLTQHQEVDHGLPGASSNYAAQEAILNTGLVFLNATAVTTDRDGDTATASASLDLGGNVKFDDDGP
IFSLGVNSSSGVVTLTQHQEVDHGLPGASSNYAAQEAILNTGLVFLNATAVTTDGDGDTATASASLDLGGNVKFDDDGP
IFSLGVNSSSGVVTLTQHQEVDHGLPGASSNYAAQEAILNTGLVFLNATAVTTDGDGDTATASASLDLGGNVKFDDDGP
IFSLGVNSSSGVVTLTQHQEVDHGLPGASSNYAAQEAILNTGLVFLNATAVTTDGDGDTATASASLDLGGNVKFDDDGP
IFSLGVNSSSGVVTLTQHQEVDHGLPGASSNYAAQEAILNTGLVFLNATAVTTDGDGDTATASASLDLGGNVKFDDDGP
IFSLGVNSSSGVVTLTQHQEVDHGLPGASSNYAAQEAILNTGLVFLNATAVTTDGDGDTATASASLDLGGNVKFDDDGP
IFSLGVNSSSGVVTLTQHQEVDHGLPGASSNYAAQEAILNTGLVFLNATAVTTDGDGDTATASASLDLGGNVKFDDDGP
IFSLGVNSSSGVVTLTQHQEVDHGLPGASSNYAAQEAILNTGLVFLNATAVTTDGDGDTATASASLDLGGNVKFDDDGP
IFSLGVNSSSGVVTLTQHQEVDHGLPGASSNYAAQEAILNTGLVFLNATAVTTDGDGDTATASASLDLGGNVKFDDDGP
IFSLGVNSSSGVVTLTQHQEVDHGLPGASSNYAAQEAILNTGLVFLNATAVTTDGDGDTATASASLDLGGNVKFDDDGP

VASNITRSGLANEGADTNLMLILDTSGSMAGSGIQTLINSTLELLERYEALGNVKVRIVTFNTSATAIGSVWMTVDAAKNALLGLTAGGNTNFDAALITA
MNAFNSGTVDGADGRIGGAQNVSYFISDGNPTVNQDWPSIPGTLAQNGIQPSEQAAWEAFLQDTNGASAGGEKIRSYALGMGTGITVSNLEPIGFNGVNG
TDIPAVVVTDISQLDSVLASTITASPISGTLISDPNPGVNFGADGGHVQSVTVNGITYTVNANLSSLSTSGSATQGSNTHSYDNSTHVLTVTISTGESLT
IDMDNGSYTFTPPANITSSINRIINYVLVDGDGDTAAATITFNIDISGFNDPLVVRDDLVLTNQPAVSGQDVISIPTWALLANDTGGSGVSSVSSVTQPV
AEGTVVLNAGSVAFTEESNSAADGTYNGSTSDPFRYTNTTGSQSATAKVDLDRTQAGENTLDGTYRSEILLGRDTSNDTIIGNDGNDILIGLGGNDTLSG
GNGDDILAGGAGNDTLDGGAGNDTATYIDATAGVTVSLNTSSAQNTVGAGTDTLTNIENLTGSKFNDTLTGNNSANTIKGLEGNDTISGLGGNDTLDGGS
GDDVIIGGLGDDILTGGSGADQFVLVKGQGGASAGAAPIDTITDFEVNIDKIVINGNNITGVSVSTPVSNTYTITVNYSDAATKHFKVTLSNSALLNDSG
KTHLNGVVISGGTATIDGTIVGAVLYMDLNANNQEDEGERLGITDQYGHVEWVVDISKFDVNGDGQYVIGEARAVQTGGFDIDTGLSYEINLYGPVGSAV
ISPLTSLLQAQLEAGMDYETANAALVAHLGLPEGTQIISFNPITGSGEVLAQNAGVMTAAIQFAEIAAIHYSTDEGHVSFAVFEAISKALLELPEGVVAD
FGDEGFLQSIANYLGLDALVTSDMIDYMATSQKALEASILTLAPGEDALTAISKIQHVTQGIYAQAIEMALIGYLPLDALKNMSAVLKAYADGDITDEQL
NSFEDKLSAVIVNWEDNNVTDTSHQATLQTERTVDDSSVKSEVNHEMAQEVNAVINDFMDEHNISLYYYDQQAQMPQEAQEQQPTEDQAEETVMHDDVVQ
DLLLDDHSLTMLANNPQLNGGNGNDVLHGTTGNDFIRGGQGNDTMTGGGGVDTFFWLSGDDDGGVDTITDFKANPVDQSSDASVLNLSDLLSDADLETNS
LDNYLNVSTTEEGDTAIKVDPNGNGNFDAPAQTIILEDVDLTAVFATNNSHDIVNQMIANGNLIVEQ
```

Leg-2 STX70670.1

```
MLAESVIGIVRAVNGLLEKVNVQGQASLVKSGARLEEGDVLTLLSGEAYIQFINGFPEALALEKPVKLDGVSPTLQYGAEDLKEQMVQEAIAKGIDPSVI
LDVLGSAAAGAEAVGSGGDAFIMDPLFGFGHVTAGYPTGPISFAYEADTQQLFWFVPEETGAIAESELTQEPESIPQIPQFTTNQAVLTVFEDALPSGIA
DSAGQARIASSSLSSLLTSSADVAASFAFNSNLSALPTLKSGGIDLSYELSADKRTLTLRESSTQGPGAEVMKLELTADGQLTQTLMNSIDHPAADSDG
EWMRFDLSPLIDVTFTRTSNGTVLESRTLPANAVVAGIQDDVPIARAQLTNNEILLDETIGMKVGDADAANDDFNPTTTVDPFNNIYGTPLGLVQNANLL
DASTSEMGGDYKNATMTHLLKITDAVSGLQTTDGTPINLFLESNGDITGRAGDVGAPAVFAIRMNPNTGAISVAQYGPIKQFDTNSHDEAVDLTGRISAV
VTAKDSDGDESNAEIPIGQLIIFADDGP
```

L2 repeats

```
SVAVNVGNEAGILLTTQDADTIGTATDAAVTSANFGNVFSNTPNYGADGAGSTVMSYSLGLIAASGADSGLDSNGSNIYLYDISGTIVGSTSATQAGVNA
SVAVNVGNEAGILLTTQDADTIGTATDAAVTSANFGNVFSNTPNYGADGAGSTVMSYSLGLIAASGADSGLDSNGSNIYLYDISGTIVGSTSATQAGVNA
SVAVNVGNEAGILLTTQDADTIGTATDAAVTSANFGNVFSNTPNYGADGAGSTVMSYSLGLIAASGADSGLDSNGSNIYLYDISGTIVGSTSATQAGVNA
SVAVNVGNEAGILLTTQDADTIGTATDAAVTSANFGNVFSNTPNYGADGAGSTVMSYSLGLIAASGADSGLDSNGSNIYLYDISGTIVGSTSATQAGVNA
SVAVNVGNEAGILLTTQDADTIGTATDAAVTSANFGNVFSNTPNYGADGAGSTVMSYSLGLIAASGADSGLDSNGSNIYLYDISGTIVGSTSATQAGVNA
SVAVNVGNEAGILLTTQDADTIGTATDAAVTSANFGNVFSNTPNYGADGAGSTVMSYSLGLIAASGADSGLDSNGSNIYLYDISGTIVGSTSATQAGVNA
SVAVNVGNEAGILLTTQDADTIGTATDAAVTSANFGNVFSNTPNYGADGAGSTVMSYSLGLIAASGADSGLDSNGSNIYLYDISGTIVGSTSATQAGVNA
SVAVNVGNEAGILLTTQDADTIGTATDAAVTSANFGNVFSNTPNYGADGAGSTVMSYSLGLIAASGADSGLDSNGSNIYLYDISGTIVGSTSATQAGVNA
SVAVNVGNEAGILLTTQDADTIGTATDAAVTSANFGNVFSNTPNYGADGAGSTVMSYSLGLIAASGADSGLDSNGSNIYLYDISGTIVGSTSATQAGVNA
SVAVNVGNEAGILLTTQDADTIGTATDAAVTSANFGNVFSNTPNYGADGAGSTVMSYSLGLIAASGADSGLDSNGSNIYLYDISGTIVGSTSATQAGVNA

GNTVFDLSVDGAGVVTLRQYAEIDHPIADDPSASGAPFDDQLAVLGNNLVSLTATALTTDGDGDTATASERIDLGGNVRFADDGP
GNTVFDLSVDGAGVVTLRQHAEIDHPIADDPSASGAPFDDQLAVLGNNLVSLTATALTTDGDGDTATASERIDLGGNVRFADDGP
GNTVFDLSVDGAGVVTLRQYAEIDHPIADDPSASGAPFDDQLAVLGNNLVSLTATALTTDGDGDTATASERIDLGGNVRFADDGP
GNTVFDLSVDGAGVVTLRQYAEIDHPIADDPSASGAPFDDQLAVLGNNLVSLTATALTTDGDGDTATASERIDLGGNVRFADDGP
GNTVFDLSVDGAGVVTLRQHAEIDHPIADDPSASGAPFDDQLAVLGNNLVSLTATALTTDGDGDTATASERIDLGGNVRFADDGP
GNTVFDLSVDGAGVVTLRQYAEIDHPIADDPSASGAPFDDQLAVLGNNLVSLTATALTTDGDGDTATASERIDLGGNVRFADDGP
GNTVFDLSVDGAGVVTLRQYAEIDHPIADDPSASGAPFDDQLAVLGNNLVSLTATALTTDGDGDTATASERIDLGGNVRFADDGP
GNTVFDLSVDGAGVVTLRQHAEIDHPIADDPSASGAPFDDQLAVLGNNLVSLTATALTTDGDGDTATASERIDLGGNVRFADDGP
GNTVFDLSVDGAGVVTLRQYAEIDHPIADDPSASGAPFDDQLAVLGNNLVSLTATALTTDGDGDTATASERIDLGGNVRFADDGP
GNTVFDLSVDGAGVVTLRQYAEIDHPIADDPSASGAPFDDQLAVLGNNLVSLTATALTTDGDGDTATASERIDLGGNVRFADDGP
```

L1 repeats

```
SVTMTVSDNNVITLNTQDADTIGAASDSDSVSFAAAFAVTPNYGADGAGTTVTTYALSVSAQGVDSGLDNNGNNIYLYNIAGSVVGSTSATQAGITTGNT
SVTMTVSDNNVITLNTQDADTIGAASDSDSVSFAAAFAVTPNYGADGAGTTVTTYALSVSAQGVDSGLDNNGNNIYLYNIAGSVVGSTSATQAGITTGNT
SVTMTVSDNNVITLNTQDADTIGAASDSDSVSFAAAFAVTPNYGADGAGTTVTTYALSVSAQGVDSGLDNNGNNIYLYNIAGSVVGSTSATQAGITTGNT
SVTMTVSDTNAITLNTQDADTIGAASDSDSVSFAAAFAVTPNYGADGAGTTVTTYALSVSAQGVDSGLDNNGNNIYLYNIAGSVVGSTSATQAGITTGNT
SVTMTVSDTNAITLNTQDADTIGAASDSDSVSFAAAFAVTPNYGADGAGTTVTTYALSVSAQGVDSGLDNNGNNIYLYNIAGSVVGSTSATQAGITTGNT
SVTMTVSDTNAITLNTQDADTIGAASDSDSVSFAAAFAVTPNYGADGAGTTVTTYALSVSAQGVDSGLDNNGNNIYLYNIAGSVVGSTSATQAGITTGNT
SVTMTVSDTNAITLNTQDADTIGAASDSDSVSFAAAFAVTPNYGADGAGTTVTTYALSVSAQGVDSGLDNNGNNIYLYNIAGSVVGSTSATQAGITTGNT
SVTMTVSDTNAITLNTQDADTIGAASDSDSVSFAAAFAVTPNYGADGAGTTVTTYALSVSVQGVDSGLDNNGNNIYLYNIAGSVVGSTSATQAGITTGNT
SVTMTVSDTNAITLNTQDADTIGAASDSDSVSFAAAFAVTPNYGADGAGTTVTTYALSVSAQGVDSGLDNNGNNIYLYNIAGSVVGSTSATQAGITTGNT
SVTMTVSDTNAITLNTQDADTIGAASDSDSVSFAAAFAVTPNYGADGAGTTVTTYALSVSAQGVDSGLDNNGNNIYLYNIAGSVVGSTSATQAGITTGNT
SVTMTVSDTNAITLNTQDADTIGAASDSDSVSFAAAFAVTPNYGADGAGTTVTTYALSVSAQGVDSGLDNNGNNIYLYNIAGSVVGSTSATQAGITTGNT
SVTMTVSDNNVITLNTQDADTIGAASDSDSVSFAAAFAVTPNYGADGAGTTVTTYALSVSAQGVDSGLDNNGNNIYLYNIAGSVVGSTSATQAGITTGNT
SVTMTVSDTNAITLNTQDADTIGAASDSDSVSFAAAFAVTPNYGADGAGTTVTTYALSVSAQGVDSGLDNNGNNIYLYNIAGSVVGSTSATQAGITTGNT
SVTMTVSDTNAITLNTQDADTIGAASDSDSVSFAAAFAVTPNYGADGAGTTVTTYALSVSAQGVDSGLDNNGNNIYLYNIAGSVVGSTSATQAGITTGNT
SVTMTVSDTNAITLNTQDADTIGAASDSDSVSFAAAFAVTPNYGADGAGTTVTTYALSVSAQGVDSGLDNNGNNIYLYNIAGSVVGSTSATQAGITTGNT
SVTMTVSDNNVITLNTQDADTIGAASDSDSVSFAAAFAVTPNYGADGAGTTVTTYALSVSAQGVDSGLDNNGNNIYLYNIAGSVVGSTSATQAGITTGNT

IFSLGVNSGSGVVTLTQHQEVDHGLPGASSNYAAQEAILNTGLVFLNATAVTTDGDGDTATASASLDLGGNVKFDDDGP
IFSLGVNSGSGVVTLTQHQEVDHGLPGASSNYAAQEAILNTGLVFLNATAVTTDGDGDTATASASLDLGGNVKFDDDGP
IFSLGVNSGSGVVTLTQHQEVDHGLPGASSNYAAQEAILNTGLVFLNATAVTTDGDGDTATASASLDLGGNVKFDDDGP
IFSLGVNSSSGVVTLTQHQEVDHGLPGASSNYAAQEAILNTGLVFLNATAVTTDGDGDTATASASLDLGGNVKFDDDGP
IFSLGVNSGSGVVTLTQHQEVDHGLPGASSNYAAQEAILNTGLVFLNATAVTTDGDGDTATASASLDLGGNVKFDDDGP
IFSLGVNSSSGVVTLTQHQEVDHGLPGASSNYAAQEAILNTGLVFLNATAVTTDRDGDTATASASLDLGGNVKFDDDGP
IFSLGVNSSSGVVTLTQHQEVDHGLPGASSNYAAQEAILNTGLVFLNATAVTTDGDGDTATASASLDLGGNVKFDDDGP
IFSLGVNSSSGVVTLTQHQEVDHGLPGASSNYAAQEAILNTGLVFLNATAVTTDGDGDTATASASLDLGGNVKFDDDGP
IFSLGVNSSSGVVTLTQHQEVDHGLPGASSNYAAQEAILNTGLVFLNATAVTTDRDGDTATASASLDLGGNVKFDDDGP
IFSLGVNSSSGVVTLTQHQEVDHGLPGASSNYAAQEAILNTGLVFLNATAVTTDGDGDTATASASLDLGGNVKFDDDGP
IFSLGVNSGSGVVTLTQHQEVDHGLPGASSNYAAQEAILNTGLVFLNATAVTTDRDGDTATASASLDLGGNVKFDDDGP
IFSLGVNSSSGVVTLTQHQEVDHGLPGASSNYAAQEAILNTGLVFLNATAVTTDGDGDTATASASLDLGGNVKFDDDGP
IFSLGVNSSSGVVTLTQHQEVDHGLPGASSNYAAQEAILNTGLVFLNATAVTTDRDGDTATASASLDLGGNVKFDDDGP
IFSLGVNSGSGVVTLTQHQEVDHGLPGASSNYAAQEAILNTGLVFLNATAVTTDGDGDTATASASLDLGGNVKFDDDGP
IFSLGVNSSSGVVTLTQHQEVDHGLPGASSNYAAQEAILNTGLVFLNAMAVTTDGDGDTATASASLDLGGNVKFDDDGP
IFSLGVNSGSGVVTLTQHQEVDHGLPGASSNYAAQEAILNTGLVFLNATAVTTDRDGDTATASASLDLGGNVKFDDDGP

VASNITRSGLANEGADTNLMLILDTSGSMAGSGIQTLINSTLELLERYEALGNVKVRIVTFNTSATAIGSVWMTVDAAKNALLGLTAGGNTNFDAALITA
MNAFNSGTVDGADGRIGGAQNVSYFISDGNPTVNQDWPSIPGTLAQNGIQPSEQAAWEAFLQDTNGASAGGEKIRSYALGMGTGITVSNLEPIGFNGVNG
TDIPAVVVTDISQLDSVLASTITASPISGTLISDPNPGVNFGADGGHVQSVTVNGITYTVNANLSSLSTSGSATQGSNTHSYDNSTHVLTVTISTGESLT
IDMDNGSYTFTPPANITSSINRIINYVLVDGDGDTAAATITFNIDISGFNDPLVVRDDLVLTNQPAVSGQDVISIPTWALLANDTGGSGVSSVSSVTQPV
AEGTVVLNAGSVAFTEESNSAADGTYNGSTSDPFRYTNTTGSQSATAKVDLDRTQAGENTLDGTYRSEILLGRDTSNDTIIGNDGNDILIGLGGNDTLSG
GNGDDILAGGAGNDTLDGGAGNDTATYIDATAGVTVSLNTSSAQNTVGAGTDTLTNIENLTGSKFNDTLTGNNSANTIKGLEGNDTISGLGGNDTLDGGS
GDDVIIGGLGDDILTGGSGADQFVLVKGQGGASAGAAPIDTITDFEVNIDKIVINGNNITGVSVSTPVSNTYTITVNYSDAATEHFKVTLSNSALLNDSG
KTHLNGVVISGGTATIDGTIVGAVLYMDLNANNQEDEGERLGITDQYGHVEWVVDISKFDVNGDGQYVIGEARAVQTGGFDIDTGLSYEINLYGPVGSAV
```

ISPLTSLLQAQLEAGMDYETANAALVARLGLPEGTQIISFNPITGSGEVLAQNAGVMTAAIQFAEIAAIHYSTDEGHVSFAVFEAISKALLELPEGVVAD
FGDEGFLQSISNYLGLDALVTSDMIDYMATSQKALEASILTLAPGEDALTAISKIQHVTQGIYAQAIEMALIGYLPLDALKNMSAVLKAYADGDITDEQL
NSFEDKLSAVIVNWEDNNVTDTSHQATLQTERTVDDSSVKSEVNHEMAQEVNAVINDFMDEHNISLYYYDQQAQMPQEAQEQQPTEHQAEETVMHDDVVQ
DLLLDDHSLTMLANNPQLNGGNGNDVLHGTTGNDFIRGGQGNDTMTGGGGVDTFFWLSGDDDGGVDTITDFKANPVDQSSDASVLNLSDLLSDADLETNS
LDNYLNVSTTEEGDTAIKVDPNGNGNFDAPAQTIILEDVDLTAVFATNNSHDIVNQMIANGNLIVEQ

Leg-3 ABQ56563.1
MLAESVIGIVRAVNGLLEKVNVQGQASLVKSGARLEEGDVLTLLSGEAYIQFINGFPEALALEKPVKLDGVSPTLQYGAEDLKEQMVQEAIAKGIDPSVI
LDVLGSAAAGAEAVGSGGDAFIMDPLFGFGHVTAGYPTGPISFAYEADTQQLFWFVPEETGAIAESELTQEPESIPQIPQFTTNQAVLTVFEDALPSGIP
DSAGQARIASSSLSTLLTSSPDVVASFAFNTNLSVLPTLKSGGIDLDYNLSSDKRTLTASIPGGAAVMQFELTADGQLTQTLMDSIDHPTADSDDSEWMR
LDLSPLIDVTFTRTSDGSVLESRTLPANAVVAGIQDDVPIARAQLTNNEILLDETIGMKAGDADAANDDFNPTTTVDPFNNTYGIPIGLVQNANLLDTST
SEMGGDYKNATMTHLLKITDAVSGLQTTDGTPINLFLESNGDISGRAGDIGAPAVFAIRMNPNTGAITVAQYGSIKQFDTNSHDEAVDLTGRISAVVRVT
DGDGDVSNAEIPIGQRIVFEDDGP

L3 repeats
IAQIAATGVKVTHDETAGVDAGSDDVASPLALFAGVSQKSTDMAGFAQSSGAVVSSAGSLVGQDNEGATIKFSLAIANAASGLQTTDGDAITLTLESGFV
VAQIAATGVKVTHDETAGVNAGSDDVASPLALFAGVSQKSTDMAGFAQSSGAVVSSAGSLVGQDNEGATIKFSLAIANAASGLQTTDGDAITLTLESGFV
VAQIAATGVKVTHDETAGVDAGSDDVASPLALFAGVSQKSTDMAGFAQSSGAVVSSAGSLVGQDNEGATIKFSLAIANAASGLQTTDGDAITLTLESGFV
VAQIAATGVKVTHDETAGVDAGSDDVASPLALFAGVSQKSTDMAGFAQSSGAVVSSAGSLVGQDNEGATIKFSLAIANAASGLQTTDGDAITLTLESGFV
VAQIAATGVKVTHDETAGVDAGSDDVASPLSVFVGVVNKSTDMAGFAQSSGAVVSSAGSVTGQDNEGATIKFSLAIANAASGLQTTDGDAITLTLESGLV
VAQIAATGAKVIHDETTGLDAGSNDVAGPLSVFAGVVNKSTDMAGFAQSSGAVVSSAGSVTGQDNEGATIKFSLAIANAASGLQTTDGDAITLTLESGLV
VAQIAATGTKVIHDETTGLDAGSNDVAGPLSVFAGVVNKSTDMAGFAQSSGAVVSSTGSVTGQDNEGATIKFSLAIANAASGLQTTDGDAITLTLESGLV
VAQIAATGAKVIHDETTGLDAGSNDVASPLSVFVGVVNKSTDMAGFAQSSGAVVSSAGSVTGQDNEGATIKFSLAIANAASGLQTTDGDAITLTLESGLV
VAQIAATGAKVIHDETTGLDAGSNDVAGPLSVFAGVVNKSTDMAGFAQSSGAVVSSAGSVTGQDNEGATIKFSLAIANAASGLQTTDGDAITLTLESGLV
VAQIAATGAKVIHDETTGLDAGSNDVAGPLSVFAGVVNKSTDMAGFAQSSGAVVSSAGSVTGQDNEGATIKFSLAIANAASGLQTTDGDAITLTLESGLV
VAQIAATGTKVIHDETTGLDAGSNDVAGPLSVFAGVVNKSTDMAGFAQSSGAVVSSTGSVTGQDNEGATIKFSLAIANAASGLQTTDGDAITLTLESGLV
VAQIAATGAKVIHDETTGLDAGSNDVASPLSVFVGVVNKSTDMAGFAQSSGAVVSSAGSVTGQDNEGATIKFSLAIANAASGLQTTDGDAITLTLESGLV
VAQIAATGAKVIHDETTGLDAGSNDVAGPLSVFAGVVNKSTDMAGFAQSSGAVVSSAGSVTGQDNEGATIKFSLAIANAASGLQTTDGDAITLTLESGLV
VAQIAATGAKVIHDETTGLDAGSNDVAGPLSVFAGVVNKSTDMAGFAQSSGAVVSSTGSVTGQDNEGATIKFSLAIANAASGLQTTDGDAITLTLESGLV
VAQIAATGAKVIHDETTGLDAGSNDVAGPLSVFAGVVNKSTDMAGFAQSSGAVVSSAGSVTGQDNEGATIKFSLAIANAASGLQTTDGDAITLTLESGLV
VAQIAATGAKVIHDETTGLDAGSNDVAGPLSVFAGVVNKSTDMAGFAQSSGAVVSSAGSVTGQDNEGATIKFSLAIANAASGLQTTDGDAITLTLESGLV
VAQIAATGTKVIHDETTGLDAGSNDVAGPLSVFAGVVNKSTDMAGFAQSSGAVVSSTGSVTGQDNEGATIKFSLAIANAASGLQTTDGDAITLTLESGLV
VAQIAATGTKVIHDETTGLDAGSNDVAGPLSVFAGVVNKSTDMAGFAQSSGAVVSSTGSVTGQDNEGATIKFSLAIANAASGLQTTDGDAITLTLESGLV
VAQIAATGAKVIHDETTGLDAGSNDVASPLSVFVGVVNKSTDMAGFAQSSGAVVSSAGSVTGQDNEGATIKFSLAIANAASGLQTTDGDAITLTLESGLV
VAQIAATGAKVIHDETTGLDAGSNDVAGPLSVFAGVVNKSTDMAGFAQSSGAVVSSAGSVTGQDNEGATIKFSLAIANAASGLQTTDGDAITLTLESGLV
VAQIAATGAKVIHDETTGLDAGSNDVAGPLSVFAGVVNKSTDMAGFAQSSGAVVSSTGSVTGQDNEGATIKFSLAIANAASGLQTTDGDAITLTLESGLV
VAQIAATGAKVIHDETTGLDAGSNDVAGPLSVFAGVVNKSTDMAGFAQSSGAVVSSAGSVTGQDNEGATIKFSLAIANAASGLQTTDGDAITLTLESGLV
VAQIAATGAKVIHDETTGLDAGSNDVAGPLSVFAGVVNKSTDMAGFAQSSGAVVSSTGSVTGQDNEGATIKFSLAIANAASGLQTTDGDAITLTLESGLV
VAQIAATGAKVIHDETTGLDAGSNDVAGPLSVFAGVVNKSTDMAGFAQSSGAVVSSTGSVTGQDNEGATIKFSLAIANAASGLQTTDGDAITLTLESGLV
VAQIAATGAKVIHDETTGLDAGSNDVAGPLSVFAGVVNKSTDMAGFAQSSGAVVSSAGSVTGQDNEGATIKFSLAIANAASGLQTTDGDAITLTLESGLV

VGRDAGGDAVFAIAIDADTGVLSLAQYESIKHPSGGASYDEAVDLSGKINAVVTVTDGDGDVATQAIGIGDAIVFEDDGP
VGRDAGGDAVFAIAIDADTGVLSLAQYESIKHPSGGASYDEAVDLSGKINAVVTVTDGDGDVATQAIGIGDAIVFEDDGP
VGRDAGGDAVFAIAIDADTGVLSLAQYESIKHPSGGASYDEAVDLSGKINAVVTVTDGDGDVATQAIGIGDAIVFEDDGP
VGRDAGGDAVFAIAIDADTGVLSLAQYESIKHPSGGASYDEAVDLSGKINAVVTVTDGDGDVATQAIGIGDAIVFEDDGP
VGRDASGKAVLAISIDADTGVLSVAQYESIKHPTGGSSYDEAVHLGGKINAVVTVTDGDGDVATQAIGIGDKVVFEDDGP
VGRDASGKAVLAISIDADTGVLSVAQYESIKHPTGGASYDEAVNLSGKINAVVTVTDGDGDVATQAIGIGDAIVFEDDGP
VGRDASGKAVLAISIDADTGVLSVAQYESIKHPTGGSSYDEAVHLGGKINAVVTVTDGDGDVATQAIGIGDKVVFEDDGP
VGRDASGKAVLAISIDADTGVLSVAQYESIKHPTGGSSYDEAVHLGGKINAVVTVTDGDGDVATQAIGIGDKVVFEDDGP
VGRDASGKAVLAISIDADTGVLSVAQYESIKHPTGGSSYDEAVHLGGKINAVVTVTDGDGDVATQAIGIGDKVVFEDDGP
VGRDASGKAVLAISIDADTGVLSVAQYESIKHPTGGASYDEAVNLSGKINAVVTVTDGDGDVATQAIGIGDAIVFEDDGP
VGRDASGKAVLAISIDADTGVLSVAQYESIKHPTGGSSYDEAVHLGGKINAVVTVTDGDGDVATQAIGIGDKVVFEDDGP
VGRDASGKAVLAISIDADTGVLSVAQYESIKHPTGGSSYDEAVHLGGKINAVVTVTDGDGDVATQAIGIGDAIVFEDDGP
VGRDASGKAVLAISIDADTGVLSVAQYESIKHPTGGASYDEAVNLSGKINAVVTVTDGDGDVATQAIGIGDAIVFEDDGP
VGRDASGKAVLAISIDADTGVLSVAQYESIKHPTGGSSYDEAVHLGGKINAVVTVTDGDGDVATQAIGIGDKVVFEDDGP
VGRDASGKAVLAISIDADTGVLSVAQYESIKHPTGGSSYDEAVHLGGKINAVVTVTDGDGDVATQAIGIGDKVVFEDDGP
VGRDASGKAVLAISIDADTGVLSVAQYESIKHPTGGSSYDEAVHLGGKINAVVTVTDGDGDVATQAIGIGDAIVFEDDGP
VGRDASGKAVLAISIDADTGVLSVAQYESIKHPTGGASYDEAVNLSGKINAVVTVTDGDGDVATQAIGIGDAIVFEDDGP
VGRDASGKAVLAISIDADTGVLSVAQYESIKHPTGGSSYDEAVHLGGKINAVVTVTDGDGDVATQAIGIGDKVVFEDDGP
VGRDASGKAVLAISIDADTGVLSVAQYESIKHPTGGSSYDEAVHLGGKINAVVTVTDGDGDVATQAIGIGDAIVFEDDGP
V-RDASGKAVLAISIDADTGVLSVAQYESIKHPTGGASYDEAVNLSGKINAVVTVTDGDGDVATQAIGIGDAIVFEDDGP
VGRDASGKAVLAISIDADTGVLSVAQYESIKHPTGGSSYDEAVHLGGKINAVVTVTDGDGDVATQAIGIGDAIVFEDDGP
VGRDASGKAVLAISIDADTGVLSVAQYESIKHPTGGASYDEAVNLSGKINAVVTVTDGDGDVATQAIGIGDAIVFEDDGP
VGRDASGKAVLAISIDADTGVLSVAQYESIKHPTGGSSYDEAVHLGGKINAVVTVTDGDGDVATQAIGIGDKVVFEDDGP
VGRDASGKAVLAISIDADTGVLSVAQYESIKHPTGGSSYDEAVHLGGKINAVVTVTDGDGDVATQAIGIGDKVVFEDDGP
VGRDASGKAVLAISIDADTGVLSVAQYESIKHPTGGSSYDEAVHLGGKINAVVTVTDGDGDVATQAIGIGDKVVFEDDGP

VASNITRSGLANEGADTNLMLILDTSGSMAGSGIQTLINSTLELLERYEALGNVKVRIVTFNTSATAIGSVWMTVDAAKNALLGLTAGGNTNFDAALITA
MNAFNSGTVGGADGRIGGAQNVSYFISDGNPTVNQDWPSIPGTLAQNGIQPSEQAAWEAFLQDTNGASAGGEKIRSYALGMGTGITVSNLEPIGFNGVNG
TDIPAVVVTDISQLDSVLASTITASPISGTLISDPNPGVNFGADGGHVQSVTVNGITYTVNANLSSLSTSGSATQGSNTHSYDNSTHVLTVTISTGESLT
IDMDNGSYTFTPPANITSSINRIINYVLVDGDGDTAAATITFNIDISGFNDPLVVRDDLVLTNQPAVSGQDVISIPTWALLANDTGGSGVSSVSSVTQPV
AEGTVVLNAGSVAFTEESNSAADGTYNGSTSDPFRYTNTTGSQSATAKVDLDRTQAGENRLDGTYRSEILLGRDTSNDTIIGNDGNDILIGLGGNDTLSG
GNGDDILAGGAGNDTLDGGAGNDTATYIDATAGVTVSLNTSSAQNTVGAGTDTLTNIENLTGSKFNDTLTGNNSANTIKGLEGNDTISGLGGNDTLDGGS
GDDVIIGGLGDDILTGGSGADQFVLVKGQGGASAGAAPIDTITDFEVNIDKIVINGNNITGVSVSTPVSNTYTITVNYSDAATEHFKVTLSNGAVLNDSG
KTHLNGVVISGGTATIDGTIVGAVLYLDLNANNQEDEGERLGITNQYGHVEWVVDLSKFDVNGDGQYVIGEARAVQTGGFDIDTGLSYEINLYGPVGSAV
ISPLTSLLQAQLEAGMDYETANAALVARLGLPEGTQIISFNPITGSGEVLAQNAGVMTAAIQFAEIAAIHYSTDEGHVSFAVFEAISKALLELPEGVVAD
FGDEGFLQSISNYLGLDALVTSDMIDYMATSQKALEASILTLAPGEDALTAISKIQHVTQGIYAQAIEMALIGYLPLDALKNMSAVLKAYADGDITDEQL
NSFEDKLSAVIVNWEDNNVTDTSHQATLQTERTVDDSSVKSEVNHEMAQEVNAVINDFMDEHNISLYYYDQQAQMPQEAQQPTEDQAEETVMHDDVVQDL
LLDDHSLTMLANNPELNGGNGNDILHGTTGNDFIRGGQGNDTMTGGGGVDTFFWLSGDDDGGVDTITDFKANPVDKSSDASVLNLSDLLSDADLETNSLD
NYLNVSTTEEGDTAIKVDPNGNGNFDAPAQTIILEDVDLTAVFATNNSHDIVNQMIANGNLIVEQ

Leg-4 STY04251.1
MLAESVIGIVRAVNGLLEKVNVQGQASLVKSGARLEEGDVLTLLSGEAYIQFINGFPEALALEKPVKLDGVSPTLQYGAEDLKEQMVQEAIAKGIDPSVI
LDVLGSAAAGAEAVGSGGDAFIMDPLFGFGHVTAGYPTGPISFAYEADTQQLFWFVPEETGAIAESELTQEPESIPQIPQFTTNQAVLTVFEDALPSGIA
DSAGQARIASSSLSSLLTSSADVAASFAFNSNLSALPTLKSGGIDLSYELSADKRTLTLRESSTQGPGAEVMKLELTADGQLTQTLMNSIDHPAADSDDG
EWMRFDLSPLIDVTFTRTSNGTVLESRTLPANAVVAGIQDDVPIARAQLTNNEILLDETIGMKVGDADAANDDFNPTTTVDPFNNIYGTPLGLVQNANLL
DASTSEMGGDYKNATMTHLLKITDAVSGLQTTDGTPINLFLESNGDITGRAGDVGAPAVFAIRMNPNTGAISVAQYGPIKQFDTNSHDEAVDLTGRISAV
VTAKDSDGDESNAEIPIGQLIIFADDGP

L2 repeats
SVAVNVGNEAGILLTTQDADTIGTATDAAVTSANFGNVFSNTPNYGADGAGSTVMSYSLGLIAASGADSGLDSNGSNIYLYDISGTIVGSTSATQAGVNA
SVAVNVGNEAGILLTTQDADTIGTATDAAVTSANFGNVFSNTPNYGADGAGSTVMSYSLGLIAASGADSGLDSNGSNIYLYDISGTIVGSTSATQAGVNA
SVAVNVGNEAGILLTTQDADTIGTATDAAVTSANFGNVFSNTPNYGADGAGSTVMSYSLGLIAASGADSGLDSNGSNIYLYDISGTIVGSTSATQAGVNA
SVAVNVGNEAGILLTTQDADTIGTATDAAVTSANFGNVFSNTPNYGADGAGSTVMSYSLGLIAASGADSGLDSNGSNIYLYDISGTIVGSTSATQAGVNA
SVAVNVGNEAGILLTTQDADTIGTATDAAVTSANFGNVFSNTPNYGADGAGSTVMSYSLGLIAASGADSGLDSNGSNIYLYDISGTIVGSTSATQAGVNA
SVAVNVGNEAGILLTTQDADTIGTATDAAVTSANFGNVFSNTPNYGADGAGSTVMSYSLGLIAASGADSGLDSNGSNIYLYDISGTIVGSTSATQAGVNA
SVAVNVGNEAGILLTTQDADTIGTATDAAVTSANFGNVFSNTPNYGADGAGSTVMSYSLGLIAASGADSGLDSNGSNIYLYDISGTIVGSTSATQAGVNA
SVAVNVGNEAGILLTTQDADTIGTATDAAVTSANFGNVFSNTPNYGADGAGSTVMSYSLGLIAASGADSGLDSNGSNIYLYDISGTIVGSTSATQAGVNA
SVAVNVGNEAGILLTTQDADTIGTATDAAVTSANFGNVFSNTPNYGADGAGSTVMSYSLGLIAASGADSGLDSNGSNIYLYDISGTIVGSTSATQAGVNA
SVAVNVGNEAGILLTTQDADTIGTATDAAVTSANFGNVFSNTPNYGADGAGSTVMSYSLGLIAASGADSGLDSNGSNIYLYDISGTIVGSTSATQAGVNA

GNTVFDLSVDGAGVVTLRQYAEIDHPIADDPSASGAPFDDQLAVLGNNLVSLTATALTTDGDGDTATASERIDLGGNVRFADDGP
GNTVFDLSVDGAGVVTLRQHAEIDHPIADDPSASGAPFDDQLAVLGNNLVSLTATALTTDGDGDTATASERIDLGGNVRFADDGP
GNTVFDLSVDGAGVVTLRQYAEIDHPIADDPSASGAPFDDQLAVLGNNLVSLTATALTTDGDGDTATASERIDLGGNVRFADDGP
GNTVFDLSVDGAGVVTLRQYAEIDHPIADDPSASGAPFDDQLAVLGNNLVSLTATALTTDGDGDTATASERIDLGGNVRFADDGP
GNTVFDLSVDGAGVVTLRQHAEIDHPIADDPSASGAPFDDQLAVLGNNLVSLTATALTTDGDGDTATASERIDLGGNVRFADDGP
GNTVFDLSVDGAGVVTLRQYAEIDHPIADDPSASGAPFDDQLAVLGNNLVSLTATALTTDGDGDTATASERIDLGGNVRFADDGP
GNTVFDLSVDGAGVVTLRQYAEIDHPIADDPSASGAPFDDQLAVLGNNLVSLTATALTTDGDGDTATASERIDLGGNVRFADDGP
GNTVFDLSVDGAGVVTLRQHAEIDHPIADDPSASGAPFDDQLAVLGNNLVSLTATALTTDGDGDTATASERIDLGGNVRFADDGP
GNTVFDLSVDGAGVVTLRQYAEIDHPIADDPSASGAPFDDQLAVLGNNLVSLTATALTTDGDGDTATASERIDLGGNVRFADDGP
GNTVFDLSVDGAGVVTLRQYAEIDHPIADDPSASGAPFDDQLAVLGNNLVSLTATALTTDGDGDTATASERIDLGGNVRFADDGP

L1 repeats
SVTMTVSDNNAITLNTQDADTIGAASDSDSVSFAAAFAVTPNYGADGAGTTVTTYALSVSAQGVDSGLDNNGNNIYLYNIAGSVVGSTSATQAGITTGNT
SVTMTVSDNNAITLNTQDADTIGAASDSDSVSFAAAFAVTPNYGADGAGTTVTTYALSVSAQGVDSGLDNNGNNIYLYNIAGSVVGSTSATQAGITTGNT
SVTMTVSDNNAITLNTQDADTIGAASDSDSVSFAAAFAVTPNYGADGAGTTVTTYALSVSAQGVDSGLDNNGNNIYLYNIAGSVVGSTSATQAGITTGNT
SVTMTVSDNNAITLNTQDADTIGAASDSDSVSFAAAFAVTPNYGADGAGTTVTTYALSVSAQGVDSGLDNNGNNIYLYNIAGSVVGSTSATQAGITTGNT
SVTMTVSDNNAITLNTQDADTIGAASDSDSVSFAAAFAVTPNYGADGAGTTVTTYALSVSAQGVDSGLDNNGNNIYLYNIAGSVVGSTSATQAGITTGNT
SVTMTVSDNNAITLNTQDADTIGAASDSDSVSFAAAFAVTPNYGADGAGTTVTTYALSVSAQGVDSGLDNNGNNIYLYNIAGSVVGSTSATQAGITTGNT
SVTMTVSDNNAITLNTQDADTIGAASDSDSVSFAAAFAVTPNYGADGAGTTVTTYALSVSAQGVDSGLDNNGNNIYLYNIAGSVVGSTSATQAGITTGNT
SVTMTVSDNNAITLNTQDADTIGAASDSDSVSFAAAFAVTPNYGADGAGTTVTTYALSVSAQGVDSGLDNNGNNIYLYNIAGSVVGSTSATQAGITTGNT
SVTMTVSDNNAITLNTQDADTIGAASDSDSVSFAAAFAVTPNYGADGAGTTVTTYALSVSAQGVDSGLDNNGNNIYLYNIAGSVVGSTSATQAGITTGNT
SVTMTVSDNNAITLNTQDADTIGAASDSDSVSFAAAFAVTPNYGADGAGTTVTTYALSVSAQGVDSGLDNNGNNIYLYNIAGSVVGSTSATQAGITTGNT
SVTMTVSDNNAITLNTQDADTIGAASDSDSVSFAAAFAVTPNYGADGAGTTVTTYALSVSAQGVDSGLDNNGNNIYLYNIAGSVVGSTSATQAGITTGNT
SVTMTVSDNNAITLNTQDADTIGAASDSDSVSFAAAFAVTPNYGADGAGTTVTTYALSVSAQGVDSGLDNNGNNIYLYNIAGSVVGSTSATQAGITTGNT
SVTMTVSDNNAITLNTQDADTIGAASDSDSVSFAAAFAVTPNYGADGAGTTVTTYALSVSAQGVDSGLDNNGNNIYLYNIAGSVVGSTSATQAGITTGNT
SVTMTVSDNNAITLNTQDADTIGAASDSDSVSFAAAFAVTPNYGADGAGTTVTTYALSVSAQGVDSGLDNNGNNIYLYNIAGSVVGSTSATQAGITTGNT

IFSLGVNSSSGVVTLTQHQEVDHGLPGASSNYAAQEAILNTGLVFLNATAVTTDGDGDTATASASLDLGGNVKFDDDGP
IFSLGVNSSSGVVTLTQHQEVDHGLPGASSNYAAQEAILNTGLVFLNATAVTTDRDGDTATASASLDLGGNVKFDDDGP
IFSLGVNSSSGVVTLTQHQEVDHGLPGASSNYAAQEAILNTGLVFLNATAVTTDGDGDTATASASLDLGGNVKFDDDGP
IFSLGVNSSSGVVTLTQHQEVDHGLPGASSNYAAQEAILNMGLVFLNATAVTTDGDGDTATASASLDLGGNVKFDDDGP
IFSLGVNSSSGVVTLTQHQEVDHGLPGASSNYAAQEAILNTDLVFLNATAVTTDRDGDTATASASLDLGGNVKFDDDGP
IFSLGVNSSSGVVTLTQHQEVDHGLPGASSNYAAQEAILNMGLVFLNATAVTTDRDGDTATASASLDLGGNVKFDDDGP
IFSLGVNSSSGVVTLTQHQEVDHGLPGASSNYAAQEAILNTDLVFLNATAVTTDRDGDTATASASLDLGGNVKFDDDGP
IFSLGVNSSSGVVTLTQHQEVDHGLPGASSNYAAQEAILNTGLVFLNATAVTTDGDGDTATASASLDLGGNVKFDDDGP
IFSLGVNSSSGVVTLTQHQEVDHGLPGASSNYAAQEAILNTGLVFLNATAVTTDGDGDTATASASLDLGGNVKFDDDGP
IFSLGVNSSSGVVTLTQHQEVDHGLPGASSNYAAQEAILNTGLVFLNATAVTTDGDGDTATASASLDLGGNVKFDDDGP
IFSLGVNSSSGVVTLTQHQEVDHGLPGASSNYAAQEAILNTGLVFLNATAVTTDGDGDTATASASLDLGGNVKFDDDGP
IFSLGVNSSSGVVTLTQHQEVDHGLPGASSNYAAQEAILNTGLVFLNATAVTTDRDGDTATASASLDLGGNVKFDDDGP
IFSLGVNSSSGVVTLTQHQEVDHGLPGASSNYAAQEAILNTGLVFLNATAVTTDGDGDTATASASLDLGGNVKFDDDGP
IFSLGVNSSSGVVTLTQHQEVDHGLPGASSNYAAQEAILNTGLVFLNATAVTTDGDGDTATASASLDLGGNVKFDDDGP

L8 repeat
TVGTISLVADEDNLPRGNNDTASGDAAQSNLTGTLPVNFGADGAGSIDFQGMHGMSAVIGNDNITYNWNAGTNTLTAYRTGGTLGVNDVFKIVVNPTTGQ
YTFTLLAAINHHAVADNTEGLVDPFVNLNYRVIDGDGDTAIGTLKVTIDDDVP

KAITPEEGFVSNKAGVVKVFNLDLDSNIGNNVGADQPGSIRFINVVDGQQAQGIIDGELVNLTTVNGQLIKLYISNDGMIITGSTAATEGAINSSNTVFK
VTMLPDNDYNLANDKYSVEMFGKIGVINTVNFSELTGESPAGNVNFKIISDQDFPGYEVLITPKDRVNPDDTVGGTVNSSSTDISISNQWIDQGSGDSEI
LRLDYGQFAIVNNKFNIEKHFIVNGASFEIHQIQGSPSNRASINVKAFDANDNNQLNDDPLDTITRIVVFTSSGGIETVIADVTSDTSFGGMTFDFQGTN
GVNITGLDDGYRVRAYTTDGYNRLEIDNISSTPFSIGGLGIQAIIDTEKEVKMTYDVRLTDSDGDSVDSSLEINIGPSLFKVGENVADTSNSNVPHTVGE
DTGVIDGSSGADVLVGDIGGIDVSQTNYNLILILDTSGSMEEQIPFGDDGSIQRIQALKNSVNELIDNLAESNALNIRVHIVQFNTHASTVGTFNLIQNG
TLQSTQVNNAHNGVNSLTAGGGTNYEAAFIEALDWVNNNGPLTANDPLNQVLFLSDGVPTYYYAGNGTTTLGGSGSSASFDTIFQPLGDSSSGSWVDNQS
EVTQLEADFGQIQAVGINLNPNTIAYNPAGTANDLTAVQILDKMEGSDPSSLSNNITTAEQLTNVLAELSLLNKLNNVGSDVIAGNEENDIIFGDTLNTD
KLAVDSGLNLFNFPKGSGWAVFEELEANHGWSRQDTLDYIASHADELGKETVLPDGGTRSGGHDTIGGGQGNDQIYGQEGNDTIDGGAGNDVIVGGTGND
TLTGGAGADQFVLVKGQGGASAGAAPIDTITDFEVSIDKIVINGNNITGVSVSTPVSNTYTITVNYSDAATEHFKVTLSNGALLNDSGKTHLNGVVISGG
TATIDGTIVGAVLYLDLNANNQEDAGERLGITNQYGHVEWVVDLSKFDVNGDGQYVIGEARAVQTGGFDIDTGLSYEINLYGPVGSAVISPLTSLLQAQL

EAGMDYETANAALVARLGLPEGTQIISFNPITGSGEVLAQNAGVMTAAIQFAEIAAIHYSTDESHVSFAVFEAISKALLELPEGVVADFGDEGFLQSISN
YLGLDALVTSDMIDYMAAGQKALEASILTLAPGEDALTAISKIQHVTQGIYAQAIEMALIGYLPLDALKNMSAVLKAYADGDITDEQLNSFEDKLSAVVV
NWEDNNVTDTSHQASLQTDRTVDDASVKSEVNHEMAQEVNAVINDFMAEHNISLYYYAQQAQMPQEAQEQQPTEDQAEETVMHDDVVQDLLLDDHSLTML
ANNPQLNGGNGNDVLHGTTGNDFIRGGQGNDTMTGGGGVDTFFWLSGDDDGGVDTITDFKANPVDKSSDASVLNLSDLLSDADLETNSLDNYLNVSTTEE
GDTAIKVDPNGNGNFDAPAQTIILEDVDLTAVFATNNSHDIVNQMIANGNLIVEQ

Leg-5 CAH14915.1
MLAESVIGIVRAVNGLLEKVNAQGQASIVKSGARLQEGDVLTLLSGEAYIQFIHGFPEALALGKPVNLYGVSPILQYGVEELNEQLVQEAIAKGIDPSII
LDVLGSAAAGAEAVGSGGDAFIIDPLFGFGQVTAGYPTGPISFAYEADTQHLFWYVPEETGVIAESELASEPESIPQVPQFTSNQATITVFEDALPSGGI
RDSVEQTTTARASLSPLISSSSEVAASFAFNTNLSVLPKLQSGGLELEYILSSDHLALSATTPDGADVVRFELTADGQLTQILIGSIDHPRADSDDSEWH
RLDLSPVIDVTFTRISDGTFLGSRTLPASAAMAEIQDDVPIARVQLTNNEILLDETVGIKFGDADAANDDFNPTTTADPFNNTYGRPIGLVQNANLLDTS
TSEMGGDYKNATMTHLLKITDAVSGLQTSDGTPINLFLESNGDITGRAGDIGAPAVFAIRMNPNTGSITVAQYGSIKQFDTNSHDEAVDLTGRISAVVTV
KDSDGDVSNVEIPIGQQIIFEDDGP

L4 repeats
SIDGSKVLSADVLTVDETNLGANATASFADNFAQAIDFGEDGAGSVTYALVLNGSNVGSGLYAIDNLDVSTADGDGIGRGGEIVLNQNGNVVTGSLGGVD
SIDGSKVLSADVLTVDETNLGANATASFADNFAQAIDFGEDGAGSVTYALVLNGSNVGSGLYAIDNLDVSTADGDGIGRGGEIVLNQNGNVVTGSLGGVD
SIDGSKVLSADVLTVDETNLGANATASFADNFAQAIDFGEDGAGSVTYALVLNGSNVGSGLYAIDNLDVSTADGDGIGRGGEIVLNQNGNVVTGSLGGVD
SIDGSKVLSADVLTVDETNLGANATASFADNFAQAIDFGEDGAGSVTYALVLNGSNVGSGLYAIDNLDVSTADGDGIGRGGEIVLNQNGNVVTGSLGGVD
SIDGSKVLSADVLTVDETNLGANATASFADNFAQAIDFGEDGAGSVTYALVLNGSNVGSGLYAIDNLDVSTADGDGIGRGGEIVLNQNGNVVTGSLGGVD
SIDGSKVLSADVLTVDETNLGANATASFADNFAQAIDFGEDGAGSVTYALVLNGSNVGSGLYAIDNLDVSTADGDGIGRGGEIVLNQNGNVVTGSLGGVD
SIDGSKVLSADVLTVDETNLGANATASFADNFAQAIDFGEDGAGSVTYALVLNGSNVGSGLYAIDNLDVSTADGDGIGRGGEIVLNQNGNVVTGSLGGVD
SIDGSKVLSADVLTVDETNLGVNATASFADNFAQAIDFGEDGAGSVTYALVLNGSNVGSGLYAIDNLDVSTADGDGIGRGGEIVLNQNGNVVTGSLGGVD
SIDGSKVLSADVLTVDETNLGANATASFADNFAQAIDFGEDGAGSVTYALVLNGSNVGSGLYAIDNLDVSTADGDGIGRGGEIVLNQNGNVVTGSLGGVD
SIDGSKVLSADVXTVDETNLGXXATASFADXFAQAIDFGEDGAGSVTYALVLNGSNVGSGLYAIDNLDVXTADGDGXGRGGEIVLNQNGNVVTGSLGGVD
SIDGSKVLSADVXTVDETNLGXXATASFADXFAQAIDFGEDGAGSVTYALVLNGSNVGSGLYAIDNLDVXTADGDGXGRGGEIVLNQNGNVVTGSLGGVD
SIDGSKVLSADVXTVDETNLGXXATASFADXFAQAIDFGEDGAGSVTYALVLNGSNVGSGLYAIDNLDVXTADGDGXGRGGEIVLNQNGNVVTGSLGGVD
SIDGSKVLSADVXTVDETNLGXXATASFADXFAQAIDFGEDGAGSVTYALVLNGSNVGSGLYAIDNLDVXTADGDGXGRGGEIVLNQNGNVVTGSLGGVD
SIDGSKVLSADVXTVDETNLGXXATASFADXFAQAIDFGEDGAGSVTYALVLNGSNVGSGLYAIDNLDVXTADGDGXGRGGEIVLNQNGNVVTGSLGGVD
SIDGSKVLSADVXTVDETNLGXXATASFADXFAQAIDFGEDGAGSVTYALVLNGSNVGSGLYAIDNLDVXTADGDGXGRGGEIVLNQNGNVVTGSLGGVD
SIDGSKVLSADVXTVDETNLGXXATASFADXFAQAIDFGEDGAGSVTYALVLNGSNVGSGLYAIDNLDVXTADGDGXGRGGEIVLNQNGNVVTGSLGGVD
SIDGSKVLSADVXTVDETNLGXXATASFADXFAQAIDFGEDGAGSVTYALVLNGSNVGSGLYAIDNLDVXTADGDGXGRGGEIVLNQNGNVVTGSLGGVD
SIDGSKVLSADVXTVDETNLGXXATASFADXFAQAIDFGEDGAGSVTYALVLNGSNVGSGLYAIDNLDVXTADGDGXGRGGEIVLNQNGNVVTGSLGGVD
SIDGSKVLSADVXTVDETNLGXXATASFADXFAQAIDFGEDGAGSVTYALVLNGSNVGSGLYAIDNLDVXTADGDGXGRGGEIVLNQNGNVVTGSLGGVD
SIDGSKVLSADVXTVDETNLGXXATASFADXFAQAIDFGEDGAGSVTYALVLNGSNVGSGLYAIDNLDVXTADGDGXGRGGEIVLNQNGNVVTGSLGGVD
SIDGSKVLSADVLTVDETNLGANATASFADNFAQAIDFGEDGAGSVTYALVLNGSNVGSGLYAIDNLDVSTADGDGIGRGGEIVLNQNGNVVTGSLGGVD
SIDGSKVLSADVLTVDETNLGANATASFADNFAQAIDFGEDGAGSVTYALVLNGSNVGSGLYAIDNLDVSTADGDGIGRGGEIVLNQNGNVVTGSLGGVD
SIDGSKVLSADVLTVDETNLGANATASFADNFAQAIDFGEDGAGSVTYALVLNGSNVGSGLYAIDNLDVSTADGDGIGRGGEIVLNQNGNVVTGSLGGVD
SIDGSKVLSADVLTVDETNLGANATASFADNFAQAIDFGEDGAGSVTYALVLNGSNVGSGLYAIDNLDVSTADGDGIGRGGEIVLNQNGNVVTGSLGGVD
SIDGSKVLSADVLTVDETNLGANATASFADNFAQAIDFGEDGAGSVTYALVLNGSNVGSGLYAIDNLDVSTADGDGIGRGGEIVLNQNGNVVTGSLGGVD
SIDGSKVLSADVLTVDETNLGANATASFADNFAQAIDFGEDGAGSVTYALVLNGSNVGSGLYAIDNLDVSTADGDGIGRGGEIVLNQNGNVVTGSLGGVD

YFTITIDEASGEVVFEQLASVWHANTANPDDQSALQALANSLVVRATVVDADGDQAVHDLDVSQGVFQVKDDGP
YFTITIDEASGEVVFDQLASVWHGNTANPDDQSALQALANSLVVRATVVDADGDQAVHDLDVSQGVFQVKDDGP
YFTITIDEASGEVVFEQLASVWHANTANPDDQSALQALANSLVVRATVVDADGDQAVHDLDVSQGVFQVKDDSP
YFTITIDEASGEVVFEQLASVWHANTANPDDQSALQALANSLVVRATVVDADGDQAVHDLDVSQGVFQVKDDGP
YFTITIDEASGEVVFEQLASVWHANTANPDDQSALQALANSLVVRATVVDADGDQAVHDLDVSQGVFQVKDDGP
YFTITIDEASGEVVFEQLASVWHANTANPDDQSALQALANSLVVRATVVDADGDQAVHDLDVSQGVFQVKDDGP
YFTITIDEASGEVVFDQLASVWHGNTANPDDQSALQALANSLVVRATVVDADGDQAVHDLDVSQGVFQVKDDGP
YFTITIDEASGEVVFEQLASVWHANTANPDDQSALQALANSLVVRATVVDADGDQAVHDLDVSQGVFQVKDDGP
YFTITIDEASGEVVFDQLASVWHGNTANPDDQSALQALANSLVVRATVVDADGDQAVHDLDVSQGVFQVKDXGP
YFTITIDEASGEVVFXQLASVWHXNTANPDDQSALQALANSLVVRATVVDADGDQAVHDLDVSQGVFQVKDXGP
YFTITIDEASGEVVFXQLASVWHXNTANPDDQSALQALANSLVVRATVVDADGDQAVHDLDVSQGVFQVKDXGP
YFTITIDEASGEVVFXQLASVWHXNTANPDDQSALQALANSLVVRATVVDADGDQAVHDLDVSQGVFQVKDXGP
YFTITIDEASGEVVFXQLASVWHXNTANPDDQSALQALANSLVVRATVVDADGDQAVHDLDVSQGVFQVKDXGP
YFTITIDEASGEVVFXQLASVWHXNTANPDDQSALQALANSLVVRATVVDADGDQAVHDLDVSQGVFQVKDXGP
YFTITIDEASGEVVFXQLASVWHXNTANPDDQSALQALANSLVVRATVVDADGDQAVHDLDVSQGVFQVKDXGP
YFTITIDEASGEVVFXQLASVWHXNTANPDDQSALQALANSLVVRATVVDADGDQAVHDLDVSQGVFQVKDXGP
YFTITIDEASGEVVFXQLASVWHXNTANPDDQSALQALANSLVVRATVVDADGDQAVHDLDVSQGVFQVKDXGP
YFTITIDEASGEVVFXQLASVWHXNTANPDDQSALQALANSLVVRATVVDADGDQAVHDLDVSQGVFQVKDXGP
YFTITIDEASGEVVFXQLASVWHXNTANPDDQSALQALANSLVVRATVVDADGDQAVHDLDVSQGVFQVKDXGP
YFTITIDEASGEVVFXQLASVWHXNTANPDDQSALQALANSLVVRATVVDADGDQAVHDLDVSQGVFQVKDDGP
YFTITIDEASGEVVFDQLASVWHANTANPDDQSALQALANSLVVRATVVDADGDQAVHDLDVSQGVFQVKDDGP
YFTITIDEASGEVVFEQLASVWHANTANPDDQSALQALANSLVVRATVVDADGDQAVHDLDVSQGVFQVKDDGP
YFTITIDEASGEVVFEQLASVWHANTANPDDQSALQALANSLVVRATVVDADGDQAVHDLDVSQGVFQVKDDGP
YFTITIDEASGEVVFDQLASVWHGNTANPDDQSALQALANSLVVRATVVDADGDQAVHDLDVSQGVFQVKDDGP
YFTITIDEASGEVVFDQLASVWHGNTANPDDQSALQALANSLVVRATVVDADGDQAVHDLDVSQGVFQVKDDGP
YFTITIDEASGEVVFDQLASVWHANTANPDDQSALQALANSLVVRATVVDADGDQAVHDLDVSQGVFQVKDDGP

L5 repeats
SVSSTKLTGVVDEDGLAGGIAGGTGDVAGQAVAASGNVATLFQSGADAPLSYSLNPNTSGLPALSSGGVALTYAVSGGTLTASAGSTQVFTFTLNANGNY
VASTTKLTGVVDEDGLAGGIAGGTGDVAGQAVAASGNVATLFQSGADAPLSYSLNPNTSGLPALSSGGVALTYAVSGGTLTASAGSTQVFTFTLNANGNY
IASATKLTGVVDEDGLAGGIAGGTGDVAGQAVAASGNVATLFQSGADAPLSYSLNPNTSGLPALSSGGVALTYAVSGGTLTASAGSTQVFTFTLNANGNY
VASATKLTGVVDEDGLAGGIAGGTGDVAGQAVAASGNVATLFQSGADAPLSYSLNPNTSGLPALSSGGVALTYAVSGGTLTASAGSTQVFTFTLNANGNY
VASATKLTGVVDEDGLAGGIAGGTGDVAGQAVAASGNVATLFQSGADAPLSYSLNPNTSGLPALSSGGVALTYAVSGGTLTASAGSTQVFTFTLNANGNY
VASATKLTGVVDEDGLAGGIAGGTGDVAGQAVAASGNVATLFQSGADAPLSYSLNPNTSGLPALSSGGVALTYAVSGGTLTASAGSTQVFTFTLNANGNY
VASATKLTGVVDEDGLAGGIAGGTGDVAGQAVAASGNVATLFQSGADAPLSYSLNPNTSGLPALSSGGVALTYAVSGGTLTASAGSTQVFTFTLNANGNY
IASATKLTGVVDEDGLAGGIAGGTGDVAGQAVAASGNVATLFQSGADAPLSYSLNPNTSGLPALSSGGVALTYAVSGGTLTASAGSTQVFTFTLNANGNY
IASATKLTGVVDEDGLAGGIAGGTGDVAGQAVAASGNVATLFQSGADAPLSYSLNPNTSGLPALSSGGVALTYAVSGGTLTASAGSTQVFTFTLNANGNY

TFTLLAKLDHPAGADENDITINLGSVIRATDSDGDTVVAAADGLVITVDDDTP
TFTLLAKLDHPAGADENDITINLGSVIRATDSDGDTVVAAADGLVITVDDDTP
TFTLLAKLDHPAGADENDITINLGSVIRATDSDGDTVVAAADGLVITVDDDTP
TFTLLAKLDHPAGADENDITINLGSVIRATDSDGDTVVAAADGLVITVDDDTP
TFTLLAKLDHPAGADENDITINLGSVIRATDSDGDTVVAAADGLVITVDDDTP
TFTLLAKLDHPAGADENDITINLGSVIRATDSDGDTVVAAADGLVITVDDDTP
TFTLLAKLDHPAGADENDITINLGSVIRATDSDGDTVVAAADGLVITVDDDTP
TFTLLAKLDHPAGADENDITINLGSVIRATDSDGDTVVAAADGLVITVDDDTP
TFTLLAKLDHPAGADENDITINLGSVIRATDSDGDTVVAAADGLVITIDDDMP

KAFTPEEGFVTNQAGIVRTFDLDFDANIDNNVGADQPGTIAFSGITNGQLVTGTVDGVPNQTLTSGGSAIHYYVSGNNVVEGWINGGPGDVGSTIVFRTT
LQPDMNYNASNDTYKFELFQPISTSTNVSITNFNGVNATSREFIYLENASAPGEDILFSGYIRNDNGSFTDATVNTSTQGIGVNNQNMNDRENLRLDFVR
NASTTGTNQNMTYEYDDHYTVNNFSFKIVQVTGSPPAGSIEVWVRAYNADDDDPTNNTVSSANNLAHQNALRDDPQVALTQILVNGVPVTPTAVNASGGY
LISGLNLNDTITLRSANGYDRVEIENARSGAHGVSNSSLNGETFDIGLFSYNTIKTTPSEININMGLSLTDSDGDKVTSSIEINLSPSVFKVGENVDDTS
SSNVSHRVGGDTGVIDSSGGADILVGDVGGVEIIGTTARIAFILDESGSMDQNFGGTTRLEVLKQTMTDILTELSNTPNASITVHLVKFASVVNGTGTFE
ITGGELQQALDFISGLQIQQGLLAGTNYEAALGQTLQWYNSQSGTVDVQQTLFFTDGAPTFYMDGNSTEYTNIARVYGNGSQTEEVLWENLFGEHAGGQA
TSDRINNLTESDSRDLDGTQSYSVDTNGDGIFETQSVNSRSFGTTQNTNDYVRSIADTFNEVDALQAHGPLRAVSIADNFSVYLQEIDSTGQPYIADSPE
VLQDILDELNPFNVLLSAGSDTIQANEENDLIFGDVLFTDKLAEDEGLDLPKGSGWAVFEELEANHGWSRQDTLDYIKNHADELGRETVLSSGSKRSGGH
DTISGGQGDDRIYGQEGNDIIDAGSGDDVIVGGTGNDTLTGGAGADQFVLVKGQGGASAGAAPIDTITDFEVNIDKIVINGNNITGVSVSTPVSNTYTIT
VSYSDAATTEHFKVTLSNGALLNDSGKTHLNGVVISGGTATIDGTIVGAVLYLDLNANNQEDEGERLGITNQYGHVEWVVDLSKFDVNGDGQYVIGEARA
VQTGGFDIDTGLSYEINLYGPVGSAVISPLTSLLQAQLEAGMDYETANAALVARLGLPEGTQIISFSPITGSGEVLAQNAGVMTAAIQFAEIAAIHYSTD
ESHVSFAVFEAISKALFELPEGLVADFSDQGFLQSISNHLGLDALVTADMIDYMAAGQKALEASILTLAPGEDALTAISKIQHVTQGIYAQAIEMALIGY
LPLDALKNMSAVLKAYADGDITDEQLNSFEDKLSAVVVNWEENNVMDTSHQASLQTDRTVDDSSVKSEVNHEMAQEVNAVINDFMDEHNISLYYYAQQAQ
MPQEAQEQQPTEDQAEETVMHDDVVQDLLLDDHSLTMLANNPQLNGGNGNDVLHGTTGNDFIRGGQGNDTMTGGGGVDTFFWLSGDDDGGVDTITDFKAN
PVDQSSDASVLNLSDLLSDADLETNSLDNYLNVSTTEEGDTTIKVDPNGNGNFDAPAQTIILEDVDLTAVFATNNSHDIVNQMIANGNLIVEQ

Leg-6 SNV10957.1
MLAESVIGIVRAVNGLLEKVNAQGQASIVKSGARLEEGDVLTLLSGEAYIQFIHGFPEALALEKPVKLDGVSPTLQYGVEDLKEQMVQEAIAKGIDPSII
LDVLGSAAAGAEAVGSGGDAFIIDPLFGFGQVTAGYPTGPIAFGYEADTQHLFWYVPEETGAIAESELTTEPESIPQIPQFNSNQATVIVFEDALTGGIQ
DSAGQVTTARTSLSPLISSSSEVASSFAFNTNLSALPKLKSGGIELDYILSSDRLALSASMPDGTNVARFELTADGQLTQILIGAIDHPTADSDDSEWMR
LDLSPVIDVTFTRTSDGTVLESRTLPDNAVVAGIQDDVPIARAQLTNNEILLDETIGMKVGDADAANDDFNPTTTADPFNNTYGRPIGLVQNANLLDTST
SEMGGDYKNATMTHLLKITDAVSGLQTTNGTPINLFLESNGDITGRAGDIGAPAVFAIRMNPNTGAISVAQYGPIKQFDTNSHDEAVDLTGRISAVVRVT
DGDGDVSSAEIPIGQLIIFEDDGP

L4 repeats
SIDGSKVLPADVLAVDETNLGVNATASFADNFAQAIDFGEDGAGSVSYALVLNGNNVGSGLYAIDNLDVSTADGDGIGRGGEIVLNQNGNVITGSLNGTD
SINGSKVLSADVLAVDETNLGVNATASFADNFAQAIDFGEDGAGSVSYALVLNGNNVGSGLYAIDNLDVSTADGDGIGRGGEIVLNQNGNVITGSLNGTD
SIDGSKVLSADVLAVDETNLGVNATASFADNFAQAIDFGEDGAGSVSYALVLNGNNVGSGLYAIDNLDVSTADGDGIGRGGEIVLNQNGNVITGSLNGTD
SIDGSKVLSADVLAVDETNLGVNATASFADNFAQAIDFGEDGAGSVSYALVLNGNNVGSGLYAIDNLDVSTADGDGIGRGGEIVLNQNGNVITGSLNGTD
SIDGSKVLSADVLAVDETNLGVNATASFADNFAQAIDFGEDGAGSVSYALVLNGNNVGSGLYAIDNLDVSTADGDGIGRGGEIVLNQNGNVITGSLNGTD
SIDGSKVLSADVLAVDETNLGVNATASFADNFAQAIDFGEDGAGSVSYALVLNGNNVGSGLYAIDNLDVSTADGDGIGRGGEIVLNQNGNVITGSLNGTD
SIDGSKVLSADVLAVDETNLGVNATASFADNFAQAIDFGEDGAGSVSYALVLNGNNVGSGLYAIDNLDVSTADGDGIGRGGEIVLNQNGNVITGSLNGTD
SIDGSKVLSADVLAVDETNLGVNATASFADNFAQAIDFGEDGAGSVSYALVLNGNNVGSGLYAIDNLDVSTADGDGIGRGGEIVLNQNGNVITGSLNGTD
SINGSKVLSADVLAVDETNLGVNATASFADNFAQAIDFGEDGAGSVSYALVLNGNNVGSGLYAIDNLDVSTADGDGIGRGGEIVLNQNGNVITGSLNGTD
SIDGSKVLSADVLAVDETNLGVNATASFADNFAQAIDFGEDGAGSVSYALVLNGNNVGSGLYAIDNLDVSTADGDGIGRGGEIVLNQNGNVITGSLNGTD
SIDGSKVLSADVLAVDETNLGVNATASFADNFAQAIDFGEDGAGSVSYALVLNGNNVGSGLYAIDNLDVSTADGDGIGRGGEIVLNQNGNVITGSLNGTD
SIDGSKVLSADVLAVDETNLGVNATASFADNFAQAIDFGEDGAGSVSYALVLNGNNVGSGLYAIDNLDVSTADGDGIGRGGEIVLNQNGNVITGSLNGTD
SIDGSKVLSADVLAVDETNLGVNATASFADNFAQAIDFGEDGAGSVSYALVLNGNNVGSGLYAIDNLDVSTADGDGIGRGGEIVLNQNGNVITGSLNGTD
SIDGSKVLSADVLAVDETNLGVNATASFADNFAQAIDFGEDGAGSVSYALVLNGNNVGSGLYAIDNLDVSTADGDGIGRGGEIVLNQNGNVITGSLNGTD
SIDGSKVLSADVLAVDETNLGVNATASFADNFAQAIDFGEDGAGSVSYALVLNGNNVGSGLYAIDNLDVSTADGDGIGRGGEIVLNQNGNVITGSLNGTD
SIDGSKVLSADVLAVDETNLGVNATASFADNFAQAIDFGEDGAGSVSYALVLNGNNVGSGLYAIDNLDVSTADGDGIGRGGEIVLNQNGNVITGSLNGTD
SIDGSKVLSADVLAVDETNLGVNATASFADNFAQAIDFGEDGAGSVSYALVLNGNNVGSGLYAIDNLDVSTADGDGIGRGGEIVLNQNGNVITGSLNGTD
SIDGSKVLSADVLAVDETNLGVNATASFADNFAQAIDFGEDGAGSVSYALVLNGNNVGSGLYAIDNLDVSTADGDGIGRGGEIVLNQNGNVITGSLNGTD
SIDGSKVLSADVLAVDETNLGVNATASFADNFAQAIDFGEDGAGSVSYALVLNGNNVGSGLYAIDNLDVSTADGDGIGRGGEIVLNQNGNVITGSLNGTD
SIDGSKVLSADVLAVDETNLGVNATASFADNFAQAIDFGEDGAGSVSYALVLNGNNVGSGLYAIDNLDVSTADGDGIGRGGEIVLNQNGNVITGSLNGTD

YFTITIDAASGTVTFDQLASVWHANTANPDDQSALQALANSLVVRATVVDADGDQAAYDLDVSQGVFQVKDDGP
YFTITIDAASGTVTFDQLASVWHANTANPDDQSALQALANSLVVRATVVDADGDQAAYDLDVSQGVFQVKDDGP
YFTITIDAASGTVTFDQLASVWHANTANPDDQSALQALANSLVVRATVVDADGDQAAYDLDVSQGVFQVKDDGP
YFTITIDAASGTVTFDQLASVWHANTANPDDQSALQALANSLVVRATVVDADGDQAAYDLDVSQGVFQVKDDGP
YFTITIDAASGTVTFDQLASVWHANTANPDDQSALQALANSLVVRATVVDADGDQAAYDLDVSQGVFQVKDDGP
YFTITIDAASGTVTFDQLASVWHANTANPDDQSALQALANSLVVRATVVDADGDQAAYDLDVSQGVFQVKDDGP
YFTITIDAASGTVTFDQLASVWHANTANPDDQSALQALANSLVVRATVVDADGDQAAYDLDVSQGVFQVKDDGP
YFTITIDAASGTVTFDQLASVWHANTANPDDQSALQALANSLVVRATVVDADGDQAAYDLDVSQGVFQVKDDGP
YFTITIDAASGTVTFDQLASVWHANTANPDDQSALQALANSLVVRATVVDADGDQAAYDLDVSQGVFQVKDDGP
YFTITIDAASGTVTFDQLASVWHANTANPDDQSALQALANSLVVRATVVDADGDQAAYDLDVSQGVFQVKDDGP
YFTITIDAASGTVTFDQLASVWHANTANPDDQSALQALANSLVVRATVVDADGDQAAYDLDVSQGVFQVKDDGP
YFTITIDAASGTVTFDQLASVWHANTANPDDQSALQALANSLVVRATVMDADGDQAAYDLDVSQGVFQVKDDGP
YFTITIDAASGTVTFDQLASVWHANTANPDDQSALQALANSLVVRATVVDADGDQAAYDLDVSQGVFQVKDDGP
YFTITIDAASGTVTFDQLASVWHANTANPDDQSALQALANSLVVRATVVDADGDQAAYDLDVSQGVFQVKDDGP
YFTITIDAASGAVTFDQLASVWHANTANPDDQSALQALANSLVVRATVMDADGDQAAYDLDVSQGVFQVKDDGP
YFTITIDAASGAVTFDQLASVWHANTANPDDQSALQALANSLVVRATVMDADGDQAAYDLDVSQGVFQVKDDGP
YFTITIDAASGAVTFDQLASVWHANTANPDDQSALQALANSLVVRATVMDADGDQAAYDLDVSQGVFQVKDDGP
YFTITIDAASGAVTFDQLASVWHANTANPDDQSALQALANSLVVRATVMDADGDQAAYDLDVSQGVFQVKDDGP
YFTITIDAASGAVTFDQLASVWHANTANPDDQSALQALANSLVVRATVVDADGDQAAYDLDVSQGVFQVKDDGP
YFTITIDAASGAVMFDQLASVWHANTANPDDQSALQALANSLVVRATVVDADGDQAAYDLDVSQGVFQVKDDGP

L2 repeat

SVAVNVGNEAGILLTTQDADTIGTATDAAVTSANFGNVFSNTPTYGADGAGSAVMSYSLGLIAASGADSGLDSNGSNIYLYDISGTIVGSTSATQAGVNA
GNTVFDLSVDGAGVVTLRQHAEIDHPIADDPSASGAPFDDQLAVLGNNLVSLTATALTTDGDGDTATASERIDLGGNVRFADDGP

L1 repeats

SVTMTVSDTNAITLNTQDAETIGALSDSDSASFATAFAVTPNYGADGAGTTVTTYALSVSAQGVDSGLDNNGNNIYLYNIAGSVVGSTSATQADITTGNT
SVTMTVSDNNAITLNTQDAETIGALSDSDSASFATAFAVTPNYGADGAGTTVTTYALSVSAQGVDSGLDNNGNNIYLYNIAGSVVGSTSATQAGITTGNT
SVTMTVSDTNAITLNTQDAETIGALSDSDSASFAAAFAVTPNYGADGAGTTVTTYALSVSAQGVDSGLDNNGNNIYLYNIAGSVVGSTSATQVGITTGNT
SVTMTVSDNNAITLNTQDAETIGALSDSDSASFAAAFAVTPNYGADGAGTTVTTYALSVSAQGVDSGLDNNGNNIYLYNIAGSVVGSTSATQADITTGNT
SVTMTVSDNNAITLNTQDAETIGALSDSDSASFATAFAVTPNYGADGAGTTVTTYALSVSAQGVDSGLDNNGNNIYLYNIAGSVVGSTSATQADITTGNT
SVTMTVSDNNAITLNTQDAETIGALSDSDSASFATAFAVTPNYGADGAGTTVTTYALSVSAQGVDSGLDNNGNNIYLYNIAGSVVGSTSATQAGITTGNT
SVTMTVSDTNAITLNTQDAETIGALSDSDSASFAAAFAVTPNYGADGAGTTVTTYALSVSAQGVDSGLDNNGNNIYLYNIAGSVVGSTSATQVGITTGNT
SVTMTVSDNNAITLNTQDAETIGALSDSDSASFAAAFAVTPNYGADGAGTTVTTYALSVSAQGVDSGLDNNGNNIYLYNIAGSVVGSTSATQADITTGNT
SVTMTVSDNNAITLNTQDAETIGALSDSDSASFAAAFAVTPNYGADGAGTTVTTYALSVSAQGVDSGLDNNGNNIYLYNIAGSVVGSTSATQVGITTGNT

IFSLGVNSSSGVVTLTQHQEVDHGLPGASSNYAAQEAILNTGLVFLNATAMTTDGDGDTATASASLDLGGNVKFDDDGP
IFSLGVNSSSGVVTLTQHQEVDHGLPGASSNYAAQEAILNTGLVFLNATAVTTDGDGDTATASASLDLGGNVKFDDDGP
IFSLGVNSSSGVVTLTQHQEVDHGLPGASSNYAAQEAILNTGLVFLNATAVTTDGDGDTATASASLDLGGNVKFDDDGP
IFSLGVNSSSGVVTLTQHQEVDHGLPGASSNYAAQEAILNTGLVFLNATAVTTDGDGDTATASASLDLGGNVKFDDDGP
IFSLGVNSSSGVVTLTQHQEVDHGLPGASSNYAAQEAILNTGLVFLNATAMTTDGDGDTATASASLDLGGNVKFDDDGP
IFSLGVNSSSGVVTLTQHQEVDHGLPGASSNYAAQEAILNTGLVFLNATAVTTDGDGDTATASASLDLGGNVKFDDDGP
IFSLGVNSSSGVVTLTQHQEVDHGLPGASSNYAAQEAILNTGLVFLNATAVTTDGDGDTATASASLDLGGNVKFDDDGP
IFSLGVNSSSGVVTLTQHQEVDHGLPGASSNYAAQEAILNTGLVFLNATAVTTDGDGDTATASASLDLGGNVKFDDDGP
IFSLGVNSSSGVVTLTQHQEVDHGLPGASSNYAAQEAILNTGLVFLNATAVTTDGDGDTATASASLDLGGNVKFDDDGP

L8 repeat

TVGSISLVADEDNLPRGNNDTASGDAAQSNLTGTLPVNFGADGAGSIDFQGMHGMSAVIGNDNITYNWNASTNTLTAYRTGGALGVNDVFKIVVNPTTGQ
YTFTLLAAINHHAVADNTEGLVDPFVNLNYRVIDGDGDTAIGTLKVTIDDDVP

KAITPEEGFVTNQAGIVRTFDLDFDANIDNNVGADQLGTITFSGITNGQVVTGTVDGVPNQTLTSGGSAIHYYVSGNNVVEGWINGGPGDVGSTIVFRTT
LQPDMNYNASNDTYKFELFQPISTSTSVSIANFSGVNSTSREFIYLEDASSSGEDILFSAYIRNDNGSFTDATANTSTQGIGVNNQNMNDRENLRLDFVR
NVSTNGTTSNMTYEYDDHYTVNNFSFKIVQVTGSLPAGSIEVWVRAYNADDDDPSNNTVSSANNLAHQDALRDDPQVALTQILVNGVPVTPTAVNASGGY
LISGLNLNDTITLRSANGYDRVEIENARSGAHGVSNSNLNGETFDIGLFSYNTIKTTPSEININMGLSLTDSDGDKINSSIEINLAPSVFKVGENVDDTS
SSNVPHRVGGDTGVIDGSGGADILVGDVGGVEVIGTTARLAFILDESGSMSQNFGGTTRLEVLKQAMTDILTELSNTPNASITVHLVKFASIVNGTGTFE
ITGGGLQQALDFISGLQIQQGLLAGTNYEAALGQTVQWFSSQSGTVDVQQTLFFTDGVPTFYMDGNSTEYTNLARVYGNGSQTEEVLWENLFGEHAGGQA
TSDRINNLTETDSRDLDGLQSYSIDTNSDGIFETQSVNSRSSGTTQTTNDLVSSIADTFNEVQALQAYGTLRAVSIADNANVYLQEIDSTGQPYLANSPE
VLQDILDELNPFNVLLAAGSDTIQANQEDDLIFGDVLFTDKLAEDEGVDLPKGSGWAVFEELEANHGWSRQDTLDYIKNHADELGRETVLSSGKRSGGHD
TISGGQGDDRIYGQEGNDVIDAGSGDDVIVGGTGNDTLTGGSGADQFVLVKGQGGASAGAAPIDTITDFEVSIDKIVINGNNITGVNVSTPVSNTYTITV
NYSDAATEHFKVTLSNGALLNDSGKTHLNGVVISGGTATIDGTIVGAVLYLDLNANNQEDAGERLGITNQYGHVEWVVDLSKFDVNGDGQYVIGEARAVQ
TGGFDIDTGLSYEINLYGPVGSAVISPLTSLLQAQLEAGMDYETANAALVARLGLPEGTQIISFNPITGSGEVLAQNAGVMTAAIQFAEIAAIHYSTDER
HVSFAVFEAISKALLELPEGVVADFGDEGFLQSISNYLGLDALVTSDMIDYMATSQKALEASILTLAPGEDALTAISKIQHVTQGIYAQAIEMALIGYLP
LDALKNMSAVLKAYADGDITDEQLNSFEDKLSAVIVNWEDNNVTDTSHQATLQTERTVDDSSVKSEVNHEMAQEVNAVINDFMDEHNISLYYYDQQAQLP
QEAQEQQPIEDQAEETVMHDDIVQDLLLDDHSLTMLANNPQLNGGNGNDVLHGTTGNDFIRGGQGNDTMTGGGGVDTFFWLSGDDDGGVDTITDFKANPV
DQSSDASVLNLSDLLSDADLETNSLDNYLNVSTTEEGDTAIKVDPNGNGNFDAPAQTIILEDVDLTAVFATNNSHDIVNQMIANGNLIVEQ

Leg-7 CCD04807.1

MLVESVIGIVRAVNGLLEKVNAQGQASLVKSGARLQEGDVLTLLSGEAYIQFIHGFPEALALGKPVNLYGVSPALQYGVEELNEQLVQEALAKGIDPSVI
LDVLGSAAAGAEAVGSGGDAFIIDPLFGFGQVTAGYPTGPIAFGYEADTQHLFWYVPEETGAIAESELTTEPESIPQIPQFNSNQATVIVFEDALTGGIQ
DSAGQVTTARASLSPLISSSSEVASSFAFNTNLSALPTLKSGGIELDYILSSDRLALTASMPDGTNVARFELTADGQLTQILIGAIDHPTSDSDDNEWMR
FDLSPVIDVTFTRTSDGTVLESRTLPDNAVVAGIQDDVPIARAQLTNNEILLDETVGVKVGDADAANDDFNPTTTADPFNNAYGIPTGLVQNANLLDTST
SEMGGDYKNATMTHLLKITDAVSGLQTTNGTPINLFLESNGDITGRAGDIGAPALFAIRMNPNTGAISVAQYGPIKQFDTNSHDEAVDLTGRISAVVTAK
DSDGDVSNVEIPIGQLIIFEDDGP

L6 repeats

SIVVSGATQTLTVDESVLTTNDTQSFAGLFTPSFGADGAAAANALSYSLGVSANGAASGVLDTASGNQVFLFLENGIVVGREGSDALDAATGDVVFNISV
SIVVSGATQTLTVDESVLTTNDTQSFAGLFTPSFGADGAAAANALSYSLGVSANGAASGVLDTASGNQVFLFLENGIVVGREGSDALDAATGDVVFNISV
SIVVSGATQTLTVDESVLATNDTQSFAGLFTPSFGADGAAAANALSYSLGVSANGAASGVLDTASGNQVFLFLENGIVVGREGSDALDAATGDVVFNISV
SIVVSGATQTLTVDESVLATNDTQSFAGLFTPSFGADGAAAANALSYSLGVSANGAASGVLDTASGNQVFLFLENGIVVGREGSDALDAATGDVVFNISV
SIVVSGATQTLTVDESVLTTNDTQSFAGLFTPSFGADGAAAANALSYSLGVSANGAASGVLDTASGNQVFLFLENGIVVGREGSDALDAATGDVVFNISV
SIVVSGATQTLTVDESVLTTNDTQSFAGLFTPSFGADGAAAANALSYSLGVSANGAASGVLDTASGNQVFLFLENGIVVGREGSDALDAATGDVVFNISV
SIVVSGATQTLTVDESVLTTNDTQSFAGLFTPSFGADGAAAANALSYSLGVSANGAASGVLDTASGNQVFLFLENGIVVGREGSDALDAATGDVVFNISV
SIVVSGATQTLTVDESVLTTNDTQSFAGLFTPSFGADGAAAANALSYSLGVSANGAASGVLDTASGNQVFLFLENGIVVGREGSDALDAATGDVVFNISV
SIVVSGATQTLTVDESVLTTNDTQSFAGLFTPSFGADGAAAANALSYSLGVSANGAASGVLDTASGNQVFLFLENGIVVGREGSDALDAATGDVVFNISV
SIVVSGATQTLTVDESVLATNDTQSFAGLFTPSFGADGAAAANALSYSLGVSANGAASGVLDTASGNQVFLFLENGIVVGREGSDALDAATGDVVFNISV
SIVVSGATQTLTVDESVLATNDTQSFAGLFTPSFGADGAAAANALSYSLGVSANGAASGVLDTASGNQVFLFLENGIVVGREGSDALDAATGDVVFNISV

DGSGNVTLDQVRAVVHDNPLDPDESTGPTQLSAANLVTLTAIATDKDGDSASATANIGLSFNFEDDGP
DGSGNVTLDQVRAVVHDNPLDPDESTGPTQLSAANLVTLTAIATDKDGDSASATANIGLSFNFEDDGP
DGSGNVTLDQVRAVVHDNPLDPDESTGPTQLSAANLVTLTAIATDKDGDSASATANIGLSFNFEDDGP
DGSGNVTLDQVRAVVHDNPLDPDESTGPTQLSAANLVTLTAIATDKDGDSASATANIGLSFNFEDDGP
DGSGNVTLDQVRAVVHDNPLDPDESTGPTQLSAANLVTLTAIATDKDGDSASATANIGLSFNFEDDGP
DGSGNVTLDQVRAVVHDNPLDPDESTGPTQLSAANLVTLTAIATDKDGDSASATANIGLSFNFEDDGP
DGSGNVTLDQVRAVVHDNPLDPDESTGPTQLSAANLVTLTAIATDKDGDSASATANIGLSFNFEDDGP
DGSGNVTLDQVRAVVHDNPLDPDESTGPTQLSAANLVTLTAIATDKDGDSASATANIGLSFNFEDDGP
DGSGNVTLDQVRAVVHDNPLDPDESTGPTQLSAANLVTLTAIATDKDGDSASATANIGLSFNFEDDGP
DGSGNVTLDQVRAVVHDNPLDPDESTGPTQLSAANLVTLTAIATDKDGDSASATANIGLSFNFEDDGP
DGSGNVTLDQVRAVVHDNPLDPDESTGPTQLSAANLVTLTAIATDKDGDSASATANIGLSFNFEDDGP

L5 repeats

VASTNKLTGVVDEDGLTGGIAGGVGDVAGQAVAASGNVATLFQSGADAPLTYSLNASTSGLPALSSGGVALTYAVSGNTLTASAGSTQVFTFTLNANGNY
IASANKLTGVVDEDGLTGGIAGGVGDVAGQAVAASGNVATLFQSGADAPLTYSLNTNTSGLPALSSGGVALTYAVSGDTLTASAGSTQVFTFTLNANGNY
IASANKLTGVVDEDGLTGGIAGGVGDVAGQAVAASGNVATLFQSGADAPLTYSLNTNTSGLPALSSGGVALTYAVSGDTLTASAGSTQVFTFTLNANGNY

SFTLLAKLDHPAGADENDITINLGSVIRATDSDGDTVTAAADGLVITVDDDTP
SFTLLAKLDHPAGADENDITINLGSVIRATDSDGDTVTAAADGLVITVDDDTP
SFTLLAKLDHPAGADENDITINLGSVIRATDSDGDTVTAAADGLVITVDDDTP

VAITPEEGFVSNKAGVVKVFNLDLDSNIGNNVGADQPGSIRFINVVDGQQAQGIIDGELVNLTTVNGQLIKLYISNDGMIITGSTATSEAAVNSGNTVFK
VTMLPDNDYNLANDKYSVEMFGKIGVINTVNFSDLAGDSPAGNVGFKIISDQDFPGYEVLITPKDRVNPDDTVGGTVNSSSTDISISNQWIEQGSGDSEI
LRLDYGQFAIVNNKFNIEKHFIVNGASFEIHQIQGGPSNRASINVKAFDADDDNQLNDDPLDAITRIVIFTSSGGVETVIADVTSDTSFGGMTFDFQGIN
GVNITGLDDGYRVRAYTADGYNRLEIDNISSTPFSIGGLGIQAIIDTEKEVKMTYDVRLTDSDGDSVDSSFDVNIAPSLLKVGENINDTSSSTVPHTVGN
DVGVIDGGRGADVLVGDVGGVKDVRIAFVLDESGSMSQNFGGSTRMDVLKQVMTDVLTELGNTPNANITVHLVKFASIVNGTGTFNITGGELQNALDFIS
GLQIQQGNLAGTNYEAALGQTALWFNSLTGAADVQQTLFLTDGAPTLYMDGNSTDYTNINAVYGHGNQTEEVLWENLFGEHAGNQATVDLFNYLNESNSR
DLDGTQSYSVDTDGDGDFETVSVNARSSGTTQSTSDLVRSTADNVNEVQALQAHGTLRAVSIANNVSVYLQDIDSTGQPYVASSPGLLQDILNELNPFNS
LNPVGSDIIQGNEADDLIFGDTLFTDKLAEDEGLNLRKGSGWDVFEELEASHGWSRQDTLNYIASHADELGKETVLSNGSTRSGGHDTISGGQGNDRIYG
QEGNDIIDGGAGNDVIVGGTGNDTLTGGSGADQFVFFRGHGSNNAGTAPTDIITDFEVNIDKIVINANNIIGVSVSNPVSNPDLSKTYTITVNYSDAPSI
EHFKVTLSNGALLNDSGKTHLNGVVISGGTATIDGTIVGAVLYLDLNANNQEDEGERLGITNQYGHVEWVVDLSKFDVNGDGQYVIGEARAVQTGGFDID
TGLSYEINLYGPVGSAVISPLTSLLQAQLEAGMDYETANAALVARLGLPEGTQIISFNPITGSGEVLAQNAGVMTAAIQFAEIAAIHYSTDERHVSFAVF
EAISKALLELPEGVVADFGDEGFLQSISNYLGLDALVTSEMIDYMATSQKALEASILTLAPGEDALTAISKIQHVTQGIYAQAIEMALIGYLPLDALKNM
SAVLKAYADGDITDEQLNSFEDKLSAVIVNWEDNNVTDTSHQATLQTERTVDDSSVKSEANHEMAQEVNAVINDFMDEHDISLYYYDQQAQMTQETQEEQ
QPVEAQAEETVIHDDVVQDLLLDDHSLTMLANNPELNGGNGNDVLHGTTGNDFIRGGQGNDTMTGGGGVDTFFWLSGDDDGGVDTITDFKANPVDQSSDA
SVLNLSDLLSDADLETNSLDNYLNVSTTEEGDTAIKVDPNGNGNFDAPAQTIILEDVDLTAVFATNNSHDIVNQMIANGNLIVEQ

Leg-8 CAH11847.1

MLAESVIGIVRAVNGLLEKVNAQGQASLVKSGARLQEGDVLTLLSGEAYIQFIHGFPEALALGKPVNLYGVSPALQYGVEELNEQLVQEALAKGIDPSVI
LDVLGSAAAGAEAVGSGGDAFIMDPLFGFGHVTAGYPTGPISFAYEADTQQLFWFVPEETGVIAESELTQEPESIPQIPQFTTNQAVLTVFEDALPSGIA
DSAGQARIASSSLSSLLTSSADVAASFAFNSNLSALPTLKSGGIDLSYELSADKRTLTLRESNTQGPGAEVMKFELTADGQLTQTLMNSIDHPTADSDDG
EWMRLDLSPLIDVTFTRTIDGSVLESRTLPANAVVAGIQDDVPIARAQLTNNEILLDETIGMKVGDVDAANDDFNPTTTADPFNNTYGIPIGLVQNANLL
DTSTSEMGGDYKNATMTHLIKITDAVSGLQTTDGTPVNLFLESNGDISGRAGDIGAPAVFAIRMNPNTGAITVAQYGSIKQFDTNSYDEAVDLTGRISVV
VTAKDSDGDVSNAEIPIGQLIIFEDDGP

L7 repeats

VVDMAVKAGAALTLDETKGVKAGDANANDEAASADANDIGYAKLVGSDLFTLTKDAGSDGXQSTLFKLLVXAPASGLVDTATXQAIVLSANAGGTEVLGK
VVDMAVKAGAALTLDETKGVKAGDANANDEAASADANDIGYAKLVGSDLFTLTKDAGSDGEQSTLFKLLVSAPASGLVDTATNQAIVLSANAGGTEVLGK
VVDMAVKAGAALTLDETQGMKAGDANANDEAASADANDIGYAKLVGSDLFTLTKDAGSDGEQSTLFKLLVSAPASGLVDTATNQAIVLSANAGGTEVLGK
VVDMAVKAGAALTLDETQGMKAGDANANDEAASADANDIGYAKLVGSDLFTLTKDAGSDGEQSTLFKLLVGAPASGLVDTATNQAIVLSANAGGTEVLGK
VVDMAVKAGAALTLDETKGVKAGDANANDEAASADANDIGYAKLVGSDLFTLTKDAGSDGEQSTLFKLLVSAPASGLVDTATNQAIVLSANAGGTEVLGK
VVDMAVKAGAALTLDETKGVKAGDANANDEAASADANDIGYAKLVGSDLFTLTKDAGSDGEQSTLFKLLVSAPASGLVDTATNQAIVLSANAGGTEVLGK
VVDMAVKAGAALTLDETKGVKAGDANANDEAASADANDIGYAKLVGSDLFTLTKDAGSDGEQSTLFKLLVSAPASGLVDTATNQAIVLSANAGGTEVLGK
VVDMAVKAGAALTLDETKGVKAGDANANDEAASADANDIGYAKLVGSDLFTLTKDAGSDGEQSTLFKLLVSAPASGLVDTATNQAIVLSANAGGTEVLGK
VVDMAVKAGAALTLDETKGVKAGDANANDEAASADANDIGYAKLVGSDLFTLTKDAGSDGXQSTLFKLLVXAPASGLVDTATXQAIVLSANAGGTEVLGK
VVDMAVKAGAALTLDETXGXKAGDANXNDEAASADANDIGYAKLVGSDLFTLTKDAGSDGXQSTLFKLLVXAPASGLVDTATXQAIVLSANAGGTEVLGK
VVDMAVKAGAALTLDETXGXKAGDANXNDEAASADANDIGYAKLVGSDLFTLTKDAGSDGXQSTLFKLLVXAPASGLVDTATXQAIVLSANAGGTEVLGK
VVDMAVKAGAALTLDETXGXKAGDANXNDEAASADANDIGYAKLVGSDLFTLTKDAGSDGXQSTLFKLLVXAPASGLVDTATXQAIVLSANAGGTEVLGK
VVDMAVKAGAALTLDETXGXKAGDANXNDEAASADANDIGYAKLVGSDLFTLTKDAGSDGXQSTLFKLLVXAPASGLVDTATXQAIVLSANAGGTEVLGK
VVDMAVKAGAALTLDETXGXKAGDANXNDEAASADANDIGYAKLVGSDLFTLTKDAGSDGXQSTLFKLLVXAPASGLVDTATXQAIVLSANAGGTEVLGK
VVDMAVKAGAALTLDETXGXKAGDANXNDEAASADANDIGYAKLVGSDLFTLTKDAGSDGEQSTLFKLLVGAPASGLVDTATNQAIVLSANAGGTEVLGK
VVDMAVKAGAALTLDETKGVKAGDANANDEAASADANDIGYAKLVGSDLFTLTKDAGSDGEQSTLFKLLVSAPASGLVDTATNQAIVLSANAGGTEVLGK
VVDMAVKAGAALTLDETXGXKAGDANXNDEAASADANDIGYAKLVGSDLFTLTKDAGSDGXQSTLFKLLVXAPASGLVDTATXQAIVLSANAGGTEVLGK
VVDMAVKAGAALTLDETKGVKAGDANANDEAASADANDIGYAKLVGSDLFTLTKDAGSDGEQSTLFKLLVSAPASGLVDTATNQAIVLSANAGGTEVLGK
VVDMAVKAGAALTLDETKGVKAGDANANDEAASADANDIGYAKLVGSDLFTLTKDAGSDGEQSTLFKLLVSAPASGLVDTATNQAIVLSANAGGTEVLGK
VVDMAVKAGAALTLDETKGVKAGDANANDEAASADANDIGYAKLVGSDLFTLTKDAGSDGEQSTLFKLLVSAPASGLVDTATNQAIVLSANAGGTEVLGK
VVDMAVKAGAALTLDETKGVKAGDANGNDEAASADANDIGYAKLVGSDLFTLTKDAGSDGEQSTLFKLLVSAPASGLVDTATNQAIVLSANAGGTEVLGK
VVDMAVKAGAALTLDETKGVKAGDANANDEAASADANDIGYAKLVGSDLFTLTKDAGSDGEQSTLFKLLVSAPASGLVDTATNQAIVLSANAGGTEVLGK
VVDMAVKAGAALTLDETKGVKAGDANANDEAASADANDIGYAKLVGSDLFTLTKDAGSDGEQSTLFKLLVGAPASGLVDTATNQAIVLSANAGGTEVLGK
VVDMAVKAGAALTLDETKGVKAGDANANDEAASADANDIGYAKLVGSDLFTLTKDAGSDGEQSTLFKLLVGAPASGLVDTATNQAIVLSANAGGTEVLGK
VVDMAVKAGAALTLDETKGVKAGDANANDEAASADANDIGYAKLVGSDLFTLTKDAGSDGEQSTLFKLLVSAPASGLVDTATNQAIVLSANAGGTEVLGK
VVDMAVKAGAALTLDETKGVKAGDANANDEAASADANDIGYAKLVGSDLFTLTKDAGSDGEQSTLFKLLVSAPASGLVDTATNQAIVLSANAGGTEVLGK
VVDMAVKAGAALTLDETKGVKAGDANANDEAASADANDIGYAKLVGSDLFTLTKDAGSDGEQSTLFKLLVSAPASGLVDTATNQAIVLSANAGGTEVLGK
VVDMAVKAGAALTLDETQGMKAGDANANDEAASADANDIGYAKLVGSDLFTLTKDAGSDGEQSTLFKLLVSAPASGLVDTATNQAIVLSANAGGTEVLGK
VVDMAVKAGAALTLDETQGMKAGDANANDEAASADANDIGYAKLVGSDLFTLTKDAGSDGEQSTLFKLLVSAPASGLVDTATNQAIVLSANAGGTEVLGK
VVDMAVKAGAALTLDETKGVKAGDANANDEAASADANDIGYAKLVGSDLFTLTKDAGSDGEQSTLFKLLVSAPASGLVDTATNQAIVLSANAGGTEVLGK

NTNGDVVFKVLLTASXGDVEVFQYRAIKHENASDHDESGAGGIIERIQAGSLKLEVTLTDKDGDSAKXXLDLGQMMRFEDDGP
NTNGDVVFKVLLTASNGDVEVFQYRAIKHENASDHDESGAGGIIERIQAGSLKLEVTLTDKDGDSAKDDLDLGQMMRFEDDGP
NTNGDVVFKVLLTASNGDVEVFQYRAIKHENASDHDESGAGGIIERIQAGSLKLEVTLTDKDGDSAKDDLDLGQMMRFEDDGP
NTNGDVVFKVLLTASNGDVEVFQYRAIKHENASDHDESGAGGIIERIQAGSLKLEVTLTDKDGDSAKDDLDLGQMMRFEDDGP
NTNGDVVFKVLLTASNGDVEVFQYRAIKHENASDHDESGAGGIIERIQAGSLKLEVTLTDKDGDSAKGELDLGQMMRFEDDGP
NTNGDVVFKVLLTASNGDVEVFQYRAIKHENASDHDESGAGGIIERIQAGSLKLEVTLTDKDGDSAKDDLDLGQMMRFEDDGP
NTNGDVVFKVLLTASNGDVEVFQYRAIKHENASDHDESGAGGIIERIQAGSLKLEVTLTDKDGDSAKDDLDLGQMMRFEDDGP
NTNGDVVFKVLLTASNGDVEVFQYRAIKHENASDHDESGAGGIIERIQAGSLKLEVTLTDKDGDSAKGELDLGQMMRFEDDGP
NTNGDVVFKVLLTASXGDVEVFQYRAIKHENASDHDESGAGGIIERIQAGSLKLEVTLTDKDGDSAKXXLDLGQMMRFEDDGP
NTNGDVVFKVLLTASXGDVEVFQYRAIKHENASDHDESGAGGIIERIQAGSLKLEVTLTDKDGDSAKXXLDLGQMMRFEDDGP
NTNGDVVFKVLLTASXGDVEVFQYRAIKHENASDHDESGAGGIIERIQAGSLKLEVTLTDKDGDSAKXXLDLGQMMRFEDDGP
NTNGDVVFKVLLTASXGDVEVFQYRAIKHENASDHDESGAGGIIERIQAGSLKLEVTLTDKDGDSAKXXLDLGQMMRFEDDGP
NTNGDVVFKVLLTASXGDVEVFQYRAIKHENASDHDESGAGGIIERIQAGSLKLEVTLTDKDGDSAKXXLDLGQMMRFEDDGP
NTNGDVVFKVLLTASXGDVEVFQYRAIKHENASDHDESGAGGIIERIQAGSLKLEVTLTDKDGDSAKXXLDLGQMMRFEDDGP

NTNGDVVFKVLLTASDGDVEVFQYRAIKHENASDHDESGAGGIIERIQAGSLKLEVTLTDKDGDSAKGELDLGQMMRFEDDGP
NTNGDVVFKVLLTASXGDVEVFQYRAIKHENASDHDESGAGGIIERIQAGSLKLEVTLTDKDGDSAKXXLDLGQMMRFEDDGP
NTNGDVVFKVLLTASXGDVEVFQYRAIKHENASDHDESGAGGIIERIQAGSLKLEVTLTDKDGDSAKDDLDLGQMMRFEDDGP
NTNGDVVFKVLLTASNGDVEVFQYRAIKHENASDHDESGAGGIIERIQAGSLKLEVTLTDKDGDSAKDDLDLGQMMRFEDDGP
NTNGDVVFKVLLTASNGDVEVFQYRAIKHENASDHDESGAGGIIERIQAGSLKLEVTLTDKDGDSAKDDLDLGQMMRFEDDGP
NTNGDVVFKVLLTASDGDVEVFQYRAIKHENASDHDESGAGGIIERIQAGSLKLEVTLTDKDGDSAKDDLDLGQMMRFEDDGP
NTNGDVVFKVLLTASDGDVEVFQYRAIKHENASDHDESGAGGIIERIQAGSLKLEVTLTDKDGDSAKDDLDLGQMMRFEDDGP
NTNGDVVFKVLLTASDGDVEVFQYRAIKHENASDHDESGAGGIIERIQAGSLKLEVTLTDKDGDSAKGELDLGQMMRFEDDGP
NTNGDVVFKVLLTASDGDVEVFQYRAIKHENASDHDESGAGGIIERIQAGSLKLEVTLTDKDGDSAKGELDLGQMMRFEDDGP
NTNGDVVFKVLLTASDGDVEVFQYRAIKHENASDHDESGAGGIIERIQAGSLKLEVTLTDKDGDSAKGELDLGQMMRFEDDGP
NTNGDVVFKVLLTASDGDVEVFQYRAIKHENASDHDESGAGGIIERIQAGSLKLEVTLTDKDGDSAKDDLDLGQMMRFEDDGP
NTNGDVVFKVLLTASDGDVEVFQYRAIKHENASDHDESGAGGIIERIQAGSLKLEVTLTDKDGDSAKDDLDLGQMMRFEDDGP
NTNGDVVFKVLLTASNGDVEVFQYRAIKHENASDHDESGAGGIIERIQAGSLKLEVTLTDKDGDSAKGELDLGQMMRFEDDGP
NTNGDVVFKVLLTASNGDVEVFQYRAIKHENASDHDESGAGGIIERIQAGSLKLEVTLTDKDGDSAKDDLDLGQMMRFEDDGP
NTNGDVVFKVLLTASNGDVEVFQYRAIKHENASDHDESGAGGIIERIQAGSLKLEVTLTDKDGDSAKGELDLGQMMRFEDDGP
NTNGDVVFKVLLTASNGDVEVFQYRAIKHENASDHDESGAGGIIERIQAGSLKLEVTLTDKDGDSAKDDLDLGQMMRFEDDGP

L8 repeat
VAGTISLVADEDNLPRGNNDTASGDAAQSNLTGTLPVNFGADGAGSIDFQGMHGLSAVIGNDNITYNWNASTNTLTAYQTGGALGVNDVFKIVVNPTTGQ
YTFTLLAAINHHAVADNTEGLVDPFVNLNYRVIDGDGDTAIGTLKVTIDDDIP

KAITPEEGFVTNQAGIVRTFDLDFDANIDNNVGADQLGTITFSGITNGQVVTGTVDGVPNQTLTSGGSAIHYYVSGNNVVEGWINGGPGDVGSTIVFRTT
LQPDMNYNASNDTYKFELFQPISTSTNVSIANFSGVNATSREFIYLENASGPGEDILFSAYIRNDNGTFTDATVNTNPDGIGVNNQNMNDRENLRVDFVR
NASTTGTNQNMTYEYDDHYLVNNFSFKIIQVTGNPPTGSLEVWVRAYNADDDDPTDNTASSANNLAHQDALRDDPQVALTQILVNGVPVTPTTVNASGGY
LISGLNLNDTITIRSANGYDRVEIENPRSGAHGVSNSSLNNETFDIGLFSYNTIKTTPSEININMGLSLTDSDGDKINSSIEINLAPSVFKVGENVDDTS
SSNVPHRVGGDTGVIDGSGGADILVGDVGGVEVVGTTARLAFILDESGSMSQNFGGTTRLEVLKQAMTDILTELSNTPNASITVHLVKFASVVNGTGTFE
ITGGGLQQALDFISGLQIQQGLLAGTNYEAALGQTVQWFSSQSGTVDVQQTLFFTDGVPTFYMDGNSTEYTNLARVYGNGSQTEEVLWENLFGEHAGGQA
TSDRINNLTESDSRDLDGLQSYSIDTNNDGIFEIQSVNSRSSGTTQTTNDLVRSVADTFNEVQALQAYGPLRAVSIADNANVYLQEIDSTGQPYLADSPE
VLQDILDELNPFNVLLAAGSDTIQANQEDDLIFGDVLFTDKLAEDEGLDLPKGSGWAVFEELEANHGWSRQDTLDYIRNHADELGRETVLSSGSKRSGGH
DIISGGQGDDRIYGQEGNDVIDAGSGDDVIDAGSGDDVIVGGTGNDTLTGGSGADQFVFFRGHGSNNAGTAPTDIITDFEVNIDKIVINANNIIGVSVSN
PVSNPDLSKTYTITVNYSDAPSIEHFKVTLSNGALLNDSGKTHLNGVVISGGTATIDGTIVGAVLYLDLNANNQEDEGERLGITNQYGHVEWVVDLSKFD
VNGDGQYVIGEARAVQTGGFDIDTGLSYEINLYGPVGSAVISPLTSLLQAQLEAGMDYETANAALVARLGLPEGTQIISFNPITGSGEVLAQNAGVMTAA
IQFAEIAAIHYSTDEGHVSFAVFEAISKALLELPEGVVADFGDEGFLQSIANYLGLDALVTSDMIDYMATSQKALEASILTLAPGEDALTAISKIQHVTQ
GIYAQAIEMALIGYLPLDALKNMSAVLKAYADGDITDEQLNSFEDKLSAVIVNWEDNNVTDASHQATLQTERTVDDSSVKSEANHEMAQEVNAVINDFMD
EHNISLYYYDQQAQMPQETQEEQQPVEAQAEETVIHDDVVQDLLLDDHSLTMLANNPELNGGNGNDVLHGTTGNDFIRGGQGNDTMTGGGGVDTFFWLSG
DDDGGVDTITDFKANPVDQSSDASVLNLSDLLSDADLETNSLDNYLNVSTTEEGDTAIKVDPNGNGNFDAPAQTIILEDVDLTAVFATNNSHDIVNQMIA
NGNLIVEQ

Leg-9 WP_080447442.1
MLVESVIGIVRAVNGLLEKVNAQGQASIVKSGARLQEGDVLTLLSGEAYIQFIHGFPEALALGKPVKLDGVSPTLQYGMEDLNEQLVQEALAKGIDPSVI
LDILGSAAAGAEAVGSGGDAFIMDPLFGFGHVTAGYPTGPISFAYEADTQQLFWFVPEETGVIAESELTQEPESIPQIPQFTTNQAVLTVFEDALPSGIP
DSAGQARIASSSLSSLLTSSPDVAASFAFNNNSSALPTLKSGGLDLSYELSADKRTLTLRESSNQGQGEEIMKFELTADGQLTQTLMNSIDHPTADSDDG
EWMRLDLSPVIDVTFRRISDGTVLESRTLPANAVVAGIQDDVPIARAQLTNNEILLDETVGVKVGDADAANDDFNPTTTADPFNNTYGRPIGLVQNANLL
DTSTSEMGGDYKKATMTHLLKITDAVSGLQTTNGTPINLFLESNGDITGRAGDTVAPALFAIRMNPNTGAISVAQYGPIKQFDTNSHDEAVDLTGRISAV
VTAKDSDGDVSNVEIPIGQLIIFEDDGP

L9 repeat
KADAKAGAVAGAVLDESPAEGGLNNPVTIAASAIAGFFETPDYGADGLGSVAYSLSGADGAGTGLWLTGQSGAANEIRLVKVSDTEYEGRVGGGAGTLAF
EVTINSGTGEVTVTQFATLEHTIDGSSAAAHDDALSLGGAAGIRVVQTVTDADGDTDAATSANALNITFEDDGP

L7 repeats
VVNMALKAGAALTLDETKGVKAGDANANDEAASADANDIGYAKLVGSDLFTLTKDAGSDGEQSTLFKLLVGAPASGLVDTATNQAIVLSANAGGTEVLGK
VVNMALKAGAALTLDETKGVKAGDANANDEAASADANDIGYAKLVGSDLFTLTKDAGSDGEQSTLFKLLVGAPASGLVDTATNQAIVLSANAGGTEVLGK

NTNGDVVFKVVLNGSNGDVEVFQYRAIKHENTSDHDESGAGGTIERIQSGSLKLEVTVTDKDGDSAKGELDLGQVMRFEDDGP
NTNGDVVFKVVLNGSNGDVEVFQYRAIKHENTSDHDESGAGGTIERIQAGSLKLEVTVTDKDGDSTKGELDLGQVMRFEDDGP

SIVLPEEGFVSNKAGVVKVFNLDLDSNIGNNVGADQPGSIRFINVVDGQQAQGIIDGELVNLTTVNGQLIKLYISNDGMIITGSTATSEAAVNSGNTVFK
VTMLPDNNYNTANDKYSVEMFGKIGVANTVNFSDLTGDSPAGNVNFKIISDQDFPGYEVMITPKDRVNPVDTSGGTVNSSSSDIGISNQWIDQGSRDSEI
LRLDYGQFAIINNNFDIEKHFTVNGASFEIHQIQGTPSNRASINVKAFDADDDNQLNDDPLDAITRIVIFTSSGGVETVIADVTSDASLGGMTFDFQGAN
GVNITGLDDGYRVRAYTNDGYNRLEVDNISSTPFSIGGLGIQSLIDTEKEVKMNYDVRLTDSDGDSVDSSLEINIVPSLFKVGENINDTSNSNVSHNVGG
DVGVIDGGLGADVLVGDVGGVDVSEKSYNLILILDTSGSMTAQIPFGDSGSIQRIQALKNSVNELIDHLSESNALNIRVHIVQFNTHASTVGTFNLIQNG
SVQATQVNNAHSGVNNLTAGGGTNYEAAFIQALNWVNNNGPLAASDLVNQVFFLSDGVPTYHYAGNGTTTLGGSGSSASFDTIFQPLGSSSTGGSSADTQ
SEVAQLEASFGQIQAVGINLNPNTIVYNPAGTANDLTAVQILDKMEGSNPSSLSDNISTAEQLTNVLADLSPINELKDVGGDVIAGNEEDDILFGDTLNT
DKLAVDSGLNLFDFPKGSGWAVFEELEANHGWSRQDTLDYITNHADELGMETVLSTGDTRSGGHDTISGGQGNDRIYGQEGNDVIDAGSGDDVIVGGTGN
DTLTGGSGADQFVFFRGHGSNNAGTAATDIITDFEVNIDKIVINANNIIGVSVSNPVSNPDLSKTYTITVNYSDAPSIEHFKVTLSNGALLNDSGKTHLN
GVVISGGTATIDGTIVGAVLYLDLNANNQEDEGERLGITNQYGHVEWVVDLSKFDVNGDGQYVIGEARAVQTGGFDVDTGLSYEINLYGPVGSAVISPLT
SLLQAQLETGMDYDTANAALVARLGLPEGTQIISFNPITGSGEVLAQNAGVMTAAIQFAEMAAIHYSTDESHVSFAVFEAISKALLELPEGVVADFGDEG
FLQSIANYLGLDALVTSDMIDYMATSQKALEASILTLAPGEDALTAISKIQHVTQGIYAQAIELALIGYLPLDALKNMSAVLKAYADGDITDEQLNSFED
KLSAVIVNWEENNVKDASHQAVSQTEHTADESLANSQADNELAQDVDTVINDFINEHNISLYYNDQQAQITQETREEQRPTEDQAEETVMHDDVVQDLLL
DDHSLTMLANNPELNGGNGNDVLHGTTGNDFIRGGQGNDTMTGGGGVDTFFWLSGDDDGGVDTITDFKANPVDQSSDASVLNLSDLLSDADLETNSLDNY
LNVSTTEEGDTAIKVDPNGNGNFDAPAQTIILEDVDLTAVFATNNSHDIVNQMIANGNLIVEQ

Leg-10 WP_052673671.1
MNRSLPTVDALNGLLIKNYPDGHLSIAKNGTQVMSGEQLFLLSGEAIVHNLGIGDAVLGVGHPFKVDGISPLLKNITTQAEVENLIEQAIANGVDPFPLL
ESLEATAAGESVPLGGGGSFFVLDPLYGAGQVNAGYDTRGIDFTVESTERDVPLFERRETTTTTQSAITEPEPPTPEPPAPEPISLTTTNLTFDETFGLQ
TGSASLLSNLSSSEVDSLTFSAPNATWDSASQTLTANDGSWSLKYNADTQNYEFTQLQAFQHADSTNPDDSMAIPVSVTLTRTDGAVATSTFNITMYDDGP

L10 repeats
RIETTSQVLNLTVDESNFTANDSANFSESFTVNAGVDGISSIKYALSVTAGSTGLVDALTNEAIVLSINEKGVVEGKTARGGELVFTLSVDSTGTVTLDQ
SINASAAADSLTVDETTLGNDASANFADNFTSSFGEDGAGQIDYSLSVHEGVTGLVDTATNEAVVLSINNDGVVEGRTANSNDLVFTVTVDSAGTVTLDQ
SINATAAADTLTVDETTLGNDASANFADNFTSSFGEDGAGQIDYSLSVHEGVTGLVDTATNEAVVLSINNDGVVEGRTANSNDLVFTVTVDSAGTVTLDQ
SINATAAADTLTVDETTLGNDASANFADNFTSSFGEDGAGQIDYSLSVHEGVTGLVDTATNEAVVLSINNDGVVEGRTANSNDLVFTVTVDSAGTVTLDQ
SINATTAADTLTVDETTLGNDASANFADNFTSSFGEDGAGQIDYSLSVHEGVTGLVDTATNEAVVLSINNDGVVEGRTANSNDLVFTVTVDSAGTVTLDQ
SINATAAADSLTVDETTLGNDASANFADNFTSSFGEDGAGQIDYSLSVHEGVTGLVDTATNEAVVLSINNDGVVEGRTATSNDLVFTVSVNGNGTVTLDQ
SINATAAADSLTVDETTLGNDASANFADNFTSSFGEDGAGQIDYSLSVHEGVTGLVDTATNEAVVLSINNDGVVEGRTATSNDLVFTVSVNGNGTVTLDQ
SINATAAADTLTVDETTLGNDASANFADNFTSSFGEDGAGQIDYSLSVHEGVTGLVDTATNEAVVLSINNDGVVEGRTANSNDLVFTVSVNGNGTVTLDQ
SINATAAADTLTVDETTLGNDASANFADNFTSSFGEDGAGQIDYSLSVHEGVTGLVDTATNEAVVLSINNDGVVEGRTANSNDLVFTVSVNGNGTVTLDQ
SINATTAADTLTVDETTLGNDASANFADNFTSSFGEDGAGQIDYSLSVHEGVTGLVDTATNEAVVLSMNNDGVVEGRTATSNDLVFTVSVNGNGTVTLDQ
SINATTAADTLTVDETTLGNDASANFADNFNLNYGADGAGNVAYSLGIKAGTTGLVDSATNQAVVLSLNN-GVVEGKT-TGGALVFTVSVDASGNVTLDQ
SINASAAADSLTVDETTLGNDASANFADNFNLNYGADGAGNVAYSLGIKAGTTGLVDSATNQAVVLSLNN-GVVEGKT-TGGALVFTVSVDASGNVTLDQ
TINASAATDSLTVDETTLGNDASANFADNFNLNYGADGAGNVAYSLGIKAGTTGLVDSATNQAVVLSLNN-GVVEGKT-TGGALVFTVSVDASGNVTLDQ
TINASAATDSLTVDETTLGNDASANFADNFNLNYGADGAGNVAYSLGIKAGTTGLVDSATNQAVVLSLNN-GVVEGKT-TGGALVFTVSVDASGNVTLDQ
TINASAATDSLTVDETTLGNDASANFADNFNLNYGADGAGNVAYSLGIKAGTTGLVDSATNQAVVLSLNN-GVVEGKT-TGGALVFKVSVDASGNVTLDQ
TINASAAADTLTVDETTLANNASANFADNFNLNYGADGAGNVSYSLGIKAGTTGLVDSATNQAVVLSLNN-GVVEGKT-TGGALVFKVSVDASGNVTLDQ

LRAVLHSPNTGSDQSATLNSPDLIQLTAAITDNDGDTALASLNIGQALNFKDDGP
LRAVVHSPNTGADQPTSLNAADLIQLTATITDKDGDSASAHLDLGQALNFKDDGP
LRAVVHSPNTGADQPTSLNAADLIQLTATITDKDGDSASAHLDLGQALNFKDDGP
LRAVVHSPNTGADQPTSLNAADLIQLTATITDKDGDSASAHLDLGQALNFKDDGP
LRAVVHSPNTGADQPTSLNAADLIQLTATITDKDGDSASAHLDLGQALNFKDDGP
LRAVVHSPNTGADQPTSLNAADLIQLTATITDKDGDSASAHLDLGQALNFKDDGP
LRAVVHSPNTGADQPTSLNAADLIQLTATITDKDGDSASAHLDLGQALNFKDDGP
LRAVVHSPNTGADQPTSLNAADLIQLTATITDKDGDSASAHLDLGQALNFKDDGP
LRAVVHSPNTGADQPTSLNAADLIQLTATITDKDGDSASAHLDLGQALNFKDDGP
LRAVVHSPNTGADQPISLNAADLIQLTATITDKDGDSASAHLDLGQALNFKDDGP
LRAVIHSPNTGPDQTTSLNTADLIRLTATITDKDGDSSSASIDIGQALNFKDDGP
LRAVIHSPNTGPDQTTSLNAADLIRLTATITDKDGDSSSASIDIGQALNFKDDGP
LRAVIHSPNTGPDQTTSLNTADLIRLTATITDKDGDSSSASIDIGQALNFKDDGP
LRAVIHSPNTGPDQTTSLNAADLIRLTATITDKDGDSSSASIDIGQALNFKDDGP
LRAVIHSPNTGPDQTTSLNAADLIRLTATITDKDGDSSSASIDIGQALNFKDDGP
LRAVIHSPNTGPDQTTSLNAADLIRLTATITDKDGDSSSASINLGNAINFKDDAP

L8 repeat
TAGTLTITADEDDLSVGNKDIVAGDDAQTNLTGTLAHNFGADGVGKIDFAAMNGQTAVVGNETVKYSWNSSNSTLTAYRAGGALGVDDVFKITVNPSTGQ
YTFVLLAAISHHAVADNNETTNASLTLNYTVTDADNDTATGAMTIVIDDDVP

TVITPEHASVGNAAGSTGTFSLDIDSNIHNNIGGDQPGTISFSGIVNGQQATGVINGSEVPLSSGGFPIRLYLVDHDSNVNTPDVVEGWIKGAPGSYGAE
EVFRVTLQPDGSYNLSNDTYKVDVFAPIETATSTTLTNLNFSTAGNKAFNYIDVANTSEDLLFSGFVRSSSGSTTTGTVNTNATAIGINNQSMNDGDNLR
IDFVNSASVTNSSNNSYNYVDHFDINNFSFNIVQVGGNTQPNSIEVWVRIYNADNDDPAGSNTSTHSQQLADDPQKVITSIQVNNVEINLSSLQSDGNGG
YLIRGLNLNDKITVSGDESYNRIEIENAISKTHGVNDSSLNGDSFDIGIFSYNKIVTDIPDVQMDFDVVIKDADGDTSSANLEVNLAQPVCIVGTNVSDT
AAANTPYEVGTGTGVITGDGGNDVLVGDTGGVDVTPKSINLVLILDTSGSMADQISFDGSTISRMQALKNAVNNLLNQLTDSDAANVRVNIIQFNTTATT
VGTYNLIVNGDPQDSSNEALGQAHSAINNLVAGGGTNYEAAFNQALAWVDSGGALSADNLINQVIFISDGAPTYYLNSSGAVAGTGNTTTQQTVFDPLGV
TDSSNEVALLEQKFGPIDAIGINVNETTPVYNPSGTANDLTAKQILDKMEGSNPSSSSNNIHSAEELTQVLSDLSPLKQLNTTGDDTIIGGNGEDILYGD
SLYTDHLAALQGLNLFDFANGSGWDVFNELESNSQYNWSREDTLNYINQHSHELAQESLLANNTGRGGGNDYIDAGAGNDRIYGQEGNDTILAGKGADIV
DGGSGNNIIDLGADSDQDTLLLNVETLKGHNTIHNFNPQQDILKFEGVLNMDDVGDLDLLDQNNPFTTSNNGQDLNVNFANGAQLTFVGLAANVDVGGLT
SIQDLVQQPVVVVNELGS

Leg-11 WP_095141722.1
MNKPLPVVDALNGLLIKNHANGQVSIAKNGTQLMAGDQVVLLSGEALLRHVTLGDAALGLERPFKIDGISPLLKNITSQADAENLIEHAIANGIDPVAVL
EALEPTAAGEEVLLGEGGNAVRIDPLYGVGSVSAGYETIGPELIFRSAERYIPLFEEEDEIASVTESLATEESPESSSPEPSSPEPLQLSVVDLAYDETD
GLALGSAAIQTNAASEDINAITFSASDATWDAASQTLRADDSSWELRFDPNTGQYEFQQLRAWTHPDTTDHDDAMSVRITVTLTRTDGAQATRDFTVRVD
DDGP

L6 repeat
RIVVENVPTPDLSVDETVLANDATGDFRFLFNAQFGTDDAAANNPLTYSLAIKSTGVDSGLVDTATQQKIYLFLENGVVVGHVGSKEGAEAFRISLDNDG
KITLDQQRALVHPDATTPDDVVHLAAADLITLEAVATDSDGDSSRAVANIGLAFNFADDGP

L2 repeats
SVVVNANAEAGVLLTTQDADTIGAATDTATTTANFSGVFSNTPTYGADGAGSTVMSYSLALAGAPGTDSGLDSDGLSIYLYDIAGVIVGSTAAALAGVDA
SVVVNANAEAGVLLTTQDADTIGAATDTATTTANFSGVFSNTPTYGADGAGSTVMSYSLALAGAPGTDSGLDSDGLSIYLYDIAGVIVGSTAAALAGVDA
SVVVNANAEAGVLLTTQDADTIGAATDTATTTANFSGVFSNTPTYGADGAGSTVMSYSLALAGAPGTDSGLDSDGLSIYLYDIAGVIVGSTAAALAGVDA
SVVVNANAEAGVLLTTQDADTIGAATDTATTTANFSGVFSNTPTYGADGAGSTVMSYSLALAGAPGTDSGLDSDGLSIYLYDIAGVIVGSTAAALAGVDA
SVVVNANAEAGVLLTTQDADTIGAATDTATTTANFSGVFSNTPTYGADGAGSTVMSYSLALAGAPGTDSGLDSDGLSIYLYDIAGVIVGSTAAALAGVDA
SVVVNANAEAGVLLTTQDADTIGAATDTATTTANFSGVFSNTPTYGADGAGSTVMSYSLALAGAPGTDSGLDSDGLSIYLYDIAGVIVGSTAAALAGVDA
SVVVNANAEAGVLLTTQDADTIGAATDTATTTANFSGVFSNTPTYGADGAGSTVMSYSLALAGAPGTDSGLDSDGLSIYLYDIAGVIVGSTAAALAGVDA
SVVVNANAEAGVLLTTQDADTIGAATDTATTTANFSGVFSNTPTYGADGAGSTVMSYSLALAGAPGTDSGLDSDGLSIYLYDIAGVIVGSTAAALAGVDA
SVVVNANAEAGVLLTTQDADTIGAATDTATTTANFSGVFSNTPTYGADGAGSTVMSYSLALAGAPGTDSGLDSDGLSIYLYDIAGVIVGSTAAALAGVDA
SVVVNANAEAGVLLTTQDADTIGAATDTATTTANFSGVFSNTPTYGADGAGSTVMSYSLALAGAPGTDSGLDSDGLSIYLYDIAGVIVGSTAAALAGVDA
SVVVNANAEAGVLLTTQDADTIGAATDTATTTANFSGVFSNTPTYGADGAGSTVMSYSLALAGAPGTDSGLDSDGLSIYLYDIAGVIVGSTAAALAGVDA
SVVVNANAEAGVLLTTQDADTIGAATDTATTTANFSGVFSNTPTYGADGAGSTVMSYSLALAGAPGTDSGLDSDGLSIYLYDIAGVIVGSTAAALAGVDA
SVVVNANAEAGVLLTTQDADTIGAATDTATTTANFSGVFSNTPTYGADGAGSTVMSYSLALAGAPGTDSGLDSDGLSIYLYDIAGVIVGSTAAALAGVDA
SVVVNANAEAGVLLTTQDADTIGAATDTATTTANFSGVFSNTPTYGADGAGSTVMSYSLALAGAPGTDSGLDSDGLSIYLYDIAGVIVGSTAAALAGVDA
SVVVNANAEAGVLLTTQDADTIGAATDTATTTANFSGVFSNTPTYGADGAGSTVMSYSLALAGAPGTDSGLDSDGLSIYLYDIAGVIVGSTAAALAGVDA

SNTIFDLSVDASGVVTLNQYEEIDHPIANDPSPTGSPFADQFAVLSNGLVTLTASALTTDGDGDTATDSETIDLG-NVRFADDGP

SNTIFDLSVNASGVVTLNQYEEIDHPIANDPSPTGSPFADQFAVLGNGLVTLTASALTTDGDGDTATDSETIDLGGNVRFADDGP
SNTIFDLSVDASGVVTLNQYEEIDHPIANDPSPTGSPFADQFAVLGNGLVTLTASALTTDGDGDTATDSETIDLGGNVRFADDGP
SNTIFDLSVDASGVVTLNQYEEIDHPIANDPNPTGSPFADQFAVLSNGLVTLTASALTTDGDGDTATDSETIDLGGNVRFADDGP
SNTIFDLSVDASGVVTLNQYEEIDHPIANDPSPTGSPFADQFAVLSNGLVTLTASALTTDGDGDTATDSETIDLGGNVRFADDGP
SNTIFDLSVDASGVVTLNQYEEIDHPIANDPSPTGSPFADQFAVLGNGLVTLTASALTTDGDGDTATDSETIDLGGNVRFADDGP
SNTIFDLSVDASGVVTLKQYEEIDHPIANDPSPTGSPFADQFAVLSNGLVTLTASALTTDGDGDTATDSETIDLGGNVRFADDGP
SNTIFDLSVNASGVVTLNQYEEIDHPIANDPSPTGSPFADQFAVLGNGLVTLTASALTTDGDGDTATDSETIDLGGNVRFADDGP
SNTIFDLSVDASGVVTLNQYEEIDHPIANDPSPTGSPFADQFAVLSNGLVTLTASALTTDGDGDTATDSETIDLGGNVRFADDGP
SNTIFDLSVDASGVVTLNQYEEIDHPIANDPSPTGSPFADQFAVLGNGLVTLTASALTTDGDGDTATDSETIDLGGNVRFADDGP
SNTIFDLSVDASGVVTLKQYEEIDHPIANDPSPTGSPFADQFAVLSNGLVTLTASALTTDGDGDTATDSETIDLGGNVRFADDGP
SNTIFDLSVDASGVVTLNQYEEIDHPIANDPSPTGSPFADQFAVLSNGLVTLTASALTTDGDGDTATDSETIDLGGNVRFADDGP
SNTIFDLSVDASGVVTLNQYEEIDHPIANDPSPTGSPFADQFAVLSNGLVTLTASALTTDGDGDTATDSETIDLGGNVRFADDGP
SNTIFDLSVDASGVVTLNQYEEIDHPIANDPSPTGSPFADQFAVLGNGLVTLTASALTTDGDGDTATDSETIDLGGNVRFADDGP
SNTIFDLSVDASGVVTLKQYEEIDHPIANDPSPTGSPFADQFAVLGNGLVTLTASALTTDGDGDTATDSETIDLGGNVRFADDGP

L8 repeat
HAGTQSLTADEDNLAKGNNNIVTGDDAQSNLTGTLVHNFGADGAGKIDFVAMNGLTATLGAESIKYSWNASDNTLTAYRANGVLGTDDVFKITVNPVNGQ
YTFTLLAAVNHHEIADNNETTNASVNLNYTVTDGDGDTSIGSLTVVIDDDVP

TIIDPEGASLGNVAGSTTTFSLDTDGNIDNNVGADQLGSISFSSIVDGQQATGVIDGDVVNLSANGFPIHLYLVDHDSNVATPDTLQGWIKGAPGDVGAI
EVFRITLQPDGNYNLNNDTYKVEIFVPIGTSTTTTITNTSFATAGNKAFNFIDVSGTTEDLLFSGYVRATNGSLTQGTVNTNSTAVGVNNQSMNDGDNLR
IDFVNSPSVTNSASNSYNYTDHFDINNFNFKIVQVNGNPPPGSIEIWVRTYDANDDDPSGTSTSVHNAQLADDPQTTISTIKVNGVSLDLDDLQSDGSGG
YLIRGLSLNDIITVGTDEGYNRIEIENARSGAHGINDSTLNGEAFDIGLFSYETIVTNVPNVNMNFEVALQDADGDTSLANLTVNLAEPVFKVGTNTSDS
TSSPATYEVGSGTGTITGNGGNDVLVGDVGGIDATLKSINLLLILDTSGSMSNQIPFGGSTISRMQALKNAVSNLLNELAESQTLNVRVHIVQFNTNAAA
VGTFNLVVNGAMVDTALEQSLIAINGLSANGGTNYEAAFQNAYNWANGGGPLTGDQNLINQTIFISDGAPTYYLNSNGTVGGTGSTTTERTVYDPLGATA
QDNVNEVALLEQTFGPIEAIGINVSETAIVYNPSGTANDLTAKQILDKMEGSNPSSLSNNIDSAEELNQVLSDLSPLNQLNTVGDDRIVGDYGDDILYGD
ALNTDELATLMGLDLFDFPEGSGWSVFDELENNSQYNWTREDTLDYINNHSHELAQETLLANGSGRAGGNDYIDSGAGNDRIYGQEGNDEILAGRGADVV
DPGTGNNIIDFGIDTDSDTLLLNLETFKGHNTIKHFDPQQDVLKFESVLGVETVDDLDELLDQNNPFSKSGDGQDLNVNFASGAQLTFEGLGPSIPDVQG
LQSLQDIVQQPIVVVNELGG

Leg-12 STX42054.1
MRNYFSINSEIKNNSLIENPSTFSNGMTITFAGNMSIKSSSLLNLLSNPDYTKFINGFSSILDLHRPFLTPPAATYQVMLENAMNEAITLDIDPTQILDT
LQATSAGKSQEVLSDGSQFFIIDPLYGQGNVGAGYPTDSFTLSTNDTTQKAILYTTNEKTTTISNNVPTTHTDINSITKSNLSTVTANGNVGNDAAGIKT
ETDLASPMTSDVNTTIVGTYGTLTLQADGSYTYQANPDTDDDTIDQFLYTMKDVDGDYSDTSAKKLHNRTGMSTTCPFLIPWLSSSTLTIKYTDSGPIAP
ADNDVQVEERTIDLSQVGVDVQNLSLATVLGSLPASAAGDQLNATGSGSYSYPLVNNAGSYETLGTNADDTNVNPLDNPDTSTSPNDDANVDQSSDSIFY
QATDTNDNTTGTTTTVDDVP

L11 repeats

SAHPDINSITEGTPLTVTAEEGVLKNDEAGADGFAAGGSVVGVRAAGSDTTSAVSTGVNTAINGTYGTLIVQANGSYTYQANPNTDGQSDVFVYTIKDGD
TARTDVASVTEGNLLTVTAAGGVLHNDTAGADNYAAGGSVVGVRAAGSDTTSAVSTGVNTAINGTYGTLIVQANGSYTYQANPNTDGQSDVFVYTIKDGD
TARTDVASVTEGNLLTVTAAGGVLHNDTAGADNYAAGGSVVGVRAAGSDTTSAVSTGVNTAINGTYGTLIVQANGSYTYQANPNTDGQSDVFVYTIKDGD
TARTDVASVTEGNLLTVTAAGGVLHNDTAGADNYAAGGSVVGVRAAGSDTTSAVSTGVNTAINGTYGTLIVQANGSYTYQANPNTDGQSDVFVYTIKDGD
TARTDVASITEGNLLTVTAAGGVLHNDTAGADNYAAGGSVVGVRAAGSDTTSAVSTGVNTAINGTYGTLIVQANGSYTYQANPNTDGQSDVFVYTIKDGD
TARTDVASITEGNLLTVTAAGGVLHNDTAGADNYAAGGSVVGVRAAGSDTTSAVSTGVNTAINGTYGTLIVQANGSYTYQANPNTDGQSDVFVYTIKDGD
TARTDVASVTEGNLLTVTAAGGVLHNDTAGADNYAAGGSVVGVRAAGSDTTSAVSTGVNTAINGTYGTLTLQANGSYTYQANPNTDGQSDVFVYTIKDGD
TARTDVASVTEGNLLTVTAAGGVLHNDTAGADNYAAGGSVVGVRAAGSDTTSAVSTGVNTAINGTYGTLTLQANGSYTYQANPNTDGQSDVFVYTIKDGD
TARTDVASITEGNLLTVTAAGGVLHNDTAGADNYAAGGSVVGVRAAGSDTTSAVSTGVNTAINGTYGTLIVQANGSYTYQANPNTDGQSDVFVYTIKDGD
TARTDVASITEGNLLTVTAAGGVLHNDTAGADNYAAGGSVVGVRAAGSDTTSAVSTGVNTAINGTYGTLIVQANGSYTYQANPNTDGQSDVFVYTIKDGD
TARTDVASITEGNLLTVTAAGGVLHNDTAGADNYAAGGSVVGVRAAGSDTTSAVSTGVNTAINGTYGTLTLQANGSYTYQANPNTDGQSDVFVYTIKDGD
TARTDVASVTEGNLLTVTAAGGVLHNDTAGADNYAAGGSVVGVRAAGSDTTSAVSTGVNTAINGTYGTLTLQANGSYTYQANPNTDGQSDVFVYTIKDGD
TARTDVASVTEGNLLTVTAAGGVLHNDTAGADNYAAGGSVVGVRAAGSDTTSAVSTGVNTAINGTYGTLIVQANGSYTYQANPNTDGQSDVFVYTIKDGD

GDLSTTTLTINLTDSGLSAPADNDVLVEERALDPAQDGADLAAGTVTGSLPNSTAETDASNQLNATGSTGPYTYSLVGAANGTYGVIQINADGTYTYTLT
GDLSTTTLTINLTDSGLSAPADNDVLVEERALDPAQDGADLAAGTVTGSLPNSTAETDASNQLNATGSTGPYTYSLVGAANGTYGVIQINADGTYTYTLT
GDLSTTTLTINLTDSGLSAPADNDVLVEERALDPAQDGADLAAGTVTGSLPNSTAETDASNQLNATGSTGPYTYSLVGAANGTYGVIQINADGTYTYTLT
GDLSTTTLTINLTDSGLSAPADNDVLVEERALDPAQDGADLAAGTVTGSLPNSTAETDASNQLNATGSTGPYTYSLVGAANGTYGVIQINADGTYTYTLT
GDLSTTTLTINLTDSGLSAPADNDVLVEERALDPAQDGADLAAGTVTGSLPNSTAETDASNQLNATGSTGPYTYSLVGAANGTYGVIQINADGTYTYTLT
GDLSTTTLTINLTDSGLSAPADNDVLVEERALDPAQDGADLAAGTVTGSLPNSTAETDASNQLNATGSTGPYTYSLVGAANGTYGVIQINADGTYTYTLT
GDLSTTTLTINLTDSGLSAPADNDVLVEERALDPAQDGADLAAGTVTGSLPNSTAETDASNQLNATGSTGPYTYSLVGAANGTYGVIQINADGTYTYTLT
GDLSTTTLTINLTDSGLSAPADNDVLVEERALDPAQDGADLAAGTVTGSLPNSTAETDASNQLNATGSTGPYTYSLVGAANGTYGVIQINADGTYTYTLT
GDLSTTTLTINLTDSGLSAPADNDVLVEERALDPAQDGADLAAGTVTGSLPNSTAETDASNQLNATGSTGPYTYSLVGAANGTYGVIQINADGTYTYTLT
GDLSTTTLTINLTDSGLSAPADNDVLVEERALDPAQDGADLAAGTVTGSLPNSTAETDASNQLNATGSTGPYTYSLVGAANGTYGVIQINADGTYTYTLT
GDLSTTTLTINLTDSGLSAPADNDVLVEERALDPAQDGADLAAGTVTGSLPNSTAETDASNQLNATGSTGPYTYSLVGAANGTYGVIQINADGTYTYTLT
GDLSTTTLTINLTDSGLSAPADNDVLVEERALDPAQDGADLAAGTVTGSLPNSTAETDASNQLNATGSTGPYTYSLVGAANGTYGVIQINADGTYTYTLT
GDLSTTTLTINLTDSGLSAPADNDVLVEERALDPAQDGADLAAGTVTGSLPNSTAETDASNQLNATGSTGPYTYSLVGAANGTYGVIQINADGTYTYTLT

KAYTTAPDANNGANTEDNRDSFTYQVTDAKGNTTTGTITVDIVDDVP
KAYTTAPDANNGANTEDNRDSFTYQVTDAKGNTTTGTITVDIVDDVP
KAYTTAPDANNGANTEDNRDSFTYQVTDAKGNTTTGTITVDIIDDVP
KAYTTAPDANNGANTEDNRDSFTYQVTDAKGNTTTGTITVDIVDDVP
KAYTTAPDANNGANTEDNRDSFTYQVTDAKGNTTTGTITVDIVDDVP
KAYTTAPDANNGANTEDNRDSFTYQVTDAKGNTTTGTITVDIIDDVP
KAYTTAPDANNGANTEDNRDSFTYQVTDAKGNTTTGTITVDIIDDVP
KAYTTAPDANNGANTEDNRDSFTYQVTDAKGNTTTGTITVDIVDDVP
KAYTTAPDANNGANTEDNRDSFTYQVTDAKGNTTTGTITVDIVDDVP
KAYTTAPDANNGANTEDNRDSFTYQVTDAKGNTTTGTITVDIVDDVP
KAYTTAPDANNGANTEDNRDSFTYQVTDAKGNTTTGTITVDIIDDVP
KAYTTAPDANNGANTEDNRDSFTYQVTDAKGNTTTGTITVDIIDDVP
KAYTTAPDANNGANTEDNRDSFTYQVTDAKGNTTTGTITVDIVDDVP

VVSNITRSSQATEGSIDTNLMLILDTSGSMSGERMEALKNSTLELLEQHEALGDVKVRIVTFDSTATAIGSGWMTVDEAKTEILNLTAGGNTNYDAALLT
ARTAFNSGTVGAADGRIADAQNKSYFISDGDPTVSQDWPDIAGTSNQVGIQPSEQTAWQNFLKDTNGAATGGEQITSYALGIGTGVELENLEPIAYDGVT
STTIEPQLIEDVSQFSDALVLTMTSAPVTGNLITDPNPDGSFGADGGHIQFITVNGMTYTYNTNTNTIVTSGSATVGTNTHSFDATTHQLTVAIENSNGE
KITVDMDDGAYTFNPPQNLTSPINRPIGFSVIDNDGDTASATISFNIDLPGRSPILIVRDDLVLTNQAAASDVINIPTWALLENDSGGIGVSSITAVSNT
SPSNEGTVSLVGGTTSAVAFTEEGSSAQDGTYNGSTANPFGYTNTTGLNSDTAHVDLNRAQANQNDLDGTFRNEILLGRDGSADRIYGDTGDDILIGLGG
NDELRGEEGDDILAGGAGNDTLIGGSGNDTATYIDATGGVTVSLATTNNQITGQGTDKLSGIENLTGSTFSDTLTGNSSANIIQGLSGNDSINASGGNDT
VIGGQGADTLTGGSGADTFVWKYGDDAGNPTDTITDFNQSQGDKIDLSNFLEGNVTPANLSSYLNVSLINGGNDTQITIDADGTGPKTAQQTIILTGVNN
LQTSSIITPTSVAAPIVFDLNNDGFNFTAHSNNSVLFDYNNGSLQMTAWVSGSDAFLAYDANNDQMINDGSEIVFSDPTQNAFTDLQGLKINYDTNFDNK
LDSNDAEFSKFGLWQDSNQNGISDPGEFKSLSDYGITSIDLSGYGEGFTAANGEVQVYSLGSFTYQDGSTGTFADVGLSLFDTPTQDTASLAEQSDLSDH
EHSLDPHHVPEQGLLGEYTHLLEDPSEMIQLGKLSEIAGMPELKLGGHMIELTISEIKQLFAEAMEQTDTTSSHSKNILDLDFKAINALTENNELNLQLA
EQGGVNVLKIHAASSDTINLENATDITKHVPQGQIGAGEQVFQIHNSDTSQSAYVHIVGTPLVHVEAPHTPPPAAHE

Aer-1 WP_167418739.1
MRTQIIDKTVVVSSVEGNVQILLADGSSRPLQPGEILQPGARLNIADDAKLMLAPYDDKPAADNGVPAGAEMPPEGQSSSSVANHQQGSPEVSPEIAA
LQQSILQGVDPTKNFEASAAGGAPAAGGGGGIGGVAGASGNGGFVVIDRIGDATIAEAGFDTGYDTLIDQNVLQEDDLLPENQLDDQDESAVTQEDQAIS
GNVLDNSNNPDGPLDSSVISYSWGSNQGIAAGVESTLDGIGTLIINADGSYTFTPALNYTGPVPAVNYTVTDGDDTNDSTLTITITPVDEPVSLNGLQVE
GGELVLDEAALPDGSAPDGAALTKSSTFTFNAADGVQSLTLGGVTLISNGQVLTTFPQGIPSPLGNQLLVTGVSYNPVTGVGSVTYSYTLNDNETHNKPA
NDGALTESFNVVLTDLDGDTTSAGLDVVVLDDVP

A1 repeats
TAVSDVNLTAASENNLVLNGNVITNDVQGADGASVTAGTLSGTYGSLVLNANGTYTYTLNPNDGDFKALTGGGVGSEVFTYTLTDADGDVSTATLTLTIK
TAVSDVNLTVASENNLVLNGNVITNDVQGADGAAVTAGTLSGTYGSLVLNANGTYTYTLNPNDGDFKALTGGGVGSEVFTYTLTDADGDVSTATLTLTIK
TAVSDVNLTVASENNLVLNGNVITNDVQGADGAAVTAGTLSGTYGSLVLNANGTYTYTLNPNDGDFKALTGGGVGSEVFTYTLTDADGDVSTATLTLTIK
TAVSDVNLTVASENNLVLNGNVITNDVQGADGAAVTAGTLSGTYGSLVLNANGTYTYTLNPNDGDFKALTGGGVGSEVFTYTLTDADGDVSTATLTLTIK
TAVSDVNLTVASENNLVLNGNVITNDVQGADGAAVTAGTLSGTYGSLVLNANGTYTYTLNPNDGDFKALTGGGVGSEVFTYTLTDADGDVSTATLTLTIK
TAVSDVNLTVASENNLVLNGNVITNDVQGADGAAVTAGTLSGTYGSLVLNANGTYTYTLNPNDGDFKALTGGGVGSEVFTYTLTDADGDVSTATLTLTIK
TAVSDVNLTVASENNLVLNGNVITNDVQGADGAAVTAGTLSGTYGSLVLNANGTYTYTLNPNDGDFKALTGGGVGSEVFTYTLTDADGDVSTATLTLTIK
TAVSDVNLTVASENNLVLNGNVITNDVQGADGAAVTAGTLSGTYGSLVLNANGTYTYTLNPNDGDFKALTGGGVGSEVFTYTLTDADGDVSTATLTLTIK
TAVSDVNLTVASENNLVLNGNVITNDVQGADGAAVTAGTLSGTYGSLVLNANGTYTYTLNPNDGDFKALTGGGVGSEVFTYTLTDADGDVSTATLTLTIK
TAVSDVNLTVASENNLVLNGNVITNDVQGADGAAVTAGTLSGTYGSLVLNANGTYTYTLNPNDGDFKALTGGGVGSEVFTYTLTDADGDVSTATLTLTIK
TAVSDVNLTVASENNLVLNGNVITNDVQGADGAAVTAGTLSGTYGSLVLNANGTYTYTLNPNDGDFKALTGGGVGSEVFTYTLTDADGDVSTATLTLTIK

NDDDGVTITNLTPKGEGGDAKVYEDDLLATRGENESAGSDSSKESTTVTGDFTISAPDGVKTLSVGGINIVVNGVVAGFPLSGVTTPLGNTLTITGYNPA
NDDDGVTITNLTPKGEGGDAKVYEDDLLATRGENESAGSDSSKESTTVTGDFTISAPDGVKTLSVGGINIVVNGVVAGFPLSGVTTPLGNTLTITGYNPA
NDDDGVTITNLTPKGEGGDAKVYEDDLLATRGENESAGSDSSKESTTVTGDFTISAPDGVKTLSVGGINIVVNGVVAGFPLSGVTTPLGNTLTITGYNPA
NDDDGVTITNLTPKGEGGDAKVYEDDLLATRGENESAGSDSSKESTTVTGDFTISAPDGVKTLSVGGINIVVNGVVAGFPLSGVTTPLGNTLTITGYNPA
NDDDGVTITNLTPKGEGGDAKVYEDDLLATRGENESAGSDSSKESTTVTGDFTISAPDGVKTLSVGGINIVVNGVVAGFPLSGVTTPLGNTLTITGYNPA
NDDDGVTITNLTPKGEGGDAKVYEDDLLATRGENESAGSDSSKESTTVTGDFTISAPDGVKTLSVGGINIVVNGVVAGFPLSGVTTPLGNTLTITGYNPA
NDDDGVTITNLTPKGEGGDAKVYEDDLLATRGENESAGSDSSKESTTVTGDFTISAPDGVKTLSVGGINIVVNGVVAGFPLSGVTTPLGNTLTITGYNPA
NDDDGVTITNLTPKGEGGDAKVYEDDLLATRGENESAGSDSSKESTTVTGDFTISAPDGVKTLSVGGINIVVNGVVAGFPLSGVTTPLGNTLTITGYNPA
NDDDGVTITNLTPKGEGGDAKVYEDDLLATRGENESAGSDSSKESTTVTGDFTISAPDGVKTLSVGGINIVVNGVVAGFPLSGVTTPLGNTLTITGYNPA
NDDDGVTITNLTPKGEGGDAKVYEDDLLATRGENESAGSDSSKESTTVTGDFTISAPDGVKTLSVGGINIVVNGVVAGFPLSGVTTPLGNTLTITGYNPA
NDDDGVTITNLTPKGEGGDAKVYEDDLLATRGENESAGSDSSKESTTVTGDFTISAPDGVKTLSVGGINIVVNGVVAGFPLSGVTTPLGNTLTITGYNPA

TGLVSYSYTLNDNEAHLTANGNNSLFEDFAVSLVDSDGDSASNTLSVQIVDDVP
TGLVSYSYTLNDNEAHPTANGNNSLFEDFAVSLVDSDGDSASNTLSVQIVDDVP
TGLVSYSYTLNDNEAHPTANGNNSLFEDFAVSLVDSDGDSASNTLSVQIVDDVP
TGLVSYSYTLNDNEAHPTANGNNSLFEDFAVSLVDSDGDSASNTLSVQIVDDVP
TGLVSYSYTLNDNEAHPTANGNNSLFEDFAVSLVDSDGDSASNTLSVQIVDDVP
TGLVSYSYTLNDNEAHLTANGNNSLFEDFAVSLVDSDGDSASNTLSVQIVDDVP
TGLVSYSYTLNDNEAHPTANGNNSLFEDFAVSLVDSDGDSASNTLSVQIVDDVP
TGLVSYSYTLNDNEAHPTANGNNSLFEDFAVSLVDSDGDSASNTLSVQIVDDVP
TGLVSYSYTLNDNEAHPTANGNNSLFEDFAVSLVDSDGDSASNTLSVQIVDDVP
TGLVSYSYTLNDNEAHPTANGNNSLFEDFAVSLVDSDGDSASNTLSVQIVDDVP
TGLVSYSYTLNDNEAHPTANGNNSLFEDFAVSLVDSDGDSASNTLSVQIVDDVP

A2 repeat
TANDDADSATEGVFTPVTGNVITGAGTIGGTAGAGVDVMGADGAAISQVVSSNGNSDSNPAGGFTVTGLYGTLTMGLDGEYSYTLTAASVPVGASEVFTY
TLKDGDGDTDPATLTINIAQDTRVPVLTIGNATVDEEGLPEGTAASGNSEIFNGSFTINTQGENLTTLTIGGQTYNLTSGGSQTLINNTEGMLTVTGVSG
PTAGVYTVNYTYTLKDNVLTHNVQGNSDTANGPSFVVSATDASGDSGTGNLQVVISDDAP

IANAVTNAGQATQVQNTNLMLILDISGSMGDPSGYQGMTRMQVMQKSALELLDKYSAYGSVMVNIVTFSTSAANPTGVWVSVDVAKAIILGLTPTDSTNY
DDALNEAIKAFGDTGRLANAQNVSYFMSDGLPNASSLSGTPATVPDGNNSLGGGNGIDGDGTTLSGETKDWADFLTANNINSYALGMGSGATQSALDPIA
YNGVSGASPANTTAVVVTDFSQLTATLLSTVVAPPLAGQLINGVAASAGADGGWLNAITVGGVTYTYNQKTDSAGVSGGTSVGTFNTATNEWTITLAGGI
LKVDMDNGAYTYTPPTSIPVGGINQVVGYSVIDNDGDTASSTLSIVINPAVGPTVVRDDFIITNQDPATIPDWALLANDTGPLAATQMITSVSGAVGGTV
TDNAGSVSFDDTSGNATPNSYEGSFNYTNSTTTDTAKVYVDVQSGSTLTGSYLDEILIGGSGNDTLNGNAGNDILLGGLGNDTLNGGEGNDLLIGGAGND
TLNGGNGNDTASYFDSAAGVTVTVNGASQNTGGAGTDSLSNMENLVGSMFNDSLTGDGNANVLSGLAGNDILSGGGGDDLLIGGTGSDTLTGGAGKDTFK
WMAGDAGGTDTIKDFTTGANGDVLDLSELLSGEHADASTLDHYLTFAPGPGSGKSTLTIDLDGAGSGTTTHTILFDNVDLTAGKSNLQIIQDLLDQGNLKVDP

Aer-5 WP_128296818.1
MDMRTQIIDKTVVVSSVEGNVQILLADGSSRSLQPGEILQPGARLNIADDAKLMLAPYDDKPAEGNGVPAGTEMPPEGQSSSSVANHQQGSPEVSPEIAA
LQQSILQGVDPTKNFEASAAGGAPAAGGGGGIGGVAGASGNGGFVVIDRIGDATIAEAGFDTGYDTLIDQNVLQEDDLLPENQLDDQDESVVTQEDQAIS
GNVLDNSNNPDGPLDSSVISYSWGSNQGIAAGVESTLDGIGTLIINADGSYTFTPALNYTGPVPAVNYTLTDGDDTNDSTLTITITPVDEPVSLNGLQVE
GGELVLDEAALPDGSASDGAALTKSSTFTFNAADGVQSLTLGGVTLISNGQVLTTFPQGIPSPLGNQLLVTSVSYNPVTGVGSVTYSYTLNDNETHNKPA
NDGALTESFNVVLTDLDGDTTSAGLDVVVLDDVP

A1 repeats

TAVSDVNPTAASENNLVLNGNVITNDVQGADGAAVTAGTLSGTYGSLVLNANGTYTYTLNPNDGDFKALTGGGVGSEVFTYTLTDADGDVSTATLTLTIK
TAVSDVNLTVASENNLVLNGNVITNDVQGADGAAVTAGTLSGTYGSLVLNANGTYTYTLNPSDGDFKALTGGGVGSEVFTYTLTDADGDVSTATLTLTIK

NDDDGVTITNLTPKGEGGDAKVYEDDLLATRGENESAGSDSSKESTTVTGDFTISAPDGVKTLSVGGINIVVNGVVAGFPLSGVTTPLGNTLTITGYNPA
NDDDGVTITNLTPKGEGGDAKVYEDDLLATRGENESAGSDSSKESTTVTGDFTISAPDGVKTLSVGGINIVVNGVVAGFPLSGVTTPLGNTLTITGYNPA

TGLVSYSYTLNDNGAHPTADGNNSLFEDFAVSLVDSDGDSASNTLSVQIVDDVP
TGLVSYSYTLNDNGAHPTADGNNSLFEDFAVSLVDSDGDSASNTLSVQIVDDVP

TANDDADSATEGVFTPVTGNVITGAGTIGGTAGAGVDVMGADGAAISQVVSSNGNSDSNPAGGFTVTGLYGTLTMGLDGEYSYTLTAASVPVGASEVFTY
TLKDGDGDTDPATLTINIAQDTRVPVLTIGNATVDEEGLPEGTAASGNSEIFNGSFTINTQGENLTTLTIGGQTYNLTSGGSQTLINNTEGMLTVTGVSG
PTAGVYTVNYTYTLKDNVLTHNVQGNSDTANGPSFVVSATDASGDSGTGNLQVVISDDAPIANAVTNAGQATQVQNTNLMLILDISGSMGDPSGYQGMTR
MQVMQKSALELLDKYSAYGSVMVNIVTFATSAANPTGVWVSIDVAKAIILGLTPTNLTNYDDALNEAIKAFGDTGKLANAQNVSYFMSDGLPNANSLSGT
PATVPDGNNSLGGGNGIDGDGTTLSGETKDWADFLTANNINSYALGMGSGATQSALDPIAYNGVSGASPANTTAVVVTDFSQLTATLLSTVVAPPLAGQL
INGVAASAGADGGWLNAITVGGVTYTYNQKTDSAGVSGGTSAGTFNTTTNEWTITLAGGILKVDMDNGAYTYTPPTSIPVGGINQVVGYSVIDNDGDTAS
STLSIVINPAVGPTVVRDDFIITNQDPATIPDWALLANDTGPLAATQMITGVSGAVGGTVTDNAGSVTFDDTSGNATPNSYEGSFNYTNSTTTDTAKVYV
DVQSGLTLTGSYLDEILIGGSGNDTLNGNAGNDILLGGLGNDTLNGGEGNDLLIGGAGNDTLNGGNGNDTASYFDSAAGVTVTVNGASQNTGGAGTDSLS
NMENLVGSMFNDSLTGDGNANVLSGLAGNDILSGGGGDDLLIGGTGSDTLTGGAGKDTFKWMAGDAGGTDTIKDFTTGANGDVLDLSELLSGEHADASTL
DHYLTFAPGPGSGKSTLTIDLDGAGSGTTTHTILFDNVDLTAGKSNLQIIQDLLDQGNLKVDP

Aer-11 ARW81648.1

MRTQVIDKTVVVSSIEGNVQILLADGSSRPLQKGEILQPGAKLNIADDAKLLLAPYDDAPTATTPDSSAPDVPVPGLQQVPDDGTATSPEIAALQQSILQ
GVDPTQNFEASAAGGAPAAGGGGGGIGGVAGASGNGGFVTIDRTGDATIAAAGFDTAYQTEPVVDTEQLAEPLLTNELSDEGEQLTVVEGAVLNGNLLTN
TVNTDGPEAASVPLFSWGDNINVTAGTSVTIDGIGSLIVNSDGSFTFTPAPNYDGAVPPVTYVVSDGTDTVQSTLELTITPVNALSDQSESVVVTEDSAV
SGNLLVNTTDNDGPQAPSITGFSWGEITNPVLGTPVTLAGVGTLLVNADGSYQFTPAPNYDGAVPPVNYSVTDGVDTVQSTLSITITPVDEPAELGGFQL
EGGDLTLSEAALADGSGSDTTALTQSGVFTFSAADGVQSLTLGDITLISNGLSITAFPQTITSPLGNQLVVTGINYNPVTGAGSVSYSYTLTDNEVHPSG
GGNNNLFEDFAVTLVDSDGDSANNTLSVRIVDDVP

A1 repeats

TASNDSNPGVASEAGLVLGGNVLGNDVQGADGAAVTAGTLTGTYGSLVLGADGSYTYTLNPADPQFVALPGGATGSEVFTYVLTDGDGDTSTATLTLNIR
TASNDSNPGVASEAGLVLGGNVLGNDVQGADGAAVTAGTLTGTYGSLVLGADGSYTYTLNPADPQFVALPGGATGSEVFTYVLTDGDGDTSTATLTLNIR
TASNDSNPGVASEAGLVLGGNVLGNDVQGADGAAVTAGTLTGTYGSLVLGADGSYTYTLNPADPQFVALPGGATGSEVFTYVLTDGDGDTSTATLTLNIR
TASNDSNPGVASEAGLVLGGNVLGNDVQGADGAAVTAGTLTGTYGSLVLGADGSYTYTLNPADPQFVALPGGATGSEVFTYVLTDGDGDTSTATLTLNIR
TASNDSNPGVASEAGLVLGGNVLGNDVQGADGAAVTAGTLTGTYGSLVLGADGSYTYTLNPADPQFVALPGGATGSEVFTYVLTDGDGDTSTATLTLNIR
TASNDSNPGVASEAGLVLGGNVLGNDVQGADGAAVTAGTLTGTYGSLVLGADGSYTYTLNPADPQFVALPGGATGSEVFTYVLTDGDGDTSTATLTLNIR
TASNDSNPGVASEAGLVLGGNVLGNDVQGADGAAVTAGTLTGTYGSLVLGADGSYTYTLNPADPQFVALPGGATGSEVFTYVLTDGDGDTSTATLTLNIR

NDDDNVTITDLTPKGEGGDATVFEDDLLSTRGAGESAGSDSSKESTTVTGSFNISAPDGVQTLSVGGINLVSGGVVASVPQSVTTGFGNTLTITAYNPAT
NDDDNVTITDLTPKGEGGDATVFEDDLLSTRGAGESAGSDSSKESTTVTGSFNISAPDGVQTLSVGGINLVSGGVVASVPQSVTTGFGNTLTITAYNPAT
NDDDNVTITDLTPKGEGGDATVFEDDLLSTRGAGESAGSDSSKESTTVTGSFNISAPDGVQTLSVGGINLVSGGVVASVPQSVTTGFGNTLTITAYNPAT
NDDDNVTITDLTPKGEGGDATVFEDDLLSTRGAGESAGSDSSKESTTVTGSFNISAPDGVQTLSVGGINLVSGGVVASVPQSVTTGFGNTLTITAYNPAT
NDDDNVTITDLTPKGEGGDATVFEDDLLSTRGAGESAGSDSSKESTTVTGSFNISAPDGVQTLSVGGINLVSGGVVASVPQSVTTGFGNTLTITAYNPAT
NDDDNVTITDLTPKGEGGDATVFEDDLLSTRGAGESAGSDSSKESTTVTGSFNISAPDGVQTLSVGGINLVSGGVVASVPQSVTTGFGNTLTITAYNPAT
NDDDNVTITDLTPKGEGGDATVFEDDLLSTRGAGESAGSDSSKESTTVTGSFNISAPDGVQTLSVGGINLVSGGVVASVPQSVTTGFGNTLTITAYNPAT

GLVSYSYTLTDNEVHPSGGGNNNLFEDFAVTLVDSDGDSANNTLSVRIVDDVP
GLVSYSYTLTDNEVHPSGGGNNNLFEDFAVTLVDSDGDSANNTLSVRIVDDVP
GLVSYSYTLTDNEVHPSGGGNNNLFEDFAVTLVDSDGDSANNTLSVRIVDDVP
GLVSYSYTLTDNEVHPSGGGNNNLFEDFAVTLVDSDGDSANNTLSVRIVDDVP
GLVSYSYTLTDNEVHPSGGGNNNLFEDFAVTLVDSDGDSANNTLSVRIVDDVP
GLVSYSYTLTDNEVHPSGGGNNNLFEDFAVTLVDSDGDSANNTLSVRIVDDVP
GLVSYSYTLTDNEVHPSGGGNNNLFEDFAVTLVDSDGDSANNTLSVRIVDDVP

VSPVDLNTRVEEFSNPGTNLMIILDTSGSMDYASGVAGFATRMAIAKASILQLIGDYDDVGDVMVRLVGFASSATTNFLGSGDVWLTAAQALNVINGITD
YLGDGGTDYDDALIKAMSAYDSAGKIIGGQSVLYFLSDGEPTESTNWPGVSGTGSTGINTAEQTAWEAFLNSNDINAYALGMGTGATAGALTPIAYNGSS
GQQQPAIIVTDLSQLASTLSGTVQVPTTGNLLTDAGVVFGADGPATLPITSISHDADGNPATPDVVYTTTYSGYNAITHVLTIPTHGGGTFSVNLLTGAY
SYSLSLDVANDYTETFRYTISDADGDVKTGVLNMITTDSSDVIAYDNGDEALVTQIMVPGATTSTTLADFGDTTNSANSGAGYNPWIYDTSGTGLSVMDL
GSTSIGSAVASNGNKWIVSTLSSSTLDGSVSGGALQLVDSNDDGAGAAELLTPEFVTGLAGTTTLSFQYDRGNVHASDTVTWSLYKFDGSSWVQLSGVGF
SGNLSGDPGSLSTITTSTLDAGTRYRVYFSVNDGSGNSDSTLQLDNIKLNVTAAATAAIALTAAHGNLLTDPNHDMMSGDPWGSVDAIGAESTVLKIWNG
SSYTAVTTSQTVAGLYGSLEIHSDGAYTYTPNADLNNVGKADVFNYQLAQSDGDQDTAQLTINIASSATAAPTVMEGTSANDALVGTSGDDVLLGHAGAD
VLNGNDGNDRLEGGAGNDTLIGGLGEDILIGGLGSDTMTGGAGSDTFKWLAGEADGSTDNITDFTLGNTASGGDVLDLSDLLVGVPTGGSNDDLAAALDS
YLQFDTATKTLTIDPDGAVGGSELTIQFQNSLDLASLGSNQDIIKQLLDDGNLKVDP

Aer-13 GAJ50574.1

MDMRTQVIDKTVVVSSIEGNVQILLADGSSRPLQKGEILQPGAKLNIADDAKLLLAPYDDAPTATTPDSSAPDVPVPGLQQVPDDGTATSPEIAALQQSI
LQGVDPTQNFEASAAGGAPAAGGGGGGIGGVAGASGNGGFVTIDRTGDATIAAAGFDTAYQTEPVVDTEQLAEPLLTNELSDEGEQLTVAEGEVLNGNLL
TNTVNTDGPEAASVPLFSWGDNINVTAGTSVTIDGIGSLIVNTDGSFTFTPAPNYDGAVPPVTYVVSDGTDTVQSTLELTITPVNALSDQSESVVVTEDS
AGSGNLLVNTTDNDGPQAPSITGFSWGAITDPVLGTPVTLTGVGTLLVNADGSYQFTPAPNYDGSVPPVNYSVTDGVDTVQSTLSITITPVDEPAELGGF
QLEGGDLTLSEVALADGSDSDTTALTQSGVFTFNAADGVQSLTLGDITLISNGLSITAFPQTITSSLGNQLVVTGINYNPVTGAGSVSYSYTLTDNEEHP
SGEGNNNLFEDFAVTLVDSDGDSANNTLSVRIVDDVP

A1 repeat

TASNDSNPGVASEASLVLGGNVLGNDVQGADGAAVTAGTLTGTYGSLVLGADGSYTYTLNPADPQFVALPGGATGSEVFTYVLTDGDGDTSTATLTLSIR
NDNDNVTITDLTPKGDGGDATVFEDDLLSTRGTGESAGSDSSKESTTVTGSFTISAPDGVQTLSVGGISLVSGGVVASVPQSVTTGFGNTLTITAYNPAT
GLVSYSYTLTDNEEHPSGGGNNNLFEDFAVTLVDSDGDSANNTLSVRIVDDVP

VSPVDLNTRVEEFSNPGTNLMIILDTSGSMDDASGVAGFATRMAIAKASILQLIGDYDDVGDVMVRLVGFASSATTNFLGAGDVWLTATQALNVINGITD
YLGNGGTDYDDALIKAMSAYDSAGKIIGGQSVLYFLSDGEPTESTNWPGVSGTGSTGINTAEQTAWEVFLNGNYVNAYALGMGTGATTGALTPIAYNGST
GQQQPAIIVTDLSQLASTLSGTVQVPTTGNLLTDAGVVFGADGPATLPITSISHDADGNPATPDVVYTTTYSGYNAITHVLTIPTHGGGTFSVNLLTGAY
SYSLSLDVANDYTETFRYTISDADGDVKTGVLNMITTDSSDVIAYDNGDEALVTQIMVPGATTSTTLADFGDTTNSANSGAGYNPWIYDTSGTGLSVMDL
GSTSIGSAVASNGNKWIVSTLSSSTLDGSVSGGALQLVDSNDDGAGAAELLTPEFVTGLAGTTTLSFQYDRGNVHASDTVTWSLYKFDGSSWVQLSGVGF
SGNLSGDPGALSTITTSTLDAGTRYRVYFSVNDGSGNPDSTLQLDNIKLNVTAAATAAIALTAAYGNLLTDPNHDMMSGDPWGSVDAIGAESTVLKIWNG
SSYTAVTTSQTVAGLYGSLEIHSDGAYTYTPNADLNNVGKADVFNYQLAQSDGDQDTAQLTINIASSATVAPTMEGSSANDALVGTSGDDVLLGHAGADV
LNGNDRLEGGAGNDTLIGGLGDDILIGGLGSDTMTGGAGSDTFKWLAGEADGSTDNITDFTLGNTASGGDVLDLSDLLVGVPTGGSNDDLAAALDSYLQF
DTATKTLTIDPDGAVGGSELTIQFQNSLDLASLGSNQDIIKQLLDDGNLKVDP

Aer-15 WP_130632086.1

MRTQIIDKTVVVSSIEGSVQIVLADGSSRPLQKGEILQPGAKLNIADDAKLVLAPYDDSPAAATPDAPAHDAPAPGQPQAPDAGTAASPDIAALQKSILQ
GVDPTQNFEASAAGGAPAAGGGGGIGGVAGASGNGGFVTIDRIGDATIAAAGFDTSYQTEPVVDTQQVVEPLLVNELTDQGEQLVVAEDGVLNGNLLDNT
VNTDGPSAASVLLFSWGGNANVAVGTSVTIDGIGTLVVNGDGSFTFTPAPNYDGAVPPVVYTVTDGTDTVQSTLELTITPVNDLADQGESVAVTEDATVS
GNLLDNTVDNDGPQAATVTGFSWGGLANTTLGTPVTLAGVGTLLVNADGSYQFTPATNYDGPVPAVSYTVTDGTDSVQSILTITITPVDEPVELAGLQLE
GGELTLNEASLAGGSNPNAAAVTQSGIFTFSAADGVQSLTLGGVALVTNGQAVTAFPQTITSPLGNQLIVTGINYNPVTGTGSVNYSYTLGGSETHTQPA
NDNSLSESFSVVLVDTDGDNTSGSLDVVILDDVP

A3 repeats

SVTLTTNSEGLGSVSVDESLVSLGGVGTDGVVSATLSAATVQAQFNPAFGADGAGSIGYSLALTGSNVASGLYAVDPAAANGQGAAIVLNQVGNVITGSA
RAGLAVEAPSLGA-SVDESLVSLGGAGSDGVASASLSAANVQAQFNPAFGADGAGSIGYSLALTGSNVASGLYAVDPAAANGQGAAIVLNQVGNVITGSA
RAGLAVEAPSLGA-SVDESLVSLGGAGSDGVASATLSAANVQAQFNPAFGADGAGSIGYSLALTGSNVASGLYAVDPAAANGQGAAIVLNQVGNVITGSA
RAGLAVEAPSLGA-SVDESLVSLGGVGSDGVASATLSAANVQAQFNPAFGADGAGSIGYSLALTGSNVASGLYAVDPAAPNGQGAAIVLNQVGNVITGSA
RAGLAVEAPSLGA-SVDESLVSLGGVGSDGVVSATLSAANVQSQFNPAFGADGAGSIGYSLALTGSNVASGLYAVDPAAPNGQGAAIVLNQVGNVITGSA
RAGLAVEAPSLGA-SVDESLVSLGGVGSDGVASATLSAANVQAQFNPAFGADGAGSIGYSLALTGSNVASGLYAVDPAAPNGQGAAIVLNQVGNVITGSA
RAGLAVEAPSLGA-SVDESLVSLGGVGSDGVASATLSAANVQAQFNPAFGADGAGSIGYSLALTGSNVASGLYAVDPAAPNGQGAAIVLNQVGNVITGSA
RAGLAVEAPSLGA-TVDESLVSLGGVGSDGVASATLSAANVQAQFNPAFGADGAGSIGYSLALTGSNVASGLYAVDPAVANGQGAAIVLNQVGNVITGSA
RAGLAVEAPSLGA-SVDESLVSLGGVGSDGVASATLSAANVQAQFNPAFGADGAGSIGYSLALTGSNVASGLYAVDPAAPNGQGAAIVLNQVGNVITGSA
RAGLAVEAPSLGA-TVDESLVGLGGVGSDGVASATLSAANVQAQFNPAFGADGAGSIGYSLALTGSNVASGLYAVDPAVANGQGAAIVLNQVGNVITGSA
RAGLAVEAPSLAA-TVDESLVSLGGVGSDGVASATLSAANVQAQFNPAFGADGAGSIGYSLALTGSNVASGLYAVDPAAPNGQGAAIVLNQVGNVITGSA

GGVDYFTLTINPSTGEVTLALLDNVWHGDTTNADDSVALTLGQGVLTLVQTVTDADGDSASAAVDLGANGVFRFEDDGP
GGVDYFTLTINPSTGEVTLALLDNVWHGDTTNADDSVALTLGQGVLTLVQTVTDADGDSASAAVDLGANGVFRFEDDGP
GGVDYFTLTINPTTGEVTLALLDNVWHGDTTNADDSVALTLGQGVLTLVQTVTDADGDSASAAVDLGANGVFRFEDDGP
GGVDYFTLTINPSSGEVTLALLDNVWHGDTTSADDSVALTLGQGVLTLVQTVTDADGDSASAAVDLGANGVFRFEDDGP
GGVDYFTLTINPSSGEVTLALLDNVWHGDTTSADDSVALTLGQGVLTLVQTVTDADGDSASAAVDLGANGVFRFEDDGP
GGVDYFTLTINPSSGEVTLALLDNVWHGDTTSADDSVALTLGQGVLTLVQTVTDADGDSASAAVDLGANGVFRFEDDGP
GGVDYFTLTINPTTGEVTLALLDNVWHGDTTSADDSVALTLGQGVLTLVQTVTDADGDSASAAVDLGANGVFRFEDDGP
GGVDYFTLTINPSSGEVTLALLDNVWHGDTTSADDSVALTLGQGVLTLVQTVTDADGDSASAAVDLGANGVFRFEDDGP
GGVDYFTLTINPTTGEVTLALLDNVWHGDTTSADDSVALTLGQGVLTLVQTVTDADGDSASAAVDLGANGVFRFEDDGP
GGVDYFTLTINPSSGEVTLALLDNVWHGDTTNADDSVALTLGQGVLTLVQTVTDADGDSASAAVDLGANGVFRFEDDGP
GGVDYFTLTINPSSGEVTLALLDNVWHGDTTSADDSVALTLGQGVLTLVQTVTDADGDSASAAVDLGANGVFRFEDDGP

A4 repeats

SVTINAVADGGITLTTQDAQTIDAASDTATGSFAAAFLAAAVPSYGADGPGTTTVSGYSLSVTDSNSGLTSNGLAITLTKVGSDIVGSTAAGEVFRISVA
SVTINAVVDGGITLTTQDAQTIGSASDTATGSFAAAFLAAAVPSYGADGPGTTTVSGYSLSVTDSNSGLTSNGLAITLTKVGSDIVGSTAAGEVFRISVA
SVTINAVVDGGITLTTQDAQTIGSASDTATGSFAAAFLAAAVPSYGADGPGTTTVSGYSLSVTDSNSGLTSNGLAITLTKVGSDIVGSTTAGEVFRISVA
SVTINAVVDGGITLTTQDAQTIGSASDTATGSFAAAFLAAAVPSYGADGPGTTTVSGYSLSVTDSNSGLTSNGLAITLTKVGSDIVGSTTAGEVFRISVA
SVTINAVVDGGITLTTQDAQTIGSASDTATGSFAAAFLAAAVPSYGADGPGTTTVSGYSLSVTDSNSGLTSNGLAITLTKVGSDIVGSTTAGEVFRISVA
SVTINAVVDGGITLTTQDAQTIGSASDTATGSFAAAFLAAAVPSYGADGPGTTTVSGYSLSVTDSNSGLTSNGLAITLTKVGSDIVGSTTAGEVFRISVA

SNGTVTLTQSAELDHLPEGVDNSNDNNLISLANGKVLLSATVTVVDGDNDTATGTVSADLGGNISFEDDVP
SNGTVTLTQSAELDHLPEGVDNSNDNNLISLANGKVLLSATVTVVDGDNDTATGTVSADLGGNISFEDDVP
SNGTVTLTQSAELDHLPEDVDNSNDNNLISLANGKVLLSATVTVVDGDNDTATGTVSADLGGNISFEDDVP
SNGTVTLTQSAELDHLPEDVDNSNDNNLISLANGKVLLSATVTVVDGDNDTATGTVSADLGGNISFEDDVP
SNGTVTLTQSAELDHLPEDVDNSNDNNLISLANGKVLLSATVTVVDGDNDTATGTVSADLGGNISFEDDVP
SNGTVTLTQSAELDHLPEDVDNSNDNNLISLANGKVLLSATVTVVDGDNDTATGTVSADLGGNIRFEDDVP

TAKDNSVVITEAGLPPFNLVMVIDTSGSMLWQIGTSTNGSPNRLELAKDALNHMIDSYVALGVPLVFTVIDFASGAVLIPQTSDPDVAKASISGLPTDGG
GTNYNAPLVLAQNQLTADLANPALAGYETKVYFLSDGAPNEGNVPAGWTSFVNSNNVEVYAVGLNVSGNATAIAQLGLVEDHGDAVTLVNNIYDLDATLQ
ATVPPPATGNVITDVDASAGVDSAGADTPVTVFQVSFTVSNPAAYVGVADSVNGNVVTFLVQGGTTGPITTPLGGHLTVNADGSYSYTPPNDVAVDTQET
FTYTIVDADGDTSSALLKILVLDTTPITASVFEDGLPGGLPGNNPALTTVAGSLAHMVVDSIGVNFGVGSTAGLPALTSGGVAITYSIIHGVGVDTLVAV
AGSKPIFTLAVEADGDYTFTLQGPIDHALADGNDLELKALNLSSAITAHEGVDNVTLVRDFIVQVEDDVPIVMTPAMGVMLNQSGVTGIFLLDGDGSLSN
NYGGDGGSIRFDSSLNGSSSGMTSGGLPVTYTLSSDGITLTAATAAGTVFIATLQPASGTYQVQMIGEVDGGQTTIDFNGGDYNFVGGNASWAGFNTIAN
DNSKDLLLTAMTNGADGGTVNTSANTGGISSGASVGSGEAMRIDFVIDLTGNPVSGGSYGNLANQTHAFEAHYDVNGASALFTSISSGGSSVKVVARQDV
DSDNDIGDGNKESLTAVAISYNGSTRIVSIGAGMSQTVAVGGRSFTVNFTDDDPTSGVAYVATVVGVVSNTKLAAYTADGYNSLEFHYAAGSDFKIGDFG
SSVATPGIPVSLQLPVTLTDGDGDAVHSNIGLSLLPEAPFTLDYDSSASGVNVTFNSTQGHAIGSDFGDSITGNSLDNILSGGDGDDLLYGLGGKDTLVG
GKGNDVLDGGSGNDTLYGEAGDDTLIGGIGNDILQGGDGKDLLIGGLGSDTMTGGAGSDTFKWLAGDADGSTDNITDFTLGNPTSGGDVLDLSDLLVGVP
SAANNNDLATALDNYLKFDTATNKLTIDTNGLTSGGSQLTVQFQGSLDLDHNGGLTTNHDIIKQLLDDGNLKVDP

Aer-27 WP_158197039.1

MHPPKTEQIQYIFATDAKRTDIYGVDIEICHVGAKDMRTQIIDKTVVVSSIEGSVQIVLADGSSRPLQKGEILQPGAKINIADDAKLVLAPYDDSPAAAT
PDAPAHDAPAPGQPQAPDAGTAASPDIAALQKSILQGVDPTQNFEASAAGGAPAAGGGGGIGGVAGASGNGGFVTIDRIGDATIAAAGFDTTYQTEPVVD
TQQVVEPLLVNELTDQGEQLVVAEDGVLNGNLLDNTVNTDGPSAASVLLFSWGGNANVAVGTSVTIDGIGTLVVNGDGSFTFTPAPNYDGAVPPVVYTVT
DGTDTVQSTLELTITPVNDLADQGENVVVTEDATVSGNLLDNTVDNDGPQAATVTGFSWGGVANSILGTPVTLAGVGTLLVNADGSYQFTPATNYDGPVP
AVSYTVTDGTDSVQSILTITITPVDEPVELAGLQLEGGELTLNEASLAGGSNPNAAAVTQSGTFTFSAADGVQSLTLGGVALVTNGQAVTAFPQTITSPL
GNQLIVTGINYNPVTGTGSVNYSYTLGGSETHTQPADDNSLSESFSVVLVDTDGDNTSGSLDVVILDDVP

A3 repeats
SVTLTTNSEGLGAVSVDESLVSLGGVGSDGVASATLSAGTVQAQFNPAFGADGAGSIGYSLALTGSNVASGLYAVDPAAANGQGAAIVLNQVGNVITGSA
RAGLAVEAPSLGA-SVDESLVSLGGVGTDGVASATLSAATVQAQFNPAFGADGAGSIGYSLALTGNNVASGLYAVDPAAANGQGAAIVLNQVGNVITGSA

GGVDYFTLTINPTTGEVTLALLDNVWHGDTTNADDSVALTLGQGVLTLVQTVTDADGDSASAAVDLGANGVFRFEDDGP
GGVDYFTLTINPTTGEVTLALLDNVWHGDTTNADDSVALTLGQGVLTLVQTVTDADGDSASAAVDLGANGVFRFEDDGP

A4 repeats
SVTINAVADGGITLTTQDAQTIGSASDTATGSFAAAFLAAAVPSYGADGPGTTTVSDYSLSVTDSNSGLTSNGLAITLTKVGSDIVGSTTAGEVFRISVA
SVTINAVADGGITLTTQDAQTIGSASDTATGSFAAAFLAAAVPSYGADGPGTTTVSDYSLSVTDSNSGLTSNGLAITLTKVGSDIVGSTTAGEVFRISVA
SVTINAVADGGITLTTQDAQTIGSASDTATGSFAAAFLAAAVPSYGADGPGTTTVSDYSLSVTDSNSGLTSNGLAITLTKVGSDIVGSTTAGEVFRISVA
SVTINAVADGGITLTTQDAQTIGSASDTATGSFAAAFLAAAVPSYGADGPGTTTVSDYSLSVTDSNSGLTSNGLAITLTKVGSDIVGSTTAGEVFRISVA
SVTINAVADGGITLTTQDAQTIGSASDTATGSFAAAFLAAAVPSYGADGPGTTTVSDYSLSVTDSNSGLTSNGLAITLTKVGSDIVGSTTAGEVFRISVA
SVTINAVADGGITLTTQDAQTIGSASDTATGSFAAAFLAAAVPSYGADGPGTTTVSDYSLSVTDSNSGLTSNGLAITLTKVGSDIVGSTTAGEVFRISVA
SVTINAVADGGITLTTQDAQTIGSASDTATGSFAAAFLAAAVPSYGADGPGTTTVSDYSLSVTDSNSGLTSNGLAITLTKVGSDIVGSTTAGEVFRISVA
SVTINAVADGGITLTTQDAQTIGSASDTATGSFAAAFLAAAVPSYGADGPGTTTVSDYSLSVTDSNSGLTSNGLAITLTKVGSDIVGSTTAGEVFRISVA

SNGTVTLTQSAELDHLPEDVDNSNDNNLISLANGKVLLSATVTVVDGDNDTATGTVSADLGGNISFEDDVP
SNGTVTLTQSAELDHLPEDVDNSNDNNLISLANGKVLLSATVTVVDGDNDTATGTVSADLGGNISFEDDVP
SNGTVTLTQSAELDHLPEDVDNSNDNNLISLANGKVLLSATVTVVDGDNDTATGTVSADLGGNISFEDDVP
SNGTVTLTQSAELDHLPEDVDNSNDNNLISLANGKVLLSATVTVVDGDNDTATGTVSADLGGNISFEDDVP
SNGTVTLTQSAELDHLPEDVDNSNDNNLISLANGKVLLSATVTVVDGDNDTATGTVSADLGGNISFEDDVP
SNGTVTLTQSAELDHLPEDVDNSNDNNLISLANGKVLLSATVTVVDGDNDTATGTVSADLGGNISFEDDVP
SNGTVTLTQSAELDHLPEDVDNSNDNNLISLANGKVLLSATVTVVDGDNDTATGTVSADLGGNISFEDDVP
SNGTVTLTQSAELDHLPEDVDNSNDNNLISLANGKVLLSATVTVVDGDNDTATGTVSADLGGNISFEDDVP

TAKDNNVVITEAGLPPFNLVMVIDTSGSMLWQIGTSTNGSPNRLELAKDALNHMIDSYVALGVPLVFTVIDFASGAVLIPQTSDPDVAKASISGLPTDGG
GTNYNAPLVLAQNQLTADLANPALAGYETKVYFLSDGAPNEGNVPAGWTSFVNSNNVEVYAVGLNVSGNATAIAQLGLVEDHGDAVTLVNNIYDLDATLQ
ATVPPPATGNVITDVDTTAGVDSAGADTPVTVLQVSFTVSNPAAYVGVADSVNGNVVTFLVQGGTTGPMTTPLGGHLTVNADGSYSYTPPNDVAVDTQEA
FTYTIVDADGDTSSALLKILVLDTTPVTASVFEDGLPAGLPGNNPALTTVAGSLAHMVVDSIGVNFGVGSTAGLPALTSGGVAITYSIIHGVGVDILVAV
AGSKPIFTLAVEADGDYTFTLQGPIDHALADGNDLELKALNLSSAITAHEGVDNVTLVRDFIVQVEDDVPVVMTPAMGVMLNQSGVTGIFLLDGDGTLSN
NYGGDGGSIRFDSSLNGSSSGMTSGGLPVTYTLSSDGITLTAATAAGTVFIATLQPASGTYQVQMIGEVDGGQTMIDFSGGGYKFVGGNGPWAGFNTLAN
DNSKDLLLTAMTNGVDGGTVNTTSHTGGISGGASVGTGEAMRIDFVIDLTGSPGSSGSYGDPATHAFEAHYDVNGASALFTSITNSSSVRLVARQDVDND
NDIGDGNKESLTAVAISYNGSTKIVNIGAGMSQTVAVGGNYFTVNFTDDDPGTGVTYVATVVGVVTNTKLAAYTADGYNSLEFHYVDGNTFKIGDFGSSV
ATPGIPVSLQLPVTLTDGDGDAVHSNIGLSLLPEAPFTLDYDSSASGVNVTFNSTQGHAIGSDFGDSITGNSLDNILSGGDGDDLLYGMGGKDTLVGGKG
NDVLDGGSGNDTLYGEAGDDTLIGGIGNDILQGGDGKDLLIGGLGSDTMTGGAGSDTFKWLAGDADGSTDKITDFTLGNPTSGGDVLDLSDLLVGVPSAA
NNNDLATALDNYLKFDTATNKLTIDTNGLTSGGSQLTVQFQGSLDLDHNGGLTTNHDIIKQLLDDGNLKVDP

Aer-32 WP_158198304.1
MRTQIIDKSVVVSAIEGNVEIVLADGSSRPLQKGEILQPGAKLAIADDAKLALSPYDDTPASDVPAGSESAEPAQPQVAASQEGAPSDIAALQESILQGV
DPTQNFEASAAGGAPAAGGGGGIGGVAGTSGNGGFVTIDRTGDATIAAAGFDTTYDTAAVINENQAEDLILENQLDDLGETAVTLEDSLVSGNLLENSTN
PDGPADASIVSYDWGINIGVPAGVAATITGVGTLIINPDGSYTFTPAPNYDGAVPPVIYQVTDGQDTVTSTLVISITPVDEEVSLDGLTQEGGEVLVSDA
NLADGSAPNDAALTQSGVFTFNAPDGVQSLTLAGVTLITNGQLGASFPQSIISPLGNVLTISAVTYNPVTGAGQVSYSYTLGDNENHTRPANDTSLSESF
SVVLTDSDGDTASDTLDITILDDIP
A5 repeat
TLAGDIGSTLGGQVQEDALTSGNSEGTGQSASASGAAGALNNLVNFGADGAGSFGLGSDVSSLTAQGLTSGGVALSYSVVGNVLTASAGSVPVFTLTVGA
DGSYSFTLSGPLDHPVADGDDSEQLAGVGIDFSGVLTATDGDGDPLTGGFPAGSFAIKVEDDVP
A3 repeat
RVVLADDAPRLGATVDESLASLGGVGGDGVASASLSAADVQAQFVPAFGADGQGSIGYSLALSGTNVASGLYAVDPLAANGQGAAIVLNKVGNVITGSAG
GVDYFTLTINPTTGEVTLALLDNVWHGDTTNADDSVVLSVDEGVLTLVQTVTDADGDRASASVDLGANGVFRFEDDGP
A6 repeats
SISAGSAANDSLQVDESNLSLNASTSFASLFTSSFGADGAGTLVYSLNVSAAGADSGLNDTATGQDILLYLESGSVVGRVGGSSGSVAFTLSVNSSGEVT
SISAGSAANDSLQVDESNLSLNASTSFASLFTSSFGADGAGTLVYSLNVSAAGADSGLNDTATGQDILLYLESGSVVGRVGGSSGSVAFTLSVNSSGEVT
SISAGSAANDSLQVDESNLSLNASTSFASLFTSSFGADGAGTLVYSLNVSAAGADSGLNDTATGQDILLYLESGSVVGRVGGSSGSVAFTLSVNSSGEVT
SISAGSAANDSLQVDESNLSLNASTSFASLFTSSFGADGAGTLVYSLNVSAAGADSGLNDTATGQDILLYLESGSVVGRVGGSSGSVAFTLSVNSSGEVT
SISAGSAANDSLQVDESNLSLNASTSFASLFTSSFGADGAGTLVYSLNVSAAGADSGLNDTATGQDILLYLESGSVVGRVGGSSGSVAFTLSVNSSGEVT
SISAGSAANDSLQVDESNLSLNASTSFASLFTSSFGADGAGTLVYSLNVSAAGADSGLNDTATGQDILLYLESGSVVGRVGGSSGSVAFTLSVNSSGEVT
SISAGSAANDSLQVDESNLSLNASTSFASLFTSSFGADGAGTLVYSLNVSAAGADSGLNDTATGQDILLYLESGSVVGRVGGSSGSVAFTLSVNSSGEVT
SISAGSAANDSLQVDESNLSLNASTSFASLFTSSFGADGAGTLVYSLNVSAAGADSGLNDTATGQDILLYLESGSVVGRVGGSSGSVAFTLSVNSSGEVT
SISAGSAANDSLQVDESNLSLNASTSFASLFTSSFGADGAGTLVYSLNVSAAGADSGLNDTATGQDILLYLESGSVVGRVGGSSGSVAFTLSVNSSGEVT
SISAGSAANDSLQVDESNLSLNASTSFASLFTSSFGADGAGTLVYSLNVSAAGADSGLNDTATGQDILLYLESGSVVGRVGGSSGSVAFTLSVNSSGEVT
SISAGSAANDSLQVDESNLSLNASTSFASLFTSSFGADGAGTLVYSLNVSAAGADSGLNDTATGQDILLYLESGSVVGRVGGSSGSVAFTLSVNSSGEVT
SISAGSAANDSLQVDESNLSLNASTSFASLFTSSFGADGAGTLVYSLNVSAAGADSGLNDTATGQDILLYLESGSVVGRVGGSSGSVAFTLSVNSSGEVT
SISAGSAANDSLQVDESNLSLNASTSFASLFTSSFGADGAGTLVYSLNVSAAGADSGLNDTATGQDILLYLESGSVVGRVGGSSGSVAFTLSVNSSGEVT
SISAGSAANDSLQVDESNLSLNASTSFASLFTSSFGADGAGTLVYSLNVSAAGADSGLNDTATGQDILLYLESGSVVGRVGGSSGSVAFTLSVNSSGEVT
SISAGSAANDSLQVDESNLSLNASTSFASLFTSSFGADGAGTLVYSLNVSAAGADSGLNDTATGQDILLYLESGSVVGRVGGSSGSVAFTLSVNSSGEVT
SISAGSAANDSLQVDESNLSLNASTSFASLFTSSFGADGAGTLVYSLNVSAAGADSGLNDTATGQDILLYLESGSVVGRVGGSSGSVAFTLSVNSSGEVT
SISAGSAANDSLQVDESNLSLNASTSFASLFTSSFGADGAGTLVYSLNVSAAGADSGLNDTATGQDILLYLESGSVVGRVGGSSGSVAFTLSVNSSGEVT
SISAGSAANDSLQVDESNLSLNASTSFASLFTSSFGADGAGTLVYSLNVSAAGADSGLNDTATGQDILLYLESGSVVGRVGGSSGSVAFTLSVNSSGEVT
SISAGSAANDSLQVDESNLSLNASTSFASLFTSSFGADGAGTLVYSLNVSAAGADSGLNDTATGQDILLYLESGSVVGRVGGSSGSVAFTLSVNSSGEVT
SISAGSAANDSLQVDESNLSLNASTSFASLFTSSFGADGAGTLVYSLNVSAAGADSGLNDTATGQDILLYLESGSVVGRVGGSSGSVAFTLSVNSSGEVT
SISAGSAANDSLQVDESNLSLNASTSFASLFTSSFGADGAGTLVYSLNVSAAGADSGLNDTATGQDILLYLESGSVVGRVGGSSGSVAFTLSVNSSGEVT
SISAGSAANDSLQVDESNLSLNASTSFASLFTSSFGADGAGTLVYSLNVSAAGADSGLNDTATGQDILLYLESGSVVGRVGGSSGSVAFTLSVNSSGEVT
SISAGSAANDSLQVDESNLSLNASTSFASLFTSSFGADGAGTLVYSLNVSAAGADSGLNDTATGQDILLYLESGSVVGRVGGSSGSVAFTLSVNSSGEVT
SISAGSAANDSLQVDESNLSLNASTSFASLFTSSFGADGAGTLVYSLNVSAAGADSGLNDTATGQDILLYLESGSVVGRVGGSSGSVAFTLSVNSSGEVT
SISAGSAANDSLQVDESNLSLNASTSFASLFTSSFGADGAGTLVYSLNVSAAGADSGLNDTATGQDILLYLESGSVVGRVGGSSGSVAFTLSVNSSGEVT
SISAGSAANDSLQVDESNLSLNASTSFASLFTSSFGADGAGTLVYSLNVSAAGADSGLNDTATGQDILLYLESGSVVGRVGGSSGSVAFTLSVNSSGEVT
SISAGSAANDSLQVDESNLSLNASTSFASLFTSSFGADGAGTLVYSLNVSAAGADSGLNDTATGQDILLYLESGSVVGRVGGSSGSVAFTLSVNSSGEVT
SISAGSAANDSLQVDESNLSLNASTSFASLFTSSFGADGAGTLVYSLNVSAAGADSGLNDTATGQDILLYLESGSVVGRVGGSSGSVAFTLSVNSSGEVT
SISAGSAANDSLQVDESNLSLNASTSFASLFTSSFGADGAGTLVYSLNVSAAGADSGLNDTATGQDILLYLESGSVVGRVGGSSGSVAFTLSVNSSGEVT

LDQERAIAHTPNSGPDQESSLLSANLVKLVGTITDKDGDSQSASVDLGSAISFKDDGP
LDQERAIAHTPNSGPDQESSLLSANLVKLVGTITDKDGDSQSASVDLGSAISFKDDGP
LDQERAIAHTPNSGPDQESSLLSANLVKLVGTITDKDGDSQSASVDLGSAISFKDDGP
LDQERAIAHTPNSGPDQESSLLSANLVKLVGTITDKDGDSQSASVDLGSAISFKDDGP
LDQERAIAHTPNSGPDQESSLLSANLVKLVGTITDKDGDSQSASVDLGSAISFKDDGP
LDQERAIAHTPNSGPDQESSLLSANLVKLVGTITDKDGDSQSASVDLGSAISFKDDGP
LDQERAIAHTPNSGPDQESSLLSANLVKLVGTITDKDGDSQSASVDLGSAISFKDDGP
LDQERAIAHTPNSGPDQESSLLSANLVKLVGTITDKDGDSQSASVDLGSAISFKDDGP
LDQERAIAHTPNSGPDQESSLLSANLVKLVGTITDKDGDSQSASVDLGSAISFKDDGP
LDQERAIAHTPNSGPDQESSLLSANLVKLVGTITDKDGDSQSASVDLGSAISFKDDGP
LDQVRAIAHTPNSGPDQESSLLSANLVKLVGTITDKDGDSQSASVDLGSAISFKDDGP
LDQERAIAHTPNSGPDQESSLLSANLVKLVGTITDKDGDSQSASVDLGSAISFKDDGP
LDQERAIAHTPNSGPDQESSLLSANLVKLVGTITDKDGDSQSASVDLGSAISFKDDGP
LDQERAIAHTPNSGPDQESSLLSANLVKLVGTITDKDGDSQSASVDLGSAISFKDDGP
LDQERAIAHTPNSGPDQESSLLSANLVKLVGTITDKDGDSQSASVDLGSAISFKDDGP
LDQERAIAHTPNSGPDQESSLLSANLVKLVGTITDKDGDSQSASVDLGSAISFKDDGP
LDQVRAIAHTPNSGPDQESSLLSANLVKLVGTITDKDGDSQSASVDLGSAISFKDDGP
LDQERAIAHTPNSGPDQESSLLSANLVKLVGTITDKDGDSQSASVDLGSAISFKDDGP
LDQERAIAHTPNSGPDQESSLLSANLVKLVGTITDKDGDSQSASVDLGSAISFKDDGP
LDQERAIAHTPNSGPDQESSLLSANLVKLVGTITDKDGDSQSASVDLGSAISFKDDGP
LDQERAIAHTPNSGPDQESSLLSANLVKLVGTITDKDGDSQSASVDLGSAISFKDDGP
LDQERAIAHTPNSGPDQESSLLSANLVKLVGTITDKDGDSQSASVDLGSAISFKDDGP
LDQERAIAHTPNSGPDQESSLLSANLVKLVGTITDKDGDSQSASVDLGSAISFKDDGP
LDQERAIAHTPNSGPDQESSLLSANLVKLVGTITDKDGDSQSASVDLGSAISFKDDGP
LDQVRAIAHTPNSGPDQESSLLSANLVKLVGTITDKDGDSQSASVDLGSAISFKDDGP
LDQERAIAHTPNSGPDQESSLLSANLVKLVGTITDKDGDSQSASVDLGSAISFKDDGP
LDQERAIAHTPNSGPDQESSLLSANLVKLVGTITDKDGDSQSASVDLGSAISFKDDGP
LDQVRAIAHTPNSGPDQESSLLSANLVKLVGTITDKDGDSQSASVDLGSAISFKDDGP
LDQVRAIAHTPNSGPDQESSLLSANLVKLVGTITDKDGDSQSASVDLGSAISFKDDGP

A7 repeat
SASAKAAASAGATLDETGGFDAVDITSSAISGLFNTPVYGTDGAGSVQYSLSATAGAGTGLWLAGQSGSASEIKLVAVANGYEGWTGGSTSGTKAFSVLM
DGNGKVTVTQYAALEHGVDGSSASDHDDSVSLAAAAAIKVVQTVADRDGDSTTATSASGLDIAFKDDGP

SAVADTITSGTNGTVNLVLVLDSSGSIGNTNMQVIKDAVTNLMNSYGNSLVKVMLVDFDSSATVKSVGSQVWLTKDQVTGQLTSISSGGSTDYDDALQAV
RDNYGTPPSADNTFVFFISDGVPSSADEAINATERNDWTNFLTQKGIDGAYAVGIGSTSVLDADLQSVAWSPSGAHNNNVVVINTAGQLSGTLTNLTQVI
DGNVTTNDSAGADGFATPKLVSVEYNGTTYTFNNSNTSFSIALGTNKGTLYIENDGDYRFTPPSGGAEGAPVEITYKIKDGDGDTSSAKLTIINPVLVVG
SNANDTGSGASTAIDDHARPNPLLAPDVDGAIVGGVGADVLIGDVGGVTSGSYNLTFMIDMSGSISGTEFQLMKDAINNLLAKFSGISQLQVEIGTFADN
SNVVGTYSSVTAAQQAVSNLTRSGGGTNYQAALTTLNTMMTGDPVADHKYVYFLTDGEPTVGSWTNSTQIANGMAALNALTAPGVVINAVGIGVPSGASF
GNNLNAIDNTPDNYLAVDSFDDLSSGLGSLFTAVSVGSDLLQGGAGNDVIFGDSIHADNVDGGWFEFVADNPGKSGGQLLNELYSQHAVYGKEGAVGGDD
TLEGGDGNDVLYGQKGNDILIGGDGIDILIGGTGSDTLTGGAGKDTFKWGAGDVGGTDVIKDFTTGTGGDVLDISELLTGEHANKNSLDAYLTFSSGPGS
GKSTLTIDLDGSGGNSTTHVIRFDSIDLTLGGTRNDQTIIEDLLNQGNLKVDP

Aer-41 WP_158512870.1
MRTQIIDKSVVVSAIEGNVEIVLADGSSRPLQKGEILQPGAKLAIADDAKLALSPYDDTPASDVPAGSESAEPAQPQVAASQEGAPSDIAALQESILQGV
DPTQNFEASAAGGAPAAGGGGGIGGVAGASGNGGFVTIDRTGDATIAAAGFDTTYDTAAVINENQAEDLILENQLDDLGETAETQEDTQVSGNLLDNSTN
PDGPVDASIVSYDWGINIRVPAGVAATITGVGTLIINPDGSYTFTPAPNYDGAVPPVNYQVTDGQDTVTSTLVISITPVDEEVSLDGLTQEGEEVLVSDA
NLADGSAPNDAALTQSGVFTFNAQDGVQSLALGNVTLITNGQSATSFPLSIISPLGNVLTITAVTYNPVTGAGQVSYSYTLGDNENHTRPANDTSLSESF
SVVLTDSDGDTASDTLDITILDDIP

A5 repeats
TLAG--DIRSTLGGQVQEDALTSGNSEGTGQSASASGAAGTLNNLVNFGADGAGSFGLGSDVSSLTAQGLTSGGVALSYSVVGNVLTASAGSVPVFTLTV
VLAGGEDERPQVGGQVQEDALTSGNSEGTGQSASASGAAGTLNNLVNFGADGAGSFGLGSDVSSLTAQGLTSGGVALSYSVVGNVLTASAGSVPVFTLTV
VLAGGEDERPQVGGQVQEDALTSGNSEGTGQSASASGAAGTLNNLVNFGADGAGSFGLGSDVSSLTAQGLTSGGVALSYSVVGNVLTASAGSVPVFTLTV
VLAGGEDERPQVGGQVQEDALTSGNSEGTGQSASASGAAGTLNNLVNFGADGAGSFGLGSDVSSLVGQGLTSGGVALSYSVVGNVLTASAGSVPVFTLTV
VLAGGEDERPQVGGQVQEDALTSGNSEGTGQSASASGAAGTLNNLVNFGADGAGSFGLGSDVSSLVGQGLTSGGVALSYSVAGNVLTASAGSVPVFTLTV
VLAGGEDERPQVGGQVQEDALTSGNSEGTGQSASASGAAGTLNNLVNFGADGAGSFGLGSDVSSLTAQGLTSGGVALSYSVVGNVLTASAGSVSVFTLTV

GVDGSYSFTLSGPLDHPVADGDDSEQLAGVGIDFSGVLTATDGDGDPLTGSFPAGSFAIRVEDDVP
GVDGSYSFTLSGPLDHPVADGDDSEQLAGVGIDFSGVLTATDGDGDPLTGSFPAGSFAIRVEDDVP
GVDGSYSFTLSGPLDHPVADGDDSEQLAGVGIDFSGVLTATDGDGDPLTGSFPAGSFAIRVEDDVP
GVDGSYSFTLSGPLDHPVADGDDSEQLAGVGIDFSGVLTATDGDGDPLTGSFPAGSFAIRVEDDVP
GVDGSYSFTLSGPLDHPTADGDDGEQLAGVGIDFSGVLTATDGDGDPLTGSFPAGSFAIRVEDDVP
GADGSYSFTLSGPLDHPVADGDDSEQLAGVGIDFSGVLTATDGDGDPLTGGFPAGSFAIKVEDDVP

A3 repeat
RVVLADDAPRIGAMVDESLASLDGVGSDGVASATLSAADVQAQFAAAFGADGKGSIGYSLALTGSNVASGLYAVDPQAANGQGAAIVLNQVGNVITGSV
GAVSYFTLTINPTTGEVTLALLGNVWHGNTSSADDSVALSVGKGVLTLVQTVTDADGDRASASVDLGANDVFRFEDDGP

A6 repeats
SISAGSAASDSLQVDESNLSLNASTSFASLFTSSFGADGAGTLVYSLNVSAAGADSGLNDTATGQDILLYLESGSVVGRVGGSSGSVAFTLSVNSSGEVT
SISAGSAANDSLQVDESNLSLNASTSFASLFTSSFGADGAGTLVYSLNVSAAGADSGLNDTATGQDILLYLESGSVVGRVGGSSGSVAFTLSVNSSGEVT
SISAGSAANDSLQVDESNLSLNASTSFASLFTSSFGADGAGTLVYSLNVSAAGADSGLNDTATGQDILLYLESGSVVGRVGGSSGSVAFTLSVNSSGEVT

LDQVRAIAHTPNSGPDQESSLLSANLVQLVGTVTDKDGDSQSASVDLGSAISFKDDGP
LDQVRAIAHTPNSGPDQESSLLSANLVQLVGTVTDKDGDSQSASVDLGSAISFKDDGP
LDQVRAIAHTPNSGPDQESSLLSANLVQLVGTITDKDGDSQSASVDLGSAISFKDDGP

A7 repeat
SASAKAAASAGATLDETGGFDSVDITSSAISGLFNTPEYGADGSGSVQYSLSATAGAGTGLWLEGQSGSASEIKLVAVANGYEGWTGGSTSGTKAFSVRI
DSSGKVTVTQYAALEHGVDGSSASDHDDSVSLAAAAAIKVVQTVTDRDGDSTTATSASGLNIAFKDDGP

SAVADTITSGTNGTVNLVLVLDSSGSIGNTNMQVIKDAVTNLMNSYGNSLVKVMLVDFDSSATVKSVGSQVWLTKDQVTGQLTSISSGGSTDYDDALQAV
RDNYGTPPSADNTFVFFISDGVPSSADEAINATERNDWTNFLTQKGIDGAYAVGIGSTSVLDADLQSVAWSPSGAHNNNVVVINTAGQLSGTLTNLTQVI
DGNVTANDSAGADGFGTPKLVSVEYNGTTYTFNNSNTSFSIALGTNKGTLYIENDGDYRFTPPSGGAEGAPVEITYKIKDGDGDTSSAKLTIINPVLVVG
SNANDTGSGASTAIDDHVRPNPLLAPDVDGAIVGGVGADVLIGDVGGVTSGSYNLTFMIDMSGSISGTEFQLMKDAINNLLAKFSGISQLQVEIGTFADN
SNVVGTYSSVTAAQQAVSNLTRSGGGTNYQAALTTLNTMMTGDPVADHKYVYFLTDGEPTVGSWTNSTQIADGMAALNALTAPGVVINAVGIGVPSGASF
GNNLNAIDNTPDNYLAVDSFDDLSSGLGSLFTAVSVGSDLLQGGAGNDVIFGDSIHADNVDGGWFEFVADNPGKSGGQLLNELYSQHAVYGKEGAVGGDD
TLEGGDGNDVLYGQKGNDILIGGDGIDILIGGTGSDTLTGGAGKDTFKWGAGDVGGTDVIKDFTTGTGGDVLDISELLTGEHANKNSLDAYLTFSSGPGS
GKSTLTIDLDGSGGSSTTHVIRFDSIDLTLGGTRNDQTIIEDLLNQGNLKVDP

AER-43 WP_158110690.1
MRTQIIDKSVVVSAIEGNVEIVLADGSSRPLQKGEILQPGAKLTIADDAKLALSPYDDTPASDVPAGSESAEPAQPQVAASQEGAPSDIAALQESILQGV
DPTQNFEASAAGGAPAAGGGGGIGGVAGASGNGGFVTIDRTGDATIAAAGFDTTYDTAAVINENQAEDLILENQLDDLGETAVTLEDTQVSGNLLDNSTN
PDGPADASIVSYDWGINIGVPAGVAATITGVGTLIINPDGSYSFTPAPNYDGAVPPVNYQVTDGQDTVTSTLVISITPVDEAVGLDGLTLEGGEVLVSDA
NLADGLAPNAAALTQSGVFTFNAPDGVQSLALGNVTLITNGQSATSFPLSIISPLGNVLTITAVTYNPVTGAGQVSYSYTLGDNENHTRPANDTSLSESF
SVVLTDSDGDTASDTLDITILDDIP

A5 repeats
TLAG--DIGSTLGGQVQEDALTSGNSEGTGQSASASGAAGTLNNLVNFGADGVGSFGLGSDVSSLTAQGLTSGGVALSYSVVGNVLTASAGSVPVFTLTV
VLAGGEDERPQVGGQVQEDALTSGNSEGTGQSASASGAAGTLNNLVNFGADGVGSFGLGSDVSSLTAQGLTSGGVALSYSVVGNVLTASAGSVPVFTLTV

GVDGSYSFTLSGQLDHPVADGDDSEQLAGVGIDFSGVLTATDGDGDPLTGGFPVGSFAIKVEDDVP
GVDGSYSFTLSGQLDHPVADGDDSEQLAGVGIDFSGVLTATDGDGDPLTGGFPVGSFAIKVEDDVP

A3 repeat
RVVLADDAPRLGVMVDESLASLGGVGGDGVASATLSGANVQAQFNPAFGADGQGSIGYSPALTGSNVASGLYAVDPLAVNGQGAAIVLNQVGNVITGSVG
AVSYFTLTINPATGEVTLALLDNVWHGNTSSADDSVALSVGKGVLTLVQTVTDADGDSARASVDLGANGVFRFEDDGP

A12 repeat
KASNVQAALVLDDEGLVGGINGGSGDVAGANTSVTGNLAYQAGADGLKSLELSGPTALGSESVTSTWDAQSGTLSISSTRGVLMTVTITDPSTGAYSVKL
LQPLMHTGVDTEDNLTLNVGYKVTDGDGDSASGVLAVTINDDTP

A13 repeat
TIQVGELSLTSSVTFLGSNAGYSNSYGYYIKGDDGTPLSGKVIWANVHSQSVGSQTDISSLDPAHTGFFVIPNGGANPGLVDGAAVTFQFINGKWQAFVG
SAALSGADGANIVFSDASLNPGGSHLQDTGSAGNQNWEDMTLSSDYDYNDVSTTVTWGGAVQLQVDESNLNHDVTADFSGVFNVQPGADGLGFQSYSLNV
QNANSGLIDTATGEQVQLRMNGSILEGRTATTGQLVFSLTVDSSGKVTLDQPRAVVHPTSDPDEATFLGSGHVNLTLTVTDKDGDHASGVLDIGKVISFK
DDGP

A6 repeat
SISANAAVQLDDDALVGGITGGTGDDPNSVKVSGTLGHSFGADGAGSIQWLTSGAPGGYTYEAGSGGSLLVKQAGVTVLTVTLNSATGAYSVTQNAPIKH
TVSANAAVQLDDDALVGGIAGGTGDDPNAVNVSGTLGHSFGADGAGSIQWLTSGAPGGYTYEAGSGGSLLVKQAGVTVLTVTLNSATGAYSVTQNAPIKH
TVSANAAVQLDDDALAGGIAGGTGDDPNAVNVSGILGHSFGADGAGSVQWLTTGAPAGFTYETNPAGALLVKQGATTVLTVTLNSATGAYSVTQNSPIQH

ADGNDENNATFTLNYRVTDADKDSVDGSLTINVDDDTP
ADGNDENNATFTLNYRVTDADKDSVDGSLTINVDDDIP
ANADQENNQAFTLNYKVNDGDGDTATGTLAINVDDDTP

VAKNDTAIVAESSGKDFNVAFVLDSSGSINGTEFATMMAAVRAAGQALFNGTDGTVQITIVAFSTGATSYTAISSLAAFESLIDSILANRPFNGNTNFTA
AVEQTMDVYNPIPGWSNQVFFISDGNPNVDTGVNNSLESGTATDWNNFIDSNGINVTTIGVGGGINGPRLQDVDLDGSGSPILVTDFDDLIDALLTQISG
NTVSGNVLLGNNNVVGGGDDDAFGADGAGRVLSIEIDGVTYTWNGVGTIAVSTGGSIAGSQLSNIVTDLGGKLSFNFTNGTWSYVAPQSVASDTSESFDY
VIIDMDGDPSQATLTVNIEDAAPVIGRVDEDELSGGITDNDLQTTVATGSLTELLIGNGPATFSLQSSPAFLPTLTSDGVAVTYNVVGNTLTAMAGAATI
FTLVVQANGAYTFTLSGPLDHPLANGNGNDNEILTLNLTEAVKATNGGNPVPLAGDLLIQVEDDVPAILATSNLVYSNSGNPGGATGVFDYSTGADTRGS
GPFSAADSDFKSIMLTGNVGGTAISAQSVTWVSESATTATFDIEFDYAPNPANPGVKEEATGTLTFDKVNGTYTVTLDGPIEGFQVLKTSASLGITGYVP
GGSSVDTTNPEVAVSQLATDFFVQFRSYAETGGGTGSNNLQTGGTNTNVFVNGETFTQTSSYVSLSNSENGVAGDTIGKGEVLDLNFYTSNPTGNLGLTP
TGRADGVYLKFDGINNEDLVIVLKLVGAGGVTTTRALVVSNSDIYRNFSGATALAALAVYGITLDNNDGAIVIESNDYNAAGESWQIYGAQILTSVEDIT
TSVALNYDRDFNDGFDGLTSFANQSDNDVVKVSDIGFIKTQTTTVDADLDFQVVVQDADNDATSSVNLHVTIEAGNVFTGTAMADIIHGSADNDTLKGLG
GDDQLIGGLGNDILIGGLGSDTLVGGSGSDTFKWGAGDIGGVDVIKDFTTGTGGDVLDISELLTGEHANSGSLDAYLNFSSDGPGTNKSTLTIDLDGTNG
GTTHVIKFDSIDLTTLGNSDLQIIQKLLDDGNLKVDP

Aer-45 WP_126623349.1
MDMRTQIIDKTVVVSSVEGNVQILLADGSSRPLQPGEILQPGAKLNIADDAKLMLAPYDDNPGQAAAAPAETDIPAEGQPSSSAENSNLPPEIAALQQSILQ
GVDPTKSFEASAAGGAPAAGGGGGIGGVAGASGNGGFVTIERIGDATIAAAGFDTSHDALTVQDLLQEDDLLPENQLDDQDEPAVTQENQAISGDLLLNST
NPDGPFAASIVSYDWGANLGIPAGVAVTLVGIGTLIINADGTFTFTPTFNYTGPVPPVNYTVTDGEDTDTSTLAITITPLDEPVSLGGLQVEGGELVLNEA
SLVDGSAPNAAALTQGGTFTFNAADGVQSLTLGGVTLISNGQVIGGFPQAILGPLGNQLLVTGISYNPVTGTGSVTYSYTLSDNEAHTQPANDTALTESFN
VVLTDSDGDSTSASLDAVILDDVP

A3 repeat
RVALVPGSGELGVLSVDESVPQLGGVGGDGIASATLTAALVQAQFSHAFGADGAGSIGYSLALSGNNVASGLYAVDPQAANGQGAQIVLNQVGNVITGSANG
VNYFTLTINPATGAVTLNLLDNVWHGNTGNSDDSISLTLNSGVLTLVQTVTDTDGDSAKASLDLGVNGTFRFEDDGP

A8 repeats
TLTVEANGEAVAALAVTLDETTGAADHYANGEGADGYVNDDAPGALARVTTQVVGGLVSLFNVGGSYGSDGAGTLSGTMSFTGVPAGGLSTNLSATDGGA
TLTVEANGEAAAALAVTLDETTGAADHYANGEGADGYVNDDAPGALARVTTQVVGGLVSLFNVGGSYGSDGAGTLSGTMSFTGVPLGGLSTNLSATDGGA
TLTVEANGEAAAALAVTLDETTGAADHYANGEGADGYVNDDAPGALARVTTQVVGGLVSLFNVGGSYGSDGAGTLSGTMSFTGVPLGGLSTNLSATDGGA
TLTVEANGEAVAALAVTLDETTGAADHYANGEGADGYVNDDAPGALARVTTQVVGGLVSLFNVGGSYGSDGAGTLSGTMSFTGVPLGGLSTNLSATDGGA
TLTVEANGEAVAALAVTLDETTGAADHYANGEGADGYVNDDAPGALARVTTQVVGGLVSLFSVGGSYGSDGASTLSGTMSFTGVPLGGLSTNLSATDGGA

ITLMGAGNGTLNGVDGDGNIVFSIQIVDVGGGVLQLQTTQFEALAHGSDGLFDEALSLLLNQGSLALQYEVIRTDADGDPVRVSDSIVLADGERSVFSFDDDGP
ITLVAVGSGTLNGVDGDGNIVFSIQIVDVGGGVLQLQTTQFEALAHGSDGLFDEALSLLLNQGSLALQYEVIRTDADGDPVRVLDSIVLADGERSVFSFDDDGP
ITLVAVGSGTLNGVDGDGNIVFSIQIVDVGGGVLQLQTTQFEALAHGSDGLFDEALSLLLNQGSLALQYEVIRTDADGDPVRVLDSIVLADGERSVFSFDDDGP
ITLVAVGSGTLNGVDGDGNIVFSIQIVDVGGGVLQLQTTQFEALAHGSDGLFDEALSLLLNQGSLALQYEVIRTDADGDPVRVSDSIVLADGERSVFSFDDDGP
ITLVAVGSGTLNGVDGDGNIVFSIQIVDVGGGVLQLQTTQFEALAHGSDGLFDEALSLLLGQGSLALQYEVIRTDADGDPVRVSDSIVLADGERSVFSFDDDGP

A3 repeat
TVGLAVEAPGLGTVMVDESLPALGGVGGDGIASATLAAATVQAQFSHSFGSDGAGSISYSLALSGSNVASGLYAVDPQATNGQGAQILLNQAFNIITGSANG
VDYFTFTIDPISGAVTLNLLDNVWHGNTSSADDSVSLTLSNGVLTLVQTVTDADGDSAEASLDLGANGTFRFEDDGP

A9 repeats
IATDEAAQSVAEGTTKLGAFDFVPGADGAGVSHINGIALTFGGDGYSQVVDVGNGVLKVKADGSYSYTADASVDTTNPVTDNLTFTVTDGDGDAVTKAAS
SATDEAAQSVAEGTTKLGAFDFVPGADGAGVSHINGIALTFGGDGYSQVVDVGNGVLKVKADGSYSYTADASVDTTNPVTDNLTFTVTDGDGDAVTKAAS

FNITDANVPTGGSTTAAVDDDGLTGGNPASTTGDLVVAPDPDTNEATFSGTLTFGFGPDGAGSVNFAAMHGTSGTVGQETVNYSWNGANNTLTATGPRGP
FNITDANVPTGGSTTAAVDDDGLTGGNPASTTGDLVVAPDPDTNEATFSGTLTFGFGPDGAGSVNFAAMHGTSGTVGQETVNYSWNGANNTLTATGPRGP

LFSVVVNPSTGAYTVTLLDNVQHATLDGQPGDNTENDAVTALTYTVKDADNSTTTGTLTITFDDDMP
LFSVVVNPSTGAYTVTLLDNVQHATLDGQPGDNTENDAVTALTYTVKDADNSTTTGTLTITFDDDMP

A6 repeat
SVGSNLTVQLDDDALANGIPDGPNDDDNAVNTNGILAHNFGADGAGIVQWLTTGAPAGFTYETNAAGSLLVKQGATTVLTVTLNSTTGAYSVTQNAPIQH
ANADQENNQAFTLNYQVTDGDNDTANGTLAINVDDDTP

LAKNDTATVAESSDKDFNVAFVLDSSGSISNSEFTTMMNAVKAAGQALFNGTDGDVKITIVAFSSDPLAYASVTSIAAFNALVDSIIANRPFDDGTDFTA
GIQQTMVSYSPIEGWSNQVFFISDGDPNEQLGTGGNSLSDATATAWNTFVDSNAINVTTIGVGDGINTARLQDVDLDGSGAPILVTDFSGLINTLLGQIS
GNFVSGNVLLGNNNAVGGGDDDSFGADGPGRIMSIKINGTTYAWDGVMDGDQQLTNITTALGGKLTFNFATGAWGYTSPQSVAADTSESFDYVIVDKDGD
QASATLTINIEDAAPVIGKVDEDELPGGITDGDAQTTIISGSLVELLVGTSSGQFSLDTTPNVMPSLTSDGVAVTYGFSGSTLTAMAGATTVFTLVVQSN
GSYTFTLLGPLDHPNANGDDNEILTLNLTGAIRASDGVNPLPLAGDLLIQVEDDVPTIQASTNLVYSNSSNPGGGTGVFDYSTGADNRGTGPFSSSDSDF
NAIGLTGTVGGSAITSQLVTWVSENASTATFDIEFNYAPNPASPGITQEATGTLIFDKINGTYTVSLDEPIEGFSVLKTSASLSITGYASGGSVPDNSQP
DVSVAKLDNDFYVQFRSAAEPGGGTGTNNLQTGGVNTNIFANGELFTQAASWVSVSNSANGVAGDTIGKGEVLDLTFHTTNPTGTVVANPDGRAEGIFLK
FDGINNEDLVVVLKLIGTGGITTTRALVISNSDIARSGSSAATLAALLAYGITLDNNDGAIVIEHNDYNGVGEDWQIYGAQILTSVEDITTTTALNFNAA
FGDTGGSNSTTNFTGQTDSDVVKVSDIGLITSENTTLDAELDFQVGVKDADNDAASNTNLHVTIEAGTTFTGSALADVIQGGEGNDTLSGLAGDDTLIGG
LGNDLLIGGTGSDTLTGGAGKDTFKWQAGDAGGTDTIKDFTPGNGGDVLDLSEMLSGEHANAATLDQYLTFASGPGTNKSTLTIDLDGSAGAGTTTHTIS
FDNVDLTAGKSNLQIIEDLLNQGNLKVDP

Pse-4 WP_009044311.1
MTTPQSPTTDLTADTPPDLYPVFPSNDTLTITALADGAPLVVATGTNVALDETSGLQNATATPDPTGDADDNDILLAALPSAFATRLTALGAGTATDAAL
SGYTGALGDTGSNAFTLNLAPGAINVDISFTDSFGAALNGLDSGLDTLDGIDILLYTDTDNNILLGRAGGPDGAIVFAAYIEETGSPLSGGKIWTVEYQP
LKHPDGSNPDDALNLLDKVFIGASQDLTFSLADAPSGQNLFLMFTKANPTVVDDGGVLRISDPTIIATGKDPADQSSGASINTGDTINTSQAGGPTTFGT
NSQMITEQEGIRFTFVTGARQDMTIPNLDQNEADLEANIDFTAMFNAKAANFDIVQLQGGKSAVVRISAFNTAVESGASFVNGYANDTSVAITNVRVINI
ATGLVIENSDGSVNDTSILISFDGGVATITGVKAGYQIEYTTAADHNRVLIENGAALDAKGNTHADFDIGGFTLRQASTTTAEIGSQMIFEDDGP

TLAFGNLIGTGTQLAQQGYWNMGSGADGLDANGLDISLVNDQFTLVRPDNTTTTGTGTLVEQSPSPDGNGAYQFAGTLTGDFDNNAATADTTVHYTLSAY
ANGTYALDLEEGFRSTIVLSSADGSLAAGGPDPVRTLMIGSEDVVFFAANPLAPQTGDNSILTGIGLGAPDPTEAQLQTNPLPSFIGSSTMNVSTSGIGV
ANNNLEGNTTAGINAGDESFVINPETLLTSMKIFIDNSVQGYNPATEELYYTIYYDNGTTSGTPIKVQAADLHTETGGQKSFTVQWDGSHLIDAVQLTMG
LGTVKIPVIQFIKETQSLANDIQLSFNATTTDKDGDTATSAFDANLFANDPSDQTFDFRLVGTTGERDAFNIDLSVAENQYQVTGFDAGPGQRDAVVLIG
DAGATVQSIDNASNDSIVTIAETGGQLTTITLLGVDLLNTDIVMASV

Pse-5 WP_009044312.1
MTTLQENLSLTSDPLVVATGVNVALDETTGLQNATTTPAPAGDADDNDILLAALPSAFATRLTALGAGTATDAALSGYTGAVGDTGSNAFTLNLAPDAIN
VDVGFTDSLGAPLDGLDSGLDTLDGIDILLYTDTDNNILLGRAGGPDGAIVFAAYIEETGSPLSGGKIWTVEYQPLKHPDGSNPDDALNLLDKVFIGASQ
DLTFSLANAPSGQNLFLMFTKANPTVVDDGGVLRISDPTIIATGKDPADESTGVNINTGDTINTSQAGGPTTFGTNNQMIVEQEGIRFTFVTGARQDVTV
PNLDQNEADVEANIDYTAVFNATTASFDVVQLQGGKSAVVKVSAFSTAVETGADFVNGYADDTSVAITNVQVIDNATGLVIENSDGSVNDTSILISFDGG
VATITGVKAGYQIEYTTSANHNRVLVENGAAVDAKGNDHADFDIGAFTLRQATTATAEIGSKMIFEDDGP

P1 repeats
AAAGTAEAGTVDEDGLANGIAGGVGDVPGELTTAGGNVSGIFQSGVDVPLTYSLSSDTSGLPALTSGGVALVYSVVGDTLTAKAGAVDVFTFSLSAAGAY
TANGTAGAGTVDEDGLANGIAGGVGDVPGELTTASGNVTGIFQSGADVPLTYALSSDTSGLPALSSGAVALVYSVAGGTLTAKAGAVDVFTFSLNAAGDY
TASGTAAAGTVDEDGLANGIASGVGDVAGEATTAIGSVTGIFQSGADVPLSYSLSSDTSGLPALSSGGVALVYSVAGDTLTAKAGAVDVFTFSLSAAGDY
TANGTAEAGTVDEDGLANGIAGGVGDVAGESTTANGSVTGIFQSGADVPLTYALSSDTSGLPALSSGGVALTYSVTGDTLTAKAGAVNVFTFSLNAVGAY

SFTLLQPLDHPAGNDENDITINLGSLLQATDKDGDTVTAAAEKLVITVDDDTP
SFTLLQPLDHPAGNDENDITINLGTLLQATDKDGDTVTAAAEKLVITVDDDTP
SFTLLQPLDHPAGNDENDITLNLGTLLQATDKDGDTVTAAAEKLVITVDDDTP
AFTLLQPLDHAAGNDENDITINLGSLLQATDKDGDTVTAAADKLVITVDDDTP

TLAFGNLIGTGTQLAQQGYWNLGSGADGLDTNGLDISLVNDQFTLVRPDDTTTTGTGTLVEQSPSPDVNGAYQFAGTLTGDFDNNAATADTTIHYTLSAY
ANGTYALDLEEGFRSTVVLSSADGSLDAGGPDPVRTLTIGTEDVVFFGANPLAPQTGDNSILTGIGLGAPDPTEAQLQTNPLPSFIGSAAMNVSTSGIGI
ANNNLEGNNTAGINAGDESFVVNPETLLTAMKVYIDNSVQGYNPATEELYYTIYYDNGTTSGAPIKVQAADLQAEAGGQTSFTVEWDGTHLIDAVQLTMG
KGTIKVPTIEFIHEVQSLASDIQLAFNATVTDKDGDTATSAFDANLFANDPANALFDFRLMGTTGERDAFNIDLAATENQYQVTGFDAGAGQRDALVLIG
DAGAVVQSVDNAGSDSIVTVAETGGQITTITLVGVDLLNTDIVMGSV

Pse-6 WP_152032113.1
MTLSTAQANGTAPVVLGASVTLDETTGLQNATTTPSPAGDADDNDILLSALPSTFATRLTALGAGTAMD
AALSGYTGAVGDTGSNAFTLNLTPGTNVLDIKFTDSLGAALNGLDSGLDTLDGTSILLYTDTNNNILLGRAGGPDGAIVFAAYIEETGSPVSGGKIWTVE
YQPLKHPDASNPDDALSLLDKVFVSTLKDGFDLTNAPSGQNLFLMFTGSNPTVVDDGGVLRVSDPTIIATGKDPADQSSGANINTGDTVNTSQGGGPTTI
GTNNQMIVEQEGIRLTFVTGARLDVTVPNLSQNEADVEANIDYTAMFNATTASFDVVQLQGGKTAMVRISAYSTAVESGVNFVNGYANDTSVAITNVRIF
DKSTGLVIENSDGSVNDPNIAISFTGGVATITGVKAGYQIEYTTSADHNRVLIENGAAVNARGDNHADFDIGGFSLRHPSTITSEIGSQMIFEDDGP

P1 repeats
AAAGTAAAGTVDEDGLANGIPGGVGDVPGEVTSVSGNVSAIFQSGVDVPLTYSLSSDTSGLPALTSGGVALVYSVVGDTLTAKAGA--VDVFTFSLNAAG
TATGVAAAGTVDEDGLANGIPGGVGDVPGEVTTASGNVTGIFQSGADVPLTYSLSSDTSGLPALTSGGVALVYSVVGDTLTAKAGA--VDVFTFSLNAAG
TAAGTAEAGTVDEDGLANGIAGGVGDVAGEATTASGNVTGIFQSGADVPLTYSLSSDTSGLPALTSGGVALVYSVAGDTLTAKAGA--VDVFTFSLSAAG
TANGTAGAGTVDEDGLANGIAGGVGDVPGEATTASGSVTAIFQSGADIPLTYSLSSDTSGLPALSSGGVALVYSVAGDTLTAKAGA--VDVFTFSLSAAG
TATGTAEAGTVDEDGLANGIAGGIGDVPGEATTASGNVTAIFQSGADVPLTYSLSSDTSGLPALSSGGVALVYSVAGDTLTANAGVGGAEVFTFSFSAAG
TANGTAGAGTVDEDGLANGIAGGVGDVAGEATTASSSVTGIFQSGADVPLTYALSSDTSGLPALSSGGVALVYSVAGDTLTAKAGT--TDVFTFSLSAAG
TATGAAEAGTVDEDGLANGIAGGVGDVAGEATTASGNVTGIFQSGADVPLTYSLSSDTSGLPALSSGGVALVYSVAGDTLTAKAGA--VDVFTFSLNAAG
TANGTAGAGTVDEDGLANGIAGGVGDVPGEATTASGSVTAIFQSGADVPLTYALSSDTSGLPALSSGGVALVYSVAGDTLSAKAGA--VDVFIFSLNAAG
TANGTAGVGTVDEDGLANGIAGGVGDVPGEATTASGSVTGIFQSGADVPLTYALSSDTSGLPALSSGGVALVYSVAGDTLTAKAGA--TDVFTFSLNAAG
TATGTAAAGTVDEDGLANGIAGGVGDVPGEATTASGSVTGIFQSGADVPLTYALSSDTSGLPALSSGGVALVYSVAGDTLTAKAGA--VDVFTFSLNAAG
TASGTAAAGTVDEDGLANGIAGGVGDVAGEATVANGSVSGIFQSGADVPLTYALSSDTSGLPALTSGGVALVYSVAGDTLTAKAGT--TDVFTFSLSAAG
TATGTAAAGTVDEDGLANGIAGGVGDVPGEATTTSGSISGIFQSGADVPLTYALSSDTSGLPALSSGGVALVYSVAGDTLTAKAGA--VDVFTFSLSAAG
TANGSAEAGTVDEDGLANGIAGGIGDVPGEVTTVSGNVSGIFQSGADVPLSYSLSSNTSGLPALSSGGVALTYSVLGSTLTAKAGA--TDVFTLSLTVAG

DYSFTLLQPLDHPAGNDENDITLNLGTLLKATDKDGDSVTAAAEKLVITVDDDTP
DYSFTLLQPLDHPAGNDENDITLSLGAMLQATDKDGDTVTAAAEKLVITVDDDTP
AYSFTLLQPLDHPAGDDENDITLNLGAMLQATDKDGDTVTAAADKLVITVDDDTP
DYSFTLLQPLDHPAGNDENDISLNLGAMLQATDKDGDTVTAAAEKLVITVDDDTP
AYSFTLLQPLDHPAGNDENDITINLGTMLQATDKDGDTVTAAADKLVITIDDDTP
DYSFTLLQPLDHPAGNDENDITLNLGAMLQATDKDGDTVTAAAEKLVITVDDDTP
DYSFTLLQPLDHPAGNDENDITLNLGAMLQATDKDGDTVTAAAEKLVITVDDDTP
DYSFTLLQPLDHPAGNDENDITLNLGAMLQATDKDGDTVTAAADKLVITVDDDTP
DYSFTLLQPLDHPAGNDENDITLNLGAMLQATDKDGDTVTATADKLVITVDDDTP
DYSFTLLQPLDHPAGNDENDISLNLGAMLQATDKDGDTVTAAAEKLVITVDDDTP
AYSFTLLQPLDHPAGNDENDITLNLGTLLQATDKDGDTVTAAAEKLVITVDDDTP
DYSFTLLQPLDHPAGDDENDITLNLGTLLKATDRDGDTVTAAAEKLVITVDDDTP
AYTFSLLQPLDHAAGNDENDLTINLGSLLQATDKDGDTVTAAADKLVITVDDDTP

TLAFGNLIGTGTDQAQQGYWNMAAGADGLGAAGLDISLVNGQFTLVRPDDTTTTGTGTLVEQSPSPDANGAYQFAGTLTGDFDNNAATADTTVHYTLSAY
ANGTYALDLEEGFRSTIVLSSADGSLDAGGPDPVRTLTIGSEDIVFFGANPLAPASGANSIQTGIGLGASDPTEAQLQTNPLPSYIGSAALNVSTAGIGV
ANNLLQGDNQAAIGAADESFVVNPQSLLTSMKVFIDNSVGGYNTATEDLYYRVFYEDGTFSNLIEVNTLTPEAGGQVSFTVEQSATSLIDAVQLTMARGE
IKIPTIEFTHETESLASDVKLVFNATVTDKDGDTATSAFDANLFANDTTDVLFDFRLVGTTGERDAFNIDLSVAENQYQVTGFDAGAGQRDALVLIGDAG
AVVQSIDNAGADSIVTVAETGGQITTITLVGVDLLNTDIVMGSV

Pse-16 KMM95656.1
MAIEITGEDVVLDETPGLQNATATPTPAGDADDNDILVASLPSDFSTRLTALSAGTATGAALSGYTGAVGNTGSDAFTVTNGESITDISFVDSTGAPLDG
ADSGLFTLDGTSILLYTDTNNNILLGRAGSATGAIVFAAYIEETGSPVSGGKIWTVEYQPLQHPDGTNPDDSLNLLDKVFVGTSQDLEFSLAGVPSGANL
FLMFTKANPTVVDVGGVLRITDPTIIATGKDPADESSGANINTGDTIITSQAANPTTFGTNSQMITEQDGIRFSFVTGARQDVTIPNLSPTEADVESNID
FTDVVNAKKASFDVVQLQSGKSAVVKISAFSTAAEPDVNFINGYANDTPVAITNVRVFNSAGVVIENSDGSVNDPTIGISFSGGVATVTGVLAGYQIEYT
TTEDHNRVLVENGAALDARGNTHADFDIGGFTLLQTAVTTAEIGSKIIFEDDGP

ALAFGNLIGTGSVLPQTGFWSNSAGADGLDAAGLDISVNSQFTLVRPNNTTTTGTATLTEVVPSPDGNGAYQFAGTLTGDFDNNADTADTTVDYTLTAFD
DGSYALDLVQGFSSTVVLSTADGSLAAGGPDPVRTLLIPDTNDPTIPSASEEVVFFSAKALASTTDILAGIGLGAPDPTEAELQTTPLPSYIDPRAMNVS
TSGIGVANNVLQGDNLVAIGATDESFVVNPESLVTAMKVFIDNSVAGYNTATEDLYYRIYYEDGTTSNLIEVNTLTPEPRGQVSFLIEQEGSLLIDAVQL
TMGRGDIKIPVIQFIQETESLASDIQLTFNATVTDRDGDSASSTFDANLFANDLAGAFDYTLVGTDDELDAFNVDLSFDENLYQITGFDTDANLRDTLVL
NGDQSAVVQIDTSGADSIVTVTETGGDVTTITLVGVDLLSSDIVLGSA

Pse-17 KMM95655.1
MDETAGLQNATATPTPAGDADDNDILVASLPSSFSTRLTALGAGTATGAALSGYTGAAGDTGSNAFTFTGGGSITDIRFVDSAGAPLNGVDSGLDTLDGT
SILLYTDTDNNILLGRAGGADGAIVFAAYIEETGSPVTGGKIWTVEYQPLKHPDATNPDDSLNLLDKVFIGVSQDLGFSLAGAPSGQNLFLMFTKLNPTT
ETVDGVVRITDPTIIATGKNPADQSSGANINTGDTINTSQGGGPTTIGTNSQMITEQEGIRFSFVTGARQNVTVPNLSQTEADVESNIDFTSVYDATSAS
FDVVQLQSGKSAVVKISAFSTAAEPGVNFINGYTGDAPVAITNVSVINISTGLVIENSDGSVNDPAIGISFSGGVATITGVLAGYQIEYTTTADHNRVLI
ENGAALDARGNDHADFDIGGFTLREASTTIAEIGSKMIFEDDGP

P2 repeats
SISATGTEPTLTVDETVLATDATQNFAANFTSAFGADGPGTLTYALGVVAGASGLTDTATGEAVNLSLNGTVVEGRTATTNLLVFTVSVAANGDVTLDQQ
SITATGEEPTLTVDETVLTTDATQNFAANFSSAFGADGAGTLTYALGVVAGASGLTDTATGEAVNLSLNGTVVEGRTATTNLLVFTVSVAANGDITLDQQ
SITATGEEPTLTVDETVLTTDATQNFAANFSSAFGADGAGTLTYALGVVAGASGLTDTATGEAVNLSLNGTVVEGRTATTGLLVFTVSVAANGDVTLDQQ
SITTTGEEPTLTVDETVLATGATQNFAANFTSAFGADGPGTLTYALGVVAGASGLTDTATGEAVNLSLNGTVVEGRTATTNLLVFTVSVAANGDVTLDQQ
SITTTGEEPTLTVDETVLTTDATQNFAANFTSAFGADGAGTLTYALGVVAGASGLTDTATGEAVNLSLNGTVVEGRTATTNLLVFTVSVAANGDVTLDQS
SISTTGEEPTLTVDETVLATNATQNFAANFSSTFGADGAGTLTYALGVVAGASGLTDTATGEAVNLSLNGGVVQGRTATTNLLVFTVSVAANGDVTLDQS

RAVVHPDATNPDDSTSLTLDDLVTLTATTTDGDGDSVQATLNIGSNLVFKDDGP
RAVVHPDATNPDDSTTLSADNLVTLTATTTDGDGDSVQATLNIGSNLVFKDDGP
RAVVHPDATNPDDSTSLTSDDLVTLTATTTDGDGDSVQATLNIGSNLVFKDDGP
RAVVHPDATNPDDSTSLTSDDLVTLTATTTDGDGDSVQATLNIGSNLVFKDDGP
RAVVHPDATNPDDSTSLSADNLVTLTATTTDGDGDSVQATLNIGSNLVFKDDGP
RAVVHPDATDPDDSTSLSADNLVTLTATKTDGDGDSAQATLNIGSNLVFKDDGP

PALAFGNLIGTGSVLPQTGFWSKSAGADGLSAAGLDISVNSQFTLVRPDNTTTTGTATLTELVPSPDANGAYQFAGTLTGDFDNNAATADTSVDYTLTAY
ADGRYALDLVQGFSSEIVLSTADGALGAGGPDPVRTLLIPEQDPPTIPSPSEEVVFFSAKALASTSDILTGIGLGEPDPTEAQIQTNPLPSYIDPSAMNV
STAGIGVANNLFQGDNLAAIGVDDESFVVNPESLLTGMRVFIDNSVGGYNTATEDLYYRAYYEDGTFSNLIEVNTLTPEAGGQVSFLIESDGTNLIDAVQ
LTMARGEIKIPTIQFIQESESLASDVQLTFNATLTDKDGDSATSTFDANLFANDAVDALFDFSLVGTGGERDAFNIDLSVDENLYQVTGFDANANLRDAL
VLNGDQSAVVQSIDISGADSIVTVAETGGQVTTITLVGVDLLSSDIVYGSV

Pse-20 WP_096481634.1
MTVIAQENVMTIREQNSSLTSDPTSLNLTSITANALSVTPGANVTLDETAGLQNLTATPTPAGDADDNDILLASLPSAFSTRLTALGAGTATGAALSGYT
GAAGDTGSNAFTLTDASGITDIRFVDSAGAPLDGVDSGLDTLDGTSILLYTDTNNNNIVVGRAGGPAGAIVFAGYIEETGSPVTGGKIWTVEYQPLKHPD
ATNPDDSLNLQDKVFVGVTQDLGFSLAGAPSGQNLFLMFTKANPTTETVNGVVRITDPTIIATGKDPADQSSGANINTGDTINTSQGGGPTTIGTNNQMI
VEQEGIRFSFVTGARQDVTVPNLSQTEADVESNIDFTGVYNSTSANFDVVQLQSGKSAVVKISAFSTAAEPGANFVNGYANDTPVAITNVQVINNSTGLV
IENSDGSVNDPTITISFTGGVATLTGIKAGYQIEYTTTSDHNRVLIENGAALDARGDNHADFDIGGFSLRQASTSTAEIGSKMIFEDDGP

P2 repeats
SVSATGTEPTLTVDETVLTTDATQNFAANFSSAFGADGAGTLTYALGVVAGGSGLTDTATGEAVNLSLNGTVVEGRTATTNLLVFTVSVAANGDVTLDQQ
SISTTGTEPVLTVDETVLTTDATQNFAANFTSAFGADGAGTLTYALGVVAGASGLTDTATGEAVNLSLNGAVVEGRTATTNLLVFTVSVAANGDVTLDQQ
SISTTGTEPVLTVDETVLTTDATQNFAANFTSAFGADGAGTLTYALDVVAGASGLTDTATGEAVNLSLNGAVVEGRTATTNLLVFTVSVAANGDVTLDQQ
SISTTGTEPVLTVDETVLTTDATQNFAANFSSAFGADGAGTLTYALGVTAGPSGLTDTATGEAVNLSLNGTVVEGRTATTNLLVFTVSVAANGDVTLDQQ
SISTTGTEPVLTVDETVLTTDATQSFAANFSSAFGADGAGTLTYALGVVAGGSGLTDTATGEAVNLSLNGAVVEGRTATTNLLVFTVSVAANGDVTLDQQ
SISTTGTEPVLTVDETVLATNDTQSFTANFSSAFGADGAGTLTYALGVVAGGSGLTDTATGQAVNLSLNGTVVEGRTAISNDLVFTVSVAANGDVTLDQL
SISTTGTEPTLTVDETVLTTDDTQNFAANFTSAFGADGAGTLTYALGVVAGGSGLTDTATGEAVNLSLNGSVVEGRTAITNLLVFTVSVAANGDVTLDQQ

RAVVHPDATNPDDSTSLTSDNLVTLTATITDKDGDSAHATLNIGQNLVFKDDGP
RAVVHPDATNPDDSTSLTSDNLVTLTATITDKDGDSAHATLNIGQNLVFKDDGP
RAVVHPDATNPDDSTSLTSDDLVTLKATITDKDGDSAQATLNIGQNLVFKDDGP
RAVVHPDATNPDDSTSLTSDNLVTLTATITDKDGDSAHATLNIGQNLVFKDDGP
RAVVHPDATNPDDSTSLTSDNLVTLTATITDKDGDSAHATLNIGQNLVFKDDGP
RAIVHPDPTNPDDSKTLTSDNLVTLTATITDKDGDSAHATLNIGQNLVFKDDGP
RAVVHPDPTNPNDSTSLSSDNLVTLTATITDKDGDSAHATLNIGQNLVFQDDGP

TLAFGNLIGTGSILPQQGYWDHSAGADGLGATGLDISLVNNQFTLVRPDNTTTTGTGTLTEQSPSPDVNGAFHFAGTLTGDFDNNAATANTTVDYTLTAF
ANGTYALDLVQGFSSQIVLSSADGSLSAGGPDPVRTLLIPPQNPPTIPSPSEEIVFFSAKATASTADILTGIGLGAPDPTEAQLQTNPLPSYIDPAAMNV
STAGIGIANNLFQGDNLAAISNADESFVINPESLLTSVKVFIDNSVGGYNTATEDLYYRAYYDDGTFSNLTEVNTLTPEAGGQVSFLIEQQGTKLIDAVQ
LTMGRGEIKIPTIEFTHETESLASDVKLAFNATLTDKDGDSATSTFDANLFANDLGTTFDFTLVGTSGERDAFNVDLSFTQNKYQVTGFDASAGLRDTLV
LNGDQNAVVQSIDNSGADSIVTVAETGGQITTITLVGVDVLNTDIVHGSA

Pse-21 WP_096480329.1
MTISTLSVVSTGVDVTLDETTGLQNATATSSPAEDANDNDTATPLPSAFSTRLTALGVGTAMDAALSGYTGAVGNTGSDAFTISGATSATDVSFVGSNGA
PLNGVDSGLDTLDGTSILLYTDTVNNNILVGRAGAGGAIVFAAYIEETGSPVTGGKIWTVEYQPLSNPDATDPDDPVDLSGVVFIGASQNLEFSLQNAPS
GQNLFLMFTTATPATETVNGVVRITDPAVIGTGKLPANQSGVDPNSTADDINITTGDTINTSQAGGPTTFGTNNQMITEQEGIRFSFVSGARQNVTIPNL
DQNEADLESNIDFTGVVNVKTVSFDVVQLQSGKSAVVKISAFSTAVESGTQFIDGYANDTKVDIVSVRVINLSTGQVIENSNGPENDPAITISFAAGVAT
LTGIKAGYQIEYTTTLDHNRVLIENGAALDAKGNTHADFDIDGLHLLQASITKAEVGSQMIFEDDGP

P1 repeats
AAAGTAVTGTVDEDGLTNGIAGGVGDVTGEATTATGSVTGIFQSGVDTPLTYALSSDTSGLAALSSGGVALVYSVLGNTLTAKAGTT--DVFTFSLTAAG
TATGTAVTGTVDEDGLTNGIAGGVGDVAGEATTATGSVTGIFQSGADTPLTYALSSDTSGLPALSSGGVALVYSVLGNTLTATAGAT--DVFTFSLTAAG
TATGTAVTGTVDEDGLTNGIPGGTGDVAGEATTATGSVTGIFQSGADTPLSYALSSVTSGLPALSSGGVALVYSVAGDTLTAKAGVAGADVFTFSLTAAG

AYTFTLLKPLDHAAGNDENDITINLGSLLKATDKDGDTVTAAAEKLVITVDDDTP
AYTFTLLKPLDHAAGNNENDITINLGSLLQATDKDGDTVTAAAEKLVITVDDDTP
AYTFTLLKQLDHAAGNDENDITINLGTLLQATDYDGDTVTAAAEKLVITVDDDTP

IVTAKSNLIYANSSNGSLVTDHGGTGVFGYSIGADTHATPYSASNSDFLSVALTGVTVGTNAITNKSVSWVSETDSQATFNVGFTYVSNNPAQGATSNAT
GTLIFDKVADTYTLKLDQEIQSFSILNTSTPGNPLQGYALNSDTTVGSNPPVSVMTLASNFFVQFEGFGVNNGTPQTFAYDSGTGLFSNDRRYVTVSSDS
IGAASDTLQSDEVIDLDFYKANPKGHTDSLLDRATSKAIFIEFTQAGIGAGKDMVVVLKLVDDVSGATINRTFIVGNSVGNDDVLNDSVPAYGFVAKAQN
GIVVFESNDYNFGTEHYSIQGAQVVTSTQNTSGNGYQLNGAIDNPATPGIEGATLSSSLTAFGGDTTEGNNEPIKIVNIGFVSTTTPDAHLTFDVALTDA
DADTTATQTLDVTIVGDNRVTASTGVNTFVIADTDVDGLLSTVLTVVDGGFATGTDKLDFTVAGSVTNFTEVPAPATNLAAFTAAADTALDGTTKYYFGV
VGGDGYLAFDADGNGITTIIELVGVTHIASTDI

Pse-24 WP_015477833.1
MATHKKNASSTSDASVSTDQQDYAPGSTASITANGFAAGSTVMFQVRHATSAGPDGTWGTPDDVLDGNAGAGHAPWYVTDGGAGDLDGKTNGSVITSWYV
NPDDSAGATFQLSAASTGADQTFGTADDTLAMASFTDSGLITVTPGVLVTLDETTELQNAIASSGTSGDADDNDILLSRLPTTFSDRLAELEAGTAMGAA
LSGYTGAVGDTGSTAFSLSGANITDVSFVGVDGKPLDGDDSGLLTLDGTSILLYTDTDNNILLGRAGENGAIVFAAYIEEIKDSPQSPPTGGKIWTVEYQ
PIQHSDATDPDDALDLHDTVFIGASQDLTFSLENAPSGQNLFLMYTTASPQTYLDGNTVRISSPAIIATGKNPADQSAGANITSGDTINTSQGGGPTTFG
TNNQMIVEQEGIRFTFVTGAKQSVTIPNLDQNEADVEANIDFTGLFDAKAASFDVVQLQSGKSAVVKISAFTTDKESGVDFVNGYSDDSAVEITSVRVIN
KSTGQVIENSDGSVNDGSIVITIASGIATITGVKAGYKIEYTTQGDHNRVLVENGAALNATGNTHADFDIGGFMLVQVAADHAEIGSRMIFEDDGP

P3 repeats
SIAPSEASAPTLTTDDTHIPDSAGPTSLANLFTSNFGTDGFKDTDHDHIQDADAITYSLGVSADNGVDSGLVDTLSGDKIYLFLENGSVVGRVGTAAGQA
SITPSEASVPTLTTDDTHIPDSDGPTSFADLFNPDFGKDGFKDADDDNVQDADAVTYALGVSADNGVDSGLVDTLSGDKIYLFLENGSVVGRVGTAAGQA
SITPSEASVPTLTTDDTHIPDSAGPTSFADLFNPDFGKDGFKDADDDNAQDADAISYSLGVSADNGVDSGLVDTLSGDKIYLFLENGSVVGRVGTSTGQA
SITPSEASVPTLTTDDTHIPDSDGPTSFADLFSPDFGMDGFKDADDDNVQDADAVTYALGVSADNGVDSGLVDTLSGDKIYLYLENGTVVGRVGTAAGQA
SITPSEASAPTLTTDDTHIPDSAGPTSFADLFNPDFGKDGFKDADDDNAQDADAISYSLGVSADNGVDSGLVDTLSGDKIYLFLENGSVVGRVGTAAGQA
SITPSEASVPTLTTDDTHIPDSAGPTSFADLFNPDFGKDGFKDADDDNVQDADAVTYSLGVSADGGVDSGLVDSQSGHKIYLYLQNGAVLGRVGTAGNMA

DPTGDIALTIAVDSNTGAVTLTQNNSVVHDDPQDPAETGTSAAGLAAANLVTLTATITDGDGDTASTSRDIGDAFKFEDDGP
DPTGDIALTIAVDANTGAVTLTQNNSVVHDDPLDPAETSSSAAGLAAANLVTLTATITDGDGDTASTSRDIGDAFKFEDDGP
DPAGDIALTIAVDANTGTVTLTQNNSVVHDDPQDPAETGTSAAGLAAANLVTLTATITDGDGDTASTSRDIGDAFKFEDDGP
DPTGDIALTIAVDANTGAVTLTQNNSVVHDDPQDPAETGTSAAGLAAANLVTLTVTITDGDGDTASTSRDIGDAFKFEDDGP

DPTGDIALTIAVDSNTGAVTLTQNNSVVHDDPQDPAETGTSAAGLAAANLVTLTATITDGDGDTASTSRDIGDAFKFEDDGP
DPAGDVALMITVAANTGAVTLTQNNSVMHNDPLDPVEAGASAAGLAAANLITLTATITDGDGDTASVSRDIGSAFRFEDDGP

SLAFGNLVGTGSVLPQLGYWDKSYGTDGLGAAGLDISLVNDQFTLVRPDNSTATGSGTLTEQSPSPDANGAFHFDGTLTADFNNDGTTDPAVHYTLTAFA
DGTYTLDLLEGFSSTHTLSSANGSLDAGGPDPVRTLTIGTEHVVFFAAVPTALPESIQIGIGSGEDDLTETQLQTDPLPSYIGTAAMNVSTSGIGIANNN
FEGNNTTGINAGDESFVVNPETLLTSMKVFIDNSLQGYNPATEELYYTIYYSDDTISGAPTKVLAGDLTAEAGNQQSFTIQAASGKMIDAVQLTMGKGAV
KIPVIEFTQQVENLASDIQLSFNATVTDKDGDTATSAFTANLFANEKAGSTFDFTLAGIAGTQEAFNVDLSRTEGKYEVTGFDVTAGQRDTLVLNGDQNA
VVDSIDTSGANSVVTVTESGGQVTTITLVGVDILNTDIVHGSV

Pse-27 WP_052248660.1
MTLDEQNSLLTTSITANALSVVATGEDVSLDETTGLQNLTATPTPAGDADDNDILVASLNSTFAARLTALGAGTATGAALSGYTGVAGNTGSNAFTISVD
PGAGITDLSFVGSNGAPLNGLDSELDTLDGTSILLYTDTNNNIVLGRAGGPTGAIVFAAYIEETGSPVTGGKIWMVEYQPLKHLNTTSADDSLNLLNKVF
IGATQDANFSLAGAPSGQQLFLMFTTMGATADVNGRIPGVSIIVTGKNPLDQSASSAAITSADTVNSSQGGGNTTLGTNNQAITEQEGLRFSFVTGARAD
VTIPNLSPGEAGLEANIDFTQYFGARAATFQVVQETGGTTAKILLTAFKNADNVNGSQSGVNFINSYANDDGEKVAITNGSVTVKTFAGVTISTAVITYN
ADGTVLITGVPTDAQITYGTATDHNRVLIENAGLLTAKNGDKTHADFDIGGFKVLQTSTSATEIGSKMIFDDDGP

P6 repeats
SADVVDGGGAVTIDETAGNQDNDSDLAAVLALFDTEVANKGTDLNPTEFAISTTPLVTTTLSSFGQDAAGGTKVLSLAIVGGDGTDSLLNTTDGKDIKLF
SADVADGGGAVTIDETAGNQDNDSDAAAVLALFDTEVVNKGTDLNPTEFAISTTPLASTALSSFGQDQESGTKVLSLAIVGGDGTDSLLNTTDGKDIKLF

KEGNLIVGRYDVANGTVTVDDPAAFAIALQQDGNVAVAQYVSLTHPTAGSSAADHDDRIDLAGLINAVVTVTDGDGDTSTDSVGIGDDINFDDDGP
KEGNLIVGRYDVANGAVTVDDPAAFAIALLQDGNVAVAQYVSLTHPTAGSSAAAHDDQVDLAGLINAVVTVTDGDGDTSTDSVGIGNDINFDDDGP

P5 repeat
AVSANSTVLLDDDALTGGNADGTDDDADAANTSGTLGLDFGQDGAGTVAYLTSGAPTGFTYVADGTSLLVKQGTTTVLTLTMVAATGAYTVAQNNPILHA
AGMTENNQAFTINYRVTDGDGDTADGTLGINVDDDTP

VIGTAPVQDADPDLGATSWTFSGDFAYSIGADKRGPTYSSAADSDFANLALSGSANDVAIVDPTVTWASENLDTATFNVSFKYDHDRSAATTPELVEGTL
VFDKVNDTYTFEMDALQTTQDVTLGEGTGYETYDMDGTIPSSGPSPVATGKLGEGFFIQITGFEAPLSAGGNTAVVAGELVSGSVAAVTLSSTALGVSGN
TIQAGEAANINFFETDPKGDLGADDFSYATDFFIKFDGYETESDDLLLIVSLADANDSSITTTRAIYVDQGDVYENDTNNSDLVGTKYEALIANDPADPN
DPFLDNNDALLIVESNDFNIQTGDNWLIKGIQILSNDAGLTGSAINLNRNVGADGASSTSAQPIVGLMGPGNTIAEDTSTNPLKIIDAGFSTTTTTPATL
DLELDFQVVDRDSDFTAVQTIDIENPVIELVGVTNLAPTDLVL

Pse-28 WP_038983706.1
MAIEITGEDVVLDETPGLQNATATPTPAGDADDNDILVASLPSDFSTRLTALSAGTATGAALSGYTGDVGDTGSDAFTVTDGAT
ITDISFVDSAGAPLNGVDSGLDTLDGTSILLYTDTNNNILLGRAGSATGAIVFAAYIEETGSPVTGGKIWTVEYQPLKHPDGTNPDDSLNLLDKVFVGTS
QDLEFSLANAPSGQNLFLMFTTADPTVVDVGGVLRITDPTIIATGKDPANQSTGVNITTGDTINTSQAAGPTTFGTNSQMITEQEGIRYSFVTGARQDLT
IPNLDQNEADVEANIDFTDVVNANKASFDVVQLQSGKTAVVKISAFSTAVESGTTFIDGYANDSPVEITSVSVINISTGLVIESSDGPEDDPAIAITFDA
DGVATITGVKAGYRIEYTTTDEHNRVLIENGAALDARGNTHADFDIGGFKLLQTAVTTAEIGSQIVFEDDGP

ALAFGNLIGTGSVLPQTGFWSNSAGADGLDAAGLDISVNSQFTLVRPNNTTTTGTATLTEVVPSPDGNGAYQFAGTLTGDFDNNADTADTTVDYTLTAFD
DGSYALDLVQGFSSTVVLSTADGSLAAGGPDPVRTLLIPDTNDPTIPSASEEVVFFSAKALASTTDILAGIGLGAPDPTEAELQTTPLPSYIDPRSMNVS
TSGIGVANNVLQGDNLVAIGATDESFVVNPESLVTAMKVFIDNSVAGYNTATEDLYYRIYYEDGTTSDLIEVNTLTPEPRGQVSFLIEQEGSLLIDAVQL
TMGRGDIKIPVIQFIQETESLASDIQLTFNATVTDRDGDSVSSTFDANLFANDLAGAFDYTLVGTDDELDAFNVDLSFDENLYQITGFDTDANLRDTLVL
NGDQSAVVQIDTSGADSIVTVTETGGDVTTITLVGVDLLSSDIVLGSA

Pse-34 WP_119892223.1
MAITITGTLIIDETLGTQNATAGGSDLTGNDVAHGLGFLNTGVSAFDTLLGAVVLHDLTGINATVSVSNGTSTDPDGSSMLSGFGADVTDLAFTNSTGGP
LLGEEALSAPGVPLLTVDGTKIFLYSYTGADLGIDENNVVFGRKANADGSANPTGDVVFAAYLQPTDASGAVQTSDLNAVGSKVWLVQYEEIKHPDTTNP
DDALSLFNLHVTVSNRSDFSLQGAPSGQNLFLMYGDGTPNATDAVIIVTGKNPINQSSDPSAITSGDTVNTGQGGGPTTLGTNNQSIVEGSGLYFTFAKG
ANTNFTVPNLDQNEADVEANIAFTSLYESNGASFSVVQSASGDKSTLEISAYTTPFNPGTAFIDNLHNNTAVAVTKVTITTQPSGHGKNAVPGETLVFDK
GVIGTTPTTLSGITVTFSVADTVVIKGLEADDLIAYNTAGPHSRVLIDNIGSTDTNFDAPFDIGGFALVNSNVTPNQFSALTFHDDGP

P4 repeats
TAQIARTADNAVLDESLGVDANDANAEDDDVPPATDPFAGSYGAPIGAVAGVNLADTTTATGTDVVGATTAVTLSIVNGNGSDSGLDTTDGTQINLWKEA
TAQIARTADNAVLDESLGLDANDANAEDDDVPPATDPFAGTYGAPIGAVAGVNLADTTTSTGEDDEGATTAVTLSIVNGNGSDSGLDTTDGTQINLWKEA
TAQIARTADNAVLDESLGLDANDANAEDDDVPPATDPFGGTYGTPIGAVAGVNLADTTTSTGEDDEGATTAVTLSIVNGNGSDSGLDTTDGTQINLWKEA
TAQIARTADNAVLDESLGLDANDANAEDDDVPPATDPFAGTYGAPIGAVAGVNLADTTTSTGEDDEGATTAVTLSIVNGNGSDSGLDTTDGTQINLWKEA
TAQIARTADNAVLDESLGLDANDANAEDDDVP-ATDPFGGTYGTPIGAVAGVNLADTTTSTGEDDEGATTAVTLSIVNGNGSDSGLDTTDGTQINLWKEA

NGDVTGRAGNASATVIFAIRIDNDGKVAVAQYDSIKHPDFPNNYDEELDLTGKLDAVVTVTDGDGDVATHNIGIGDGINFQDDGP
NGDVTGRAGNAGATVIFAIRIDNDGKVSVAQYDSIKHPNFPNNYDEELDLTGKLDAVVTVTDGDGDVATHNIGIGDGINFQDDGP
NGDVTGRAGNASATVIFAIRIDNDGKVAVAQYDSIKHPNFPNNYDEELDLTGKLDAVVTVTDGDGDVATHNIGIGDGINFQDDGP
NGDVTGRAGNASATVIFAIRIDNDGKVAVAQYDSIKHPDFPNNYDEELDLTGKLDAVVTVTDGDGDVATHNIGIGDGINFQDDGP
NGDVTGRAGNASATVIFAIRIDNDGKVAVAQYDSIKHPDFPNNYDEELDLTGKLDAVVTVTDGDGDVATHNIGIGDGINFQDDGP

P5 repeat
TVSANNTVLLDDDALTGGNPGGSNDDANSANLSGILGHAFGEDDEGAAVAYRTNGAPTGFSYELSGTSLLVKQTQNGVLTTVLTLTLVPGTGAYTVAQDNP
ILHAAGLTENNQAFTITYRVTDGDGDTADGTLSINVDDDTP

VVTAKSSLVYANTSNEPPVTDVGGTGVFAYSIGADKHVAPYSDPAGLTPNSDFLSVALTGVTVGANVITNTSVSWASETDSQAVFNVGFTYVSNNPAQGA
TTNATGTLTFDKAADTYTLKLDQEIESFSILNTSTPGNPLQGYALNSAATVGSNPPVSVMTLASNFFVQFEGFGVINGTPQTFAYDSVAGLFSNDRRYVT
VSSASIGAASDTLQSDEVIDLDFYTENPKGFATSADPDNPVNPRATSKAIFIEFTQAGPGAGKDMLVVLKLVDDLGNTINRTFIVGNSTGNDDVFNANLP
AYGFVAKAQNGIVVFESNDYNFGTENYSIQGAQVVTSTQGVSGTGYQLNGAIDNPATPGSEGATANAQIAFGGATTEGNNEPIKIVNIGFVTSTTPDAHL
TFDVVLTDADGDATATQTLDVTIVGGDGVTASANADTFVIGDTDVDGLLSMVMTVIDGGFASGIDKFDFTAAGSGTNYTEVLAPATNLAAFTAAADAALD
GTTDYYFGVVGGDGYLAQDADGAGITNIIQLVGVIDMASTDIV

Pse-35 WP_069085698.1
MAITITGTLIVDETLGTQDNDVLAGLGALITGVSAFDTLLNAVVVQDLTGVNVTVAVSNGSSADLDGSSMLSGFGADVTDLAFTNSSGGPLLGEEAQSAP
GVPLLTVDGTKIFLYSYTGADLGIDENNVVFGRKGVWNGSEYVADPSGDVVFAAYLQPTDASGAVQASDLNAVGSKVWLVQYEEIKHPITTNPDDALSLF
NLHVTVSNRSDFSLQGAPSGQNLFLMYGDGTPSATDTVIIVTGKNPINQSSDPSAITSGDTVNTGQGGGPTTLGTNSQQIVEGTGLYFTFATGANPNFTV
PNLDQNEADVEANIAFTELYGTNGASFSVVQSASGDKSTLEISAYTTPFNPGTAFIDNLHNNTLVEVTKVTITTPPSGNGKKAVPGETLVFDKGSIGTTP
TTLSGITVTFSGKTVEIKGLEANDLIAYNTNGLHNRVLIDNIGSTDTNFDAPFDIGGFALVNSNVTPNQFSALTFHDDGP

P6 repeats
SADVADGGGAVTIDETAGNQDNDSDAAAVLALFDTEVTNKGTDLSPAEFAISTTPLATTALSSFGQDQEGATKVLSLAIEGGDGTDSLLDTTDGKNIKLF
SADVADGGGAVTIDETAGNQDNDSDAAAVLALFDTEVTNKGTDLSPAEFAISTTPLATTALSSFGQDQEGATKVLSLAIEGGDGTDSLLDTTDGKNIKLF

KEGNLIVGRYDVANGTVTGDDPAAFAIALLQDGNVAVAQYVSLTHPIAGNSAAAHDDRINLAGLVNAVVTVTDGDGDSSTDSFGIGDDINFDDDGP
KEG-LIVGRYDVANGTVTGDDPAAFAIALLQDGNVAVAQYVSLTHPIAGSSAAAHDDRVNLAGLVNAVVTVTDGDGDSSTDSFGIGDDINFDDDGP

P5 repeat
TVSANNTVLLDDDVLTGGNPGGTGDDADSANVSGTLGHAFGQDGAGTVAYLTSGAPTGFTYVADGTSLLVKQGTTTVLTLTMVAASGAYTVVQNNP
ILHAAGLTENNQVFTINYRVTDGDGDTADGTLSINVDDDTP

VVTAKSNLVYANTSNEPPATDVGGAGVFAYAIGADKHTGIYSALNSDFLSVSLTGVTVGANAITNKVVSWASETDSQAEFNVGFTYVSNPVQQTTTNATG
TLTFNKVAGTYTLKLDQEIESFSILNTSTPGNPLQGYALNSATPVGSNPPVSVMTLASNFFVQFEGFGVINGTPQTFAYDTVAGLFSNDRRYVTVSSASI
GAASDTLQSDEVIDLDFFTENPKGFATSADPDNPVNPRATSKAIFIEFTQAGPGAGKDMLVVLKLVDDITGATINRTFIVGNSTGNDDVFNANLPAYGFV
AKAQNGIVVFESNDYNFDLGGGVFENHSIQGAQVVTSTQGVSGTGYQLNGAIDDPATPGGEGATTNAQIAFGGDTTEGNNEPIKIVNIGFVASTTPDAHL
TFDVVLADADGDATATQTLDVTIVGGDDVTASANADTFVIGDADVDGLLSMIMTVIDGGFASGIDRFDFTAAGSGTNYTEVLAPVTNLAAFTAAADAALN
GTTQYYFGVVGGNGYLAQDIDGAGITNIIQLVGVNNMASTDIV

Pse-36 WP_169910027.1
MAIILNSSITVDETSGLQNAATSTDIEDINDNDITVAAINATDDTGDLPFPFESRLFALLGATPVKNAALSGYTGAAGNTGADLITITGTYVDLAFTDAQ
GKALGDPGNANGGSDWSGLYTLDGTKIFLYSDTNNNIVLGRAGSEGATPAPDDDLANPNGTIIFAAYLEQTATGAKVWMVQQEQLMHPDTANPDDVVDMT
NHLWVTASQDLAFDFAGLPSGQNGFLMFKAGGSTTGLIVTGMDPDQVPANQADTVNTSQGAGPTTIGTNSQGIKAGEGMIFSFITNPDARYTVPDLTHGE
ATNESNILFGGLFAASGATIIIAQATPPKTCAATISAFTTGLEVGSAYYDGLLDGNGHAVNGADNLGYIASVTVIGANGVAIAGLTGVTTNTTLGSISVT
FTTSNAGLDRTATIVGFEANFSLQYTVDGTHNRVVIENSGTGNSTFDIGGFHLPNVVAVPQEVGSQLNFEDDGP

P12 repeat
AAAGLPVTALVDEDSLAGGIDGGVGDAGLLVPASASGTVATVFTSGTDAPASYSLSNDTSGVQVFDSAATAVALASKGETVKYDVIGNTLWGYVGAAAEY
VAATDRAVFKLELTNTSDGSYTFTLLDQLDHPDTVGGDNSENELLLQLGSVLKVTDKDGDSVTATAQKLVITVDDDTP

P13 repeats
IATLNQLTGTVDEDGVLEGAANAGPGDGIAGGTGDVAGEIVLASGNISTLFQSGADEALNYSLTTNGLAALGLKSAGVTVTYAVAFDAVSSTWLLTASAG
IATVNQLTGTVDEDGVLEGAANAGPGDGIAGGTGDVAGEIVLASGNVSTLFQSGADEALSYSLTTNGLAALGLKSAGVTVTYAVAFDAVSSTWLLTASAG
IATVNQLTGTVDEDGVVEGAANAGPGDGIAGGTGDVAGEIVLASGNVSTLFQSGADEALSYSLTTNGLAALGLKSAGVTVTYAVAFDAVSSTWLLTASAG
IATINQLTGTVDEDGVLEGAANAGPGDGIAGGTGDVAGEIVLASGNVSTLFQSGADEALNYSLTTNGLAALGLTSGGVAVTYAVGFDAGSSTWLLTASAG
IASINLLTGTVDEDGVHEGAVDSGPGDGIPGGTGDVAGEVVLASGNVSALFQSGADEPLSYSLTTNGLTALGLKSGGVTVTYATTYDAGSNTWLLTASAG

AGNTAFTFSLHATTGAYAFTLVDQLDHAPGLNENNLTIALGTAINATDFDGDTVTAAANGLVITVNDDTP
AGNTAFTFSLHATTGAYAFTLVDQLDHASGLNENDLSIALGTAINATDFDGDTVTATANGLVITVNDDTP
AGNTAFTFSLHATTGAYAFTLVDQLDHASGLNENDLTISLGTAINATDFDGDTVTAAANGLVITVNDDTP
AGNTVFTFSLHAATGAYAFTLVDQLDHAAGQNENDLSIALGTTINATDFDGDSVTAAANGLVITVDDDTP
AGNSVFTFSVQAATGAYAFMLLDQLDHAAGQNEDDLNIALGTAINATDYDGDSVTAAANGLLITVDDDSP

VVTAKSNVVYANSSNPTPGGTGVFAYDIGADDRLGTVYSSANSDLSVSMGDSTVGTTAITNKVVTWASESDTSAVFNLSFTYVSNNPTLGALTNATGTMT
FDKVADTYTLSLADDIDSFSILKTSAAQGFTGYNIGGTTPINQQPPVSVAKLADDFFVQFSGFAEQGSGTGANNIMAGGNNAFVPGELFSAATSWVSVSG
TAAGVAGDTIQQGEVMDLDFFTASPFGDTTAAPTATSKGIYVKFDGIGSEDLILVLKLIDPDDNSLITRAIIVDSEDIIIKNASTSPNNFHPIANPAAYG
ITLDQNDGAVIVESNDYNFGNENYVIQGLQVLVSTEGVTGSGYNLNGNVGVSGASTGNTLTFSNESGEGSKSNPVEAGEAGTWDGDVVKITDLGFVTSST
PDAHLTFNVVVTDADADATPAQTLDVTIEGDKTFTGGVGADTFNFSGLDTDGSLTAATAVISAGFTSGVDTLNFPTAGTGANYTEVAAPAANVAAFITAA
DTALNGTTNYYFGVVGGDGYLAYDGDGTGITSIIKLVGVTDIAPTDIV

Pse-38 TDU99816.1
MRKLTRKSIVDFCRNKFPAVSPVTVIRQESTMAIILNSSITVDETSGLQNAATSTDIEDINDNDITVAAINATDDTGDLPFPFESRLFALLGATPVKNAA
LSGYTGAAGNTGADLITITGTYVDLAFTDAQGKALGDPGNANGGSDWSGLYTLDGTKIFLYSDTNNNIVLGRAGSEGATPAPDDDLANPNGTIIFAAYLE
QTATGAKVWMVQQEQLMHPDTANPDDVVDMTNHLWVTASQDLAFDFAGLPSGQNGFLMFKAGGSTTGLIVTGMDPDQVPANQADTVNTSQGAGPTTIGTN
SQGIKAGEGMIFSFITNPDARYTVPDLTHGEATNESNILFGGLFAASGATIIIAQATPPKTCAATISAFTTGLEVGSAYYDGLLDGNGHAVNGADNLGYI
ASVTVIGANGVAIAGLTGVTTNTTLGSISVTFTTSNAGLDRTATIVGFEANFSLQYTVDGTHNRVVIENSGTGNSTFDIGGFHLPNVVAVPQEVGSQLNFEDDGP

P12 repeat
AAAGLPVTALVDEDSLAGGIDGGVGDAGLLVPASASGTVATVFTSGTDAPASYSLSNDTSGVQVFDSAATAVALASKGETVKYDVIGNTLWGYVGAAAEY
VAATDRAVFKLELTNTSDGSYTFTLLDQLDHPDTVGGDNSENELLLQLGSVLKVTDKDGDSVTATAQKLVITVDDDTP

P13 repeats
IATLNQLTGTVDEDGVLEGAANAGPGDGIAGGTGDVAGEIVLASGNISTLFQSGADEALSYSLTTNGLAALGLKSAGVTVTYAVAFDAVSSTWLLTASAG
IATVNQLTGTVDEDGVVEGAANAGPGDGIAGGTGDVAGEIVLASGNVSTLFQSGADEALNYSLTTNGLAALGLKSAGVTVTYAVAFDAVSSTWLLTASAG
IATVNQLTGTVDEDGVLEGAANAGPGDGIAGGTGDVAGEIVLASGNVSTLFQSGADEALSYSLTTNGLAALGLKSAGVTVTYAVAFDAVSSTWLLTASAG
IATVNQLTGTVDEDGVVEGAANAGSGDGIAGGTGDVAGEIVLASGNISTLFQSGADEALNYSLTTNGLAALGLKSAGVTVTYAVAFDAVSSTWLLTASAG
IATINQLTGTVDEDGVLEGAANAGPGDGIAGGTGDVAGEIVLASGNVSTLFQSGADEALNYSLTTNGLAALGLKSAGVTVTYAVAFDAVSSTWLVTASAG
IATINQLTGTVDEDGVVEGAANAGPGDGIAGGTGDVAGEIVLASGNVSTLFQSGADEALNYSLTTNGLAALGLTSGGVAVTYAVGFDAGSSTWLLTASAG
IASINLLTGTVDEDGVHEGAVDSGPGDGIPGGTGDVAGEVVLASGNVSALFQSGADEPLSYSLTTNGLTALGLKSGGVTVTYATTYDAGSNTWLLTASAG

AGNTAFTFSLHATTGAYAFTLVDQLDHAPGLNENNLTIALGTAINATDFDGDTVTAAANGLVITVNDDTP
AGNTAFTFSLHATTGAYAFTLVDQLDHAPGLNENNLTIALGTAINATDFDGDTVTAAANGLVITVNDDTP
AGNTAFTFSLHATTGAYAFTLVDQLDHASGLNENDLTIALGTAINATDFDGDTVTAAANGLVITVNDDTP

AGNTAFTFSLHATTGAYAFTLVDQLDHASGLNENDLTIALGTAINATDFDGDTVTAAANGLVITVNDDTP
AGNTAFTFSLHATTGAYAFNLVDQLDHASGLNENDLSIALGTAINATDFDGDTVTAAANGLVITVNDDTP
AGNTVFTFSLHAATGAYAFTLVDQLDHAAGQNENDLTIALGTTINATDFDGDSVTAAANGLVITVDDDTP
AGNSVFTFSVQAATGAYAFMLLDQLDHAAGQNENDLNIALGTAINATDYDGDSVTAAANGLLITVDDDSP

VVTAKSNVVYANSSNPTPGGTGVFAYDIGADDRLGTVYSSANSDLSVSMGDSTVGTTAITNKVVTWASESDTSAVFNLSFTYVSNNPTLGALTNATGTMT
FDKVADTYTLSLADDIDSFSILKTSAAQGFTGYNIGGTTPINQQPPVSVAKLADDFFVQFSGFAEQGSGTGANNIMAGGNNAFVPGELFSAATSWVSVSG
TAAGVAGDTIQQGEVMDLDFFTASPFGDTTAAPTATSKGIYVKFDGIGSEDLILVLKLIDPDDNSLITRAIIVDSEDIIIKNASTSPNNFHPIANPAAYG
ITLDQNDGAVIVESNDYNFGNENYLIQGLQVLVSTEGVTGSGYNLNGNVGVSGASTGNTLTFSNESGEGSKSNPVEAGEAGTWDGDVVKITDLGFVTSST
PDAHLTFNVVVTDADADATPAQTLDVTIEGDKTFTGGVGADTFNFSGLDTDGSLTAATAVISAGFTSGVDTLNFPTAGTGANYTEVAAPAANVAAFITAA
DTALNGTTNYYFGVVGGDGYLAYDGDGTGITSIIKLVGVTDIAPTDIV

Pse-42 WP_064582715.1
MASLVGIVSKAVGEVFAVAADGSRRILHQGDKLFVGEQLVTGHEGAVAVAIAGGGELTLGRDSSMPMSNQLVAAAHHQYADSAPAQAPVAPSQQDITDVK
ALQAAIAAGQDPSQVAEATAAGPSFSAGGNAKAGGGHSFVLLTETGGHVDPDVGFPTGPLGSGPLFPRLFDGIPSPEQAQAAPPTPPEPPTPPVDGVPSA
GNASAVVDEDGLPGGIQGNDGLPGVGASASGSLGYDFGADGPGTFAWSTTGLPALTSGGVALSYSVSPDGHVLTASANGQTVFTLTLTNLANGTYELVLQ
QPLDHPQHGVEDDIALSVPYTLTDGNGTPASGNLAILINDDSP

P8 repeat
-TAGLAQEGEAPSLLSHDEGTRGGASESVS ADFASAFKATLAYGADGAG-SVAWHYSLNVTSQGEA-SGLTSAGQGIHLYLIDGVVVGSTAASAGAISS
ANTVFSLAVDAASGKVTLTQYAAIDHALPGATSGFDSQQVSLGSDLVQLQGSVTITDRDGDPASASQNLDLGGRIGFADDGP

P7 repeats
SIGIGASGAQLGNLVTQDADTIGSNFDSATTSFAAAFAITSQSTGADTPA-AVNWSYALKLDVAEGTESGLNSAGQKIYLYEQNGQIIGSTSSTEAGVDS
SISVGLSGNAPVLLHTQDADTIGANFDTATTSFAAAFAITSQSAGADTPA-AVSWSYALKLDVAEGTQSGLNSAGQKIYLYEQNGQIIGSTASSEGGVNA
SICIDLSGKVPVLLHTQDADTIGSNFDSATTSFAAAFAITSQSAGADTPA-AVNWSYALKLDVAEGTQSGLSSAGQKIYLYEQNGQIIGSTASSEGGVNA
SICIDLSGGKPVVLLTHDADTIGSNFDTATSSFAAAFAITSQSAGADTPA-AVSWSYALKLDVAEGAQSGLNSAGQKIYLYEQNGQIIGSTSNTEGGVNA
SISVGL-GSQSVLVQTQDSDTIGANSDTASGDFTSAFVITSQSAGADAPASAVSWSYSLKLLAGQGADSGLSSGNATIHLYDVGGKIVGSTATSLQGVTT

SNTIFNLKVDG-SGNVTLTQYAEIDHALPGATSNYAAQQAFLGTGLVSLDATATITDYDGDKASSSASIDLGGKVAFDDDGP
SNTIFNLKVDG-SGNVTLTQYAEIDHALPGATSDYATQQTPLGTGLVSLDATATITDYDGDKASSSASIDLGGKVAFDDDGP
SNTIFSLKVDG-SGNVTLTQFAEIDHPLPGSTSDYSAQQTPLGTGLVSLQATATLTDFDGDKASSSASLDLGNKVAFDDDGP
SNTIFNLKVDG-SGQVTLTQYAEIDHALPGATSNYATQQAPLATGLVSLQATATITDYDGDKASSTASLDLGNKVAFADDGP
ANTVFDLRLDGNSGKVILTQYSAIQHDGPGASGNYDSQVAWLKTGLVGLEGSASITDYDGDKASSSQTLDLGNKIGFADDGP

TAVDDHPACLLVQPPPAVNLTLVLDVSGSMQGSKLAQAKAALINMLHEYAAMGLLIQVSLISFATSASAASHYSFDDAGDSGYTSLVNAINGLQAGGNTN
YEAALNRAQTSINADLNAPGADPAAINRVYFISDGQPTTGSTNSALNAWQSFLAAHNGSTNNVEAFAVGVGTSITDQDLVRVDSHGTPVIVTDPSDLSAT
LVNLSHLDAVSGNLLANDLPVSADGTVRITQVSIGGETFAVDANGNFTNLDPSASHATGSYNASSGLLTISTDNGTLTLYLKDGGGHHAGDYTYAVKASV
SFPESGQISESFQYTIVDGDGDTASANLDICVKYGAPILVVGSNASDTDGSTTPHVVPSPLGPDHGTIAGSFGNDVLIGDVGGVSVTTTPGKNYNIALII
DTSGSMADPSGTTGLSRMALAKAALINLLNQIKGHDGTINLALVSFADGSTLTQVHGLNSTNVATLISAINSLGAVGGTNYEAALKSTVDWFNATVGGGA
NAAHDYINLAFFLTDGNPTTYIGDNSNSGTVTNYHDIKDALDVANNLLNGGGLFSGSDHVSVNAIGIGNGINSGILQFFDNTSTTGTATLHFTGNTPDLT
ANVGQPQIINTAEQLQTALQEGFSNTSPQAVGHDHLVGGDGSDILFGDVINTDALSWTGHAAGTHNGAGYQGLVDYLTAANGGAAPSQGQVTNYIVAHAY
ELNVAGDTRGGNDTLEGGKGDDLLFGQGGNDTLLGGDGNDILFGGMGDDTLTGGAGADQFVWQKGEVGHDVIKDFNIGEGDSLNLADLLQGEHATADSLA
QYLTLSVNATTSTTTISVNPTGATGGATTQSIDLANVDLVARYTDHHGGAIVSGGDTHAVLNGLLADHAVKVDTV

Pse-47 WP_052239091.1
MATLMGVVSKVIGEVFAVAADGSRRPLEQGDKVFVGEQLVTGANGAVALKVAGGGEITLGRDSALPLTSQMLAAAHQAQNGDEQVAQQPAPPSAKDTADV
KALQAAIAAGADPTQQAEATAAGPQAGNAANGKPGGGHSFVLLTEVGGHIDPTIGFPTGPIGSGPLPLREFDGVPQPRAEAVTPPEVLPPDGQPSAGPNG
PGTANVFEAGLPGGIPDGAGEGATFTGSLGYNFGSDGVGSFSWGTAGLPSLTSGGVAITYSVSPDGHVLTGSANGTPVFTVTLTDINTGAFEVNLLQPVD
HPVKGSEDTLTFDIPYTITDGNGTPANGTLTVGIVDDAP

P8 repeat
QAGLSADNGVDVLLQTHDANTAGAAFDTSTADYSSAFQVTANYGGDGAGSTQLTYSLNLVGGNGANSGLTSGGATIYLYSVGGAIVASTSASAAGITGEN
TVFSLSVNGSGVVTLTQYSAIDHALPGSESGYASQTAVLDSGLVQLQGNLTVTDRDGDSVSSSSSIDLGGRVSFTDDGP

P9 repeats
TVAVGNTEGLHLTVDETNLGQDATS-SMAVGDLFNAQFGADGAGTITYRI-GTTDNTDSGLKDTATGDKIFLFNTANGVEGRVGGADGAVAFRVTLGSD-
RIAPVEGGDIHLTVDETQLGQAAT--SNSVADLFNGQFGADGAGSISYKL-EAADNTDSGLKDTATGERIFLYNTANGVEGRLEGT-STVAFRVTIDGD-
SITPCQDADIKLTVDETHLGVNDTS-SITVSDLFTSHFGADGAGAITYKL-TTEDGKDSGLTDTATGEKIFLYNTANGVEGRVGSS-ADVAFRVTLGAD-
SITPCQDADIKLTVDETHLGVNDTS-SITVSDLFTSHFGADGAGAITYKL-TTEDGKDSGLTDTATGEKIFLYNTANGVEGRVGSS-ADVAFRVTLGAD-
SITPCEDADIKLSVDETHLGVPDTS-SASVADLFTTHFGADGVGEITYKL-TGTDNTDTGLKDTATGSRVFLYNTANGIEGRLEGT-STVAFRVSIDGD-
SIAPSSIYDISLEVDESDLSNDARVESSLVSLLFETHFGADGPGSITYKV-STVDGTDSGLK-TSQGESILLYNVDGTVVGKVQG-SGAIAFVVSED-Q-
RIEPCTDADIKLTVDETKLGVDAQASAGDVAALFTGHFGADGAGGITYKV-TTTDGTDSGLKDTATGGKIFLYNTANGVEGRVEGT-NLVAFSVN-QHN-
SIQPCTDADIRLEVDESNLASDARVEAPLVSELFTASFGADGAGSVTYKV-STVDGTDSGLRETAGGARILLFNDGNGVVGKVEG-TGAIAFVVRPD-G-
SIVANQADGPALVVDESNLGANA---SASVSSLFTSHYGADGAGSLVYKVVAAQDGSDSGLRVTS-GDKIYLYNEGNGVVGRVGGQSGAVAFTLTVN-N-
SITASAQGAPALVVDETNLGIDA---SANITSLFTSSYGADGAGSLVYKVVAAQDGGDSGLRVTS-GDKIYLYNEGNGVVGRVGGANGAVAFTLTLN-N-
SITANAAAAPTLTVDETHLNVDA---TANFSTAFTANAGADG-GSTTYSL-SATTGTQSGLQTTDGQNIVLVKVSATVVEGHVGTASGATAFTLTLDSGT

GKITLDQVRALVHPDAADANDPLSLGTGKISLSATVTDADGDHQSASLDLGSKLTFLDDGP
GKVTLEQLRALAHPDASDPNDALTL-AGKIALTATITDKDGDSATAQIDLGGKLTFLDDGP
GKVTLDQVRAVVHPNTSDANDGVSLDANSVTLTATITDKDGDSAQAHIDLSGKLTFLDDGP
GKVTLDQVRAVVHPNTSDANDGVSLDANSVTLTATITDKDGDSAQAHIDLSGKLTFLDDGP
GKVTLEQLRALAHPNTSDANDALNL-SGQVTLTATITDKDGDSASAHVDLGSKLTFLDDGP
GALMLDQRLALKHPDTNDADDPLRLESGKITLTATITDGDGDKASAHVDLGSKMVFYDDGP
GVITLDQQRALVHPVTTDANDPLSIGADKITLTATITDGDGDHASASIDLGGKLTFLDDGP
GALTLDQRVAFIHPDAHNDDEALSIASGKITLTATITDKDGDSDSASIDLGSKLVFHDDAP
GTVTLDQQLALKHPDGNNPDDALSVASGKISLTATITDGDGDSHSASVELGSQLSFHDDGP
GSVTLDQQMALKHADASNPDDALSIADGKISLTATITDGDGDSKSASLDLGSKLSFHDDGP
GVVKLDQQLALKHPNASDPNDPLSLASGKIGLVATVTDGDGDTKSASIDLGSKLSFLDDGP

TAVDDSPRCLVQQAPPAVNLTLVVDVSGSMAGDKLTNAKAALVNLVQQYAAMGIAIHVSLLSFAATAKDLGDFNFTGTSDAQYTALVNKINGLSADGETN
YQAALDLARTNVTSDITVSGHDPAAINRVYFISDGEPTTGNTNSAITQWKAFLANPDGDGNDATNHVDAYAVGLGASITDADLKGVTNTGDPIIVTKPSD
LTATLQNLVTIDSVSGNLLANDLPVSADGTVRITQVEFGGEKFAVDAAGNLTNENPNATTATGSYNASSGLLTIQTANGTLTVYLKDVTGHQAGDYSYAS
KAGVAFPESGQISEVFKYTIVDGDGDTASANLNICLKYGQPILVVGSNSGDVDGSTVLHTVPSPLDADKAGQINGSGGNDVLIGDAGGVSVYTQPGKNYN
ISLIVDTSGSMADPSGSNMSRMALAKNALINLVNTIKGHDGVINVSLVSFSDNSHVTTFNGLSAANVTALIAAINALSASGATNYDDAMKAASQWFNTQV
TTNHADAAHGYINLAFFLTDGNPTVSNSDSSSGNITNYGDMNNALVSGNDMLHHAGALTGDNAVDVNAIGIGNGINSNYLKFFDNTDTTGTGSVTLDGKT
INASVGEPQIINTAEQLQAALQEGFSTSTPVTVGNDHLVGGSGNDIIFGDVINTDSLAWAGHAAGSHNGQGYQALVDYLTATNGSAPTVAQVTNYIVQHA
DQFNVAGDTRGGNDILEGGAGNDLMFGQGGNDKLIGGPGDDIMYGGTGADEFIWKSGDTGHDVIKDFSIAEGDSLNLADLLQGALPTADSLSHYLSFSVN
SGTTSIAVSPTSGGAATQTIDLANVDLSAKYAGHAGSGVLSAGDTHTVLNGLLGDHAIKTDTV

Pse-57 WP_063543780.1
MAKSIGVVSKVIGQVFAVGSDGAKRLLVEGDRLFAGEQLETGAAGAVAVRLHNGAELTLGRGSSLEMTPDLLANRAPHVHAPDTLPSQAQLSDVERLQQA
IAAGADPTQEGEAPAAGPGANGAPGALGGGHSFVLLTEVAGRVDPTIGFPTAGFNGIPELANRYIGTLDVDNNAGPFGPPAIPPINPPINPPVNPPVTPP
DNPVTLRGLDLSPAELTLNEANLPLGSASNPAALTQSGSFTVVAPDGVFNLSVGGINVVTAGVVTGVGQSITTGLGNILTITGYNPSTGVVSYSYTLTGA
EQHADGAGNNELGEHFPVLVSDTDGDVASGSLDVIIRDDVP

P10 repeat
TAVNDTNAVTATEQHTQLTGNVLDNDVQGADRVVGGPVVAGTYVGTYGTLVLASDGSYTYTLNPEDADFKNLNGGGHGVETFTYTLKDADGDTSSATLTL
NITNLDDPVTLGGLDVEGGELTVYEKHLSNGSAPDSTALTQSGSFTVSAPDGLQTLTVGGISVVSGGLVAGFPQSITTALGNTLTITGYNPTTGVVSYSY
TLLGNEGHPVGEGTNSLSEHFSVIAQDTDGSSATATLDVNIVDDVP

P11 repeats
QAQPDSASVVEGGTVSGNVLLNDQLGADQAGTSNVVVGARAGSDTSTSAIGNLNTAIGGLYGTLTIDAAGNATYHANPNAVAPAGATDVFVYTIRDTDGD
KAHSDFANVFEGGTVSGNVLVNDVFGADVVADGKYVVGVRAGGDTSTSAIGQLGVQVVGQYGYLTLDAQGNATYHANPDSVTPPGATDVFVYTIRDADGD
KAHCDVASVTEGGVVSGNVLDNDIVGADVRGDGQYVVGVRAGSDTSTSAIGQVGSQVMGQYGYLTVDAQGNATYHANPNSVSQAGATDVFVYTLRDADGD
KAHCDVASVTEGCVVSGNVLDNDIVGADARADGQYVVGVRAGSDTSTSAIGQLGSQVMGQYGYLTLDAQGNATYHANPDSVSKAGASDVFVYTIRDADGD

ESTTTITINVKDCSLIAGPNDGVTVYEKALDLVKDGNDLAPGTVTGSAPSSTGETASGSLAGSVSGGIGALTYTLVGSAVGQYGQIQLNADGSYTYTLTS
ESTTTITIDIHDCSLVATPDGGVTVYEKALDLVKDGNDLAPGTVTGSDPHSPGETASGSLAGSVSGGIGAMTFTLVGNATGQYGQIQLNADGSYTYTLTS
ESTTTITINVHDSKLVACEDSEVKVYEKALDLNKDGNDLAAGNVTGSDPHSTAETASGSLAGSVGGGVGALTFSLVGNATGQYGQIQLNADGSYTYTLTS
ESTTTITINVQDSKLVACEDRDVTVYEKALDLVKDGNDLAAGTVTGSDPHSTAETASGSLVGSVGGGVGALTYSLVGNAVGQFGQIQINADGSYTYTLTS

APKSPDNVNDGPNSLTESFTYEVKDSLGNSTTSTIVITIVDDVP
APKSPGNVNDGANTLTESFTYQVKDSLGNSTTGTIVISIVDDVP
APKTPGNANDGANSLTESFTYKVTDSVGNSTTSTIVITIVDDVP
APKTPGGADDGANRVVEYFTYKATDSLGNSVTSSIAISIVDDVP

KAVCAERSITPGQVDSNILLVIDVSGSMNVDSGVQGLSRLALAKQAINALLDKYDDMGDIKVQIVTFATGAWVKTPVWVSIAEAKSIVAGLSADGSTYYD
SAATKAQEAFVTSGKIAGAQNISYFFSDGVPTNGHSMTAVRETAWESFLDNNGIKSYAIGLGTGVNQANLDPLAYDGSSHVDTDSVVVTDLSQLSAVLSG
TVQGAPITGSLMSGGHFGADGGFIKALLVDGTTYTFDPKANAGQGGYTVAGGPDKASFDTTGNTLSIKTALGGTLLVDMDTGEFTYTPPKDAGSSQVEKF
GFTVSDNDGDTSSASLVVKINSNSAPVAGADHIITNIAGASVTVPAEALLANDSDADHDHLSAAPITFNTGFADKGAGFTVGSQVQTLKFDGTVNKPENQ
LKDLARSDFTAVSGSMTAALVIAGYLGSISTGNANDEDTLTVTLRKGESLSLDHDRPAGNLMLEWKDANGNYQPIADGGSFTASHDGVYSIHVINSENAL
GNSKAAENYKLSLVIDYSHAENEVYQGSYTLSDNHGGSASGAVDITYQAGHTLQGTAGDDVLLAGNGDDTLNGGAGHDVLVGGVGNDALYGGEGDDLLIG
GPGNDLLDGGPGNDTASYASATAGVTVDLGHVGAQNTGGAGLDTLVSIENLIGSDDNDTLIGNDGDNVLSGGLGDDILIGGGGNDILIGGPGNDTLTGGS
GNDTFVWQKGDTGHDRVTDFTPGSDRLDLSQLLQGENATAASLDDYLHFKVSGSGVNVVSTLEVSSVAGAAATQTIDLAGVDLAQHYGVTAGAGGMIAAG
HDTATVINGMLNDHSLKVDTV

Pse-63 SFP88769.1
MATLIGIVRLVVGEVFAVAADGGKRQLGEGDRVYVGEQLLTGGGGAVAIRLVNGGELTVGRDSSLRLDNQLLAGEDARAAIQSATPEHAPSALELAEVAE
AQQAIAAGVDPTLSLPATAAGPGGGGAGSGGGGISFVLLDETAERVDPNIGYPTGPLAFAPLFPEVFPAGADESPESFPALIIEYFADATGQVLVQRGFV
DEAALPGGSQAQSNAETSYGRISISSPDGIAAVQVLDKNGNWVDVMDGGTVQGQYGTLVVAPNGSWVFTLDSSAPHPGATLTGTDDQFQQSFQVRVIDND
GDVSPPATLVIDIADDGP

P15 repeats
TAFADSNAVLEGDVVTGNVLTDGADDEFGADGPSAGGGVVGVRVAGADTTTAVTGGVGVGMSISGLYGTLTLQANGSYSYDGNPNVVPETGATEVFVYTI
TAQADLNSVVEGDAVSGNVLSDGAADKFGADGPAAGGGVVGVRAAGADTSTAVTGGVG--MSIGGLYGTLTLAADGSYTYTSTPNTVPLAGASDVFVYTI
TAQADLNSVVEGAAVSGNVLSDGTADVFGADGPAAGGGVVGVRAAGSDTSTAVTSGVG--TTISGLYGTLTLAANGSYTYSSTPNAVPPAGASDVFVYTI
TAQADLNSVVEGAAVSGNVLTDGTADVFGADGPAAGGGVVGVRAAGSDTATAVTSGVG--TTISGLYGTLTLAADGSYTYTSTPDKVPLAGASDVFVYTI
TAHADLNSVVEGAAVSGNVLSDGSADVFGADGPAAGGGVVGVRAAGSDTSTAVTGGVG--TTISGLYGTLTLAANGSYTYTSTPNAVPPAGASDVFVYTI
TAHANIDAVIEGDVVSGNVLTDGVGDVFGADGPAAGGGVVGVRAAGSDTTTAVTSGVG--TTISGLYGTLTLAADGSYTYTSTPNAVPLAGASDVFVYTI

RDGDGDLSTTTLTISLSDSALRVNGEDDLSVNEAAL---------ASGSNPLSTAETIAGTVADNVQG--GTGPYTYALQGSGTGSYGTLTFNADGSYSY
KDGDGDLSTTTLTINLSDGGLRVNDEGDLSVNEAAL---------ASGSNPLSTAETIAGTVADNVQG--GTGPYTYALQGSGTGSYGTLTFNADGSYSY
KDGDGDLSTTTLTINLSDGGLSAPADQDVRVYENALDLVKDGLDLAPGTVTGSLPGSTGETDADNQLDAVGVVPLSYVLVSDAAGTYGTIQINLDGSYTY
KDGDGDLATTTLTISLSDSGLSAPVDQDVLVYENALDLLQDGADLAPGTVTGSLSGSTGETDADNQLDAVGVVPLSYVLVGEAAGTYGTIEINPNGSYTY
KDGDGDLSTTTLTINLSDGGLTAPADQDVRVYENALDLVKDGLDLAPGTVTGSLPGSSGETDADNQLNATGVGTLTYALVGEAAGAYGTIQIDADGSYTY
KDGDGDLSTTTLTIYLIDSGLYAPDDHDVRVYEHALDLVKDGQDLAPGTVTGSLPGSNGETDANNQLNAIGMGTLTYALVGEAAGAYGTIKINADGSYTY

TLTQPYDTSPDANNSAHTENNAETFTFVVTDANGNTQTGTITIDIVDDVP
TLTQPYDTSPDANNSAHTENNAETFTFVVTDANGNTQTGTITIDIVDDVP
TLTSPYISGG-ANDGANIELGVDSFTYQVTDGHGNILTGTIRIDIVDDVP
TLTSPYTSAGDANDGANVEAGVDTFTYQVTDGNGNTLTGTIRIDIVDDVP
TLTKPYTSSPDANDGANVEAGADSFTYQVTDGYGNTQTGTIHIDIVDDVP
TLTKPYTSSPDANDGANVEAGADSFTYQVTDGYGNTQTGTIHIDIVDDVP

RAFLPEHVMQVDQVSETRSISGDLRFAEAAGADGVGDVAFTFTEGALALDANGNRITLDDQQLYLYYGADNTQLVAKTGPNGDVGFTIDIDAATDSYTLT
TFGVLSNDTSTILSNLSGVGSAGNVNFKGLIDIGGTLQDVLMSSTSGSINSTPTDVAVGNQWISTAENIRFDFLNDLAVNAQSSTGFSYSSHNLISSFRQ
SIAQVQSSNGIVNLTILAIIADNDNVFGSGDPGESYVPLSVSDIRVYDALGNDVTGSVTLVDNGDSITIRGMYDDWSYEVSSATQFSALQVTGAAGGSSF
ALGPFSYSQVIPDQPIELSYQIHASDGDGDAVFSSLQATLYPRTDTLEGDAGSNSLSGTAGADWIFGHDGNDSLSGLLGDDALSGGAGDDILNGGQGNDL
LSGGAGADTFVWSSGDTGIDRITDFTPGEDHLDLSDLLVGVDGALDSPELASSLTGYLNMAFGASTTITVNPDAAGPLPANQSIVLEGIDLSQVYGSSDE
ATIIQGMLDDGSLKVV

Pse-64 WP_147285553.1
MATLIGVVSQVVGEVFAVASDGSRRPVSEGDRLYAGEQLITGMSGAVAVALTNGQELTLGRDSGIALNAQLIASRDELQAPVVEVSPTAPSDDDLTDVER
LQAAIEAGDDPTQQGEATAAGPGAGAGGAGGAGGGHSFVMLSEVGGALDPVIGFPTGPLAAAPLFPEGEVFAPADFSPELTVVFLDKEGSVVTGPGVVDE
AALNGGENGGGQPGSNPGSDAETTSGTLIINSPDGIGRIEVLDASGNWVNVTGGGVVQGAYGVLVFDAAGNWTYTLTDNSLDHSNPNAIGADDQLFDNFS
VRVFDLDGDVSPTVPLIIAINDDGP

P16 repeat
SAGLEFSREGGEVVLDESAGLQPGSHDVAGPLAVFNGVANPSSDMRAYAQGTAPAVVANYNFGADEGGATAVYSLALVGSGEGEQGPQVQAVGLTTTSGS
PITLSVENGLVVGRDAGGNAAFAISIDPDTGIISVVQYQSLHHPDASDANDSIDLSGLIRAVLTVTDGDGDQAVASADIGQLIRFVDDAP

P17 Repeat
TAAIERVEGVRVTHDESAGLQNGIATPTPGGDANDNDTNAGSVVGLFSSVSNTGTDLSSPGYATSNGAVVDISGSSTGEDNEGATTVLSLSIVGGDGSDS
TAVIQRVEGVRVTHDESAGLQNATETPTPQGDARDDDTGNPDVIALFAGVSNVGTDLE--GYAASAGPVVSIAGTSTGEDNEGATTVLSLEITGGDGTDS
TANIQVVEGVRVTHDETPGLQNGTATPTPGSDANDNDTNTPSVIALFSSVTDAGTALTT-GYASSNGPVVSTSGSSTGEDQEGATTVLSLEV---QNADS

GLTTTDGQTIRLYLENGLIVGRVDGGETSPGAAAFAIAIGQDGSISVAQYISLHHPDTGSADDAVSLAGKVRAVVTVTDGDGDVDTAQVGIGGAIRFEDDGP
GLTTTNGQVIRLYMENGMIVGRVDGGDQGPGAAVFAVAIGQDGTISVAQYMSLNHPDTGSRDEGVDLGGLVRAVVTVTDGDGDVDVAKVNIGGAIRFEDDAP
GLSALDGSPIVLSVENGLVVGRVDGGIYN-GQAAFAVAIGQDGSISIAQYLPLQHPDTASHDEALNLSGKLRAVVTVTDGDGDVSVDSVGIGGVVRFEDDGP

P18 repeat
TATKVTATNVLDDEGLNGGINGGPGDVSGAHTSTSGNLGYDAGADGLKSIELSGPSTLGSENVTSTWDPDTNTLTISSVRGDLMTVVLTDLASGAYTVNLLK
PLMHTVDGTEDNITLNVGYKVTDGDGDTADGSLTVTINDDTP

P19 repeat
TLQVGALDLNSSVTFHGTDAGYSNSYGYYIKGEDGTPLSGKVIWANVHDQSSGDTFDLGDLDPASTGFFIIPNGGGNAGLLNGADVTFQLVGGKWQAFVGGT
PLIGADGANVLFSDATLNPGGSHLQDTGNAGNQNWEDKTDTSDYDYNDVSTSVTWGSTLQLQVDETHLEQGNSATATADFSGLFNVQPGADGLGSLDYELQV
TNPASGLVDTETGEDVVLFLEGDQIVGRVGDANGEAVFTLTVSADGKITLEQLRAIEHPTADPDEAAFLGSGHVELKLTVTDGDGDSVSGGLDIGKVISFRDDAP

P20 repeats
SISAGEIATGELEVDETNLLVDKTVDYSGAFTSSYGADGAGSISYKLDISASGADSGMKDSASGGNIKLYLEGGQVVGRVGGPAGAISFTVSVDPDGKVT
TITAGQAANGSLEVDESFLGVDATRDFSGVFTHSFGNDGAGTITYALAISAEGASTGLKDTASGSDIKLYLEGGEVVGRVGDQSGAIAFKVTVDAAGKVT
SIRATAIPADSLQVDETNLLGNATTDFSGAFIKAYGADGAGSISYKLDISASGADSGLKDTATGSDIKLYLENGEVVGRVGDQNGAISFKVTVDADGKVT
TISAGQAGVASLQVDETTLAINATTNFSGAFTSNYGADGAGSISYVLDVKSQGVDSGLKDSASGSDIKLYLEGGEVVGRVGDQNGAIAFKVTVDASGNVS
TISAGQAGVASLQVDETTLAINATTNFSGAFTSNYGADGAGTIGYALEVKAQGADSGLKDSVSGSDIKLYLEGGEVVGRVGSDTGAIAFKVTVDGSGNVT
TISAGQAGVASLQVDETTLAIDTTTDFSGAFTSNYGADGAGNIGYLLEVKAQGVDSGLKDTASGSDIKLYLEGGEVVGRVGDHNGAIAFKVTVNASGHVT

LDQKLAIVHSPNTGPDQATGLSAADLVKLVATITDKDGDSNSASLNLGNAISFRDDAP
LDQKLAIVHSPDSGPDQAAGLSAADLVKLVATITDKDGDPDSATLNLGNAISFKDDAP
LDQVRAIVHSPNSGPDQESSLGAADLVKLVATITDKDGDSNSATLNLGNAISFKDDAP
LDQVRAIVHSPNTGPDQATGLSAADLVKLVATITDKDGDSNSASLNLGNAISFRDDAP
LDQVRAIVHSPNTGPDQATGLSAADLVKLVATIIDKDGDSNSASLNLGNAISFKDDAP
LDQVRAIIHSPDTGPDQATGLSSADLVKLVATITDKDGDSSSASLELGKAISFKDDGP

P21 repeat
KITGFELQGGGRVYVDESVGTAGSSRDENGYVAPNDEQGHGNVLGYARIQGSDLFNLATNGGSDGLDASRTQFALSLVSEGVDSGLDATAGGNILLYTDS
NGNVLGKVGLDTIFKISVNTVDGSITLEQYKAIAHGNTASHDELALIREGLVKLGVTVYDKDGDSDSASLDLGKVVGFEDDGP

SVIQPDCTEVEESNGFTTQGVIQADYGSDGGGFDLSANAGLTDDGLFYTVVKENGVTTLTATVGDAQGEVFFVLKVYDDPNVLGGGNNYELTIVNARPVT
IADFDLQAIAAGQPQASFLVQSGEGLAVTLTGSGLVNPSEQGVGVGGNNLINGAESLTMAFSATVYSAEVSVQKLSSSDQLSWTALDGNGQVVAAGTYNG
QGSENSQFSFDLADSAFITGSAAALAGGFTTLIFASNDGDYRIQYITLEQQFLPPSLDLSFQVDVVDGDNDVASTQLDVCFVNDEPTGGFVFDRVDEDGL
PGGIPGGVQDDEGATTLVTGTLGYDYGDDGFGSFAWQPSGLPSVTSGGQAVEYQVSGNGQVLTAQTVGSHQPVFTVTLTNPATGAFTFELHAPLDHPAPA
SGSVENNLDFQFAYQMVDGNGSTAQGTLHISVDDDSPAQPKDIAKSASEPQGIHTNLMVVLDLSGSMDDAPSGVSGFSTKLALAKDAVQRLIDSYDNLGD
VMVRIVTFANTASAVGNVWMTASDAKAWLTALANNAGNGSTNYDDALIKAMNAYDSTGKLTGTGVQSVSYFLSDGQPTLSNANPGSNNSGSQYNPELGDG
IGAGEEADWIAFLTAKGIKSYALGMGLGSDLDKTLLNPIAYDGKAGVNTDGILVSNLNQLNDTLQGTVTGTVISGNLISEGSNGFGADGPGVLPVAAITH
NNVTYTSASAAYDAASHTLTFTTAGGGSFSVNLLTGAYTYSFNGDVAENRTDTIRYTLVDRDGDAATAELKLTITDSSEVHAYDNYSQAIVGQTWVTPDP
VTKVLADFSSTSNPASSGPNYNPWVFDTVNDFLPSGDERTVVTTNDWVNVAADKWGVSSIAVNNTSGNSGVNGVRVEGGQLQLRDTSSADGVSTKAITPS
FQIAEGSTGTLSFQVGQAATGSGDSFTWKLYSLSETNVWDEVSDVQSQTQGATISLLNLAGGTYRVYFEANDRSSTNQTRNSNQYRVTIDNITLVTLAAA
VLVDTATAVAGNVLTDPNNYLGSDQPWGAVDSKGSEGAVLSVLDNGNFVAVGTSKALVGQWGTLLIHGDGAYTYTPDPDYSNLGKEDVFTYQLTQPDGDV
STAHLVVGIGAGAAVQPNVLMGNEGLNDTLLGSEASDVLLGLGGNDTLRGLGGNDRLEGGDGNDTLIGGKGNDVLVGGIGNDTFVWTADDRGGNYHDIVK
DFGNGDDKLDLSQLLQGIEGPATADVLTQYLSFDFVSEPGSTVINVASAGSGTPVDQTITLENTVLSGGNAADIIQGMLDHNQLVA

Pse-67 WP_157719523.1
MARLIGVVKTIVGEVHAVAADGSKRLLEQGDRVYAGEQLLTGADGAVAIRLVNGEELTVGRESILTLDDQLLAGEEARARIEPSSSAEAPSSALLTDVEQ
IQKAIAAGIDPTTGLPATAAGPGAGGAGGAGAGGGGISFVLLGETADRVDPNIGYPTGPLPFAPLSPTEFPVDADELPDNGVIIDGLGVVGGEVRLDEDD
LPNGTDRSDPLTQNGSFTITALDGIATVSIAGITLTYAELANASTYPLVIDTPAGELVINGYSGTETGGTVSYTYTLQAPVDHAPGSDLAGESFPVVVTD
TDGSSASASLDIVIADDVP

P22 repeats
LVFAEPQQGLAEGATITGVFDFTAGADGATVTHINGIQLVFGADGFSQVIDTGNGLIKVKADGIYSFTADASVISREPVVDNLTFTVTDGDGDAVTQDAS
SATEEPLRVLDEGDSFSGVFDFVAGADGANVTHINGQALIFGQDGYSQAIDTGHGLIKVKADGSYSFTADASVLNPEIVTDNITFTVTDGDGDAVTENFS
SASDEGQRSLLEGTTVVGSFDFVAGADGASVTHVNGQALSFGQDGYSQQIDTGHGLIKVKADGSYSFTADASVISDVAVVDNLTFTVTDGDGDAVTKNAS

FSITDANAPTAGDARAAVDEDGLEGGNAASSAGDMVVSPDPDGNEATFSGTLPHDFGADGPGAITFIAMDGKIGTVGTETVTYSWNADSNTLTATGPRGA
IGILDANAPSAGKTAAAVDDDGLSGGNPVSTLGDLLVAPDPDLDEATFSGTLPHDFGGDGPGTISFASMQGETQAIGSETVRYSWDDDTSTLTAIVEGGG
FSITDANVPTAGQTFAAVDDDGLLGGNPASTQDDLVVTPDPDLNEATFSGVLVHDFGPDGPGSISFAAMDGKTATVGTETVTYSWNAANNTLTAIGPRGV

-----LFTVVVD-PLTGSYTVTLLQNVLHESLDGQLGDNSENEATADLIYYAVSDADDSTAQGTLSITFDDDMP
RDGLQLFTVAVN-PLTGAYTVTLLQNVLHAPLNGVAGDNTENDAIADLVYYTVSDADNSVARGTLSITFDDDMP
-----LFTVEVDDPTTGEFTVTLHDNVLHPSLDGQVGDNTENDATVDLIYYTVADADDSKAQGTLTITFDDDGP

P23 repeats
TQTVTANAGGTAAVTVSLDETTGPSDHYAAGEAADSYVNDDVPGALARATTTLSGGLLGLFNVSGSYGSDGVGALITEVSFQGVPAGGLATNLVATDGGA
TQTVTANAGGTVAVTVSLDETTGPSDHYAAGEAADSYVNDDVPGALARATTALNGGLLGLFTVSGSYGSDGAGSLSTAVTFQGVPAGGVATNLVATDGGA
TQTVTANAGGTAAVTVSLDETTGPSDHYAAGEAADSYVNDDVPGALARATTALSGGLLGLFTVSGSYGSDGAGSLSTVVSFTGVPAGGLATNLVATDGGA
TQTVTANAGGTAAVTVSLDETTGPVDHYAAGEAADSYVNDDVPGALARATTTLSGGLLGLFNISGSYGSDGAGSLSTVVSFTGVPAGGLATNLVATNGGA
TQTVTANAGGTVAVTVSLDETTGPSDHYAAGEAADSYVNDDVPGALARATTTLSGGLLGLFTVSGSYGSDGAGSLSTAVTFQGVPAGGVATNLVATDGGA
TQTVTANAGGTVAVTVSLDETTGPSDHYAAGEAADSYVNDDVPGALARATTALNGGLLGLFTVSGSYGSDGAGSLSTAVTFQGVPAGGVATNLVATNGGA
TQTVTANAGGTAAVTVSLDETTGHADHYAAGEAADSYVNDDVPGALARATTALSGGLLGLFNISGSYGSDGAGSLSTVVSFTGVPAGGLATNLVATNGGA

ITLIASSPTQLSGVDADGDTVFTIAIVNVGGALQLQTTLYEALDNGTDGLFDEAVSLLLEPGSTLSLQLQVTRTDGDGDTIVASDRVVLANNTTSAFSFDDDGP
ITLIASSPTQLSGVDADGDTVFTIAIVNVGGALQLQTTLYEALDNGTDGLFDEAVSLLLEPGSTLSLQLQVTRTDGDGDTIVASDRVVLTNNTTSAFSFDDDGP
ITLIASSPTQLSGVDADGDTVFTIAIVNVGGALQLQTTLYEALDNGTDGLFDEAVSLLLEPGSTLSLQLQVTRTDGDGDTIVASDRVVLANNTTSAFSFDDDGP
ITLFATGAGVLEGKDATNQTVFKIEIVNVGGSLQLQTTLYEALDNGTDSLYDEAVSLLLEPGSTLSLQLQVTRTDGDGDTIVASDRVVLANNTTSAFSFDDDGP
ITLIASSPTQLSGVDADGDTVFTIAIVNVGGALQLQTTLYEALDNGTDGLFDEAVSLLLEPGSTLSLQLQVTRTDGDGDTIVASDRVVLANNTTSAFSFDDDGP
ITLFATGAGVLEGKDATNQTVFKIEIVNVGGALQLQTTLYEALDNGNDSLYDEAVSLLLEPGSTLSLQLQVTRVDGDGDTIVASDRVVLANNTTSAFSFDDDGP
ITLFATGAGVLEGKDATNQTVFKIEIVNVGGSLQLQTTLYEALDNGNDSLYDEAVQLLLAPGSTLSLQVQVTRVDGDGDQIVASDTIVLANNTTSAFSFDDDGP

SPFTPDDVHVVLGIENPLPTTVTKSLNFAGAAGSDGVGSIEFTITDGQVFQDVDDNDLFLNNELLYLFYQGDNQHIVAKTADGDVAFSAQINPGGDVSIT
MFEGTFISNELITVVKDLSGIGGGNVPFKGLNIGTTQNPDLDGSDDVLVSSEILPLGGSAGTVNSNSTELGVGQGNEISNTEVIRYDLVTNLSVVDSPNN
KSYSFSGYQSDIYAFKQKISISGGGKDADFRLRIYEVTGSATNATSTMVGGGLGTISQIPLALLASEIVIYDAAGNVQSNANHVAQDGNGVILYNIGNDW
TFEIHSVDAQGNPEDFNALEIEGIESVNGTVSVDGTTTSFKLAEFSFGQSTDIQPVSFELPVTGFDGDGDAVTSNIDMTVYPDTKSIWGTESGETLTGTD
GQDYMFGYGGNDTLIGDPGDDILFGGQGNDTLTGGAGKDTFVWQADDVAAGAIDTITDFSKGFSMGGDRLDLADLLSGEHGHSGDIGNLLGYIDISTANL
GGTAAVDTVIRVSSSGNFDPAAGAGVGAIPVDQTIVLQDVNLLSAAPGGYGSSEADVITGMLGDGTLKVDTV

Vib-1 WP_119299450.1
MQSQVVTQPVTVTSLSGNVVVVNAQGQARIVNAGDVLQPDDIIITVNQSAIELQTTQGGVQIDENCVACLPEFSADGQPEVQAAPIQGQINLDLAQLDTA
NFDEQAIAAIQQAILDGVDPTTALEAAAAGAGAGGSANGGAITIDYNFLEVLASTAFDTQGYNQTFSTTQTLVNPLRFAAGGESLSTQVTEGSLSLGTYP
QTSTVTSLITAGSLALLPASFVPEAAFLTSLLAELNQDITSSGQPVVFRYDAATNSIIGEQNGSTVLSIAISAESIGRDVNLTITTTLSQPIDHLPSVGG
GLVSISDDQISIALQLTGTDSNGNVIQAPIDVVVAINDGSAPVMVDEPTLSLNENDLPAGSDGADPLTVSGQFDTQLGSDQVASYQIDPSTANPIAGLTS
QGDAVILGEPTLIDGNRVYQATAGGRDIFQLTLNADGSYQFVLQGTLDHAAGSDALTISLPIVAIDYDKDSSAPGNLNIEIQDDKP

Vi1 Repeats
IIIGAEPLTVAEQTLDTGSIGGGASLVADGNFTTTQGSDGVVSYRLDSLTDAVAGITSGGVAVTLSES-VD-ANGNYTYTATA--GGEPVFTLLLNQDGS
YFTNVTSLNVHENDLPQGSDVTKEPLTASGQFELVQGSDRVSSFTLDGSVNPVQGLTSNGVAVTLSAP-VDDGNGNLTYTAMA--GSVTVFSLTLNSDGT
YFTNVQGLYVHENDLPQGSDTDKEPVTVNGQFQLVQGADTVASFALDSSVNPVQGLTSNGVAVTLSAP-VDDGHGNLIYTAMA--GPVTVFTLTLNTDGT
YFTNVQGLYVHENDLPQGSDTDKEPVTVNGQFQLVQGADTVASFALDSSVNPVQGLTSNGVAVTLSAP-VDDGHGNLIYTAMA--GPVTVFTLTLNTDGT
YFTNVQGLYVHENDLPQGSDTDKEPVTVNGQFQLVQGADTVASFALDSSVNPVQGLTSNGVAVTLSAP-VDDGHGNLTYTAMA--GSVTVFTLTLNTDGT
YFTNVQGLYVHENDLPQGSDTDKEPVTVNGQFQLVQGADTVASFALDSSVNPVQGLTSNGVAVTLSAP-VDDGNGNLTYTAMA--GAVIVFTLTLNSDGT
YFTNVQGLYVHENDLPQGSDTDKEPVTVNGQFQLVQGADTVASFALDSSVNPVQGLTSNGVAVTLSAP-VDDGNGNLTYTAMA--GAVIVFTLTLNSDGT
YFTNVQGLYVHENDLPQGSDTDKEPVTVNGQFQLVQGADTVASFALDSSVNPVQGLTSNGVAVTLSAP-VDDGNGNLTYTAMA--GAVIVFTLTLNSDGT
YFTNVQGLYVHENDLPQSSDTDKEPVTVNGQFQLVQGADTVASFALDSSVNPVQGLTSNGVAVTLSAP-VDDGNGNLTYTAMA--GAVTVFTLTLNSDGT
SFIHHQPLSVNEDDLPQGSDGSKESLSASGQFTVVQGADQVVSYQLDSSVNPVQGLTSNGVAVVLSAP-VDDGNGNLTYTATA--GGNPIFTLVLNTDGS
TITAVQALSVDEDDLTQGSDTTKESLTATGHFTTTQGADHVVSYTLNLTTNPLAGVTSGGQVLTLSDS-VD-THGNHTYTATK-PDGTLIFTLQLNANGS
SVISAQALSVNEDDLATGTDQSKESTTANGQFTTTQGADGIAHYQIDTSTSSDTGLTSQGQPVVWGAPSITTTSSGQVYTYQGIANGVVIFTLVLRADGS

YRFTLQSSLDHALNSDELLVNFTVIATDFDGDTASITLPVTVKDDKP
YSFTLSAPVDHALNSDSLTLNFKVIATDFDGDSDSIVLPVKINDDKP
YSFTLAAPVDHALNSNDLTLNFKVIATDFDGDSDSIVLPVKINDDKP
YSFTLAAPVDHALNSNDLTLNFKVIATDFDGDSDSIVLPVKINDDKP
YSFTLAAPVDHALNSNDLTLNFQVIATDFDGDSDSIVLPVKINDDKP
YSFTLAAPVDHALNSNDLTLNFKVIATDFDGDSDSIVLPVKINDDKP
YSFTLAAPVDHALNSNDLTLNFKVIATDFDGDSDSIVLPVKINDDKP
YSFTLAAPVDHALNSNDLTLNFKVIATDFDGDSDSIVLPVKINDDKP
YSFTLSAPVDHALNSDSLTLNFKVIATDFDGDSDSIVLPVKINDDKP
YTFTLSGPIDHASGSDSKQLNFKVLAVDFDGDKASVNLPVTIIDDKP
YQFTLSGPLDHAASSDELLLNFNVVATDYDGDISSIVLPVTVVDDQP
YSFTLNGAVDHPLNANELTLNIPVLAQDADGDTSPITLPVTIVDDVP

ILHDKNIALQEGSVASSVNLFSRDNNLSPDTQGADRGVITHFSAVDEAGRDIQFREGSVLSNDIELNGAAKTVTVVEIVNGVSRDLGTLTIQPNGTATFT
PVTQLDHTDGNDIKFTVDVTATDYDHDTSTEQLNITISDHKATITQQKFTGYEDQGHDAALNLVPAGEQSNAQDNLGGLPVEALKLALQVNLYDVDQGES
LGEVSIWNPNQIRGDFYYLDSANQLVKLDVDPASGHVVLPAALLQQSINGTIATVENLYFVPDRHYSTGNGGMNASVSVEILHNGVRDHFTNGNMRIEIE
SVADIATWKSSSEFHYDAVEDGSHVSLNIAAETQDNSNPEALTYQIRFTENGANANLVYSDGSPIPTKTDANGTYYEVPANKIAQVQVDPADNFAGQIKL
DVTVITKESANYVAGKQTAQSETKEIVIDVAPEADRGSFTVNRISIFEDNASNQNAVDPSVEHDPLLLSEVISMTGSSDADGSEALFVRLSDFTDTGATL
VWLGSGPSPITVGTYPNGETYYEIPQSALSQVEVLPTKHSNENFSFVVEGIVKDTVNLSTGQVQDIESLGSKTVNVTVKGVADLPTIEFLNGNTQWQTFN
DGVQSGVITTVSEDSRVDLNFSIISGEIADSPTDSSETISVLLSNIPDGVKLFDSDGTSVDLVFAGYDSNHKPIYQANLTVAQVVTGIQVQPVASSTANI
DIKATVIVTENDGHVRQVEETIRILVEPKIDVTENYHNAVSGNEDDRIHVTWVPQNTPGNIQNPDAQEYFSRVEISGFPDGSRVFVNNVEVTLINGVLVL
EPAAGQSDLDFSNQVSAAGYIQVIPPHNSSTDFTLDTTITVKEQDHEYVDAANPGQGIAEEVIHGSIGVKVNPIVEPDGQLLVENAGSVTQTVQADPSGK
IDFTINDASGGQAGANVIRFDNLDSNTAGSYQSDELVDQLVVSFGNVPQEVLNQLLITGAINNGDGTWTITNEADFRIKAPNGLVYSSNNDPDNNGFNDI

KITITAKVYDQGEDSSEVKITKQVSTELTLSFPTVVTGNNSVAAQLNWVGDADDLVIGKEDNTVNLGQQIQDKLMVNATGFDAVADELSIVINASDLPAG
ASIGGQDFNFVDGQYVFKGTLNPDGSISGLEGLVLIPPRDFAGDFKLPITFVTTDTQSGDEKTLTAQVPVAISPVADVPSSSGDQPLDNHVTPSITLNVQ
ETLGLDANHQPTDLANDTPTQDGIAYEDGIVHLNLAIGLADSLNGSTQGQEVLTEVTLTLNDTNSGVFVDANGQSLGTSITLTQAELPAVLSEIYFKPAP
NYPSGNDINTVGITVAGKVTDSTVFDETNASSQGVSSSDADKTFTSQVSFEVKPVVDDITIGSGSPISVTGDEDSWIALADQGNAFNVSLNDNDGSEQFV
SLVLTGLPTDFLVKSLSSDYVVKNNGGGEWSVQIRNPNLTSLDLSALAIKPAKDFSGEVQLGIKVFTQESLLNQPVEHSDQFTLHVTPIGDDVDITPAQE
VAGNEGQAIDISLGAQILDKAPSLPGGANYTENSPETLRVEISGVPDGAFLSLADGTLGTSLGGGVWVFEINAQQLDKVVFNSGDNNQLNWNGNLHFKVQ
SVDTGLAGDQHLGSAQEFDVHVDVTAVNDRPELINVQDQVTEEDTPLLLNSFTLADIDAQLDDPNADYTLQIGVNSGVLIIDSSLSSGLTTQGDGTGALS
ITGNVAEINAAIGAGLVKFVPSPDFYGQVAVTLNVNDNGNAGIEIAGDASTAHDNSAQFVIDVTAVNDKPEVDGIHLTAQIDEASGQKLTGITVSDVDYA
GSHTNDVMKVTLSISEGILSVQAPAGSSVAVSYALDGSVILEGSPEAINALLNHSDSAYGLFVGAAAIAGTQINLTVTAQDMGVYFENASGMALEESKTY
PIQVNPVANAPSLSMNPAFGYAQQIYANQSVSAQGIALLGAIAALTDLHETLSLRVDHLPAGASLSSTAGSVTDLGNGRREVSPDALESLKVVGLEEGVH
TLSLTALSTESDGSSAPSANSIDYQIEIAADGSLLDHRSATDDSLLVADNSGMTLLSGSGDDFVQGGAGDDVLVGGLGADILVGGTGADMFKWTLDGVDD
KVDHIRDFNVSEGDSIDLIDVVQDLGNHLTMEQLLNNLGVSNQLTAQVVDNDVTLQVTTDNQVQQTIVIENLATQIDFTGMSSLDIIGTLLDQNLLRHD

Vib-7 WP_025442669.1
MSTQTVPQNAIVNAVKGEVLVLDLTGKIRVVSAGDALNTGDVIVTENNASLDVLINNELYLVDQNCVACLPEPSSEQPETLVQTPVDGQIAFDPTAIDSA
NFDANDVAAIQQAILEGADPTAILEATAAGGDASGSANAGYVTVEYNNPEMLASTFFETSAARTGVQDREETDDINVTIFADGGQSLESEVTEGSISLTT
YPQSIATSISVEAGDLPLDPSSFVPSAASLESLLAELNTDIQSGGKAVEFSYDEAQNAIIGVQDGNEVLRIEIEATSLGRDIELEVTTTISQGIDHVASV
DDGQVSIVGDSISIEFEMTGTDIGGNSIRTPIDFVTTIIDGDNPAPQDISFENEESSSTPITGTFVEIGSDQLASVTFNQESLSQFDGLLTDNQATVATL
SDDGSNITLTVAGTGEVVLSISLNTDGTYQFEQFKPLEQTNDSDTIALSLPTTIVDYDQDTVSNTFVITIADGDNPVINNVTSLSLDESGVEQGSLQGIA
ITSGTGSISAAAGSDIIDHFEVEPTEFNVSGELQSQGQTVLLELTSDVNGVRTYEGYIELDGVRITVFDISIDSPSQGEYQFNLYEQLDHTGANDESLTF
TIPVYAVDADGDRSSLTLGSNDAKAAEIVIEVKDDAP

Vi2 Repeats

SIIGVEALTVDEDDLASIGSDQNDSVSVDGKFTTTEGSDRVVSYQLDASTNPIDGLTSHGEAVELVE--TANADGSFTYTA-TANGNS-VFTLVVNV
TIENVEPLTVDENDLITNGG-QSNALLVEGQFTTTQGSDGVVQYQLETGSDPLNGLTSNGQVITLAE--VSNADGSFTYTA-TANGNP-VFTLQVNS
VVTDFEAISVDEDDLAGIGSDQNDPVSIDGQFTTTEGADRVVSYQLDSNATPVDGLTSQGVAVTMTE--TANSDGSFTYTA-TAGTNP-VFTLTVNP
TITNVEAISVDEDDLANIGSDQNDSLTIDGQFTTTQGSDRVVSYQLDTSVDVVAGLTSQGNPVTLIE--TANPDGSFSYVA-AADGNP-VFTLVVKT
VITNVDAIQVDEDDLTGIGSDQSDALSINGQFAITQGSDGVVSYQLDSSADPVAGLTSHGEVVDLVE--TANADGSFTYTA-TANGNP-VFTLVVNV
TITNVDAIMVDEDDLSGVGSAQDGVVSIDGKFTTTEGSDRVVSYQLDSSTDPVTGLTSHGEAVVLVE--TANADGSFTYSA-TADGNP-VFTLIVNV
TITNVDAITVDEDDLTSIGSAQDGVVSIDGKFTTTEGSDRVVSYQLDGSTNPVAGLTSHGEVVDLVE--TANADGSFTYTA-TANGNP-VFTLVVNT
TINNVQAITVDEDDLAQIGSAQDGSVSIDGHFTTNQGSDGVVSYQLDASAMPVDGLTSQGVAVTLSE--TANPDGSFTYTA-TAGTNP-VFTLIVNP
TIENVVPLTVDENNLVTNGG-QSNALLVEGQFTTTQGSDGVVQYQLETGSDPLNGLTSNGQVITLVE--VSNANGSFTYTA-TANGNS-VFTLQVNN
VISAVDAINVDEDDLVNVGSDQNDPVSIDGKFTTTEGADRVVSYQLDSNAKPVDGLTSQGNPVTLIE--TANPDGSFSYVA-AADGNP-VFTLVVKT
VITNVDAIQVDEDDLTGIGSDQNDAISINGQFAITQGSDGVVSYQLDASADPVAGLTSQGVAVTMTE--TANPDGSFTYQA-SAGGNA-VFTLIVNV
TITNVDAIMVDEDDLSGVGSAQDGVVSIDGKFTTTEGSDRVVSYQLDSSTNPVAGLTSHGEAIVLVE--TANADGSFTYSA-TADGNP-VFTLVVNV
TITNVDAITVDEDDLTSIGSAQDGVVSIDGKFTTTEGSDRVVSYQLDGSTNPVAGLASHGEVVDLVE--TANADGSFTYSA-TANGNP-VFTLVVNT
TINNVQAITVDEDDLAQIGSAQDGSVSIDGHFTTNQGSDGVVSYQLDASAMPIDGLTSQGVAVTLSE--TANPDGSFTYTA-TAGTNP-VFTLTVNP
VITNVAPITVDEDDLINIGSDQKDALSIDGKFTTTQGSDSVVSSQLDASAMPVDGLTSQGVAVTMTE--TANADGSFTYTA-TAGANA-VFTLVVKP
EIKTATPLFLDEDDLPSGSELSKDSLAASGSFDSVEGADTIVSYQLDLSGNPISGVTSGGQAVTLVQ--TGVNNNNYTYQGQTPDGKS-VFTLVLNA
TLDGIDANSVLTVDEDDLPTVGSDGNEPTSIIGKFVATEGADHIVEYHIVDLNTPVQGLTSGGQSLTLVE--VSNSGGVSVYEAIIDGTTTPAFRVTLDV
TIDSLAQGSEQTVDEDDLP-LGTDRTQDTIIGGTFDVTEGADQVTSIQLSDLTTPVSTLMSGGEAITLVL--TSSTGGVNVYQGITVNSQEVVFELTLNA
VVTNIDRLQVHEDDLPLGSDETKEPLTVSGQFEVTS-ADGIDSFVLDLNSNLLPALTSGGESIAITQDATASTSDALVYVGKTVGGDT-IFTLTLHQ
TIEAIRPGSHLSIDEDDIPNKGSDGQGDHIIGGHFDVVDGADSIVSFQLEDLVSPVAGLTSAGQPLELVE--YSNANGVIEYRAYVQGTTDIVFKLTLNA
SITGVDNSSQLTIDEDDLP-AGSDTSGLRVLDGHFNVVAGADEIVSYHVSDLAGAVAGLQSNGQDVELRL--VSETDGVSTYEAVIVGTNTQIFTLTLDA

-DGSYNFTLEGTIDH--ASGSDALTLNFPITATDFDGDTSTM-VLPVTIQDDVP
-DGSYSFELQGAVDH--APNSDTLTLDFSIIATDFDGDTSKI-TLPVTIVDSLP
-DGSYNFTLEGAIDH--AANSDSLTLNFPITVTDFDGDTTSA-VIPVTITDDQP
-DGSYNFTLEGPIDH--AINSDELTLNFPIIATDFDGDTTSA-TIPVTIVDDKP
-DGSYNFTLEGPIDH--ASGSDELTLNFPIIATDFDGDTSSA-VIPVTIVDDQP
-DGSYNFTLEGPIDH--AINSDELTLNFPIIATDFDGDTSSA-VIPVTIVDDQP
-DGSYNFTLEGPIDH--ATGSDELTLNFPIIATDFDGDTSSA-TIPVTIVDDQP
-DGSYNFTLEGPIDH--ASNSDELMLNFPIIATDFDGDSSVM-VLPVTIQDDVP
-DGSYSFELQGAVDH--APNSDTLTLDFSIIATDFDGDTSQV-TLPVTIVDSLP
-DGSYNFTLEGPIDH--AINSDELTLNFPIVATDFDGDTTSA-TIPVTIVDDKP
-DGSYNFTLEGPIDH--ASGSDELTLNFTIIATDFDGDTSSA-VIPVTIVDDQP
-DGSYNFTLEGPIDH--AINSDELTLNFPIIATDFDGDTSSA-VLPVTIVDDQP
-DGSYNFTLEGPIDH--ATGSDELTLNFPIIATDFDGDTSSA-TIPVTIVDDQP
-DGSYNFTLEGPIDH--ASNSDELTLNFPIITTDFDGDTTSA-TIPVTIVDDQP
-DGSYNFTLEGTLDH--PIGADELTLNFPIIATDFDGDTTSA-TIPVTIVDDRP
SDGSYKFELLEPLDHPTANGQNDIVINLPIAATDFDGDVSNTLTLPITVVDDVP
SNNSYEFDLRKPLDHPDGNQQNNIIIELPITVTDGDGDVSPVFTLPITVVDDVP
-DGRYDFELSGALDH--ATNSDDLTINLPIVITDGDNDSVNA-TLPVTILDDKP
EEDRYQFELFAQLDHPNGNGENELVIDFPVNATDFDGDVSNTISLPITVVDDVP
KDNSYQFELVGPVDHPAGAGENSLTLDIPISVTDFDGDTSASVNLPITIVDDVP
-DGTYKFTLEGVLDH-DVQGEDLLTLNLPVFATDVDGDTAGI-NLPVTITDDVP

TIYDSSITRVEGQGTRTVQLFQDPVEGDLNYGADGSELTSFSADDSGIYFKQNGIDMTTVDLNGSNQTVFVHKTLNGVDTEIGRLIVRTDGSISFRPNDD
LDHTDAASIDFTVHVTATDGDGDTSTADLDISVTDRNAQIDTSSVLSFEDKGRDGSILGTENANTQDNLSGLDTTPAKVELVINLHDLDRNESLGDITIR
DASTHNGTFYYRNANGEYIELTPVNGSVVLDGSNVIQSFNGEFVTLENLYFVPDRHFATGDSGIDPRIRVEILNNGVSDHIINGRLNIQVESVADIATWT
ANSTFNYTVDEDGNNVSLNISAQTQDSSNPESIVYELVFTQGEGNATLVYSDGSAIAQTGGVYLVDASRIGDVQVDPIDNFSGEIKIDVTAITTESVNPL
TGKETARSETETIVIDVTPVADAGSFTVNRINIFEDNARTQDTVDPVTDHDPLQLSEVISMKPSSDMDGSEELFVRISNFSIDGVTLVWLDGANPSQIVE
VTDGSGNVLYYEIPESHLTNVEVLPPLHSNDDFSFNVEGIVKDKASLSTGSAQDVLSLGNKTVIVDVKGVADIPIVELNDKSGIWQEFDDGNVRGVQTFV
DENGQVDISFSILSGEFKDNPNDHSETVTVLLSNIPDGVEIFDNDGNSVDLTFVGYDGNNQPIYEANITQANINSGIVIKPEASSTENIHITATTIVTEN
DGHTRTSTGEIRVIVGPVIDARNNYTVVSEGDEDTRFNIDWKPTTSQSPDADEFFSEVTISGFPPSCTVFVDGVAQTLVAGTLTLTPQANESEQDFSARV
TQSGYVQVELEQDSSADFDLSTTLTVKEIDHEYVDAANPGQGIAEAVITGSVHVQVNPVVEPEDTSGAIGDQTRLLVTESNGTAIDVVKSDAQGAIDFTI
NTSAGGQAGANIIKYQEFDASSDEVVTELVVQLHTTDPAILNQLVIIGALNEGDGRWTIIDEENFCIKAPSGLDLTPNDDTDNGDNGGLSQIGLTIYARV
NDLGEDSVEKDATEIRQTDVVLEFPTVLTPQTSVAAEIDVTDDVQIEGSEDNFVDLGAQLTSKIDVINPDGVEDVLTIIIDPSSPGIPPGLIITGTDVDF
INGKYVFQADIDASGNIVGLEGLTMRVAEDYAGDFVLPVRFVTKDTGSADEKSSTELIPVQILPVADVPSSAGDQPLDRNITPDVTVDITGTLGLDANKQ
PVDDLNNDVPTVDGIGYEDGLIQLNLNVDFADGFNNTLGGRETLTNIKLTLDDTTQGEFVDSNGNSLGTSIEFNEADILAGALDNVLFKPSENYPVGGGQ
NTVKINIEGEITDVAVFDQSTLINPGDNVDVRTFTDDVTFEVTPVVDDIVITGTDPSQPIVVTGDEDTLISLNQSGTGVTISLTDDDGSESFVSLKLTGI
PNDFVVESNSSDYIVKNAGNGEWSIQVKDLTQTSIDLSDIQIKPPKHFSGEVEIGISVFIQEELLKVPTERNSNFTLVVNPIGDDVDVNPDTQVSGNEGE
DITINVNALVVDNKESIGDGANYQENDPETLRIEMSNVPDGASLSLPDGTTFVDQGSGVFVLEINAQDLDQIVFNSGDRNDNSWAGSLHFKVQAVDTGLD
GSQSLGNAEEFDVSVDVTAVNDRPDFVNVVDVETPEDNALLLNSFGISDVDAVLDNPNAEYVLNVAVDSGYLALNANVISKYGLTVQGDGTGAVELKGSV
ADLNAAIAEGLIEFNPDLNFFGDVTVNITVDDQGNEGVVISGVDDTLNTNSSSFVIDVTAVNDAPETSPVTLTSIGEDSGVFAISASDLLVNATDVENDN
LTVSNVQLVDPSSGSLAFNSATGNWEFTPAPGYNGPVELTYDITDDGTTNGVSDPKTVSGSASFEVFDVNDAPQTSEVTLSSIEEDSGAVSITASELLAN
ATDPENDNLAVSNVTLADPSAGTITQISAT-EWRFEPSENFNGDVSFVYDITDDGTTNGAPDPITISGSAVMNVQAVNDAPEIDGSLVTSTIFESAGQKI
SGITIADVDFTGIHENEIMTISLSTSEGDVSVIAPSGSGVTQGVGLAGETVLMGTLSQLNAVLTSTDPNVGVFVDASDVNSSAISLTVTADDNGIYYENS
TGTSLQTTETFDINVTPVADKPTLAFDANFNYIQRITASQSASFQGVALVGIIAALTDLDEVLALEVTGVPRGATLTSDATTSSISFDSATSTWTVPADE
IDTLHIDNVRQGDHDITLTAVSTESNGDQAFSDPIDISINVTSNNRDIDVSSETEDNLLLGSDRGISLIGGAGDDRIIGGDGDDILIGGLGSDILTGGGG
NDIFKWTQDTVDEGAVDTITDFTLNEDTIDLKDVIADLNDPMAGIDELLAHIQADYDATTDNVSLSITTDANVHQTIVVENLGQAIDFNGLSSNEIVESL
LNRGVIDNG

Rhizobium
Rhi-1 WP_064253643.1
MAIAITSAGAFVVLDETEDLQNATATPSPAGDADDNDTSNPLPAAFSTALSSHGVVTFAEAALSGHNGAAGNTGANIITVTGATATTDFAFRGENGAAFT
AYEAGATSTLNSGLSAVAPDGTITEIYLFADPDDNNIVYGVAGDSGDDPIVFAIYLEEVKNASNITIGAKMWTVLADGYTLAHTTDDHDESLDLADKLFV
AAVAENDFSFANAPSGQNLFMMFGNTSLAILVTGEDPADQSAGQNVSNDGDTVNTGQGGGATTLGTEGQQIKAQKALVVTFVTGANSNFLVPGLTPTEAN
LESNIAFSGYAQDVTGAAFTISQMTPGNANTTATVELQAFLTADASGTGYIDNNPLTTGDAAVAIASVQVIRGGVDVTGTLGVNVTISGGIATITGVKDD
DIIQYTTTDDHNRVLIRNDQPASGQGSNVSFDLGGFTITQVSSDVEEVGSKVRFEDDGP

Rs1 repeats
AIDLTSLAPTIAVDESVGTGGSTKDEPGGAAANDEAAAGAPAGAIGYAVTAAATLFSETTEPGSDGEDGTAYSLVLTAGATGLTDIATGSAVVLSLVGGV
AIDLTSLAPRIGVDESVGTGGSAKDEPGNAQPNDETTAGAPAGAIGYKVTAAATLFSETADAGSDGQDGKAYSLVLNAGATGLTDIATGSAVVLVNNGGV
AIDLTSLAPTIGVDESVGTGGSAKDEPGNAQPNDETTAGAPAGAIGYAVTTAATLFSETVDAGSDGQDGKAYSLVLNAGATGLTDIATGSAVVLVNNGGV

VEGRSATGNHLVFTIAVASTTGNVTTTLYRALSHGADSNDHDSAVSMASGLVELQATLTDGDGDTGSDKIELGSLIDFEDDGP
IEGRSATGNHLVFTIAVASATGNVTTTLYRALDHGADSNDHDSAVSMATGLVELQATLTDRDTDTASDKIELGSLIDFEDDGP
IEGRSATGNHLVFTIAVASATGNVTTTLYRALSHGADSNDHDSAVSMASGLVELQATLTDGDGDTGSDKIELGSLIDFEDDGP

AVTVADSNGNYDDGAQGTWDDNNPGSDGFASLSVNFDSYEIDDDGLITVDTALTKTGNFTFTGSITDDFNGDGTDETVGFTLTFDPVNDTYDLDVTTPPP
TTRTFDTSQGSLKAGGPDAVQTLLFGGSEAGADDIVFFGVVATAPIQGVSSPPTNDIEDLVVEGAGDLTEAQIEGLFPIPSLINPSTQMNVSTSGIGINN
NNLNGASEGAGTGAFAGTSITSGDESFVVNPETVVDKVTVFIDNSVGGYNPATEELYFTVYYTDGTVQAATKVTAGMLTPVTSGVAAGGFSFEIDGGAKQ
IDAVQLTMGEGTIKIPVIAFSVEQVFDPEPLQLDFTATLFDGDGDSSSDSFSIDLIEAVV

Rhi-4 WP_113478518.1
MAITITPTGAFVVLDETDGLQNEAATPTPAGDADDNDISSGSLPTAFSGRLSFYTLAVDEAALSGYNGTTSSNIITVTGATASSDFALTGEDGVAFTAYV
SGATSTFNSGLSAVADDGTITEIFLFTDPTNNNIVYGVAGDSGDDPIAFAIYLEEVKTSGITTGAKMWTVIADGYTIAHDVDGSTVADHDDFLDLTDKLF
VSAIGENNFSFANAPSGQNLFMMFGDTSLAILITGEDPANQSAGQHVSNDGDTVSSSQGGGPTTLGTEGQHIKAQKALVVTFVTGANADYIAGPNAGNNP
GQPLSPTEANVEANIAFSGYAQDVLGAGLTISQMNPGSSNTTATVELEAYLTADGTGTNYINNNDPLVTGDTQINIASVQVIRGGIDVTNTLTITLSGLG
ATITGVKDNDVIQYTTDSAHNRVLIRNDQPSGSNVSFDLGGFKITQVASDVEEIGSKVRFEDDGP

Rs2 repeat
NAVAKNVAGPTATVDESPLPAAGDGVNTATIAAATIAALFEAPAFGADGQGSVSYTLSGTDNAQTGLWLTGESGAANEIRLFKVSDTLWEGHKGVVGGTL
AFTVSINSSTGEVTLTRSSATLEHTTDGSSAAAHDDALTMAATANLFVVQHITDGDGDTDSATAVNPLTLKFEDDGP

AVTVNDTSGTYAAGAQGTWSDDDPGSDGFASLSVTFDGYEIDANGAQPGSALTQTGLAFDGSITDDFNGDGIDETVEFTLTFNRAANTYDLEVTTPPTTI
ITFDTSQGSLKAGGPDAVQTLLFGGSEAGADDIVFFGVVPTAPFENGTPPPATNDIEDLVVVGATDLTEGQIEALLPLPGLITPGTQMNVSTSGIGVNNN
NLNGADQGAGTGAFAGTTITSGDESFVVNPEKVVDKVTVFIDNSVGSYNPATEDLYFTVYFTDGSVQAPVKVTAGMLTPVTSGVASGGFSFEIDGGDKQI
DAVQLTMGKGTIKVPVIAFSVEQAFEPEPLSMDFTATLFDGDGDSSSDAFTIDLAETVV

Rhi-5 WP_113174696.1
MAIIITPTGESVVLDETDGLQNLTATPSPAGDADDNDILSSSLPTALSSRLTFYGLTLDESALSGYSGAAADTGDNILNVTGATATSDFSLTGSGGAAFP
DYEAGATSTFSSGLSALDDAGNITEIFLFTDPTNNNIVYGVAGDSGDDPIVFAIYLDEVKTSGITTGTKLWTALADGYTLAHNTDGTTVADHDDSLDLTN
KLFVAAVAENDFSFANAPSGQNLFMMFGTTSLAILVTGEDPANESAGQQVSNDGDTVNTGQGGGATTLGTEGQQIKAQKALVVTFVTGANSQFLVPDLSP
GEATDETSIAFTGYAQDVLGGEITISQMTPGNTNTTATVEIEAYLTADGTGDNYIDNNPLVTGDAEVNIASVSVIRAGIDVTGTLGVTVTISGNTATITG
VKDDDVIQYTTDDDHNRVLVRNDQPAQGAGSNVSFDLGGFTINQVSSDVEEVGSKVKFEDDGP

TVTVNDISGTFTDDPQTGTWVHAPGEDDFKSLNVSFDSYEIDDNGPVAGSALTKTGDFTFTGSITDDFNGDGIDETVPFALTFNPDTDQYTFQMENPPTT
IIQRDTTQGALKAGGPDPVQTLLFGGSETGADDVVFFGAVANAPQSGVAGDPPPNDLLDLIGTGATDLTEDQIQAFLSPTNQIPTLINSATKMNVSTSGI
GINNNNLDGNGVLGIQAGDESFVFNPEEVVDKVTVYIDNSVGGYSPPTEQLYYTIYFTDGTISSNNLVSAADLHNAPRKDPTVPDVAEGGKFFEIDGGTK
QIDAVQLTMGAGTVKIPVIQWTIEQEFNPQPLHLDFTASLFDGDDDSSQDPFAIDLVDATV

Rhi-8 WP_072597184.1
MAIVITSTGVFAVLDETDGLQNATATPAPPSPSGDADDNDILSSSLPGPFSTRLTSYSLTVDEAALSGYTGAAGNTGANIINITGATATTDLALTGANGA
AFPDYETGATSAFSSGLFAVADDGTISEIFLFTDPTNNNIVYGVAGDSGDDPIVFAVYLEEVKTSGITTGAKMWSVLADGYTLAHTTDGSSVAAHDDFLD
LTNKLFVSAIAENDFSFANAPSGQNLFMMFGNTTLAILVTGEDPANQSAGQNVSNDGDTVNTGQGGGATTLGTEGQQIKAQKALVVTFVTGANSNFLVPG
LTPTEANLESNIAFSGYAQDVTGAAITISQMTPGSASTTASVELQAFLTADTSGTGYIDNSPLTTGDAAVAIASVQVIRGGVDVTGTLGVNVTISGGIAT
IMGVKDDDIIQYTTTGDHNRVLIRNQPATGQGSNVAFDLGGFTITQVSSDVEEVGSKVRFEDDGP

RS2 repeats
DAVAKNVAGPTATLDESPLPTSGDGVNSVTIAATTIAALFETPDFGEDGQGSVSYTLSGTAGAQTGLWLTGESGAANEILLVKVSDTQWEGRKAGAGGTL
DAVAKNVTGPTATLDESPLPTAGDGVNSVTIAATTIAALFETPDFGSDGQGSVSYTLSGTDGAQTGLWLTGESGLANEILLVKVSDTQWEGRKAGAGGTL

DAVAKNVAGPTATLDESPLPTAGDGVNSVTIAATTIAALFETPDFGADGQGSVSYTLSGTAGARTGLWLTGESGLANEILLVKVSDTQWEGRKAGVGGTL

AFTVSINGTTGAVTVTRSSATLEHTVDGSSAAAHDDALTMAATANLFVVQHVTDGDGDTDSATATNPLTIKFEDDGP
AFTVSINGTTGAVTVTRSAATLEHTVDGSSAAAHDDALAMAATANLFVVQHVTDGDGDTDSATATNPLTIKFEDDGP
AFTVSINGTTGAVTVTRSAATLEHTTDGNSAAAHDDALTMAATANLFVVQHVTDGDGDTDSATATNPLTIKFEDDGP

AVTVDNSSGTYDAGAQGTWNDNDPGSDGFESLSVNFDSYEIDGDGLVTVDAALTKTGEFTFTGSITDDFNSDGADETVGFTLTFDPVNDTYDLDVTTPPP
TTRTFDTSQGSLKAGGPDAVQTLLFGGSEAGADDIVFFGVVATAPIEGVSPPPPSANDIEDLVVVDPINGTDLTEAQIEGLFPIASLINPSTQMNVSTSG
IGINNNNLNGASEGAGTGAFAGTTITSGDESFVVNPETVVDKVTVFIDNSVGGYNPATEDLYFTVYYTDGTVQAATKVAAGMLTPVTSGVAAGGFSFEID
GGAKQIDAVQLTMGQGTIKIPVIAFSVEQVFDPEALQLDFTATLFDGDGDSSSDTFSIDLVEAVV

Rhi-9 WP_072597182.1
MAIAITSTGAFVVLDETEDLQNATATPSPAGDADDNDTSTPLPAAFSTALTSHGVVTIAEAALSGHNGAAGNTGANIITVTGATATTDFAFRGENGAAFT
AYEAGATSTLNSGLSAVAPDGTITEIYLFADPDDNNIVYGVAGDSGDDPIVFAIYLEEVKNASNITIGAKMWTVLAEGYTLAHTTDDHDESLDLADKLFV
AAVAENDFSFANAPSGQNLFMMFGNTSLAILVTGEDPADQSAGQNVSNDGDTVNTGQGGGATTLGTEGQQIKAQKALVVTFVTGANSNFLVPDLAPTEAN
LESNIAFTGYAQDVTGAAFTISQMTPGNANTTATVELQAFLTADASGTGYIDNNPLTTGDAAVAIASVQVIRGGVDVTGTLGVNVTISGGIATIMGVKDD
DIIQYTTTGDHNRVLIRNDQPATGQGSNVAFDLGGFTITQVSSDVEEVGSKVQFEDDGP

AITVDNSSGTYDLGAQGIWSDDPGSDRFDSLEVTFDGYSIGGSAVTVDTSLGTKNETDVNGNYVFTGSITDDFNGDGADETVGFTLTLDPNDPDDPDDNT
YDLDVTTPPPTTRTFDTSQGSLKAGGPDAVQTLLFGGSEAGADDIVFFGVVATAPIQGVSSPPTNDIEDLVVEGAGDLTEAQIEGLFPIPSLINPSTQMN
VSTSGIGINNNNLNGASEGNGTGAFAGTTITSGDESFVVNPETVVDKVTVFIDNSVGGYNPATEDLYFTVYYTDGTVQAATKVAAGMLTPVTSGVAAGGF
SFEIDGGAKQIDAVQLTMGQGTIKIPVIAFSVEQVFDPEALQLDFTATLFDGDGDSSSDSFSIDLVEAVV

Rhi-10 WP_018325016.1
MAITITATDELVILDETDGLQNATATPSPAGDADDNDILSSSLPVAFSGRLTFYGLTLDEAALSGYSGAVADTGDNILNVTGATASTDFALTASGGAGFP
DYETGATSTFSSGLSAVAGDGTITEIFLFTDPTNNNIVYGVAGDSGDDPIVFAVYLDEIKTSGITTGAKLWTVLADGYTLAHNTDGSTVADHDDSLDLTN
KLFVAAVAENDFPFAGAPSGQNLFMMFGTTSLAILVTGEDPANESAGQQVSNDGDTVNTGQGGGATTLGTEGQQIKAQKALVVTFVAGANSEFLVPGLVP
SEATDEASIDFTGYAQGVRGAEITISQMTPGSASTTATVEIQAYLTADGTGNNYIDDDPLVTGDTKVNIESVHVIRGGIDVTDTLTVTISANTATITGVK
DDDVIQYTTTDDHNRVLVRNDQPTQGPGSNVAFDLGGVTITQVSSDVEEVGSKVKFEDDGP

TVTVNDIFGTFTEDPQTGTWDHAPGEDGFKVDGDGFLNVTFDSYEIDANGPVAGSALTKTGDFTFTGSITDDFNGDGDDETVGFTLTFDPDTDQYTFVMD
NPPTQVIQRDTTQGALKAGGPDPVQTLLFGGSDANADDVVFFGAVATAPASGVTGNTPPNDLLDLIVNGAPDLTEEQIQAFLTPTNQIPTLINGGTQMNV
STSGIGVNNNNLDGTATGIQAGDESFVFNPEEVVDSVTVYIDNSVGGYTPPTEELYYTVYFTDGTVSSNNLVTAAILQNAPRKDPNIPDVAEGGKFFVID
GGTKQIDAVQLTMGAGTVKIPVIQWTIEEEFNPQPLSLDFTATLFDGDDDFDSDTFSIDLVDATV

Rhi-11 WP_092586626.1
MAITITPTGELVVLDETDGLQNATATASPAGDADDNDFGGVVPTALSSRLTFYGVAPDEAALSGYSGAAGDTGDNILNVTGATLDSDFALTGEGGAAFP
DYGAGTSSFSSGLFALDDATGTTTEIFLFTDPTNNNIVYGVAGDGGTDPIVFAVYLDEVKTSGITTGTKLWTALADGYTLAHNTDGTTVTDHDDSLDLT
NKLFVAAVGEDSFSFEKAPSGQNLFMMFGNTSLAILVTGEDPANESAGQQVSNDGDTVNTGQGGGATTLGTEGQQIKAQKAMVVTFVTGADSQFLVPDL
TPGEATDETSIAFTGYAEHVRGASITISQMTPGGSTTTATVELAAYFTTDAAGDSYINDDPLVTGDTEINITDVNVIRAGVDVTGTLSVNYDGNTAVIT
GVKDDDIIEYTTDADHNRVLVRNDQPEKGSGSNVSFDLGGFTITQVSSDVEEVGSKVKFEDDGP

TVTVEDLDGTFTDAVQTGGIWDHTNSEDDLNSLNVNFDGYQIDAHPAVVVDAALTKTGGFTFDGSITDDFNGDNVDETVPFTLIFNPDTDAYTFQMENPP
TTIIQRDSTQGALKAGGPDPVQTLLFGGSETGADDVVFFGAVATAPQSGVAGDPPPNDLLDLIVTGATDLNEDQIQAFLSPTNQIPTLINADTKMNVSTA
GIGINNNNLDGNGTLGIQAGDESFVFNPEEVVDKVRVYIDNSVGGYSPPAEQLYYTIYYTDGTISSNNLVSAADLQNAPRKDPTVPDVAEGGKFFEIDGG
TKQIDAIQLTMGAGTVKIPVIQWTIEQEFNPEPLHLDFTASLFDGDGDSDEDAFAIDLADATV

Rhi-13 WP_126056483.1
MTTNIDSDNVSASFDERAVSTEHAGLTPAQEIQVAQAASTGQAAPADPVPVDVGSGAPVQPEAPAAAANPPANGAPAAAANAPAGAAHDYVADASNVVRL
PANVSIDNIKVDGHNLVLVQPDGSEIVIKDGALNVPTFILGEVEVPRVALIAALEASHVDVAFGADGSISAGPGNGSPNSAGGNFSVPPGGIGDGFDLSA
LLPPTALQFGQLDRRELFPSIVDRKVSVVAVSLLTPDEAASETGLDGGPSQSPGSDAPSNSETSSTGTITIDAPDGIGSIVINGVTVTGVGQQIQGQFGY
ITIVSFNPVTGAIEYNYTVTESVTHPDHTNGYDPNDTVPDNFSITVTDSDGDTASTTFAVAIIDDVP
Rm0 repeat
TAVADTDAVAAGSFAPVAGNVMDGSGSDGNAAGADTKGADDATVVGVAAGNTNTDVDNAATLNTPIQGEYGKLTLHADGSYTYVRDAGTPGGVNDVFTYT
IKDGDGDTSHTTLTISIGNSTPEISDLTPEANGGDVIVNENDLLASRGPDESAGSDTSKESTTQGGTFTINSPDGIASLAIDGHTFITNGTFTGGSFTTA
LGNTLTVTGYDAGTGVVSYTYTLNDNEAHATAQDTNSLFENFTVTLTDEDGQSANGTLSVNIVDDVP

Rm1 repeats
AASAEASQDVAEGATVTGTLDFVAGADGATVTHIDGTLLVFGQDGYSQAIDIGDGSIKVKAEGSYSFTADNPVIGTGAASATYTVTDSDGDTATAGISFA
TASAEGSQNVAEGATLTGTLDFVAGADGATVTHIDGTLLVFGQDGYSQAIDIGDGSIKVKADGSYSFTADSPVNGTGAASATFTVTDGDGDTATAGISFA

VTDANHPTTGTAAASVDDDGLADGNAASTTGDIAVPNTDGDNNEATFSGTLGGSVGGDGAGANGFSFAALNGTTGTVGQENVAYSWDAGTHTLTATTTSG
VTDANTPTSGTAAASVDDDGLAGGNAASTTGDIAVPNTDGDNNEATFSGTLGGSLGGDGAGANGFSFATLNGTTGTVGQENVAYSWDAGTHTLTATTTSG

DRIGTALFTVHVTDPATGAYTVALLDNVLQAQGPNDENNASVDLGYVITDADGSTAPGTLTITFNDDAP
DRIGTALFTVHVTNPATGAYTVTLLDNVMHTGGPNNENNATVDLGYVITDADGSTAPGTLTITFNDDAP

Rm2 repeats
SLTVGTVNESAITLVTQDAQTIGPASDTASASFAAAFLAAVTPSYGADGAGSTVISGYTLNVTNSASGLTSQGEAITLAKVGNDIVGSTASHGEIFRIAV
SLTVGTVNESAITLVTQDAQTIGPASDTASASFAAAFLAAVTPSYGADGAGSTVISGYTLNVTNSASGLTSQGEAITLAKVGNDIVGSTASHGEIFRIAV
SLTVGTVNESAITLVTQDAQTIGPASDTASASFAAAFLAAVTPSYGADGAGSTVISGYTLNVTNSASGLTSQGEAITLAKVGNDIVGSTASHGEIFRIAV
SLTVGTVNESAITLVTQDAQTIGPASDTASASFAAAFLAAVTPSYGADGAGSTVISGYTLNVTNSASGLTSQGEAITLAKVGNDIVGSTASHGEIFRIAV
SLTVGTVNESAITLVTQDAQTIGPASDTASASFAAAFLAAVTPSYGADGAGSTVISGYTLNVTNSASGLTSQGEAITLAKVGNDIVGSTASHGEIFRIAV
SLTVGTVNESAITLVTQDAQTIGPASDTASASFAAAFLAAVTPSYGADGAGSTVISGYTLNVTNSASGLTSQGEAITLAKVGNDIVGSTASHGEIFRIAV
SLTVGTVNESAITLVTQDAQTIGPASDTASASFAAAFLAAVTPSYGADGAGSTVISGYTLNVTNSASGLTSQGEAITLAKVGNDIVGSTASHGEIFRIAV
SLTVGTVNESAITLVTQDAQTIGPASDTASASFAAAFLAAVTPSYGADGAGSTVISGYTLNVTNSASGLTSQGEAITLAKVGNDIVGSTASHGEIFRIAV

DANGTVTLTQSQQIDHLPESLDTTNNNAHIDLGSGLVTLSATATVTDGDHDQATSTVSTDLGGNIGFDDDVP
DANGTVTLTQSQQIDHLPESLDTTNNNAHIDLGSGLVTLSATATVTDGDHDQATSTVSTDLGGNIGFDDDIP
DANGTVTLTQSQQIDHLPESLDTTNNNAHIDLGSGLVTLSATATVTDGDHDQATSTVSTDLGGNIGFDDDVP
DANGTVTLTQSQQIDHLPESLDTTNNNAHIDLGSGLVTLSATATVTDGDHDQATSTVSTDLGGNIGFDDDIP
DANGTVTLTQSQQIDHLPESLDTTNNNAHIDLGSGLVTLSATATVTDGDHDQATSTVSTDLGGNIGFDDDIP
DANGTVTLTQSQQIDHLPESLDTTNNNAHIDLGSGLVTLSATATVTDGDHDQATSTVSTDLGGNIGFDDDVP
DANGTVTLTQSQQIDHLPESLDTTNNNAHIDLGSGLVTLSATATVTDGDHDQATSTVSTDLGGNIGFDDDIP
DANGTVTLTQSQQIDHLPESLDTTNNNAHIDLGSGLVTLSATATVTDGDHDQATSTVSTDLGGNIGFDDDVP

TVLDKTDLYFANSGTVSGTGVFDYSIGADSHSTYSSLNSDFAAITLAGTVAGNAITAPTVTWASETSTQAVFNISFSYLTGGATTQETGTLTFDKVAGTY
TVDLADPISAVTISTVSNSSSITGYQPGSSTTDNTQPEVAVAQVNSNLFIQFTGYAEPGNGTGANNLQAGSVDGSTLTFVNGELITQASTYVSISGTANG
VAGDTMGKGEVMDMDFFTTNPTGFTGLTPDAQVSSMFLKFDGIGNTEDFIVILKLYDPTTNEYTTKAMYVENADIFKGPGSGPGAYSSVTLDNNDGLLII
ESNDYNAAGQHYVLVGAQITPTDEGISGTAINLNGAIGAGGASTTTQDISSDSNDLGFKISDIGLVSTTTTAQNADLTFNVTLKDADNDTTTAQQLDVHV
TNGVTYTGTADAETMQGTANGDTLNGNGGDDILQGFGGNDLLIGGAGQDTLTGGTGADTFKLDHLDIKDLIVDYSGVGGEGDKIDLTALFDTAPGGGNIG
NFVNYDAGTGTLSVDTSGSGNPANFVEVAQLTNHPAANTITLLYDDGTTTHTTTANVV

Rhi-14 WP_126092540.1
MTTSIEFDAVSDSRDEHSTVGEHPVTAGVQVAQAASGQAAGEPVPVDVGNGAPAQGAGNAQNAQAADKPAADADAPAANAPAGNAPAANAAASNAPHEYH
AEAGNVVKLPANVSIDNIKVDGHNLVLVQPDGSEIVIKDGALNVPTFIIGDVEVPRVALIAALESSHVDVAFGADGSISAGGNAPGSAGGNFSVPPGGIG
DGFDLTALLPPTALQFGLLDRRELFPSVLDKGISVVATSLLSPDEAASETGLNGGPSQSPGSDAPSNSETSSTGTITIDAPDGIGSIVINGVTVTGTGQQ
IQGQFGYITIVSFDPNTGVVEYNYTVTESVTHPEHTDGYDPNDTVPDNFSITVTDSDGDTASTTFAVAIIDDVP

Rm0 repeat
TAVADTDSVAAGQIGPATGNVLVGGTDAGDANTTDGVADTQGADGATVVGVVKGTTNADLDDTSTLGVQIQGAYGKLTLHADGSYTYVRDANTPGGVNDV
FTYTIKDGDGDTSHTTLTISIGNSTPEITDLTPEANGGDVTVNEDDLLASRGSGESAGSDTSKESTTQGGTFTIHSPDGIASLNIGGHDFITNGVFTAGS
FTTALGNTLEVTAYNAATGEVSYTYTLNDNEAHATGQDTNSLFENFDVTLTDLDGQSASSTLSVDIVDDVP

Rm1 repeats
TASAEASQNVAEGATITGTLDFVAGADGATVTHIDGTLLVFGQDGYSQAIDIGDGFIKVKADGSYSFTADNPVIGTGAASATYTVTDGDGDTSTAGLSFV
TASAEAPQNVAEGGTITGTLDFVAGADGATVTHIDGTLLVFGQDGYSQSIDIGDGFIKIKADGSYSFTADSSVAGTGAASATYTVTDGDGDTATAGISFV

VTDANHPTTGTTAASVDDDGLVGGNPASTIDDINANIGDADGPASSEASFSGTLGGSVGGDGAGANGFSFAGLNGTAGTVGQENVTYGWDSGTNTLTATT
VTDANTPTSGSAAATVDDDGLAGANAASTTGDIDANAGEVPLSAS-EAIYNGTLGGSLGGDGAGTNGFSFATLDGTSGTVGQENVAYSWDSGTNTLTATT

TSGDRIGTALFTVHVTDPATGAYTVTLLDNVLHAGGPNDENAVDPTTSLTYTITDADNSSTTGTLTITFDDDAP
TSGDRIGTALFIVHVTDPATGAYTVTLLDNVLQAQGPNNEN--NATVNLGYVITDADGSTAPGTLTITFNDDAP

Rm3 repeats
TISAPGASASLTVDETNLAVNDTQAFASAFTSSYGADGPGTITYALGFNAGATGLVDTATNQAVVLSLENGQVVGRAGVGGPIVFTVSTDASGNVTLDQQ
SISAPGASASLTVDETNLAVNDTQAFASAFTSSYGADGPGTITYALGFNAGATGLVDTATNQAVVLSLENGQVVGRAGVGGPIVFTVSTDASGNVTLDQQ
SISAPGASASLTVDETNLAVNDTQAFASAFTSSYGADGPGTITYALGFNAGATGLVDTATNQAVVLSLENGQVVGRAGVGGPIVFTVSTDASGNVTLDQQ
SISAPGTSASLTVDETNLAVNDTQAFASAFTSSYGADGPGTITYALGFNAGATGLVDTATNQAVVLSLENGQVVGRAGVGGPIVFTVSTDASGNVTLDQQ

RAVVHPTSNPNEPVSLSADNLVTLTATITDKDGDSSSATLNIGQNLTFLDDGP
RAVVHPTSNPNEPVSLSADNLVTLTATITDKDGDSSSATLNIGQNLTFLDDGP
RAVVHPTSNPNELVSLSADNLVTLTATITDKDGDSSSATLNIGQNLTFLDDGP
RAVVHPTSNPNEPVSLSADNLVTLTATITDKDGDSSSATLNIGQNLTFLDDGP

TVLDKTDLYFANSGTVSGTGVFDYSVGADAHTTYSSLNSDFAAITLAGTVAGNAISSPTVTWASETATTAVFNVSFDYLTGGVSTHETGTLTFDKVAGTY
TVDLADPISAVTISTVSNSSSITGYQPGSSTIDNTQPEVAVAQVNANLFIQFTGYSEPGSGTGNDNLQAGSIDGNPLTFVNGEFITQASSYVSISGTANG
VAGDTMGKGEVIDMDFFTTNPTGFTNQIPDAQVSSMFLKFDGIGNTEDFIVILKLYDPTTNLYTTKAMYVENADIFKGPGSGPGAYSSVSLDNNDGLLII
ESNDYNTAGQHYVLVGAQITPTDEGITGTAINLNGAIGAGGASTTFQNLSSDTNDLGFKISDIGLVSTTTTAQNADLTFNVTVKDADGDTSTAQQLDVHV
VNGVTYTGTADAETMQGTANDDILNGNGGDDILQGFAGADTLNGGDGNDLLIGGLGHDTLTGGAGADTFKLDHLELSIKDLITDYNGADGDKIDLTALFQ
TGGASVTDFVKYDAGTGALSVDTDGAGAGATFVEVATLTPHPAAGTINILYDDGIHAQPQTTTV

Rhi-20 WP_126085826.1
MTTSIEFDTVSNSWDENATAGEHPISGVQVAQATGGQAAPAGEPVPVDVGSGAPAQGAANAQNAQGADKPAADANAPASNAAAGNAPAANAAASNVPHEY
HADAGNVVKLPANVSIDNIKVDGHNLVLVQPDGSEIVIKDGALNVPTFIIGDVEVPRVALIAALESSHVDVAFGADGSISAGGNGSPSSAGGNFSVPPGG
IGDGFGLTALLPPTALQFGLLDRRELFPSIAPDKGVSVVAVSLLSPDEAASETGLNGGPQQSPGSDAPSNAETSSIGTITIDAPDGIGSIVINGVTVTGA
GQQIPGQFGYLTIVSLDPNTGAIEYNYTVTESVTHPEHTNGYDPNDTVPDHFSITVTDTDGDTASTTFAVAIIDDVP

Rm0 repeat
TAIADTDSLAAGQVGPATGNVLVGGTDAGDTNTTDGVADTQGADGATVVGVAKGTTNADLDDASTLDVQVQGAYGKLTLHSDGSYTYVRDANTPGGVNDV
FTYTIKDGDGDTSHATLTISIGNSTPEISDLTPEANGGDVIVNEDDLLASRGPGESDGSDASKESTTQGGTFTIHSPDGVASLNIGGHDFITDGVFTAGS
FTTALGNTLTVTAYNAATGEVTYTYTLVDNEAHDPIQGTNSLFESFDVTLTDLDGQSANSTLSVNIVDDVP

Rm1 repeats
TASTEAPQNVAEGATITGTLDFVAGADGATVTHINGTPLVFGQDGYSQSIDIGDGSIKVKADGSYSFTADNPVIGTGAASATYTVTDSDGDTSTAGMSFV
TASTEAPQNVAEGGTITGTLDFVAGADGATVTHINGTLLVFGQDGYSQSIDIGDGSIKVKADGSYSFTADSSVAGTGAASATYTVTDGDGDTATAGISFV

VTDANHPTTGTAAAAVDDDGLAGGNPASTTGDINANIGDADGPASSEASFSGTLGGSVGGDGAGANGFSFAGLNGTTGTVGVENVSYSWDAGTNTLTATT
VTDANTPTSGSATATVDDDGLAGGNPASTTGDINANVGDADGPASSESSFSGTLGGSVGGDGAGANGFSFATLNGTSGTVGQENVTYSWDAGTNTLTATT

TSGDRIGTALFTVHVTDPATGTYTVTLLDNVLHAGGPNDENAIDPTTSLTYTITDADNSSTTGTLTVTFDDDAP
TSGDRIGTALFTVHVTDPATGAYTVTLLDNVLQAQGPNDEN--NATVNLGYVITDADGSTAPGTLTITFNDDAP

Rm2 repeats
TASVGTVNDAAITLVTQDAQTIGPASDTASASFAAAFLAAVTPSYGADGAGSTVISNYTLNVTNSVSGLTSQGEAITLVKVGNDIIGQTASHGDIFKISV

SLTVGTVADAAITLVTQDAQTIGPASDTASASFAAAFLAAVTPSYGADGAGSTVISNYTLNVTNSASGLTSQGEAITLVKVGNDIIGQTASHGDIFKISV
SLTVGTVADAAITLVTQDAQTIGPASDTASASFAAAFLAAVTPSYGADGAGSTVISNYTLNVTNSVSGLTSQGEAITLVKVGNDIIGQTASHGDIFKISV
SLTVGTVADAAITLVTQDAQTIGPASDTASASFAAAFLAAVTPSYGADGAGSTVISNYTLNVTNSASGLTSQGEAITLVKVGNDIIGQTASHGDIFKISV
SLTVGTVADAAITLVTQDAQTIGPASDTASASFAAAFLAAVTPSYGADGAGSTVISNYTLNVTNSASGLTSQGEAITLVKVGNDIIGQTASHGDIFKISV

DAAGTVTLTQYQQIDHLPESLDTTNNNAHIDLGNGLVTLSATATVTDGDHDQATSTVSTDLGGNIGFDDDIP
DAAGTVTLTQYQQIDHLPESLDTTNNNAHIDLGNGLVTLSATATVTDGDHDQATSTVSTDLGGNIGFDDDIP
DAAGTVTLTQYQQIDHLPESLDTTNNNAHIDLGNGLVTLSATATVTDGDHDQATSTVSTDLGGNIGFDDDIP
DAAGTVTLTQYQQIDHLPESLDTTNNNAHIDLGNGLVTLSATATVTDGDHDQATSTVSTDLGGNIGFDDDIP
DAAGTVTLTQYQQIDHLPESLDTTNNNAHIDLGNGLVTLSATATVTDGDHDQATSTVSTDLGGNIGFDDDIP

Rm4 repeat
SVTVTAAPATKVALDETATSSSVATIDTGAIVKGDDPDVSGSGYISTATSVGALVTGNAAFGADGPAASGNTTYALTVTNTNSGLTLTDGSAIVLQQLAN
GTVVGVVSGGAFDGQAAFAMTINAATGAVMVEQYLSLDHPAEATAANSFNSYDETLTLASGSLGVTVTVTDGDHDMATSNTADVSNQITFDDDGP

PTFTHIQNAIVANQDNNVVTGTHDLAFGADGETSIEITPLTNITGVTYLPVVHNVDGSSDLIAQVNGSNFFDLKVNPDGTYTFTLIESRPVADQTFNFAG
VAGSQGTSQFTLGDATFNAIDTNNNGSIGSTEILKPTSNGFGVGNGNLDTGEQFKVTFATGIDKLSFFVEHEAAGAFTMTYTTDTGLTGTVTATVDGWLT
IDPTGDFNSITFTVTDGKAKFDNFAYSKLILPSDQTFNFSVSGVDGDGDHSASQTLSVTTLGEHPAGTPINGTAGDDAIAGTSGNDTIYGLAGHDTLYGG
AGDDVLIGGLGHDTMTGGTGADTFKLDQLELNIKDLITDYHGAGPGGEGDKIDLSSLFDTAVGGNIVDYVHYDAGTKTLSVDTNGTTGGANFVDVAVLQN
GPAAGTINILYDDTNHVQHTATI

Rhi-24 RWC29680.1
MTTNIELDSFSGSLNEHSASTEHGDQTSPAEMQVAQANSTQQAAPAASEPVPVDVGGGAPVKPEAPAEAKAPPAASPHEYVADASHVVRLPANASIDNIK
VDGQNLLLEQADGSVIVIKDGALNVPTFIIGDVEVPRVALLAALEASHVDVAFGADGSISAGPGGSNSSAGGDFSVPPGGIGDGFDLSALLPPTALQFGL
LDRRELFPSIVDRQVTVTATSLLAPTEAASETGLDGGGAQSPGSDAPSSSETSGVGSISIDAPDGIGSIVINGVTVIGVGQQIPGDFGFLTIVSYNPSTG
AIQYNYTVTESVAHPEHTNGFDPNDTVPDNFSITVTDVDGDSASTTFAVAIVDDVP

Rm5 repeats
TAVADNATQATENAAVTVNVFANDVPGADGVNITDPTKVSYVAGSLAGGAGTVTYHNDGTFTYAPVAGEEGTVTFQYQIIDGDGDSSVATVTINLLKDST
TAHDDSGTQASENAAVTVNVFANDVPGADGVAIADPAKVSYVANSLAGGAGTVTYHNDGTFTYAPVAGEEGTVTFQYQIIDGDGDSSVATVTINLLKDST

PTIDVTPQAPDASGHSAVNEAGLAVRGIEPAGSGEIADNNGTNNSDPSETATGSLNITTGGDTIGHLYVTDKDNTQIEVTNAAGGILVHGQYGDLTITGT
PTIDVTPQAPDASGHSAVNEAGLAVRGIEPAGSGEIADNNGTNNSDPSETATGSLNITTGGDTIGHLYVTDKDNTQIEVTNAAGGILVHGQYGDLTITGT

PGTGYTYSYTLLDNSSGNTTHDDFAVQVVDTDGDPASTTLTINIVDDVP
PGTGYTYSYTLLDNSSGNTTHDDFAVQVVDTDGDPASTTLTINIVDDVP

Rm6 repeat
TAHADTDAVQSGETVTGNVETGTSTHGTGAADTAGADGIASIAWTGIAGTTVTGTYGVLTVDANGGYSYHANPSASGVDIFNYTITDGDGDTSPSTLTIT
VSDGTPQPVAATVKVDEAALDTSTTGDDIDHGTATGTNPSSAAETVTGTLTLGDPDSPHVTNIAGATSSAVGGIPVTVQGTYGVLQIDQDGHYTYTLMTP
FTTAPATDNGAVTEPGKDVFTYTVTDSFGNSSTSTISVDIIDDVP

Rm7 repeats
SVTASATQPPLTVDESNLGQNATASFASVFTPVFGADGAAAANSVTYALGFNAGATGLVDTATGQAVVLSLNGTVVEGRTAVSNELVFTVATDASGNVTL
SVTASATQPPLTVDESNLGQNATASFASVFTPVFGADGAAAANSVTYALGFNAGATGLVDTATGQAVVLSLNGTVVEGRTAVSNELVFTVATDASGNVTL

DQQRAVVHPNAADPNDAKSLSADNLITLTATVTDKDGDTATATANIGQNLTFLDDGP
DQQRAVVHPNAADPNDAKSLSADNLITLTATVTDKDGDTATATANIGQNLTFLDDGP

Rm4 repeat
SVTVTAASAVKAALDETATSSGVATINTGAIVKGDDPDVSGSGYISTATSVGALVTVSALFGADGPAASASTAYALAVTNANSGLTLTDGSAISLQLVGT
AVVGVVSGGTFNGQAAFAISINATTGAVTVEQYLSLDHPNEATTANSFNSYDETITLANGSLGVTVTVTDGDNDTATSNTADVSNQITFDDDGP

TLGAFTSGTIPNEVGSVAGLFSLVSGADGLDHFNITGPVIAGITYTTTTAADGTTILHANSGATEVFSLTVRSDATYEFDLVTPQATTIQKVNLLTLSAG
NANFAETADGSVEFTSSGTVNSSNTGFGVNNQFLDNGESFTMEFHTSSPTIVDTPAIVNPSYVDSVLFKGLNDSANNGTLTINWTAVNTSTGQTQTGSAV
WTDPQPTLLIDPTITFNQLTLSAVGSNSAKLRLDNADISKTILPSDVTLDFHIAAVDKDGDATPAQTLSIHEVAGDAGGNFTLPGFAGTVNDVIAGSTHI
DTITGGAGTDIADYTGSLLAVSINLADDGHANAAAFSTTPADATIGGGDATGDTLTGIEGLIGGSGDDYLYGNSGANYLAGGLGNDTLRGEGGADTLIGG
AGNDLLVGGAGSDTLTGGTGADTFRLENLDIKDLITDFSGVGGDGDKIDLTALFDSAPANVGNFVNYDAGTGTLSVDTSGSGNPANFVEVAQLVNHPAAN
TITLLYDDGVNQHTTTANVV

Rhi-26 WP_126062539.1
MTTNIEFDAVSNSWDEHNSAAENHISTGVQVAQATTGQAASEPVPVDVGSGAPAQGAANAPAANQPAADANAPASNAPASNAAQGNAPAANAPAANAAAA
NVPHEYHAEAGNVVKLPANVSIDNIKVDGHNLVLVQPDGSEIVIKDGALNVPTFILGDVEVPRVALIAALEASHVDVAFGADGSISAGGNGSPSSAGGNF
DHPAGDIGNGFGLTALLPPTALAFGQPEHRELFPGLLPDSTPTIGDLTPETTGGDTIVYEKGLPASSGSEGSGEQAAPGPDGDPSEHNTGTFTINSPDGI
GSVNINGQVISGAALANSANVHVYITTPLGTLTITGYDASTGQVSYTYTLEHSDQHPDGNGTNSTFDNITVTVTDTDGDVSLPGTLSVNIVDDVP

Rm5 repeats
TAHNDSGTQASENAAVTVNVFANDVPGADGVNIADPTKVSLVANSLTGSGTVVYHNDGTFTYTPAAGEQGTVTFQYQIVDGDGDPSVATVTINLLNDSTP
TAHNDSGTQASENAAVTVNVFANDVPGADGVNIADPTKVSLVANSLTGSGTVVYHNDGTFTYTPAAGEQGTVTFQYQIVDGDGDPSVATVTINLLNDSTP
TAHNDSGTQASENAAVTVNVFANDVPGADGVNIADPTKVSLVANSLTGSGTVVYHNDGTFTYTPAAGEQGTVTFQYQIVDGDGDPSVATVTINLLNDSTP
TAHNDSGTQASENAAVTVNVFANDVPGADGVNIADPTKVSLVANSLTGSGTVVYHNDGTFTYTPAAGEQGTVTFQYQIVDGDGDPSVATVTINLLNDSTP
TAHNDSGTQASENAAVTVNVFANDVPGADGVNIADPTKVSLVANSLTGSGTVVYHNDGTFTYTPAAGEQGTVTFQYQIVDGDGDPSVATVTINLLNDSTP
TAHNDSGTQASENAAVTVNVFANDVPGADGVNIADPTKVSLVANSLTGSGTVVYHNDGTFTYTPAAGEQGTVTFQYQIVDGDGDPSVATVTINLLNDSTP
TAHNDSGTQASENAAVTVNVFANDVPGADGVNIADPTKVSLVANSLTGSGTVVYHNDGTFTYTPAAGEQGTVTFQYQIVDGDGDPSVATVTINLLNDSTP
TAHNDSGTQASENAAVTVNVFANDVPGADGVNIADPTKVSLVANSLTGSGTVVYHNDGTFTYTPAAGEQGTVTFQYQIVDGDGDPSVATVTINLLNDSTP

TIAIAGGSDTSVNEAGLPARGGEPAGSGEIADNNGTNNSDPSETATGSLNITTGGDTIGHLYVTDKNNVQVDVTNAAGGILVHGQYGDLTITGTPATGYT
TIAIAGGSDTSVNEAGLPARGGEPAGSGEIADNNGTNNSDPSETATGSLNITTGGDTIGHLYVTDKNNVQVDVTNAAGGILVHGQYGDLTITGTPATGYT
TIAIAGGSDTSVNEAGLPARGGEPAGSGEIADNNGTNNSDPSETATGSLNITTGGDTIGHLYVTDKNNVQVDVTNAAGGILVHGQYGDLTITGTPATGYT
TIAIAGGSDTSVNEAGLPARGGEPAGSGEIADNNGTNNSDPSETATGSLNITTGGDTIGHLYVTDKNNVQVDVTNAAGGILVHGQYGDLTITGTPATGYT
TIAIAGGSDTSVNEAGLPARGGEPAGSGEIADNNGTNNSDPSETATGSLNITTGGDTIGHLYVTDKNNVQVDVTNAAGGILVHGQYGDLTITGTPATGYT
TIAIAGGSDTSVNEAGLPARGGEPAGSGEIADNNGTNNSDPSETATGSLNITTGGDTIGHLYVTDKNNVQVDVTNAAGGILVHGQYGDLTITGTPATGYT
TIAIAGGSDTSVNEAGLPARGGEPAGSGEIADNNGTNNSDPSETATGSLNITTGGDTIGHLYVTDKNNVQVDVTNAAGGILVHGQYGDLTITGTPATGYT
TIAIAGGSDTSVNEAGLPARGGEPAGSGEIADNNGTNNSDPSETATGSLNITTGGDTIGHLYVTDKNNVQVDVTNAAGGILVHGQYGDLTITGTPATGYT

YSYTLLDNTSGNTTHDDFAVQVVDSDGDPASTTLSISIVDDVP
YSYTLLDNTSGNTTHDDFAVQVVDSDGDPASTTLSISIVDDVP
YSYTLLDNTSGNTTHDDFAVQVVDSDGDPASTTLSISIVDDVP
YSYTLLDNTSGNTTHDDFAVQVVDSDGDPASTTLSISIVDDVP
YSYTLLDNTSGNTTHDDFAVQVVDSDGDPASTTLSISIVDDVP
YSYTLLDNTSGNTTHDDFAVQVVDSDGDPASTTLSISIVDDVP
YSYTLLDNTSGNTTHDDFAVQVVDSDGDPASTTLSISIVDDVP
YSYTLLDNTSGNTTHDDFAVQVVDSDGDPASTTLSISIVDDVP

TAHNDSGSVNEGSLLTVLAAAGVLVNDVRGADGATIDGVRAAGADTTTAVSGGVNTDIVGLHGTLHLNADGSYTYQSTAHSINANTTDVFVYTIKDGDGD
LSTTTLTINLTDVNDSPTVANTENWMSSDPSQQTSGTPSYPDGYPLLVNIPTDADGDNLVVTATGAIPAGVFYFNGVSYVALTTGTVLYNPSSGINLLDD
LVYKPTVTANDTVTKSLSLDVYDGTVHVGQTVGIHEVPPTSLPSDTSQIGNGNSPLTSGNDQVATLHLSQATVNAITADPHGSTIVVYTDFQESPFTTPI
DPSERNPGVYGDSSAGSHREQEVQVEVRIGTDRFAIVEDDTSPATFEQSWFYDPATGLMKATVDYDHIYLLDASGKATSTTLASYLITHPPSVNDTWTLS
YFDNDGGNYQARTVNFSFFSHDPGDPGITVNGDATLADQIYGTSGVDHLFGNGGNDVIIGRGGNDIIDGGAGNDILIGGLGKDTMAGGTGADTFKLDSLD
VKDLITDYNKGEGDQIDLTALFDKADNVALSNYVQYNSTTHTLSVDSSGSGNQANFVDVAQLTNAPAAGTINILYDDTNHVQHPVTI

>Rhi-29 WP_095084448.1
MTISFILNDEAVNDQTTGLQTGDNVDDAFTDTDVAYSALPASFRTYLESTLGLNSAFPASIGVATKTNSVTVNATAGSQLVGTTFTDGNGGTLNGDDSGL
NTLGGKDILLFADGNNTVIGRYDSDGNGIVNNLDAIAFVVFKQDAINAGATSDQVTFNIVTYVPIFHGGNPNPDDAVDLGNTLKLAASETLNFGFAGAPS
GSNLFMTFGDPNSTQIVVIGKDPLNQSEGGNISTKDVLNISQAGSTTSFGVNGNAINPTEGAYITYVSGTNTNFLVPNLDQNEADVEANIDFQNVVSATG
ASFTVNQTNPGIGPVTVKISAFTTAAESGVGFVDGLTNDTHVAITSFSLTNVVVKTGNTQYTPAASIDANGDLIITGLSSGDTVQWTTSGTHNRVLIENI
SNADSDPSNDNNTFDIGGFGIVTSNSASEFVGQQIVIEDDGP

Rm8 repeats
TAGIVQGAPTVAHDETAGIQADADDTTAPAVVALFAGVSNVSSDLSPTGYAQDPGAVVSSTGSSFGADQEGGTTAFSLAVSAAGVDSGLDTTAGTSIFLF
TAGIVQGAPTVAHDETAGIQADADDTTAPAVVALFAGVSNVSSDLSPTGYAQDPGAVVSSTGSSFGADQEGGTTAFSLAVSAAGVDSGLDTTAGTSIFLF
TAGIVQGAPTVAHDETAGIQADADDTTAPAVVALFAGVSNVSSDLSPTGYAQDPGAVVSSTGSSFGADQEGGTTAFSLAVSAAGVDSGLDTTAGTSIFLF
TAGIVQGAPTVAHDETAGIQADADDTTAPAVVALFAGVSNVSSDLSPTGYAQDPGAVVSSTGSSFGADQEGGTTAFSLAVSAAGVDSGLDTTAGTSIFLF

KEGDLVVGRIGGAAGAAAFAVAINSTTGVVSVAQYASIHHPTPGASHDETVNITDSALLAVVTVTDGDGDTATSSTGIGDAVQFQDDGP
KEGDLVVGRIGGAAGAAAFAVAINSTTGVVSVAQYASIHHPTPGASHDETVNITDSALLAVVTVTDGDGDTATSSTGIGDAVQFQDDGP
KEGDLVVGRIGGAAGAAAFAVAINSTTGVVSVAQYASIHHPTPGASHDETVNITDSALLAVVTVTDGDGDTATSSTGIGDAVQFQDDGP
KEGDLVVGRIGGAAGAAAFAVAINSTTGVVSVAQYASIHHPTPGASHDESVNITDSALLAVVTVTDGDGDTATSSTGIGDAVQFQDDGP

TITVPFDGDPGTPAIDHETLHNVLNATATGAFGYDIGKDSHNAAFYTGGGSDFVDTNTGLAGVQISLTGTVDNPGTPGITQAVATLTSEDVASATFTFS
FHYDKDPITAGVQDATAGGTLTFDKAADTYSITLTDPIDGFSFNVLHTSELLRKAPPGNTGHPELVVTELDLNNGTTDTNGFYVQFTANSNPAGSPFGF
NGTGDGAPDGPPLDTAYNNGQFVTSSFEDWVSATQSTNGVAGDTIQKNELLTLRFFNENILGDVNPGAPGGGTEKTTPTDSVSGVAIKFDGIGNSEDLI
VVLDLIDDKGTASTADDVKTTRSIIVENQDFFKLSTGGVPSPYNSEFTLDNNDALLIIESNDYNLAAGENFKIQGMQIMQSGNGLAGSAINLNGITDNP
NTIPIEGASSGTQAWEAQDNDVLKIVDIGFVQTTSGTLNANLDFAFQIADADADTTGTQHIPVDVVA

Rhi-30 WP_129553402.1
MTTSIEFDAVPNSTDEPSAVGEHPISAGVQVAQAATGQAAQPAGEPVPVDVGSGAAAQGTANAPAANAPAANTAASNVTHEYHAEAGNVVKLPANVSIDN
IKVDGHNLVLQQPDGSEIVIKDGALNVPTFIIGDVEVPRVALIAALEASHVDVAFGADGSISAGGNGSPNSAGGNFQVPPGGIGDGFGLTSLLPPTALQF
GQPDNRELFPSLLKPDSTPSIGTIGGASVNEAGLPARNGEPEGSDSASNSETTSGTIGFTSPDGVSEVTLGGHVLTGAPQTFTDATGSLTASYTYDPATG
TGSISYTYTLLDNTSGDNTSVSFAVGVTDSDGDATPPGSLVINIIDDAP

Rm9 repeats
TAVADTDVVPSGSHAAIDGNVITG--AGSDGNAAGADVKGADDVTVVGVAAGTTAGGEDANVGTVIQGTYGKLTLNADGSYSYTRDAGTPGGHDDVFTYT
TANLDTNSIASGQFGPVSGNVLANDVIGADGPATTSPAGG-----IVGVVAGTTAGGEDANVGTVIQGTYGKLTLNADGSYSYTRDAGTPGGHDDVFTYT
TANLDTNSIASGQFGPVSGNVLANDVIGADGPATTSPAGG-----IVGVVAGTTAGGEDANVGTVIQGTYGKLTLNADGSYSYTRDAGTPGGHDDVFTYT
TANLDTNSIASGQFGPVSGNVLANDVIGADGPATTSPAGG-----IFGVVAGTTAGGEDANVGTVIQGTYGKLTLNADGSYSYTRDAGTPGGHDDVFTYT
TANLDTNSIASGQFGPVSGNVLANDVIGADGPATTSPAGG-----IFGVVAGTTAGGEDANVGTVIQGTYGKLTLNADGSYSYTRDAGTPGGHDDVFTYT
TANLDTNSIASGQFGPVSGNVLANDVIGADGPATTSPAGG-----IVGVVAGTTAGGEDANVGTVIQGTYGKLTLNADGSYSYTRDAGTPGGHDDVFTYT

IKDGDGDLSHTTLTISIGDSTPTIGGLTPQAQGGDTTVNEKGLPASSGSEGSGELAAPGADGDPSEHNTGTFTISSPDGIAKLTINGTDISAAALANSGT
IKDGDGDLSHTTLTISIGNSTPTIGGLTPQAQGGDTTVNEKGLPASSGSEGSGELAAPGADGDPSEHNTGTFTISSPDGIAKLTINGTDISAAALANSGT
IKDGDGDLSHTTLTISIGNSTPTIGGLTPQAQGGDTTVNEKGLPASSGSEGSGELAAPGADGDPSEHNTGTFTISSPDGIAKLTINGTDISAAALANSGT
IKDGDGDLSHTTLTISIGDSTPTIGGLTPQAQGGDTTVNEKGLPASSGSEGSGELAAPGADGDPSEHNTGTFTISSPDGIAKLTINGTDISAAALANSGT
IKDGDGDLSHTTLTISIGDSTPTIGGLTPQAQGGDTTVNEKGLPASSGSEGSGELAAPGADGDPSEHNTGTFTISSPDGIAKLTINGTDISAAALANSGT
IKDGDGDLSHTTLTISIGNSTPTIGGLTPQAQGGDVTVNEDDLLAG---RGAGESAGSDTSKESTTQG-GTFNVSSPDGIATLSIDGH----AFITNGVF

THVDVTTPDGNTLTITGYDAGTGQVSYTYTLLDNETHS-GAGTDSIFDNMTVTVTDTDGDASSGTLSVQIVDDVP
THVDVTTPDGNTLTITGYDAGTGQVSYTYTLLDNETHS-GAGIDSIFDNMTVTVTDTDGDASSGTLSVQIVDDVP
THVDVTTPDGNTLTITGYDAGTGQVSYTYTLLDNETHS-GAGIDSIFDNMTVTVTDTDGDASSGTLSVQIVDDVP
THVDVTTPDGNTLTITGYDAGTGQVSYTYTLLDNETHS-GAGIDSIFDNMTVTVTDTDGDASSGTLSVQIVDDVP
THVDVTTPDGNTLTITGYDAGTGQVSYTYTLLDNETHS-GAGIDSIFDNMTVTVTDTDGDASSGTLSVQIVDDVP
TAGSFTTALGNTLNVTGYNAATGVVTYTYTLNDNEAHAAGQGTNSLFENFTVSLTDQDGQNATGTLSANIGDDVP

TAHNDTNSAQSGQTVTGNVETNDTAGADGIASIAWTGASGNTVTGSYGTLTIAADGSYSYHANPNASGTEVFTYTITDGDGDTSPATLTITVTNGQPQPA
AATQTVNEAALDTTTTGSDIAHGTVTGSNPSSTAETVSGTLSLGDPDSPHVTNIQGATSSVVGGSAVTVQGTYGVLQIDQNGNYTYTLTKPFDTSPDANN
GTNTETGKDVFTYTVTDAYGNTSTSTISINIIDDVRTAPVPDYAVLSNGAGNPVTFALDEDHTLTGNYGADGPGTVQFATSLDGVDSGLTSGGTHIIYDV

SADGHTLYGMANNVAVFTVTLDPTNATYSVDMNGTVDSVSQVTFSNGSYDFDGGNGAWAGFVPTGQKNSGTPVNDDSRDVLFTPFGTGTTINGNANSFGV
GGGSGGQNIGAGEGIRVDFVQDLTGNTMGAGGYGVPANRDHVFDSHYVANGATFTFGDGTSNTLLKITAFDDNATTGTDGNKTVGDGVHDSVTSVAITYD
GETQMVSFADIGTTATSYTVGNSGGLTDRTYTVHFVDLGDGKYAAEITGVLDTHVSVSTFTADGYSSLELLNASPTDDSGNDFAITGFGAAVQTTDPVNF
NLPIEIVDGDGDKATSEIGVTLYGTGVQDHSADAAGASHLYTSTVALPNIIGSAFNDTITGDVSANVLYGGAGADTINGSGGNDTLVGGAGNDIMNGGAG
NDLLVGGAGQDTLTGGTGADTFKLDHLDIKDLISDYNGAEGDKIDLSSLFETSGGNIADYVHYDAGTKTLS
VDVNGTTGGANFVDVAVLQNAPAAGTITILYDDNNHTQHSTTI

Rhi-31 ESY26103.1
MAIDITAHDIIIDESAGLTDNDIDPSDPLYSTNTTLQYLLNLGTALEVAFKEDFVVASADPGETITSVVLAQDSSGTAFSPTDGAVTGIRTVDGNYVWLF
QDPNNADVVIGVIGTSDPTSVGEPDPLLDPLAFSFALDGDADLYLVQYVALMNPDPDNADVDLIDLDQLVYASVTGTSVLNFSSLGDAPPGHNKWYILDA
DTASTQKILVTAHDNGVQAEVNVSTQGLGVSSQDVRFGRELQIDLINGGTQSAGKNFTNTPTAPNYTDHIETVSTAGFSISQSTPTHTVADIEVHAYNND
DNAKGATLPGDDNDAEVNIASVTFKLNGVVTDAATLGITVDYTNLGVILHNVGEGVTVDFTADGTFDRFTIKNIDAQKDYFDVKEVHFGTDQTNVASAEV
GSLINFDDDGP

Rm10 repeat
SIVPSGNDVPTLVVGDSDFVTDDSTSFAALFTPNFGVDGPAATDDVTYTLDVLGGGSNVASGVFDTLSGLEVLLNVVGGDIVGTAGTDEVFRISVDADGN
VTLDQSRAVVHGDPTDPVEADTPVILAANLVSLTATATDGDGDSAHTSVDIGGAFTFEDTGP

AIAVENATGDYDSGAHGTWTHDPDADGFNLLGVTLDDYTIDSNPKVTSGLSLTPDAADPYTFTGSIHDDFNGDGTAEDVDFTLTFDPVNGTYDLDVDTPP
GTITTFDTTQGSLKAGGPDAVQTLQFPPAGHDGDVIFFSNLVTAPLENGAGSFPNDIEDLVNNDPTEATLEGYGATSLINSSSKMNVSTSGIGVNNNNLD
GKSGGTPLFAGTSITSGDESFVVNPQQDVDKVTVYIDNSVGGYNPATEDLYYIVYYTDGTVSGPTDVTAGMLSNAPRHDPLIPDVAEGGKYFEIDGGAKQ
IEAVQLIMGVGTVKIPVIQFSLEQESNPAPLDLDFTANLVDGDNDSFTDTFSVHVDAPDGII

Rhi-36 WP_073986818.1
MATTITINGNLIIDEAASVQNSVTDSDAGVPAGPDNEVEWSTLLATMSQALQDELKALSLLPNAGETASDTTATFPQVAVRTIGSTSSDFIQLNTNTITD
LQFSDSNGNKIADGTATQIFATHNGAQIFLYTDASNNIVYGREGTWNGTTWVADTSGAVAFAIVMDDTTTGNNVTGGNVWTIQYEALKQPNTSGIDDADT
SSLAGLVDVKAIFTSTTTANFGDFSHVPSGSDEWAAIEQTKNAGTDANDPDMIITGLASGSKVTVSTQGLGNNSQALNSGEAVRVDVVNHVNYPDFPLTN
PDVHDLSKLSYTTHVDAVTEGSFSITQVNPGSSKTTVAVHIWAFNELSNLQGAALVSSASIGSGGLTGNDQTLVQIIQSSIKVWNNLTKTGNPLDLAANG
ITITAAPDGKSFIISGLKKNYTVDFQVNQSDHMDRFVVGNAQPSGSNVSFDAGNFTFNISSTTSGTEQTAVGGNLLFEDDGP

Rm11 repeat
TITASTTNEPALTVSEATLGTPSSGAFGGQFTVDYKADGPAASNPTTYALSTPGGDSGLIDTLTGQHVQLSKVGSQIVGTINAGATTVFTVSVDGTGQVT
LTQSRAVVQAIGSNPDTGEGIGLTGSNLVVLNATATDGDGDSASTSLDLTPQLTFTDVGP

TIAVNDIQNGTYTGGGNGTWSEAPNADGFKSLAVTLNDYTIDSHSTVTVNSLLGTDTTTDANGNYVFNGTINADFNNDGISDAVSFKLTFNPTNNTYKVD
VTSQPSTIITFDTSQGSLAPGGPSPVQQLNFSSGPAAGQSVVFFGAIPTADPGPTAGANNDIFDLVEVGQPDLTKAQIDALLKPTNQIPTLINGSTQMNV
STSGIGINNNNLDGSGANIQSTDESFVVNPTQLVDKVKVFIDNSVGGYDPGTEDLEYRVYYSDGTVSAYKKVQAGDLSPVTSGVASGGKSFEISDAIGGP
QIDAVQLTMANGTVKIPVVQFSIRQAFLPQQLAMNLTATLTDGDNDTNQDAFKIALA

Rhi-37 WP_040987538.1
MADAIEIAGDDTVDITLTNATSPVQGLGFVALDSNGQNAAPVDGLDSGQQTLDGNEIFLYTDPNNDNVLLGREGSGTTADSGGQIVFAVYLEEVTNQNNV
ITGGQLWTVLFEPLAHPDDTNSDDVVTLPDALGVAATGEQSFSFAGAPAGNNLFMAFGSSADALLVTGTSINHTVNSSKGGGATTLGTDAQDVRAGKGMY
FTYVTGMDPNFLAPNLSHQEATTVGDIAFTGLQDSDAASLTIVKLTGGTSVSVKLTAFTTDVESGANYFPGLGDADDDPVTITHVYINGTEVTFTTSGDG
VIVSGVHDGDVIKFETSGDHNRVLVENAEAAGSNIHFSIGGFSIDNAVTAAVPVGDHLEFYDDGP

AIAVADVADGDYTNGAHGTWTNDPGQDGFGSLGVTFDSFQIDSHGVVTADATNSTFTDNLDGTFDGSITADFNGDGKNDTVNFTLTLNSDDTYDLTVTTP
PTTTTTFSTDQGSLDAGGPDPVRTLTIGSEDVVFSAVKATTATADIKAFLNASEATIQGSATYLSPNQMNVSTAGIGLGDNLFEGNSIAGIDGATTSGGK
IDESFVVDPEGTVSSMTVIINNKTNGGYTPSNEDLFYRIYYSDGTVSADHKVVTGDLTVTSSTASFTVGDPNGPNDIDAVQLIMGRGTIKVPTITFSVST
EFSPQPLDVTFTAELFDGDHDSQTDPFTVHLDAA

Rhi-40 WP_052468903.1
MSLVIVTQDIIIDETASLQDDDINPTIAPYNANTTLQYLETLGSPIETAFQANFVQATASAGETIDSITLTQDAAGHPFSTTVGVNSGIQTVDGNYVWLF
RDPTHANVVIGVIGTSNPNAAPSDASGPLAFSFGLSGSGASADLYLVQYVPLFNPDPNNPDDRIDLTGKVFASVSGSTVVNFSQLGDAPPGHNQWYILDA
DTASSEKILVTAHDNGVQANVNVSTQGLGVSSQDVRFGRELQIDLINGGTQSAGKNFTNSPLAPDYDSHIETVTSAGFSVSQSTPTNSKADIEIHAYNTA
DNAEGASFPTDNTETESNISGVTFKLNGVTRTAAALGITMDFGGNGLILHNVGTGVTVDFTADATFDRFTIKNVDPQKDYFDVKEVHFGGKSTNAASAQV
GSFINFDDDGP

Rm12 repeats
SITASAINAPPLTVDETVLATDATGTFAAQFTPTFGADGQGATPVSYALSTPGGASGLTDTATGESVVLSLVNGQILGKTATTGLTVFVVSVDASGQVTL
SITASAINAPPLTVDETVLTTDATAGFAAQFTPTFGADGQGATPVSYALSTPGGASGLTDTATGESVVLSLVNGQILGKTATTGLTVFVVSVDASGQVTL

DQQRAIVHANPNDPNESRGLTGSNLVVLTATATDGDGDHASAPLDLTPLLAFKDDGP
DQQRAIVHANPNDPDETRGLTGSNLVVLTATATDGDGDHASAPLDLTPLLTFKDDGP

SITVDDIASGTYAAGGSSTWTDPPGADGFKSLNVTFNNYTLDAHSPVTVNSSLGTLTQTDANGNYVFNGTIIDDFTNDNKVNPQTVAFKLTFDAVDPGSG
TYNIQFITPPTSTTTLSTANGTLGAGGPDPVQTLTIGNQKIVFSAVKESALVGSIEANLDKTEAQIQTNPLPSYIDPASMNVSTSGIGVGNNNFDGNAVA
GVDGQTTSGGAYDESFVIDPTGFLVSSMKIYIDNSVGGYDPSTEGIFYRTYTRNADNSITAGSITKVLGGDLTSEAGGQKSFVIPSVDGKNDLDAVQLFM
GSGTVKIPVIEFNISNTFNADPISMNFTATIADGDSDTKTDTFKIALA

Rhi-41 WP_129411922.1
MANSFEIESNSASWNQQSMRAEHDDPSVSLQVAQATSGSQPAGQENQPAGAASEPKPVDVGATPAPTPAQAAATPPSSSGEYIVGADKTVHLPANTSIDN
IRVEGTNLVLQQADGSVVVIKDGAVDVPTFIVGEVEFPRVALLAALQTSGVDVAFGADGSISAGQGAASNPSAGGNFEHPAGGIGDGFDLSSLLPPTDLQ
FGQLDERELYPAAPNRDPLFADFSALALSDEGLAGGNPDNDGSSDTTNATLITGRFPATDPDGDALTYTFGQPSGLTSGGVPLVWTGQGTAQLIGSVNGV
PVITITAAGDSYTIQLLGPIDHPNDTRPGSEDDLTFTVPVTATDGKGGSTTANMSVTIEDDSPAFVGAGQSVLRVDEDGLASANADSGRSGENDFGKQTV
SHIELSTVINPGADGLGSYHFVSGALTQLAGLGLSSNGAAISYVVSPDGHTITGSAGGVSVFTVELTATGSLTFTLLGQVDHVNSPGNVETNLSIDLSGL
IEVSDKDGDTLTPPVGSVVVSVGDDIP

Rm13 repeats
VAALVLREGASILVDESLGQNGGPETEPAGTLGRVVVPGASLFNTAGSSVGSDSAGATKTFSLEIAAGGHTGLFLTGQ-SGAANEILLVDNGGVIEGRVG
TAVLALAGKASIVVDESLGQNGAPETEPVGTLGQVTVLGTSLFDTTGSLAGQDEEGATTAFSLEVAAGGHTGLFLTGQ-SGAANEILLVDNGGVIEGRVG
TAVLALAGKASIVVDESLGQNGAPETEPVGTLGQVTVLGTSLFDTTGSLAGQDEEGATTAFSLEVAAGGHTGLFLTGQ-SGAANEILLVDNGGVIEGRVG
TAVLALAGKASIVVDESLGQNGAPETEPVGTLGQVTVLGTSLFDTTGSLAGQDEEGATTAFSLEVAAGGHTGLFLTGQ-SGAANEILLVDNGGVIEGRVG
TAVLALAGKASIVVDESLGQNGAPETEPVGTLGQVTVLGTSLFDTTGSLAGQDEEGATTAFSLEVAAGGHTGLFLTGQ-SGAANEILLVDNGGVIEGRVG
TAALALKAGASIIVDETLGQNAGEAEGGAAGLGQVTVLGTSLFDTTGSSAGQDEEGATTAFSLEIAGGGQTGLYLSGAVHDAAHEIVLVDNGGVIEGRVG

GAGGTLAFTVSIDAATGNVVLTQYESLNHPTPGASYDEALFLATGALKAVYTVTDGDKDIASAAVDLGGQRGVIAFEDDGP
GAGGTLAFTVSIDPSSGNVTLTQYESLNHPTSGASYDEALFLATGILKAVYTVTDGDKDVASASVDLGGRAGVIAFEDDGP
GAGGTLAFTVSIDPSSGNVTLTQYESLNHPTSGASYDEALFLASGILKAVYTVTDGDNDVASASVDLGGRAGVIAFEDDGP
GAGGTLAFTVSIDPSSGNVTLTQYESLNHPTPGASYDEALFLATGILKAVYTVTDGDQDVASASVDLGGRAGVIAFEDDG-
SAGGTLAFTVSIDPSTGNVTLTQYESLNHPTPGASYDEALFLASGILKAVYTVTDGDQDVATASVDLGGAAGVIAFEDDGP
GAGGVLAFTVSINATNGNVTLTQLQALDHPDT-TNADEALYLSSNVLKAVYTVTDGDHDVSTAKVDLGGRAGVIAFEDDAP

Sequence between Rm13 and Rm14 repeats
TAVVGGAGIQATVAEDTMATADGDASNGIPDAGQTSSSDEASGTLGSLGALFNAGADAPLTFSLNPSTSGLPVLYSHGIQLVYNVAGGVLTATAGANTVF
TLTVNADGSWNFDLKDQLDHVAGSGDAGTLLRTTVGSVPSIDFSSMIVATDADGDKATGAAPGSFTVTVQNDVPVVVALPPQTGTETTSYSLTGVQAGHD
LYKVLNGANDKDILLSGRTGTTQDAVNTSNDIGVGNGQNINGKDNNSAEEYLVLDFVKNGVATGNNMSTTFTATEHYSVASLTFKVSQTTGPGTASVFLR
AFSVPTTEDNNSGSANFTDNARVAITTVSVNGTNWPVTVVYAADGVTPIGYLIQGVNAGDSIKVTGAADFGRLEISNYDGVTINTGGGGSATISGAKSFG
VLISGSESTVIQPFEVRHDESPLVNNTADPNIADDTAAPLPSSLATRIGGLALTELGHAVSSGTVAGFFTSQPGADEPAAITYQLANGTANTPFTGQDSG
LTTTDGGIAIKLFTDPSDPRILWGIAGNDFASGQKVFAAYVDDAGKLWLVQFQAIHNPVAGSTAADYDDAVSVIGNIHVQANVTDADGDVASAVSGTPIR
LTFQDDGPHAINDIDTVLGADMTATGNVLTGIDIAAGDANTDDGVADKLGTDGGKVASVAGVSTDSSPDAAHNFQVIGNYGSLVLNEDGSYVYTRFNSDP
VVSQDIFTYTIKDGDGDISTATLTIGVADNGTLITNLTPAAGGGDATLSEANLSDGSSSNPAALVQTGSFNVFAPDGIQSVQIGGTVLTIAQLGAASSAS
PIAISGNPAYGSLEIIGFDSGSGEITYRFTLTDNTLTHTNITADATPGDRGTDDPVFANYTVTVTDTDNDSSSGTLAIRVLDDGP

Rm14 repeats
TAHADADAGIEGALIGGNVLTGIGTTGGVSGADVFGADGPTVAGGGVTGVATGSNTASPVTGNVGTPIVTALGTLTLNGDGSYTYQAKPNVVPAGGATDT
TAHADADAGIEGALIGGNVLTGIGTTGGVSGADVFGADGPTVAGGGVTGVATGSNTASPVTGNVGTPIVTALGTLTLNGDGSYTYQAKPNVVPAGGATDT
TAHADADAGIEGALIGGNVLTGIGTTGGVSGADVFGADGPTVAGGGVTGVATGSNTASPVTGNVGTPIVTALGTLTLNGDGSYTYQAKPNVVPAGGATDT
TAHADADAGIEGALIGGNVLTGIGTTGGVSGADVFGADGPTVAGGGVTGVATGSNTASPVTGNVGTPIVTALGTLTLNGDGSYTYQAKPNVVPAGGATDT
TAHADADAGIEGALIGGNVLTGIGTTGGVSGADVFGADGPTVAGGGVTGVATGSNTASPVTGNVGTPIVTALGTLTLNGDGSYTYQAKPNVVPAGGATDT
TAHADADAGIEGALIGGNVLTGIGTTGGVSGADVFGADGPTVAGGGVTGVATGSNTASPVTGNVGTPIVTALGTLTLNGDGSYTYQAKPNVVPAGGATDT

FVYTITDGDGDTSTVTLTINLSDSGITAS-NDAIVNEAGLSTGSDASSNSEIFATGQITASSAVPIVSYAITSVGGGTGSYGTLSLNPTTGAYTYTLTSR
FVYTITDGDGDTSTVTLTINLSDSGITASDNDAIVNEAGLSTGSDASSNSEIFATGQITASSAVPIVSYAITSVGGGTGSYGTLSLNPTTGAYTYTLTSR
FVYTITDGDGDTSTVTLTINLSDSGITASDNDAIVNEAGLSTGSDASSNSEIFATGQITASSAVPIVSYAITSVGGGTGSYGTLSLNPTTGAYTYTLTSR
FVYTITDGDGDTSTVTLTINLSDSGITASDNDAIVNEAGLSTGSDASSNSEIFATGQITASSAVPIVSYAITSVGGGTGSYGTLSLNPTTGAYTYTLTSR
FVYTITDGDGDTSTVTLTINLSDSGITASDNDAIVNEAGLSTGSDASSNSEIFATGQITASSAVPIVSYAITSVGGGTGSYGTLSLNPTTGAYTYTLTSR
FVYTITDGDGDTSTVTLTINLSDSGITASDNDAIVNEAGLSTGSDASSNSEIFATGQITASSAVPIVSYAITSVGGGTGSYGTLSLNPTTGAYTYTLTSR

FDTSPDADNATNTEQNRDVFTYTATDANGNTVTGTIEVDIIDDVP
FDTSPDADNATNTEQNRDVFTYTATDANGNTVTGTIEVDIIDDVP
FDTSPDADNATNTEQNRDVFTYTATDANGNTVTGTIEVDIIDDVP
FDTSPDADNATNTEQNRDVFTYTATDANGNTVTGTIEVDIIDDVP
FDTSPDADNATNTEQNRDVFTYTATDANGNTVTGTIEVDIIDDVP
FDTSPDADNATNTEQNRDVFTYTATDANGNTVTGTIEVDIIDDVP

TARAGAAMNVLETDGQTNGTSLLANDTQGADGATLTHVNLGSGFVAITTGTSLGGGVYSFSVSGIGVYTFKADGTWTFDPAVNASTSNQSGSFTYRITDG
DGDTSEAAQVITIANVNNVPTGGSVTASVDDEGLLHGLVGGTGDDTTTASAFATGTLTGSGGDGALSYSFANLAGTPAVTIGQEQVTYSWDSTLNRLVAT
ISSSTIAARVGQVLFTVDVTPSTGAYQVSLINAVLHTNANNAEAGDLQLALQYKVSDSDADTSAGDTGTGTLTINFDDDSPVNFTAQAMTIENGANAIGS
GALNFYESIGADGGSAVFSGTNGSTLMSGASAVTSGGKTVHLYGFGTDTLTARIDLDGNGTDETTVFTVKLSPNAKSEPSDVYTVQFSRALDDGSGTTIT
TSNLSSTSKRDYKMVDDTSANDQDILISASHSNGTQGAVNGSSSGSSVAFGVDQPVVGTGDILRFDFAKSVTVTGSANNSYTDGAHYNTNGFSFTVDGTG
GSGVLVVAYDANDNTANSYADLTNDAASKDTITQIYKNGVLLSLASLTASGGGYIVPSVTGDIISVYTADGYNRIEVSHASGADFQISNVGYLRFDAGTP
LNMSFNVQATDADGDTSTGSITLTTTPPTTTINGTAGHDNLVAGLGVDTLNGLGGDDTLVGGAGADVLVGGAHSTLGDTASYQNSAAGVTVDLTLVGVAQ
TSTGDASGDKLTGIENLIGSNFNDLLTGDGNANVLYGLDGNDTLHGGGGDDMLYGGAGLNHLYGEGGKDTFVIDQSALSEVGMVDVIHDYSNADGDVVDL
SELLNVALGTNVGTAGYVDYDNASGALKVDVDGGGNNYVTVATLNPGLTTVNILYSEDNHDKTGTAT

Rhi-42 WP_146992040.1
MQGSFQVAQATGTGNSSTNSAPVRVYKLTKPLTDQAVVVNLGYDQKAKVDFSSIANEKITLVHIGEKLIIILFDNQSTVTVEPFFDSRADGITIEMAPGRD
VSVQEFASLFPIGTDGSVLPAADNGGNSNGNAQASGANFSPFAVDPLDPVPTNVLAGPEGLPGSNDTGPTGTTLPQGSTPAKPTISLGVAPDLAVDESFL
TVATNGIAGSGLGPAGATVATGLMPFTITAPGGEKSVAFALSISAQGVDSGLIDSQTGNHVFLFQENGQIVGREGGQNGLADFTISVDATGHITLTDLRS
MHEGTGEVGGDINEGVHLPAGMVTLTATVTDVFNQTGTATADVGPHLTFLDDGP

Rb1 repeats
AISLSGTASSLNTFEAYLSAATNAGINGSTPDASPTQGHALDTESFAHAFTIVTGADGATTAYALGIAANGTATNLIDSASGLGVVLDQSGNTISGYVAG
SISLSGTVGSLNTFEAYLSAATNAGVNGSTPDAVPTQGHALDTESFAGAFTVVTGADNATTAYTLSIAGNGTATNLIDSNSGLGVVLDQTGNTISGYVTG
SIALTGKIGSLNTFEAYLSAATNAGVDGSTPDAVPTQGHALDTESFAGAFTVVTGADNATTAYALSIAANGTATNLIDSASGLAVVLDQSGNTVSGYVTG
SITLSGNVNSLNTFEAYLSAATNAGVDGSTPDAVPTQGHALDTESFAGAFTVVTGADGATTAYTLSIAGNGTATNLIDSASGLGVVLDQTGNTVSGYVTG
SITLSGNVNSLNTFEAYLSAATNAGIDGSTPDAVPTQGHALDTESFAGAFTVVTGADNATTAYTLSIAGNGTATNLIDSASGLGVVLDQTGNTISGYVTG
SVTLSGNVNSLNTFEAYLSAATNAGINGSTPDAVPTQGHALDTESFAGAFTVVTGADNATTAYALSISANGVPTNLIDSASGLAVVLDQTGNTISGYVTG
SIGLSGRVGSLNTFEAYLSASTNAGINGSTPDAVPTQGHALDTESFAGAFTVVTGADNATTAYALSISANGVPTNLIDSASGLAVVLDQTGNTISGYVTG
SISLSGNVNSLNTYEAYLSAATNAGINGSTPDAVPTQGHALDTESFAGAFTVVTGADNATTAYALSISANGVPTNLIDSASGLAVVLDQTGNTISGYVTG
SISLSGNVNSLNTYEAYLSAATNAGINGSTPDAVPTQGHALDTESFAGAFTVVTGADNATTAYALSIAANGTPTNLIDSASGLAVVLDQTGNTISGYVTG
SISLSGNVNSLNTFEAYLSAATNAGINGSTPDAVPTQGHTLDKESFAGAFTVVTGADNATTAYALSISANGTPTNLIDSASGLAVVLDQTGNTISGYVTG
SISLSGRVGSLNTFEAYLSASTNAGVNGSTPDAVPTQGHTLDKESFAGAFTVVTGADNATTAYALSIAANGTPTNLIDSASGLAVVLDQSGNTVSGYVTG
SISLSGNVNSLNTFEAYLSASTNAGVNGSTPDAVPTQGHALDKESFAGAFTVVTGADNATTAYALSISGNGVATNLIDSASGLAVVLDQSGNTVSGYVTG
SISLSGNVNSLNTFEAYLSAATNAGINGSTPDAVPTQGHALDTESFAGAFSVVTGADGATTAYTLGISGNGVATNLIDSASGLGVVLDQTGNTVSGYVAG
SISLSGNVNSLNTFEAYLSAATNAGINGSTPDAVPTQGHALDTESFAGAFTVVTGADGAITAYTLGISGNGVATNLIDSASGLGVVLDQTGNTISGYVAG
SIKLSGTVASLNTYEAYLSAATNAGINGSTPDAAPTQGHTLDKESFAGAFTVVTGADGATTAYALSIANNGIATNLIDSASGLGVVLDQTGNTVSGYVAG

HEGQAAWLVFTLSVNTATGDVTLTQDRAVHENIASSPDTGEGISLTGGLVTLTATVTDNDGDSASQKLDLSTHVTFHDDGP
HEGDAAWLVFTLTVNTATGDVTLTQDRAVHENTASSPDTGEGIGLNEGLVTLTATVTDKDGDSAAQKLDLSSHVTFHDDGP
HEGDAAWLVFTLAVNTATGDVTLTQDRAVHELTASSPDTGEGVSLTGGLVTLTATVTDNDGDSASQKLDLSSHVTFHDDGP
HEGDAAWLVFTLAVNTATGDVTLTQDRAVHELTASSPDTGEGISLTGGLVTLTATVTDKDGDSAAQNLDLSSHVTFHDDGP
HEGDAAWLVFTLAVNTATGDVTLTQDRAVHELTASSPDTAEGISLSNGLVTLTATVTDKDGDSAAQNLDLSSHVTFHDDGP
HENDAAWLVFTLSVNTATGDVTLTQDRAVHELTASSPDTGEGISLTGGLVTLTATVTDKDGDSAAQNLDLSSHVTFHDDGP
HENDAAWLVFTLSVNTATGDVTLTQDRAVHELTASSPDTGEGISLTGGLVTLTATVTDKDGDSAAQNLDLSSHVTFHDDGP
HENDAAWLVFTLSVNTATGDVTLTQDRAVHELTASSPDTGEGISLTGGLVTLTATVTDKDGDSAAQNLDLSSHVTFHDDGP
HEGDAAWLVFTLAVNTATGDVTLTQDRAVHELTASSPDTGEGISLTGGLVTLTATVTDKDGDSAAQKLDLSSHVTFHDDGP
HENDAAWLVFTLAVNTATGDVTLTQDRAVHELTASSPDTGEGISLTGGLVTLTATVTDKDGDSAAQNLDLSSHVTFHDDGP
HDNDAAWLVFTLTVNTATGDVTLTQDRAVHELTASSPDTGEGISLTGGLVTLTATVTDKDGDSASQNLDLSTHVTFHDDGP
HEGDAAWLVFTLAVNTATGDVTLTQDRAVHELTASSPDTGEGISLTGGLVTLTATVTDKDGDSAAQNLDLSTHVTFHDDGP
HEGQAAWLVFTLTVNTATGSVTLTQDRAVHELTASSPDTGEGISLTGGLVTLTATVTDKDGDSAAQNLDLSTHVTFHDDGP
HENQAAWLVFTLSVNTATGSVTLTQDRAVHENTASSPDTGEGISLTGGLVTLTATVTDKDGDSAAQNLDLSSHITFHDDGP
HDNQAAWLVFTLAVDPATGNVTLTQDRAVHENTASSPDTGEGISLTGGLVTLTATVTDKDGDSASQNLDLSTHVTFHDDGP

SWTSAEHGVLANQAGFSVTGDLQYITGADGLASLYFTGVSLDPLHPTATTIQAGGFAVSEYVDASGVLHGVANGADVFTVTLNSATSTYSFDLLHTLDAY
TSLSVNPANLGGSGPVVYATLADTTGNPVTNLTGYHTTGLFNVNSWLNETHDTLTSLQVTQDFVNASASGEGVSSNNFNTGDFMRFDFGAVGDNSNLDHW
TYGAAPGAVSVATGNQITFTVNPIGGTAIIDYVVHSLDGTVTHGVYDSSVNGTTLTANGHGNIDYVELYELSGKSKILLSGITELVNNGQTDVPFTVGVK
DGDGDTTSGSFDVNVNGATTLTGTAGNDVIVAGASAETLTGNGGNDKFVLNLSAHFTISDFNSGDLVLLDVPGLSLPAGTSNPLPANQFTSSAATGADQN
AASAWNESASTNKFFFNNTTHELWYSANGTGSDKVDLAHLSTGVPAAANIHIY

Rhi-44 WP_095733983.1
MQGSFQVAQATGTGNSTNSAPVRIYKLTKPLTDQAVVVNLGYDQKVKVDFSSIANEKITLVHIGEKLIILFDNQSTVTVEPFFDSRTDGQHNITIEMAPG
RDVSVQEFASLFPIGTDGSVLPAADNGGNSNGNAQASGANFSPFAVDPLAPVGTNALAGNEELPGFTDNAPTGFVLNGISPAPTISLGTAPDLAVDESFL
TVATNGVAGSGLGPAGSTVATGLMPFSISVPGGEKSLTFALSVSAQGVDSGLIDSQTGNHVFLFLENGQVVGREGGQNGLADFTISVDATGHITLTDLRA
MHEGVGEAGGDINEGVHLPAGMVTLTATVTDLFNQTGTATADVGPHLTFLDDGP

Rb2 repeats
LNPTVTLSGVNPGELLTFDGGLAGGNFTGTQDVHDTNASATVATENFSGAFTFGNLNAAGADG-GTTAISYTLGLHAGVVDGSASGLTHAGSTIFVY--D
KVPTVTLSGLKTGELLTFDGGLAGGNFTGTQDVHDTNASATVATEDFSTAFTIGNTNLYGADGAGATTIDYTLGFHAGVADGVDSGLTHAGSTIYLY--D
QTPTVTLAAFTAPVLLTFDGGLAGGNFTGTQDSHDTNASATIATEDFSGAFTIGNTSLYGADGAGATTINYTLGFHAGVADGVDSGLTHAGSTIYLY--D
QTPTVTLAAFTAPVLLTFDGGLAGGNFTGTQDVHDTNASATIATEDFSGAFTIGNTNLYGADGAGATTINYTLGFHAGVADGVDSGLTHGGSTIYLY--D
QTPTVTLAAFRAPVLLTFDGGLPGGNFVGTQDVHDTNASATVATEDFSGAFTIGNTNLYGADGAGATTINYTLGFHTGVADGVDSGLTHGGSTIYLY--D
QAPTVTPGAFNAPVLLTFDGGLAGGNFTGTQDSHDTNASATIATEDFSGAFTVGNTSLYGADGPGSTTISYTLALHTGVADGSASGLTHAGSTIYLY--D
QTPTVTLAAFTAPVLLTFDGGLAGGNFTGTQDAHDTNASATVATEDFSGAFTIGNTNLYGADGAGATTISYTLGFHTGVADGVDSGLTHAGSTIYLY--D
QTPTVTLAAFRAPVLLTFDGGLAGGNFTGTQDAHDTNASATIATEDFSGAFTIGNTNLYGADGAGATTINYTLGFHTGVADGVSSGLTHAGSTIYLY--D
QTPTVTLAAFTAPVLLTFDGGLAGGNFTGAQDSHDTNASATIATEDFSGAFTIGNTNLYGADGAGATTINYTLGFHTGVADGVSSGLTHAGSTIYLY--D
QTPTVTLAAFTAPVLLTFDGGLAGGNFTGPQDSHDTNASATIATENFSGAFTIGNTNLYGADGAGATTINYTLGLHSGVANGSPSGLTHAGSTIYLYLDS
QTPTVTLAAFTAPVLLTFDGGLAGGNFTGTQDSHDTNASATIATENFSGAFTIGNTNLYGADGAGATTINYTLGLHSGVANGSPSGLTHAGSTIYLYLDS
QTPTVTLAAFTAPVLLTFDGGLAGGNFVGTQDSHDTNPSATIATENFSGAFTIGNTNLYGADGAGATTINYTLGLHSGVANGSPSGLTHAGSTIYLYLDS

VGGVITGSTSATAGGINAGNTVFTLSVGLTTGIVTLTQFESIDHSQTDTYNGSYINDIKSLAANLIDLKASAVTTDNDGDTSATISAVLDLGGNVQFGDDGP
VGGAITGSTSTTAGGINAGNTAFTLSVDLTTGIVTLTQLESIDHSQTDTYNGSYISDIKQIAANLIDLTASAFTTDSEGDKTVTASASVDVGHQIQFGDDGP
VGGVITGSTSTTAGGINAGNTAFTLSVDLTTGIVTLTQLESIDHSQTDTYNGSYLSDIKQIAANLIDLTASAFTTDSEGDKTVTASASVDVGHQIEFGDDGP
VGGVITGSTSTTAGGINAGNTAFTLSVDLTTGIVTLTQLESIDHSQTDTYSGSYINDIKQIAANLIDLTASAFTTDSEGDKTVTASASVDVGHQIEFGDDGP
VGGVITGSTSTTAGGINAGNTAFTLSVDLTTGIVTLTQLESIDHSQTDIYNGAYINDVKQLAANLIDLTASAFTTDSEGDKTVTASASVDVGHQIQFGDDGP
VGGVITGSTSTTAGGINAGNTVFTLSVGLTTGIVTLTQLESIDHSQTDTYSGSYINDVKQIAANLIDLTASGFVTDSEGDKSVTATATIDVGHQIEFGDDGP
VGGVITGSTSTTAGGINAGNTAFTLSVGLTTGIVTLTQLESIDHSQTDTYSGSYINDIKQIAANLVDLTASAFTTDSEGDKTVTASASVDVGHQIEFGDDGP
VGGVITGSTSTTAGGINAGNTAFTLSVGLTTGIVTLTQLESIDHSQTDTYSGSYINDIKQIAANLIDLTASAFTTDSEGDKTVTASASVDVGHQIEFGDDGP
VGGVITGSTSTTAGGINAGNTAFTLSVGLTTGIVTLTQFESIDHSQTDTYSGSYINDVKQLAANLIDLTASAFTTDSEGDKTVTASASVDVGHQISFGDDGP
VTGIVTGSTSATQAGINAGNTAFTLSVGLTTGIVTLTQLESIDHSQTDTYSGSYINDIKQLAANLIDLTASAFTTDHEGDKTVTASASIDVGHQISFGDDGP
VTGIVTGSTSATQAGINAGNTAFTLSVGLTTGIVTLTQLESIDHSQTDTYSGSYINDVKQLAANLIDLTASAFTTDHEGDKTVTASASIDVGHQISFGDDGP
VTGIVTGSTSATQAGINAGNTAFTLSVGLTTGIVTLTQFESIDHSQTDTYSGSYINDVKQLAANLIDLKASAFTTDSEGDKTVTASASIDVGHQISFGDDGP

Rb3 repeat
KAPTLTVSAATVGVDETPGVQTVGGASDVLGSTAITFNGAAATVAGLFATVANAGTDTDVSSAVLDNGALSFASTGAASILTASGGSYGADGAGTTVYAL
SVLNAASGLTLTDGTAITLSLDASGRIIGTVGADAANPSLTGKVAFAIAIDPATGQLYVAEYLSLHQNDTTNPNDVLTMAAGKVGATVTVTDSDGDHATS
SADIGTHISFLDDGP

TLGAFVNGTLPNDIGSVHGTFAVNFGADGFGGFTLTGPTIAGIAYSTVTLTDASGHAIGTELIGSAGATQVFDLKVFEDGTYTFDLLHPQAATTVTDSLL
GLTSGHVPWVELADGSIEFSSPGDINSSTQGFGVGNNFLNGGETFTMEFHVPGTGVGTNDLPNSNPQFVDFVNFSVNSGNVGDTVSWTATDTVHNITQSG
TASLDATGHLLIDPTISFNLLQVTGVGNESIRLTAAEIGKTVLPSDQNLSFGIVATDGDGDHTTSSTLGIHVVAGDSSGNFTLTGGATADVIATSSHTDT
VVGGGGTDIVDYRDDTTGVTVNLQTGTGTGGSAQGDTYSSIEGILGGSGNDTLTGLAAGGNYLDGGAGNDTLNGGAGNDTFVLQLNGGGHDTINNFNALS
DQIFVNLGDNLSIGQASTVAAANFHTGDETNAATWNGGTGKEFVFNAATQELWYSANGTGTDKVDLAHVSTGVPAATNVHVY

Rhi-46 WP_137476231.1
MQGSFQVAQATGTGNSTNSAPVRIYKLTKPLTDQAVVVNLGYDQKVKVDFSSIANEKITLVHVGEKLIILFDNQSTVTVEPFFDSRADGQRNVTIEMAPG
REVSVQEFANLFPISTDASVLPAADNGGNSNGNAQASGANFSPFAVDPLAPVPTNVLAPQEELPNLVTPLPPTGFVLGIAPPPIISGNPLSALIVDESFL
TAATNHGVAGSGLGPAGATVASVQVSFNLTVPGGQQSLTYALSVTTPNEDSGLIDSATGQHVLLTVNAAGVVEGRTAISGDLVLTISVDATGHVTLTDLR
AVHEATPGNFNEGITLASGLITLTATVTDNNNQSASASVDVGSHLTILDDGP

Rb4 repeats
VAGTVT---GATDTLVLDETRPVGSDTAGGTTPTGLASVTADFSNNFTGGSYGADGAGSTAYTLHLTGTNVASGLYALDPTDT---NPAHFGQGAQIVLN
TAGTVIEKEGPAETLVLDESRPVGGDTPGGFAPSGLASVTADFSHDFTSGSYGSDGPGHTAYTLKLTGSNVASGLYALDPTDTTTADGDGIGQGAQIVLN
TAGTVT---GPTDTLVLDETRPVGTDTAGGTAPTGLDTVTADFSNNFTPGSYGSDGPGHTTYTLNLVGANVASGLYALDATDTTTADGDGIGQGAQILLN
SAGTVI---AAADTLVLDETRPVGTDTAGGAAPTGLDTVTADFSNNFSAGSYGSDGAGNTTYTLKLTGANVASGLFALDATDTTAADGDGVGQGAQILLN
TAGTVA---GPTDTLVLDETRPVGSDTVGGTAPTGLDTVTADFSNNFTGGNYGSDGAGNTAYTLKLTGANVASGLYALDPTDT---DPTHFGQGAQILLN
TASTLS---GATDTLVLDETRPVGSDTAGGTAPTGLASVTADFSNGFGGGSYGSDGPGNSAYTLKLTGANVASGLYALDATDTTTGDGDGIGQGAQILLN
TAGTVI---AAADTLVLDETRPLGTDTTGGTAPTGLDTVTADFSSNFSAGSYGSDGPGNTTYTLKLTGANVASGLFALDATDTTTGDGDGIGQGAQILLN
TAGTVT---GPTDTLVLDETRPVGSDTAGGTAPTGLDTVTADFSNNFTGGNYGSDGPGNTAYTLKLTGVNVASGLYALDPTDT---DPTHFGQGTQIVLN
TAGTVT---GPTDTLVLDETRPVGSDTAGGTAPTGLDTVTADFSNNFTAGSYGSDGAGSTTYTLKLTGANVASGLYALDPTDT---DPTHFGQGTQIVLN
TAGTVT---GPTDTLVLDETRPTGTDTAGGTAPTGLDTVTADFSNNFTAGSYGSDGAGSTTYTLKLTGANVVSGLYALDPTDKTAGDGDGIGQGAQIVLN
TAGTVT---GPTDTLVLDETRPVGSDTAGGTAPNGLDTVTADFSNNFTGGSYGSDGAGSTTYTLKLAGANVASGLYALDPTDKTASDGDGIGQGAQIVLN
TAGTVT---GPTDTLVLDETRPVGTDTAGGTAPNGLVSVTANFSDNFTGGSYGSDGAGSTTYTLKLTGSNVASGLYALDPTDKTAGDGDGIGQGAQILLN

QVGDTITGSANGTTYFTITINETTGIVTFTQVNNIWHADPTNPDDAATLTLSSASLLQVVQTITDADGDSVSAPINLGGGVFTIQDSGP
QSGNTITGSAGGTTYFTITIDPSTGIVTFTQSNNIWHSDPKNPDDPATLTLSSPSLLQVVQTITDADGDSVSTPLDVGTRVFTIQDSGP
QVGNTITGSAGGTDYFTITIDPSTGIVTFTQLNNIWQSDPTNPDDAATLTLAGANLLQVVQTIVDADGDSVTTPLDVGTGVFTIQDSGP
QVGNTITGSAGGTDYFTITIDPSTGIVTFTQLNNIWHSDSTNPDDTATLTLSSATLLQVVQTITDADGDSVSTPLDLGTGVFTIQDSGP
QVGDTITGSAGGTNYFTITIDETTGVVTFTQLNNIWHSDPTNPDDAATLTLSAANLLQVVQTITDADGDHVSTPLDLGTGVFTIQDSGP
QVGNTITGSAGGTDYFTITIDPSTGIVTFTQLNNIWHADPTSPDDAATLTLTRPELLQLVQTITDADGDSVTTSLDLGTGVFTIQDSGP
QVGNTITGSVGGTNYFTITIDPSTGIVTFTQLNNIWHSDPTNPDDSATLTLSAANLLQVVQTITDADGDSVTTPLDVGTGVFTIQDSGP
QVGDTITGSANGTTYFTITINEMTGIVTFTQVNNIWHADPTNPDDSATLTLSAANLLQVVQTITDADGDSVTTPLDVGTGVFTIQDSGP
QVGDTITGSANGTTYFTITINETTGVVTFTQVNNIWHADPTNPDDAATLTLSAANLLQVVQTITDADGDSVATPINVGTGVFTIQDSGP
QVGDTITGSANGTTYFTITINETTGIVTFTQVNNIWHADPINPDDAATLTLTRPELLQVVQTITDADGDSVTTPLNLGTGVFTIQDSGP
QVGNTITGSVGVTNYFTITIDPSTGIVTFTQLNNIWHSDPANPDDAATLTLSAANLLQVVQTITDADGDSVTTPLNLGTGVFTIQDSGP
QSGNTITGSVGATTYFTITIDPATGIVTFTQVNNIWHSDPNNSDDAATLTLASANLLQVVQTIVDADGDSVSTPLDLGTGVFTIQDSGP

Rb3 repeat
TAPTATVSAAKIGVDETPGVQTIGGASDVLGSTAITFNGAASTVAALFSTVANKGVDTDVSAASLDNGALSFASTGASSILTVAGGSFGSDGAGSTTYAL
SVLSAASGLTLTDGTGIELSLDASGRIIGTVGNDVANPSLTGQTAFAIAIDPITGQLYVADYLSLHHNSTASPNDLLTMTAGKIGATVTLTDADGDHVTA
TADISTHISFLDDGP

SFGTIEHGIIADYAGMSVLGNLPVFSGADGFGAYDFTLAGNTAPAGLTFNGQAVHYSIDAFGQSLTAYIGAAPSAANNIFTLNLDDTTGHYLFTLLQPFT
ETTIQNVGSSTSFGSGPTAEQILSSSTNQLAVISGTAADGTQGGVNGSTVGWGVDNNGFNVNEKMLFDFTDGASASPVGNAGFHPPAHESVANYTFSGYK
AGDDIHYVVSYWDGVHAASITSVSGDFDPSTHATVATEWSTPTAPAGLQLYTVQFTDVTGSGKVDLVGVGVSSTTNVSEDLSFNVSTIDGDGDTASSTIN
ININGSTTLLGTAGNDVIVAGPTSETLTGNGGDDKFVLNLSAKEIISDFNSGDTLLLDVAGLSLPTGVSNALTAGQFTSSAVTGGTENSASAWNESASTN
KFFFNNTTHELWYSANGTGSDKVDLAHLSTGVPAATNVHIF

Rhi-50 OMI11765.1
MQGSFQVAQATGTGNSTNSAPVRIYKLTKPLTDQAVVVNLGYDQKVKVDFSSIANEKITLVHVGEKLIILFDNQSTVTVEPFFDSRADGLKNVTIEMAPG
REVSVQEFATLFPISTDASVLPAADNGGNSNGNAQASGANFSPFAIDPLAPVPTNVLAPQEELPNLVTPVPPTGFVLGVAPPPTISGNPLSALIVDESFL
SAATNHGVAGSGLGPAGATVASVQVSFNVTVPGGQQSLTYALSVTTPNEDSGLIDSATGQHVLLTVNAAGVVEGRTAISGDLVLTVSVDAAGHVTLTDLR
AVHEATPGNFNEGITLASGLITLTATVTDNNSQSASASVDVGSHLTILDDGP

Rb5 repeat
AITMTATAPADALVVDETNLAINASANFADNFTVTSSYGADGAGTTASAYTLGIKSAGVESGLIDVATGQHVYLYVNGNVVEGRVGAGGAADPAGVIDFT
VSVSAAGVVTLDQVHALQHPNAANPDDVVTLSATDLITLTRTDTITDRDGDTTSGSASINIGQALSFHDDGP

Rb6 repeats
SLGTPTAAVAGQLTLDETNLTGAPISTSANFAGNFDGGTPHYGTDGPGHVDYALALSANGTGSGLYALDPADVTTADNDGIGQGSQILLYQLADGSIVGK
SLGTPTAAVAGQLTLDETNLTGAPISTSANFAGNFDGGTPHYGTDGPGHVDYALALSANGTGSGLYALDPADVTTADNDGIGQGSQILLYQLADGSIVGK

VGAAADGSGGTLYFTIGVAANGDVTFTQDKNIWHSNTGNNDELASLTTAANTLLLNQTVTDADGDHVTKGIDLGSGVFAIHDDGP
VGAAADGSGGTLYFTIGVAANGDVTFTQDKNIWHSNTGNNDELASLTTAANTLLLNQTVTDADGDHVTKGIDLGSGVFAIHDDGP

Rb3 repeat
TAPTATVSAAKIGVDETPGAQTTGGASDVLGSTAITFNGAASTVAALFSTVANKGVDTDVPGASLDNGALSFASTGASSILTVAGGSFGSDGAGSTTYAL
SVLSAASGLTLTDGTGIALSLDASGRIIGTVGNDAANPSLTGQTAFAIAIDPITGQLYVADYLSLHHNSTASSNDLLTMTAGKIGATVTLTDADGDHVTA
TADISTHISFLDDGP

SFGTIEHGIIADYAGMSVLGNLPVFSGADGFGAYDFTLTGNTAPAGLTFNGQAVHYSIDAFGQNLTAYIGAAPSAANNIFTLTLDDTTGHYLFTLLQPFT
ETTIQNVGSSSAFGSGPSQEQILSSSTDQLAVISGTASGGAQGSVNGSTNGWGVGNNNFDPNEKLLFDFTDGASASPGGNAGFHPPAHESVANYTFSGYK
AGDDIHYVVSYWDGVHAASITSVSGDFDPSTHTTVATEWSTPTAPAGLQLYTVQFTDVTGSGKVDLVGVGVSSTTNVSEDLSFNVSTIDGDGDTASSTIS
ININGTATLLGTAGNDVIVAGPTNETLTGNGGDDKFVLNLSAHEIISDFNSGDTLLLDVAGLSLPTGVSNALTAGQFTSSAVTGGTENNASAWNESASTN
KFFFNNTTHELWYSANGTGSDKVDLAHVSTGLPAATNVHIF

Rhi-51 WP_063694591.1
MNEQFRIAQATGAGNSNNASPPRIFKLTKPFADQAVVLNLGYDQKVQVDFSAIANEKITLVHVGEKLIILFDNHSTVTVEPFFDSRHDQLGNLTIQVAPG
RDLSVAEFASAFPIINDPSAASAVLPAAGDSLGQNNAQASGANFSPFTVDPLPPVGTNTLAPQEDLPTFQTIPPTGFVQQPTTPPAPTISLGTLPELVLD
ESFLTSATNGIDGSTPDALSTVISGILPVTLNVPGGQQSLTFALSISASGVDTGLIDSQTGNHVFLFLESGVVVGREGKDAATAASGPQDFTLSVDSSGK
LTLTDLRSVHQGSGEDGDISEGIHVPAGLISLTATVTDNSNQSTSASVDVGPHLTLQDDGP

Rb7 repeats
SIHVVLDGEGEGQVLTNLVVDESNLTAVTNGIEGSIHNGINSAIGWFGGIFQANSGADGVADSEHPISYALSIGPSGTSGLVDAQTGEAVVLLMNGTTVE
SIQIVL--EGEERTLPSLIVDESNLQAATNGVDGSIHNGNNTASASFGSIFLAETGADGVPDPQHPISYALSIDTSKSSGLVDAQTGEDVVLTMNGTTVE

GHTAASNLLVFTLSVDSSGFVTFTVDRSVKEADGTNPDTNEGISLSDGAVTLTATITDNDGDTATAGIDIGRQVTFLDDGP
GHTASSNLLVFTLEVDSDGNVTFTDNRSVKEADGTDPDTNESISLASGVVTLTATITDNDGDSADSSIDLGSRVKFLDDGP

Rb8 repeats
TAHIALNGEATLIVDETDGVGAPGEVDPVGGNLGTNTILGASLFTNTSEFGADGAASTTPTVYSLTLLSSVSGLTDTATGNAITLFLNGGVIEGHAGTLS
TAHIALNESPTLIVDETDGVGAPGEVDPVGGNLGTNTILGATLFTNTSVFGSDGAASATPTVYSLSLLSTVSGLTDTATGNAITLFLNAGVIEGHAGTLS
TAHISLNESPTLIVDETDGVGAPGEVDPVGGNLGTNTILGATLFNNTSVFGSDGAASTTPTVYSLSLLSTVSGLTDTATGNAITLFLNGGVIEGHAGTLS
TAHISLNESPTLIVDETDGVGAPGEVDPVGGNLGTNTILGATLFTNTSVFGSDGAASTTPTVYSLTLLSTVSGLTDTATGNPITLFLNAGVIEGHAGTLS

TAHIALNGSPTLIVDETDGVGAPGEVDPVGGNLGTNTILGATLFTNTSVFGADGAASATPTVYSLSLLSTVSGLTDTATGNAITLFLNAGVIEGHAGTLS
TAHISLNESPTLIVDETDGVGAPGEVDPVGGNLGTNTILGATLFNNTSVFGSDGAASTTPTVYSLSLLSTVSGLTDTATGNAITLFLNGGVIEGHAGTLS
TAHIALNGSPTLIVDETDGVGAPGEVDPVGGNLGTNTILGATLFNNTSVFGSDGAASTTPTVYSLSLLSTVSGLTDTATGNAITLFLNGSVIEGHAGTLS
TAHIALNGSPTLIVDETDGVGAPGEVDPVGGTLGTNTILGATLFNNTSVFGSDGAASATPTVYSLSLLSTVSGLTDTATGNAITLFLNAGVIEGHAGTLS
TAHISLNESPTLIVDETDGVGAPGEVDPVGGNLGTNTILGATLFNNTSVFGSDGAASTTPTVYSLSLLSTVSGLTDTATGNAITLFLNAGVIEGHAGTLS
TAHIALNGSPTLIVDETDGVGAPGEVDAVGGNLGANTILGATLFTNTSVFGSDGAASTTPTVYSLSLLSTVSGLTDTATGNAIKLFLNGGVIEGHAGTLS
TAHIALNGSPTLIVDETDGVGAPGEVDPVGGNLGTNTILGATLFNNTSVFGSDGAASATPTVYSLSLLSTVSGLTDTATGNAITLILNGGVIEGHAGTLS
TAHIALNGSPTLVVDETDGVGAPGEVDPVGGNLGTNTILGATLFTNTSVFGSDGAASTTPTVYSLSLLSTVSGLTDTATGNAITLFLNGSVIEGHAGTLS
TAHIALNGSPTLIVDETDGVGAPGEVDPVGGNLGANTILGATLFTNTSVFGSDGAASTTPTVYSLSLLSTVSGLTDTATGNAIKLFLNGGVIEGHAGTLS
TAHIALNGSPTLIVDETDGVGAPGEVDPVGGNLGTNTILGATLFTNTSVFGSDGAASATPSVYSLSLLSTVSGLTDTATNKAITLFLNGGVIEGHAGTLS

TDPLVFTLSIDASTGDTTMTQFRAVVHGTETDPDTSEAATLADNAVGVTVTVTDNDGDSSHDTANLNGVVKFLDDGP
TDPLVFTLSIDSSTGDTTLTQFRAVVHGTETDPDTSEAATLADNAVGVTVTVTDKDGDSSHDTANLNGVVKFLDDGP
TDPLVFTLSINSSTGDTTLTQFRAVVHGTETDPDTSEAATLADNAVGVTVTVTDKDGDSSHDTANLNGVVKFLDDGP
TDPLVFTLSINSSTGDTTLTQFRAVVHGTETDPDTSEAATLADNAVGVTVTVTDKDGDSSHDTANLNGVVKFLDDGP
TDPLVFTLSINASTGDTTLTQFRAVVHGTETDPDTSEAATLADNAVGVTVTVTDKDGDSSHDTANLNGVVKFLDDGP
TDPLVFTLSINSSTGDTALTQFRAVVHGTETDPDTSEAATLADNAVGVTVTVTDKDGDSSHDTANLNGVVKFLDDGP
TDPLVFTLSINASTGDTTLTQFRAVVHGTETDPDTSEAATLADNAVGVTVTVTDKDGDSSHDTANLNGVVKFLDDGP
TDPLVFTLSINSSTGDTTLTQFRAVVHGTSSDPDSSEAATLADNAVGVTVTVTDKDGDSSHDTANLNGVVKFLDDGP
TDPLVFTLSINASTGDTTLTQFRAVVHGTETDPDTSEAATLADNAVGVTVTVTDKDGDSSHDTANLNGVVKFLDDGP
TDPLVFTLSINASTGDTTLTQFRAVVHGTSSDPDSSEAATLADNAVGVTVTVTDKDGDSSHDTANLNGVVKFLDDGP
TDPLVFTLSINASTGDTTLTQFRAVVHGTSSDPDSSEAVTLADNAVGVTVTVTDKDGDSSHDTANLNGVVKFLDDGP
TDPLVFTLSINSSTGDTTLTQFRAVVHGTSSDPDSSEAATLADNVVGVTVTVTDKDGDSSHDTANLNGVVKFLDDGP
TDPLVFTLSINSSTGDTTLTQFRAVVHGTSSDPDSSEAATLADNVVGVTVTVTDKDGDSSHDTANLNGVVKFLDDGP
TDPLVFTLSINSSTGDTTLTQFRAVVHGTSSNPDTSEAVTLADNAVGVTVTVTDKDGDSSHDTANLNGVVKFLDDGP

Rb5 repeat
TIAVSTTAPADALVVDETNLAINATANFADNFTVVSSYGSDGPGTIASTYTLAVKSASVDSGLIDVATGQHILLVMNGNTVEGHVGATPTLAFTVTVDAA
GVVTLDQMRALKHPNASDPDDTVTLTAADLITLTRADTITDKDGDSANSSSSINIGQALTFHDDGP

STISPDYAILNNSIGASFTGYLDVDHNIDNNVGADQLGTISFANITNGDLAIGVVNGSNATLTSGGQNIHLYLVDNDSNPTTPLQLQGWVGSAPGGGGTE
VFAVTLNPDGSLATSNDQYTMQLFAQIGATETRTINDFSALGQNTQQFKALDVANTTQDLLFSGYVQQSNGSSNASGGSSVSASTTGIGVANNSMNDGDN
LRIDFVNNATVTNGNNNEYNYTTHYNINDFQFGIVQVNGSPPADSIEVWVRIYNANDDTSGNNTATDSAHLADDSQLNTITGIQVNGVNVNLASLTTDGN
GGYLVTGLDLHDTVLVHSSGTGYDRIEIENALQTPTFNNPALNGESFDIGTFSYVTTATTIPNVSMSFDVALTDADGDSSTSSLGMTLVSPGTLFNDYSA
SGTGVTATVGDAADPQANIKGSNFNDTLTGNSAANVLSGGPGDDTLRGMGGDDVLIGGAGNDKFVLEATAALNGHDTIADLQSTDGIFVDVASQALTIGT
AASISAAQFVSSSAGVPNEASAGAWNGSANSFFFNNTTHELWYSANGTGTDKVDLAHVSTGIPTAAAVHTF

Mus-1 WP_092826367.1
MALDIQTQDIIIDESTGLQDDDVNPSLPPFDTNTTVQYLLGLDDSGGLTSPEVAFQSDFVQVTASAGETITSVVLSQNLNGTPFSTTVGVNSGIQTVDGN
FVWLFQDTTHANVVIGVIGTSDAATMPAPTDPLAYSFALINTSATHADLYTVQYVPLLHPDATNPDDRIDLANKVFASAAGTTTVSFSGQNAAPGNHDFY
VINSTTDASKQVLVTGFLGANNATANVSTQGFGINNQSINPTETLQVDFVTGGTLPAGSGSQIQYGSHLDNVMQAGFTINQITPSNPNLRVDITIRALDV
TGNEQGSDFFNGSPTTAAPITSLKLTGQSGLASPIIADGTYDVAGNVDITVSGMGTGVVTITGLDNITTVDVTTSSQMDRLTVTGVDSNEGLDITEVHFT
SQTPNALTEEVGSFINFDDDGP

M1 repeats
SISTTGTEPTLIVDETVLATDASASFAANFSSAFGADGAGTLTYALSVVAGPSGLVDTATNQAVNLSLNGSVVEGRTAVSNDLVFTVSVDASGNVTLDQQ
TITITGAEPTLTVDETVLGTDATQSFVANFSSAFGADGAGTITYALSVVAGASGLVDTLTNEVVNLSVNGTVVEGRTAVTNALVFTVSVDASGNVTLDQQ

RAVVHADPDNPDDAATLASDDLVKLTATITDKDGDHQSASLNIGQNLVFEDDGP
RAVVHDPNNGPNDTETLTSDDLIKLTATITDKDGDHQSATLNIGQNLVFTDDAP

TITAPFDADPSLAGVQSPEQLGNAVGQTASGIFGYDIGSDAHLAAFYAGGGSDFVDTNGALAGVQINLTGTVDNAQNPNITNAVATLASESLASASFDFS
FHYDKDPITAGVQDATAGGTLVFDKAADTYTFTLNDVIDGFSFNVLHTNELIAKAPAGNTGHPEIVAEQLTPNSDPNPFFVQFTANSTTNSIGFGFNSTG
DGAPNGPPTDTAFTQGAHDMVTNVNEDWVSATQATNGVAGDTIQKGEVLTLRFFSENILGDVAPNAPGGGTERLDPTTSASGVVIKFDGVGNSEDLVLIL
DLKDANGNEVTRAVNVQNSDLIKGNANVPFPYNTEFTLDNNDALLILEQNDYTVAGETFQIQGVQIMQSANGLTGDAINLNGATGANGGSSATSNLTAWD
PTDNDVLKIVDIGFVQQTSGTIDANLDFSFALVDADGDTTATQHLLVNVSNGFIV

Mus-2 WP_012332371.1
MALDLVAQDIIIDETTGLQDNDVNPSLPPHSTNTTLSYLLGLDGPGGLASPEVAFKANFVQATASAGETITSVTLSQNLAGNAFSTTVGVNSGIRTVDGN
YVWLFQDATHANVVIGVIGTSDPAAAPAATGPLAFSFALLNTSNTNADLYTVQYVPLLHPDATNPDDRIDLTDKVFASVAGTSVANFSGQNAPPGINQFY
LLDSSNDASKQILVTGFLGTANATPNVSTQGFGVDNQSINPTETLQVDFVTGGTLHAGSASQIQYSSHLETITQAGFTINQITPSNPNLRVDIKISAFNV
QGNEQGLNFYDGSPTTTAPITSLKLTGQSGFASPITANGTYATGSGNVTVSGLGTNVVTITGLDNITTVDVTTGSPMDRLTVTGVDANEGCDITEFHFTT
QTSNAYTEQVGSFINFDDDGP

M1 repeats
SISTTGTEPTLTVDETVLATDDTKSFAANFSPAFGADGAGAVTYALSAIAGPSGLVDTATNQAVNLSLNGSVVEGRTAVGNDLVFTVSVDASGNVTLDQK
SISTTGMEPTLTVDETVFTTDDTKSFAANFSPAFGADGAGAVTYALSVVAGPSGLVDTATNQAVNLSLNGSVVEGRTAVGNDLVFTVSVDASGNVTLDQK
SISTTGTEPTLTVDETVFTTDDTKSFAANFSPAFGADGAGAVTYALSVVAGPSGLVDTATNQAVNLSLNGSVVEGRTAVGNDLVFTVSVDASGNVTLDQR

RAVVHANANDANDAATLAADNLVQLTATVTDKDGDHQAATLNIGQNLSFLDDGP
RAVVHADPNDANDAATLAADNLVTLTATVTDKDGDHQAATLNIGQNLSFLDDGP
RAVVHANANDPNDAATLAADNLVQLTATVIDKDGDHQAATLNIGQNLSFLDDGP

TITAQFDADPATPGIQTPEHLGNAVGQTATGTFGYDIGHDSHPATFYDATHSDFVDTNPAVPGIQIGLTGTVDNTQNPNITQAVATLTSESATSATFNFS
FHYDKDPLTAGVQDSTAGGTLVFDKVAGTYTFTLTDVIDGFSFDVLHTSELVSKQPTGNTGHPLIVAEQLTPNGDPDPFFVQFTGNSVTQQIGFGFNSTG
DGAPSGPPTDVSFTQGAHELVTNVNEDWVSATQSTNGVAGDTIQKGELLTMRFFKENILSDVDPSAPGGGTERLDPTTSASGVVIKFDGIGSSEDLILIL
DLKNAAGNEITRAVNVQNSDLIKGNANIPAPYNTEFTLDNNDALLIIEQNDYTVAGETYQIQGIQIMQSANGLTGNAIDLNGATGANGGSSATSNLTAWD
PTDNDVLKITDIGFVQQTSGTINANLDFSLALVDADGDATATQHINVNISNAFIV

Mus-3 WP_175607160.1
MALDIVAQDLIIDETSGLQDDDTSATNATVTYLLSLDSAGGLASPQVASQADFVVASASAGETISSIILTQNSGGTPFSTSVGVNSGIKTVDGNYVWLFQ
DATHSNVVIGVIGTSDPAAAPAATGPLAFSFALVSTSATHADLYTVQYVPLLHPDTNDPDDRIDLTDKVFASVSASTVVNFSQLGDAPPGHNDWYILDAD
VASTQKILVTAHDTGVQSEVNVSTQGLGVASQDVRFGRELQIDLISGGTQSAGKDFTNSPTAPNYTAHLDNISSAGFSISQSTPTNTKANIEIHAYNNDD
NAKGAAFPGDDNDTEINITGATFKLNGVATTAAALGITVDLSGTGMILLNVGEGVTVDFTTAGGASGSFDRFTIKNIDPDKDYFDVKEVHFTSDTPSAYT
EQVGSFINFDDDGP

M1 repeats
ISTTGTEPTLTVDESALATNDSKSFAANFNSAFGADGAGTLTYALSVVAGPSGLVDTATNQAVNLSLNGTVVEGRTAVSNDLVFTVSVDASGNVTLDQIR
ISTTGTEPTLTVDESALATNDSKSFAANFSSGFGADGAGTLTYALSVVAGPSGLVDTATNQAVNLSLNGTVVEGRTAVSNDLVFTVSVDASGNVTLDQIR
ISTTGTEPTLTVDESALATNDSKSFAANFNSGFGADGAGTLTYALSVVAGASGLVDTATNQVVNLSLNGTVVEGRTAVSNDLVFTVSVDASGNVTLDQIR
ISTTGTEATATADETDLSTNPTANFASNFSSGFGADGAGTLTYALSVVAGASGLIDTLTGEAVNLSVNGTVVEGRTAVTNALVFTVSVDATGVVTLDQQR

AVVHPDTGNDDDSKTLAADNLVKLTATVT KDGDHQAATLNIGQNLNFKDDGP
AVVHPDTGNDDDSKTLAADNLVKLTATIT KDGDHHAATLNIGQNLIFKDDGP
AVVHPDTGNDDDSKTLAADDLVKLTATVT KDGDHHAATLNIGQNLNFKDDGP
AVVHDPNNGPNDTETLTSDNLVKLTATIT KDGDHHSATLNIGQNLIFTDDAP

TITAPFDADPGTAGIQTPETLGNSAGATASGTFGYDIGHDSRPTSFYDATHSDFVDSNSTLAGVQIDLTGTVDNSQNPNITNAVATLTAESATSATFGFT
FHYDKDPITAGVQDSTAGGTLVFDKVADTYTITLTDPIDGFSFDVLHTADLVAKQPTGNTGHPLIVAEQLTPNADPNPFFVQFTANSVTQQIGFGFNATG
DGAPVGDTTFNNAGAIHDMVTNVNEDWVSATQATNGVAGDTIQKGEVLTLRFFKENILSDVDPNAPGGGTERLDPTTSASGVVIKFDGIGNSEDLVVILD
LKNANGNEITRAVNVENADLIKGNANIPFPYNTEFTLDNNDALLIIEQNDYTVAGETYQIQGIQIMQSSNGLTGTAINLNGAIGANGFSNATSNLTAWDP
ADQDVLKIVDIGFVQQTSGTIDANLDFAFKVADADGDATLLQHIPVGVLNA

Mus-11 WP_112948475.1
MALDILAQDIIVDESTDLQDDDINPSGNTNPTLLYLLSLDDPGGLTSPEVAHQANFVQASASAGETISGIVLAQDASGTPFSKTVGVNSGIQTVDGNYVW
LFQDPTNPNVVIGVIGTSDPLAEPDETGPLAFAFGLDPTSATKADLYLVQYVPLLHPDETNPDDRIDLTDHVFASVSGTTVLNFSGQNAAPGNHDFYAIN
SSNDASKQLLVTGFVGAANATANVSTQGFGVNNQSINPTEVLQVDFVTGSTLAAGSGSQIQYGSHLDNVTQAGFTINQITPSAETRRVDITIKAFDNTGN
EQGSDFFNGTTTTAVDITSIKLTGTSGFATTITADGTYATGSGNVTISGLTGTGNAVTITGLDNITTVDIATATQMDRLTVTGVDATEGCDITEFHFTSQ
STNAHTEEVGSFINFDDSGP

M2 repeats
TLEITAAPVVGAAEVIEASGVGGQDEVTITPPTFNSSAVDGKTTNVTYALALAGGAATGLTTTDGNHAITLVVDSANQISGKYDSDGNATLDATAFTVTL
TLLITAAPVVGAAVVGEASGASGQNQVTITPPTFNSSAVDGKTTNVSYALSLAGAPATGLLTTQGNFAITLVAVSATLISGQYDSDGDTVLDSTAFTVTL

SGTTVTLTSLVALEHSNAPQGAGEDNSLDLNGLINVVATVTVTDGDEDVVSQQSTSAGLSLTFNDTDP
SGTTVTLNSLVALEHDNS-QGAGEDNTLDLNGLINVVATVTVTDGDEDVVSQQSTSSGLSLTFNDTDP

TITAAFDADPGTPGIQTPEHLANTAGATASGTFGYDMTDRHTAAEYAAGISDFVDSNGALAGVQIGLTGTVDNAQNPNITQAVATLTAETETSATFNFSF
HYDKDPITAGVQDATAGGTLVFDKVADTYTFTLTDVIDGFSFDVLHTSELVAKAPTGNTGHPLIVAEQLTPNGDPDPFFVQFTANSTTQRIGFGFNSTGD
GAPSGTPADTAFTQGAHEMVTNVNEDWVSATQATNGVAGDTIQKGELLSLRFFAENILPDVNPGASGGGTERLDPTTKAGGIVIKFDGIGNGEDLVVILD
LKDANGNEITRAVNVQNSDLIKGNANVPAPYNTEFTLDNNDALLIIEQNDYTVAGETYQIQGVQIMQSANGLTGDAINLIGATGTNGGSNATSNLTAFDT
TDNDVLKIVDIGFVQSTSGTIPADLDFAFQVADADADQTATQHILVDVS

Mus-14 WP_065377507.1
MALDIQTQDIIIDETTLLQDDDVNPSLAPHVTNPTLLYLLTLDDPGGLTSPEVAHQADFVQASASAGETISGIVLAQDASGTPFSTTVGVNSDLRTVDGN
YIWLFQDPNNANVVIGVIGTSDPLAEPDETGPLAFAFGLDPTSATKADLYLVQYVPLLHPDATNADDRIDLTDHVFASVTGTSVLSFLGQNAASGNHDFY
VINSSTDASKQLLVTGFVAAANATANVSTQGFGIGNQSINPTETLQCDFVTGGNLPAGSGSQIQYGSHLDNVTQAGFTINQITPSAETRRVDITIRAFDN
TGNEQGSDFFNGTTTTAVDITSIKLTGTSGFATTITADGTYATASGDVTISGLTGTGNAVTITGLDNVTTVDFTTATQMDRLTVTGVDATEGCDITEFHF
TSQSSNAHTEEVGSFINFDDSGP

M2 repeats
TLEITAAPVVGAAVVVEASGAGGQDEVTITPPTFDASAVDGQTTNVTYALALAGGVATGLQTTDGNFAITLVADSDNLISGQYDSDGDDVLDSTAFTVTL
TLEITAAPVVGAAVVVEASGAGGQDEVTITPPTFDASAVDGQTTNVTYALALAGGVATGLQTTDGNFAITLVADSDNLISGQYDSDGDDVLDSTAFTVTL
TLEITAAPVVGAAVVVEASGAGGQDEVTITPPTFDASAVDGKTTNVSYALSLAGAPATGLLTTQGNFAITLVAVSATLISGQYDSDGDDVLDATAFEVTL

SGTTVTLTSLVALEHDNTQDATEDNTLDLDGLINVVATVTVTDGDEDVVSEESTSSGLSLTFNDTDP
SGTTVTLTSLVALEHDNTQDATEDNTLDLDGLINVVATVTVTDGDEDVVSEESTSSGLSLTFNDTDP
SGTTVTLSSMVALEHDNSQGATEDNTLDLNGLINVVATVTVTDGDEDVVSEESTSLGLSLTFNDTDP

TITAPFDADPVAPGIQTPEHLANTLNATASGTFGYDMTDKHTAAEYAAGASDFVDTDAVTLGIQIGLTGTVDNAQNPAITQAVATRTAETETSATFNFSF
HYDKDPITAGVQDATAGGTLVFNKEDDTYTFTLSDVIEGFSFDVLHTSELVAKAPTGNTGHPLIVAEQLTQDDDPNPFFVQFTANTTTRQIGFGFNSTGD
GAPAGPPTDTSFTQGAHDMVTNVNEDWVSATQATNGVAGDTIQKGELLTLRFFQEDILPDVNPGAPGGGTENVDPTAKASGIVIKFDGIGNSEDLVLILD
LKDANGNSITRAVNVQNSDLIKGNANVPPPYNTEFTLDNNDALLIIEQNDYTVAGETYQIQGIQIMQSANGLTGNGINLNGATGAANGSNATSNLTAFDT
TDNDVLKIVDIGFVQNTSGTISADLDFAFQVADADGDQTDTQHILVDVA

Mus-15 WP_183245945.1
MALDITTQELVIDETTGLQDDDSSVSNSTVTYLLSLDSAGGLTSPEVAVQADFVVASASAGETITSVVLTQDSNGTPFSTTVGVNSGIKTVDGNYVWLFQ
DATHANVVIGVIGTSDANAAPDPTGPLAFSFALVSTSATHADLYTVQYVPLFHPDDTNPDDRIDLTDKVFASVEGTSTVSFLGSNAAPGNRDFYLIDSSS
DASKQVLVIGLNGLTANVSEQGFGVKNQSINPGETLQVDFVTGGTQPAGTDASEIQYGSHIETITQAGFTINQITPSQPDKRVDITISAFNNTGNDQGSN
FYDGTATTAVDITSIKLTGASGFASIITADGTYATGSGNVTITGLSGTGNAVTITGLDNVTTVDITTASPMDRLQVAGVDANEGCDITEFHYSVTSTNAH
TEEVGSFINFDDDGP

M2 repeats
TLEITAAPVVGAAEVVEASGAGGQSQATITPPTFTASAVDGYTTDVSYELALAGGTATGLQTTVGNHAITLVVDSATQISGKYDSDGNSSLDATAFTVTL
SLSVTAVPVVGSATVTEASGAAGQSQATITAPGFTASAVDGYTSDVSYVLSLVNA-ATGLQTTEGNHAITLVADSATQISGKYDSDNDTTLDATAFTVTL

SGTTVTLTSLVALEHSNSPQGVGEDNTLDLNGLINVVATVTVTDGDNDSTSSQSTSAGLSLTFDDTDP
SGTTVTLTSLVALEHSNT-QGPGEDNTLDLNGLINVVATVTVTDGDNDSTSSQSTSAGLSLTFNDTDP

IITAPFDADPAAPGIQTPEQISNTAGATASGTFGYDMTDKHTAAEYLAGVSDFVDADTILGGVQITLTGTVANAQNPNITNAVVTLASESLTSASFNFSF
HYDADPITAGVQDATAGGTLVFDKVADTYTFTLNDVIDGFSFDVLHTAELLSKEPTSNTGHPEIVVERLAADDPTTAVDEDFYVQFTANSITQKIGFGFN
STGDGAPVGDTTFNNGGATHDMVTNTHEDWVSATQATNGVAGDTIQQGELLTLRFFNSNVGIANEATDPTAKAAGVAIKFDGIGSSEDLILILDLKDANG
NEITRAITVDNADIFKTGQVPFPYNTEFSLDNNDGLVVIEANDYNLSGETYQIQGIQIMQSANGLTGSGINLNGAVGANGGSVQNGAFTLQSFTDASFGN
EQDVLKIVDIGFVQNTSGTIDANLDFSFQIVDGDGDTTPIQDLLLHV

Mus-18 WP_087575419.1
MALDILAQDIIVDETTGLQDDDVNSSLSPHNNATVQYLLSLDSAGGLSSPQVAFQADFVQATASAGETITTVVLAQSVSGTPFSTTVGVNSGIQTVDGNY
VWLFQDATHANVVIGVIGTSDAAAMPAATGPLAYSFALMSTSATTADLYTVQYVPLLHPVATDPDDRIDLTDHVFAAVSGTTTVSFSGQNAAPGNHEFYV
INSPSDAAKQILVTGFLGAANAQANVSTQGFGVNNQSINPTEKLQVDFVTGGTLAAGSASQIQYGSHIETITKAGFTVNQITPSNPNLRVDITISAFNVQ
GNEQGLNFWDGSPTTAAPITSIKLTGQSGFASPITADGTYDVAGNVDIVVSGLGTNAVTIRGLDNITTVDITTSSPLDRLTITGVDSNEGLDVTEFHFSS
QTPNAYVEQVGSFINFDDDGP

M2 repeats
TLSITAAPVVGAAEVVEASGAGGQSQATITPPTFTAGAADGFTTNVTYALALAGASATGLLTTAGNHPITLVVDSANQISGKYDSDGNTTLDATAFTVTL
TLSITAPPVVGAAEVVEASGAGGQSQATITPPSYTAGAVDGQTTNVTYALALAGASATGLLTTTGNHPITLVVDSANQISGQYDSDGNATLDATAFTVTL
TLSITAPPIVGAAEVVEASGAGGQSQVTITPPSYTAGAVDGQTSNVTYALALAGAAATGLLTTAGNHPITLVVDSANQISGQYDSDGDTVLDATAFTVTL

SGTTVTLRSLVALEHSNAPQGVGEDNTLDLNGLINVVATVTVTDGDNDVVSSQS-TSAGLSLTFDDTDP
SGTTVTLRSLVALEHSNAPQGVGEDNTLDLNGLINVVATVTVTDGDNDVVSSQS-TSAGLSLTFDDTDP
SGTTVTLTALVALEHSNAPQGVGEDNTLDLNGLINVVATVTVTDGDNDVVSSQSVTTGGLSLTFSDTDP

TLTVPFDADPVAPGNQTPEELGNAVGATASGAFGYDMTDRRTAAEYAAGGSDFVDTNASLNGVQIDLTGTVDNAQNPGITNAVATLTAETTTSASFDFSF
HYDKDPITSGVQDATAGGTLVFDKAADTYTITLNDVIDGFSFNVLHTSELVAKQPTGNTGHPLIVAEQLTPNADPNPFFVQFTANSTTQQIGFGFNATGD
GAPNGPPTDTAFTQGAHDLVTNVNEDWVSATQSTNGVAGDTIQKGELLTLRFFQENILTDVNPGAPGGGNERLDPTAKASGVVVKFDGIGNSEDLIVILD
LKDANGNEITRAVNVQNADLIKGNANIPLPYNSEFTLDNNDALLIIEQNDYTAAGETYQIQGIQIMQSANGLTGNAINLNGAVGTNGGSNATGSLTAWDP
TDNDVLKIVDIGFVQQTSGTIDANLDFAFNIADADLDLLGVQHINVQISEMFI

Nit-1 WP_084226822.1
MQNLTATPAPAGDADDNDILVSSLPAAFSTRLTALGAGTPTVGAALSGYTGAVGNTGSNIVSITPAPGGTITDLGLVGPGGAPLNGLDSGLDTLDGVNIL
LFTDTNNNIVLGRAGAADGPIVFALYLEEIGTPVSGAKMWSVQYAPLKNTDPTNPDDSLNLLDKVFVGASQDLTFSLANAPSGQNLFLMFTTANPTTVND
GGVIRITDPAIIATGKDPANQSTGVNINTGDTINSSQGGGTTTFGTNSQMITEQEGIRFSFVTGARQNVTIPNLDQNEADVEANIDFTNVFNARAATFDI
VQLQSGKSAVVKVSAFSTAAEPGVNFIDGYGNDSSVAITHVKVTLNGTVLIDTGVNATANGVTVAFNAGVATISGVKAGEKIEYTTTADHNRVLIENGAA
LNATGDTHADFDIGGFKLLQVSTATAEVGSHVKFEDDGP

N1 repeat
SINTTGVEPVLTVDETVLATNDTKNFAANFTSAFGADGAGTLAFALGISASGADSGLVDTASGNHVFLFLNAGVVQGRAGTDAVTAATGPIVFTVSAAAN
GNVTLDQLRAVVHPDTTNPDDAKTLSADNLVTLTGTITDKDGDHASATLNIGANLVFEDDGP

N2 repeat
TVSANNVVKLDDDALSGGNPGGTGDDIDAENVSGTLGHSFGADGGSIAYLTTGAPSGFSYVADGDNLLVKQGTTTVLTLTLNTATGAYIVTQNAPIMHAA
GLDENNQAFAINYRVTDGDGDTADGTLAINVDDDTP

TLDFGNLVGTGTIAPQYGEWNGSVGADQPGNLDISLNQFQLVRPDGTTIDGSSFTFDELAGSPNASGDFLFAGSLTADFDNIAGTADTTVDFTLTALHDG
RYVFDLEQGFGSTITFSSADGSLDAGGPDPVRTLTIPPDEQVVFFGVQATTSDSDIVSAIGLGEPDLTEAQIEAGGFSFLGSANMNVSTSGIGIGNNNLD
GNSTAGINAGDESFVINPETLLTGMKVFIDNSVQGYDPTTEELYYKIFFDDGTNSGNVKVLSGDLTAEAGGQTSFAIDTQGSKLIDSVQLTMGKGVIKIP
VIEFIQATENLASDIKLDLTASLTDADSDTATSNFSVDLFANDLSGTFDFTLLGASSDLDGFNVDLAASEDAYRIEGFDTSADTLFLLGDLGAGVSINNT
GADSIVSVTETGGAQTTVITVVGVDLALTDIALV

Nit-4 SEF55012.1
MNEIITTTGVTITLDESAGLQNLTATPAPGGDANDNDIANSALPSVFSARLTALGLGSPTIGTALSGYTGAAGNTGSNIISVTPEPGGTITDLGFVGPGG
APLNGLDSDLNTLTGTNILLYTDTNNNIVLGRAGAADGPIVFALYLEETGTPVSGAKIWSVQYAPLENIDPTNPDDSLNLLDKVFVAASQDLEFSLANAP
SGQNLFLMFTTANPTTVDDNGVTRITDPAIIATGKDPANQSTGANITTGDTVNSSQGGGTTTFGTNSQMIVEGDGIHFSFVTGARQNVTIPNLDQNEADV
EANIDFTDVFDARAATFDVVQLQSGKSAVVKVSALSTDSEPGVDFIDGYGDDNSVPITHVKVALQNGTVLIDTSTGGTANGVTVAFNTGVATISGIKAGY
NIEYTTTADHNRVLVENAGSGRGNASADFDIGGFQLLQVSATTVEVGSKVNFEDDGP

N1 repeat
SINTTGTEPTLTVDETVLATNDTKNFAANFTSAFGTDGAGTLTFALGISASGVDSELVDTATNNPVFLFLESGVVVGREGTDAANAVGGDVVFTVGVVPS
GVDAGDVTLDQQRAVVHPDATNPDDAKSLLVDTLVTLTGTIIDEDGDSQSATLNIGQNLVFEDDGP

N2 repeats
TVTANSLVKLDDDALAGNPGGTGDDIDAENVSGTLAHSFGADGAGSIAYLTTGAPAGFSYVADGDNLLVQQGGVTVLTLTLNTATGAYLVTQNAPIVHAA
TVAANSLVKLDDDALAGNPGGTGDDIDAENVSGTLAHSFGADGAGSIAYLTTGAPAGFSYVADGDNLLVKQGTTTVLTMTLNTATGAYLVTQNAPIAHAA

GLDENNQAFTINYRVTDGDGDTADGTLALDVDDDTP
GLDENNQAFTINYRVADGDGDTADGTLAIDVDDDTP

TLDFGNLVGTGTIAPQYGEWNGSVGADQPGNLDISLSQFQLVRPDGSTIAGSSFTFNELAGSPNPAGDFLFAGSLTADFDNNAGTADTTVDFTLTALQNG
RYVFDLIQGFGSTITFSSANGSLDAGGPDAVRTLTIPPDEQVVFFGLQATTSDSDIVSAIGLGEPDLTEAQIEAGGFSFLGSANMNVSTSGIGIGNNNLD
GNGTAGINAGDESFVINPETLLTGVKVFIDNSVGGYDPATEELYYKIFFDDGTNSGSIKVLSGDLTSEAGGQKSFAIDTQGAKLIDSVQLTMGKGVIKVP
VIEFIKSTENLASDVKLDFSASLTDADSDTATSNFSVDLFANDLGQTFDFTLVGASSDLDGFDVDLSASQVSYKIEGFDKPADKLFLLGDSGAGVSIDNS
GANSIVSVTETTGGQTTVITVVGVDLASSDIVLI

Nit-6 WP_090575218.1
MALNIATTGETITLDETAGLQNATATPASAGDANDNDIANTALPTAFSARLTALGLGAPTIGTALSGYTGAAGNTGSNIISVTPAPGGDVTDLGLVGPGG
APLNGLDSGLDTLSGANILLYTDTNNNIVLGREEGTNAIVFALYLEETGTPVTGAKMWTVQYAPLSNTDPANHDDSLNLLDKVFVAASQDLEFSLANAPS
GQNLFLMFTTANPTVVNGRITDPAIIVTGRDPLNQSQGGNITSGDTINSSQGGGTTTFGTNNQMIVEGEGLRFSFVSGAQQDVTIPNLDQNEADVEANID
FTSMFNARAATFDVVQLQSGKSAVVKVTALSTAAEPGVNFIDGYGNDSTIPITHVKVSQGGTVLIDTSTGGTANGVTVAFNAGVATISGVKAGYNIEYTT
TGDHSRVLVENAGSGRGNASADFDVGGFQLLQVSTATVEVGSKVSFEDDGP

N3 repeats
TIAANTDPTPTLTTDDTEIVDTAGPVSFAGLFTSAFGNDGFKDADDDVQDTDAITYTLGVSGANAVSGLTDTLTGDGVVLNKVGNDIVGTAGAAGPEVL
TIAENAEPTPTLTTDDTEIVDTAGPVSFAGLFTSAFGNDDFKDADDDVQDTDAITYTLGVSGANAVSGLTDTLTGDGVVLNKVGNDIVGTAGAAGPEVL
TIAANTEPTPTLTTDDTEIPDTAGPTSFAGLFTSAFGTDSFKDSDDNDIQDADAITYALGVSGANAVSGLTDTLTGDGVVLNKVGNDIVGTAGAAGPEVL

KIMVDATSGEVTLIQSRAVVHDDPLDPSESTSPATLLTADLVTLAATITDGDGDTATATRSIGDAFKFEDDGP
RITVDA-SGNVALSQSRAVVHDDPLDPSESSSPATLLTADLVTLAATITDGDGDTATATRNIGDAFKFEDDGP
RITVDA-SGNVALSQSRAVVHDDPLDPSESSSPATLLTADLVTLAATITDGDGDTATATRNIGDAFKFEDDGP

TLDFGNLVGTGTIAPQYGEWSRLVGADQPGNLDISLNQFQLVRPNGTTINGSSFTFNELAGSPDASGNFLFAGSLTADFDNIVSTPDTTVDFTLTALNDG
RYVFDLVQGFGSTVTFSSEDGSLDAGGPDPVRTLTIPPDEQVVFFGVQATTSDSDIVSAIGLGEPDLTEAQVEAGGFAFLGAANMNVSTSGIGIGNNNLD
GDTTAGITAADESFIINPETLLTGMKVFIDNSVGGYDPATEELYYKIFFDDGSNSGNIKVLSGDLTAEAGGQVSFGIETQGSKLIDSVQLTMGKGVVKIP
VIEFIQATENLASDVLLDFTASLTDADGDSATSDFSVDLFANDLEGTFDFTLTGASGVLDSFNIDLSALQDSYKIEGFDTPDDQLALLGDLGATVSIDNS
GADSIVSVTETGGQTTSITVVGVDLVPANIVIA

Var-1 WP_095744550.1
MAALNLTAGSTVTLDESANLQNLAATPAAAGDSNDNDISASSLPTAFSTRLTAQGVGTAINAALSGYNGSNTGTNAFTFSVTGTVTDMSFTDANGAPLNG
LDSGLNTADGEDIFLYTDATNNNVVYGKTALNVVVFAAYLEETGSPVNGAKIWTVQYEAMSNPDAANADDPIDLTDKAFVSVSQDTEFNLADAPSGQNLF
LMFTTGSPTIVDGRISNVSIIATGKDPANQSGANPNSTADDINISTGDTINTSKAGGPTTFGTNSQMITEQEGIRFTFVTGARADVTIPNQDQNEADLES
NIDFTGMFGARTADFDVVQLQSGKSAQVKISAFSTAAESGNSFIDGYAGDATVAITSVRVLNSATGAVLETFSAGAEGGLSSSLAISITGGVATITGVVA
GQSIEYTTSADHNRVLIENGAALNASGNTHADFDIGGFRLLRSSTATAQIGSQMIFEDDGP

Va1 repeats
SIAVADTAPPDALTVDESALATNASASFADNFTSTPSYGADGAGTVTSTYALSLKTSGADSGLDDVATGNSVFLFLNASGVVEGRQGTDAAAAATGDIVF
SIAVADTAPPDALTVDETALATNASASFADNFSSTPTYGADGAGTTSSAYALSVKSAGIASGLVDTLTNQSVVLTVNAG-VVEGR------TAISGALVF
SIVVADTAPPDTLTVDETALATNASASFADNFSSTPTYGADGAGTTSSAYALSVKSAGVASGLVDTLTNQSVVLTVNAG-VVEGR------TAISGALVF
SITVADTAAADPLTVDETILATNASASFADNFTSTPTYGADGAGTVASAYALSVKSAGVASGLVDTLSNQSVVLTLTAGGVVEGR------TAGSGLVVF

TVSVNAAGTVTLDQVRAIVHPNIANPDDSKTLAAADLIVLTRTDTITDRDGDPNTGSDFLNIGQTLNFEDDGP
TVSVDAAGLVTLDQLRAVVHPDATNPDDSKTLAAADLVVLTRTDTITDKDGDQNTGSDFLNIGQALNFEDDGP
TVSVDAAGLVTLDQQRAVVHPDATNPDDSKTLAAADLIVLTRTDTVTDKDGDQNTGADFLNIGQALNFEDDGP
TVSVNAAGLITLDQQRAVVHPDAANPDDSKTLSAADLVVLTRTDTITDKDGDQNTGSDFLNIGQALNFEDDGP

PTIVARTDLIYANTSNPSPGGTGTFDYEIGADARSSYSVSNSDFSAITLTGTVGNAAITSPTVTWTSETATGASFAVSFSYKPDPGSATTTNATGTLSFD
KVNGTYSLALNAPIAGFNTVSTGQALGFTGYTVGTSTVDQTQPDVSVAQLSSNFFVQFTGYSEPGGGTGANNIQAIAPAAPLGTANGTAFVNGELFKQAG
TWVSVSNLANGVAGDTIQQGEVLDLDFFAANPFGFTTATPTTQAAGMFLKFDGIGSEDLVLVLKLVDPDDGSRTTKAIIIDNADIIKSGGTIPAPYSISL
DNNDGAVIIESNDFNTGTENYVIEGAQLLVSTEGVTGTAINLNALTGAGGNSTTLQEFSGTPGTANPEAATSDNDVIKISDIGFVTLNSGQLDANLQFSL
AVKDADGDATATQVLDVGIVGGTAFTGGADNEAIQGSAGNDTLNGGGGNDTLTGGLGKDSLTGGDGNDIYDFNAVAESAAGANRDVVSGFMSGADTLDLS
GIDAISGNANADDAFSYLGDAAFTNVAGQLRFDVATQTLQADIDGNGTADFEVQLVGVVAPPPLDDFFM

Var-3 AGU49274.1
MSVLITTAISTVTLDESAGLQNPVATPAPAEDANDNDILASSLPTVFSSRLTTLGAGAPTKAALSGYTGSNTGANAFTFTASGTVTDVSFTGATGAPLNG
LDSGLNTADGEDIFLFTDTTNNNIVLGKTSAGVIVFAAYLEETGTPVSGAKIWMVQYEAISNPDPTNPDDAVNLLNKVFVSVSQDTEFNLEGAPSGQNLF
LMFTKDNPTIVDGRISEVTIIATGKDPANQSGPNPNITSDDININTGDTINTSQAGGPTTFGTNSQMITEQEGIRFTFVTGARADVTIPNLDQNEADLES
NIDFTGMFGARTADFDVVQLQSGKTAQVKVTAYSTAVESGNSFIDGYAGDTVVSITSVRVLDSVTGAVLESFSGGAEGGASSTIAISITGGVAMITGVKA
GYSIEYTTSADHNRVLVENGATLDATGNTHADFDIGGFRLLQVSAATAEVGSMMFFEDDGP

Va1 repeats
SIVVADTAPPDALTVDETLLATNASASFADNFSSTPSYGADGAGTVTSAYALSLKTSGADSGLVDVATGNSVFLFLNASGVVEGRQGTDATAAATGDVVF
SIVVADTAPPDALTVDETLLATNASASFADNFSSTPSYGADGAGTVTSAYALSLKTSGADSGLVDVATGNSVFLFLNASGVVEGRQGTDATAAATGDVVF
SIVVADTAPPDALTVDETLLATNASASFADNFSSTPNYGADGAGTVTSAYALSVKSAGVASGLVDTLTNQSVVLTVNA-GVVEGR------TSISGALVF
SIVVADTAPPDALTVDETLLATNASASFADNFSSTPNYGADGAGTVTSAYALSVKSAGVASGLVDTLTNQSVVLTVNA-GVVEGR------TSISGALVF
SIVVADTAPPDALTVDETLLATNASASFADNFSSTPNYGADGAGTVTSAYALSVKSAGVASGLVDTLTNQSVVLTVNA-GVVEGR------TSISGALVF

TVSVSAAGLATLDQQRAIVHPDTTNPDDSKTLAAADLIVLTRTDTITDQDGDQSTGSDFINIGQALNFEDDGP
TVSVSAAGLVTLDQQRAIVHPDTTNPDDSKTLAAADLIVLTRTDTITDQDGDQSTGSDFINIGQALNFEDDGP
TVSVDAAGTVTLDQLRAIVHPDTTNPDDSKTLAAADLIVLTRTDTITDQDGDQSTGSDFINIGQALNFEDDGP
TVSVDAAGTVTLDQLRAIVHPDTTNPDDSKTLAAADLIVLTRTDTITDQDGDQSTGFDFINIGQALNFEDDGP
TVSVDAAGLVTLDQQRAIVHPDTTNPDDSKTLAAADLIVLTRTDTITDQDGDQSTGSDFINIGQALNFEDDGP

SIVARSDLIYSNTSNPSPGGTGAFDYEIGADARLSYSSSSSDFSAITLTGTVGSAAITSPTVTWSSETSTGASFAVSFSYKPDPSSANTTSATGTLSFDK
VNGTYSLALTAPIAGFSTVSTGQALGFTGYTQGTQTVDQTQPDVSVAQLSSNFFVQFTGYSEPGGGTGANNIQAIGPVDPLGTADGTAYVNGELFKQAGT
WVSVSNLANGVAGDTIQKGEVLDLDFFAANPFGITTTTPTTQAAGMFLKFDGIGSEDLVVVLKLVDPDDNSRITKAIIIDNADIIKFGGTIPAPYSITLD
NNDGAVIIESNDFNTGSENYVIEGAQLLVSTEGVTGTAINLNPVTGAGGGSTTFQEFSGTPGTANPEAATTDNDVIKISDIGFVTLNSGQLDASLQFSLA
VKDADGDATATQVLDVSIVGGTTFTGGADDEAIQGSAGNDTLNGGAGNDTLTGGLGKDTLTGGDGNDVYDFNAVAESPAGANKDVISGFTSGADKIDLSG
VDAIPGNGDDAFSYLGDGAFTGVAGQLRFDVATNTLQADVDGNGVADFEVQLVGVVVPPPLGDIIV

Var-4 AGU49286.1
MPALNMTAGSTVTLDESATLQNLAATPAVAGDSNDNDISASSLPTAFSSRLTAQGVGTAINAALSGYDGTNTGTNAFTFSVSGTVTDVSFTDANGALLNG

```
FDSGLDTADGEDIFLYTDTTNNNIVYGKTALNVVVFAAYLEETGSPVTGAKLWTVQYEAISNPDASNPDDAVNLADKLFVSVASASEFSFANVPSGQNLF
AMFGNATAAIVVTGKNPANESTGVNINTGDTVNTSLGGGLTTIGTNNQMIDPPSAKNPGEGMYFTFVTGANADDTVPNLDQNEADEESNIDFTGLLGTTE
ASFTIAQLQPNKAATLKISAFTTVLASGDAYVDNLQNNTAINISSVVVRDSAGQLVTGLSIDLSGDTAVISGIKAGYVIEYQTDANHNRVLIENVGSATN
NNLNASFDIGGFSLPKFEQATGEVGSMMVFEDDGP
```

Va2 repeats

```
SVVLAAPTDTALVNTQDADTIGAATDSATQDFSTAFGVASSSYGADGAGTTVSSFALGVATQGGDSGLKSDGATIYLYVVSGKVIGSTSGTEAGILATNT
SVVLAAPIDTALVNTQDADTIGAATDSATQDFSTAFGVASSSYGADGAGTTVSSFALGVATQGGDSGLKSDGATIYLYVVAGKVIGSTSGTEAGILATNT
SVVLAAPIDTALVNTQDADTIGAATDSATQDFSTAFGVASSSYGADGA-TTASSFALGVATQGGDSGLKSDGATIYLYVVAGKVIGSTSGTEAGILATNT
SVVLAAPIDTALVNTQDADTIGAATDSATQDFSTAFGVASSSYGADGAGTTVSSFALGVATQGGDSGLKSDGAVIRLYLVSGNVIGSTAATEADITAGNT
SVVLAAPIDTALVNTQDADTIGAATDSATQDFSTAFGVASSSYGADGAGTTVSAFALGVATPGGASGLTSDGVSIKLYLVSGNVIGSTAATAGAITAGNT

IFDLAVSAIGSVTLQQFAEIDHALPGDSSNYADQEATLADTLITLTNTVTVTDGDGDTASDSKVLNIGANIKFDDDGP
IFDLAVSASGSVTLQQFAEIDHALPGDSSNYADQEATLADTLITLTNTVTVTDGDGDTASDSKVLNIGANIKFDDDGP
IFDLAVSASGSVTLQQFAEIDHALPGDSSNYADQEATLADTLITLTNTVTVTDGDGDTASDSKVLNIGANIKFDDDGP
IFDVTVSGTGSVTLQQFAEIDHALPGDSSNYADQEAALADTLITLTNTVTVTDGDGDTASDSKVLNIGANIKFDDDGP
IFDLAVSATGSVTLRQFAEIDHAGPGVSSNFAAQQATMADTLITLTNTVTVTDGDGDTASDSEVLNIGANIKFDDDGP

TVTAISDLTGANDGLPIAGTYNFSVGADDVDNASTDGIVLNSLTGTTGGGRAITDAVVSHFAEDATTVTYNFSFNYYPGPTSTTTQAATGTVVFNKTDGT
FAFDMDQLIGGQTTFSTSSPLASFNYDTEGNNSPEIVVQQYSSDFFGVLSASSARPPSDSGDLMSGNDHAFTTGEIFTSESTAFVNVATNTLGVNSDTVQ
AGELLNFDFYRSNPVSNPTSTSPPQQPGAAIVGTDKAYADAIDITIDQITDGEDVAILLKLFDASTNTTTTRLLIANSATDYQSAGGGTKIVSIGEDDYN
SAIYQIAGVQVLSSTEDLTGTGISLSTHDTVNLTSAGMNYADTADNDVFKIIKIDVITETTINSDVDLNFAGQLIDGDADYANFDFDVHLEIDGIANLIG
TTNQPTAIA
```

Hal-1 WP_040184700.1

```
MSESLVNITGTDVTIDETAGLQNSGSTPPGDADDDDVLFSTIPELFSLRLTALGATDPALGAAESDGDVVTFSPTGELGNVALTDADGALLDGDDSGLKT
TDGESILLYTDTDNDNIVLGKTAGGTLVFAIYLEETGGTATTGPTGGKLWTVQYEAIYHDGGDTNLDGDGNNHDSAVDLTGLVHVTAFEAQTFSFENAPS
GSNLFMAFGDASQALVITGENPADESSGENVSSGSTVNSSQGGGTTTLGIDGQQIKAQQAIVVTFVTGAEADYLAGPAAGNDPGQPLSPTEALDEANIQF
SGYVLAQAAQFTISQMTPGNTNTTTSLEINAYLTADGSGDHYIDGNPLVQGDSAVNITTDSVEVLRGDVNVVGTLGVTVNYTEDGVVVMGVKNGDVVRYE
TDADHNRVLIRNDQPEKGSGSNVAFDLGGFTLTEVAGDTDEIGSKIFFEDDGP
```

H1 repeats

```
AIALTSLAPTIAVDESLGTAGSSKDEPGNAAPDDETNANAPAGAIGYAVTTAATLFTETADAGTDGEASKVYALVLNAGASGLTDTASGQAVVLSDNSGV
AIALTSLAPTIAVDESLGTAGSSKDEPGNAAPDDETNANAPAGAIGYAVTTAATLFTETADAGTDGEASKVYALVLNAGASGLTDTASGQAVVLSDNSGV
AIALTSLAPTIAVDESLGTAGSSKDEPGNAAPDDETTTGAPAEAIGYAETAASVLFSETADAGTDGEASKVYALVLNAGASGLTDTASGQAVVLSDNSGV
AIALTSLAPTIAVDESLGTAGSSKDEPGNAAPDDETNANAPAGAIGYAETAASVLFSEVADAGTDGEASKVYALVLNAGASGLTDTASGQAVVLSDNSGV
AIALTSLAPTIAVDESLGTAGSSKDEPGNAAPDDETTTGAPAEAIGYAETAASVLFSETADAGTDGEASKVYALVLNAGASGLTDTASGQAVVLSDNSGV
AIALTSLAPTIAVDESLGTAGSVKDEPGDAANNDETTTGAPAEAIGYAVTTAATLFSETADAGTDGEASKVYALVLNAGASGLTDTASGQAVVLSDNSGV
AIALTSLAPTIAVDESLGTAGSSKDEPGNAAPDDETNANAPAGAIGYAVTTAATLFTETADAGTDGEASKVYALVLNAGASGLTDTASGQAVVLSDNSGV

IEGRTDDANADLVFSIAVDATTGDVTTTQYRALDHGADSNDHDSAVSMAAGLVELEATLTDGDTDTASSKIELGSLIGFEDDGP
IEGRTDDANADLVFSIAVDATTGDVTTTQYRALDHGADSNDHDSAVSMAAGLVELEATLTDGDTDTASSKIELGSLIGFEDDGP
IEGRTDDANADLVFSIAVNGTTGDVTTTQYRALDHGDDGNDHDIAVSMTSGLVELEATLTDGDTDTASSKIELGSLIGFEDDGP
IEGRTDDANADLVFSIAVDATTGDVTTTQYRALDHGADSNDHDSAVSMAVGLVELEATLTDGDTDTASSKIELGSLIGFEDDGP
IEGRTDDANADLVFSIAVDATTGDVTTTQYRALDHGADSNDHDSAVSMAAGLVELEATLTDGDTDTASSKIELGSLIGFEDDGP
IEGRTDDANADLVFSIAVDATTGDVTTTQYRALDHGADSNDHDSAVSMAAGLVELEATLTDGDTDTASSKIELGSLIGFEDDGP
IEGRTDDANADLVFSIAVDATTGDVTTTQYRALDHGADSNDHDSAVSMAAGLVELEATLTDGDTDTASSKIELGSLIGFEDDGP

AIAVSNASGTYATPAEATGDFVDGSGTDGFGDLAITFNWYQIDGEGLIQTSGNPNVTFVESVTDTTWAGTIFDDFNGDGVSETVAFTLDVDLDTNEYTVT
LDTPPAQETTTSTADGSLDAGGPDAVRTLLFGGDDAGSDDIVFFGAVATAPRSDVLGGNPYPAVPNDIEDLVVEGEPDLTEAQIEGFLTPTNQIPTLINA
STQMNVSTSGIGINNNNLDGDGNDGGSGAFFGTSITSGDESFVVNPEPLVDSVTVYISSTVQGYDPETEDLYYTVYYADGTIDSPQKVTEDDLTRYANND
VNVPKEAKGGSSFTIEDEDGDRQIDAVQLTMGLGTIKIPVIEFNTLTQFDPESLELNFTATLEDGDEDTATDDFTVDFSPEPPAELSYVSLDDPDVLL
```

Hal-2 BOX17_03680

```
MDGVDSGFDRTSTGNDILLYTDTDNDNIVLGRDEMTDEIVFAIYLTEPAGDPLESSIWVVEYDAIEHDGADQLNDGDGNDHDASYDLANLVYVTAFESQE
FSFAGAPPGSNLFMWFGDLDQSIIVTGRDPANQSEGENISSGDTVNTSGNTDTSLGTNGQQVKAGEGLYFTFASGSTSSTPDDYIVPNLSQTEADVEANI
TPLALTDARGASVTVSQVNPGGNTTVSMRITAYTTDLETGVNFLDGLGDADDVPVLITGVTINGTSVSFTTDGDGVIVSGVKAGDVVGYTTVADHQRVLI
ENAQPESGKGSNVSFDLGGFALTEVAGDTDEIGSKISFYDDGP

GDPTVALSGDDPVGLTFDGGLQPDGNFTGAESSDDTNAS-PTIAVVDFSDAFTIGNLNDFGADGAGESVVSYALQLA--VAEGTASGLTSDGDPIRLYIN
ADPTVALSGAEPVGLTFDGGLT-DGNFTGTESGDDTSTSTPLVAVVDFSTAFTIGIQDDFGADGGDSTVVSYALQLATGVAEGDPSGLTSGGDPIRLYIN
ADPTVALSGAEPVGLTFDGGLT-DGNFTGTESGDDTSTSTPLVAVVDFSTAFTIGIQDDFGADGGDSTVVSYALQLATGVAEGDPSGLTSGGDPIRLYIN
ADPTVALSGAEPVGLTFDGGLT-DGNFTGTESGDDTSTSTPLVAVVDFSTAFTIGIQDDFGADGGDSTVVSYALQLATGVAEGDPSGLTSGGDPIRLYIN
ADPTVALSGAEPVGLTFDGGLT-DGNFTGTESGDDTSTSTPLVAVVDFSTAFTIGIQDDFGADGGDSTVVSYALQLATGVAEGDPSGLTSGGDPIRLYIN
ADPTVALSGAEPVGLTFDGGLT-DGNFTGTESGDDTSTSTPLVAVVDFSTAFTIGIQDDFGADGGDSTVVSYALQLATGVAEGDPSGLTSGGDPIRLYIN
ADPTVALSGAEPVGLTFDGGLT-DGNFTGTESGDDTSTSTPLVAVVDFSTAFTIGIQDDFGADGGDSTVVSYALQLATGVAEGDPSGLTSGGDPIRLYIN
ADPTVALSGAEPVGLTFDGGLT-DGNFTGTESGDDTSTSTPLVAVVDFSTAFTIGIQDDFGADGGDSTVVSYALQLATGVAEGDPSGLTSGGDPIRLYIN
ADPTVALSGAEPVGLTFDGGLT-DGNFTGTESGDDTSTSTPLVAVVDFSTAFTIGIQDDFGADGGDSTVVSYALQLATGVAEGDPSGLTSGGDPIRLYIN

DDGTLVTGSTAGDEASVAAGNTVFTLALDTTTGVLTQTQSAVIDHGTTDTYEGAYIVDERALANGLVELVASAETTDGDHDVSNVVSSTLDLGGNVRFGDDGP
DDGTLVTGSTDTSEGA-GDGFTVFTLALNATTGVLTQTQSAVIDHGTTDEYEGAYIVDVRELANGLVELVASAETTDSEGDTSGVNSEALDLGGNVRFGDDGP
DDGTLVTGSTDTSEGA-GDGFTVFTLALNATTGVLTQTQSAVIDHGTTDEYEGAYIVDVRELANGLVELVASAETTDSEGDTSGVNSEALDLGGNVRFGDDGP
DDGTLVTGSTDTSEGA-GDGFTVFTLALNATTGVLTQTQSAVIDHGTTDEYEGAYIVDVRELANGLVELVASAETTDSEGDTSGVNSEALDLGGNVRFGDDGP
DDGTLVTGSTDTSEGA-GDGFTVFTLALNATTGVLTQTQSAVIDHGTTDEYEGAYIVDVRELANGLVELVASAETTDSEGDTSGVNSEALDLGGNVRFGDDGP
DDGTLVTGSTDTSEGA-GDGFTVFTLALNATTGVLTQTQSAVIDHGTTDEYEGAYIVDVRELANGLVELVASAETTDSEGDTSGVNSEALDLGGNVRFGDDGP
DDGTLVTGSTDTSEGA-GDGFTVFTLALNATTGVLTQTQSAVIDHGTTDEYEGAYIVDVRELANGLVELVASAETTDSEGDTSGVNSEALDLGGNVRFGDDGP
DDGTLVTGSTDTSEGA-GDGFTVFTLALNATTGVLTQTQSAVIDHGTTDEYEGAYIVDVRELANGLVELVASAETTDSEGDTSGVNSEALDLGGNVRFGDDGP
DDGTLVTGSTDTSEGA-GDGFTVFTLALNATTGVLTQTQSAVIDHGTTDEYEGAYIVDVRELANGLVELVASAETTDSEGDTSGVNSEALDLGGNVRFGDDGP

AVTVNDASGTYQAGADGTWSDLPGADGFDSLNVTFDSFEIDANGTQTTSTTNSTFARTGNLSWAGSVTGDFTDDGVINEQTVDFSLTFDPVADTYSIVID
DLPTSTSTFDTSQGTLKAGGPDEVRTLLFGGEPPTDAGNDDIVFFGAVPTAVRSDPAEPVDIDSILDLVVRGELDLTEEEIEALLPLPDLVNPGTQMNVS
TAGIGINNNNLDGADEGDGTGAFAGTTITSGDESFVVNPETLVDSVTVYISSTVQGYDTATEDLYYTVYYADGSVSGPILVEELDLTHYANNDTSVPKEA
KGGSSFTIESGENQVNQIDAVQFTMGLGTVKIPIISFDVETEFEPEPLAMDFTATLEDGDNDTATDDFTVNLL
```

Figure S4. Alignment of empty and filled DDxP proteins.
Empty and filled proteins from Pseudomonas and Rhizobium are aligned
DDxP motifs are highlighted. High consensus sequences are in red.

PSE-4/PSE-5

```
PSE-4  MTTPQSPTTDLTADTPPDLYPVFPSNDTLTITALADGAPLVVATGTNVALDETSGLQNATATPDPTGDADDNDILLAALPSAFATRLTALGAGTATDAALSGYTGALGDTGSNAFTLNLA
PSE-5                           MTTLQENLSLTSDPLVVATGVNVALDETTGLQNATTTPAPAGDADDNDILLAALPSAFATRLTALGAGTATDAALSGYTGAVGDTGSNAFTLNLA

PSE-4  PGAINVDISFTDSFGAALNGLDSGLDTLDGIDILLYTDTDNNILLGRAGGPDGAIVFAAYIEETGSPLSGGKIWTVEYQPLKHPDGSNPDDALNLLDKVFIGASQDLTFSLADAPSGQNL
PSE-5  PDAINVDVGFTDSLGAPLDGLDSGLDTLDGIDILLYTDTDNNILLGRAGGPDGAIVFAAYIEETGSPLSGGKIWTVEYQPLKHPDGSNPDDALNLLDKVFIGASQDLTFSLANAPSGQNL

PSE-4  FLMFTKANPTVVDDGGVLRISDPTIIATGKDPADQSSGASINTGDTINTSQAGGPTTFGTNSQMITEQEGIRFTFVTGARQDMTIPNLDQNEADLEANIDFTAMFNAKAANFDIVQLQGG
PSE-5  FLMFTKANPTVVDDGGVLRISDPTIIATGKDPADESTGVNINTGDTINTSQAGGPTTFGTNNQMIVEQEGIRFTFVTGARQDVTVPNLDQNEADVEANIDYTAVFNATTASFDVVQLQGG

PSE-4  KSAVVRISAFNTAVESGASFVNGYANDTSVAITNVRVINIATGLVIENSDGSVNDTSILISFDGGVATITGVKAGYQIEYTTAADHNRVLIENGAALDAKGNTHADFDIGGFTLRQASTT
PSE-5  KSAVVKVSAFSTAVETGADFVNGYADDTSVAITNVQVIDNATGLVIENSDGSVNDTSILISFDGGVATITGVKAGYQIEYTTSANHNRVLVENGAAVDAKGNDHADFDIGAFTLRQATTA

PSE-4  TAEIGSQMIFEDDGP---------------------------------------------------------------------------------------------------------
PSE-5  TAEIGSKMIFEDDGPAAAGTAEAGTVDEDGLANGIAGGVGDVPGELTTAGGNVSGIFQSGVDVPLTYSLSSDTSGLPALTSGGVALVYSVVGDTLTAKAGAVDVFTFSLSAAGAYSFTLL

PSE-4  ------------------------------------------------------------------------------------------------------------------------
PSE-5  QPLDHPAGNDENDITINLGSLLQATDKDGDTVTAAAEKLVITVDDDTPTANGTAGAGTVDEDGLANGIAGGVGDVPGELTTASGNVTGIFQSGADVPLTYALSSDTSGLPALSSGAVALV

PSE-4  ------------------------------------------------------------------------------------------------------------------------
PSE-5  YSVAGGTLTAKAGAVDVFTFSLNAAGDYSFTLLQPLDHPAGNDENDITINLGTLLQATDKDGDTVTAAAEKLVITVDDDTPTASGTAAAGTVDEDGLANGIASGVGDVAGEATTAIGSVT

PSE-4  ------------------------------------------------------------------------------------------------------------------------
PSE-5  GIFQSGADVPLSYSLSSDTSGLPALSSGGVALVYSVAGDTLTAKAGAVDVFTFSLSAAGDYSFTLLQPLDHPAGNDENDITLNLGTLLQATDKDGDTVTAAAEKLVITVDDDTPTANGTA

PSE-4  ------------------------------------------------------------------------------------------------------------------------
PSE-5  EAGTVDEDGLANGIAGGVGDVAGESTTANGSVTGIFQSGADVPLTYALSSDTSGLPALSSGGVALTYSVTGDTLTAKAGAVNVFTFSLNAVGAYAFTLLQPLDHAAGNDENDITINLGSL

PSE-4  ---------------------------TLAFGNLIGTGTQLAQQGYWNMGSGADGLDANGLDISLVNDQFTLVRPDNTTTTGTGTLVEQSPSPDGNGAYQFAGTLTGDFDNNAATADTTV
PSE-5  LQATDKDGDTVTAAADKLVITVDDDTPTLAFGNLIGTGTQLAQQGYWNLGSGADGLDTNGLDISLVNDQFTLVRPDDTTTTGTGTLVEQSPSPDVNGAYQFAGTLTGDFDNNAATADTTI

PSE-4  HYTLSAYANGTYALDLEEGFRSTIVLSSADGSLAAGGPDPVRTLMIGSEDVVFFAANPLAPQTGDNSILTGIGLGAPDPTEAQLQTNPLPSFIGSSTMNVSTSGIGVANNNLEGNTTAGI
PSE-5  HYTLSAYANGTYALDLEEGFRSTVVLSSADGSLDAGGPDPVRTLTIGTEDVVFFGANPLAPQTGDNSILTGIGLGAPDPTEAQLQTNPLPSFIGSAAMNVSTSGIGIANNNLEGNNTAGI

PSE-4  NAGDESFVINPETLLTSMKIFIDNSVQGYNPATEELYYTIYYDNGTTSGTPIKVQAADLHTETGGQKSFTVQWDGSHLIDAVQLTMGLGTVKIPVIQFIKETQSLANDIQLSFNATTTDK
PSE-5  NAGDESFVVNPETLLTAMKVYIDNSVQGYNPATEELYYTIYYDNGTTSGAPIKVQAADLQAEAGGQTSFTVEWDGTHLIDAVQLTMGKGTIKVPTIEFIHEVQSLASDIQLAFNATVTDK

PSE-4  DGDTATSAFDANLFANDPSDQTFDFRLVGTTGERDAFNIDLSVAENQYQVTGFDAGPGQRDAVVLIGDAGATVQSIDNASNDSIVTIAETGGQLTTITLLGVDLLNTDIVMASV
PSE-5  DGDTATSAFDANLFANDPANALFDFRLMGTTGERDAFNIDLAATENQYQVTGFDAGAGQRDALVLIGDAGAVVQSVDNAGSDSIVTVAETGGQITTITLVGVDLLNTDIVMGSV
```

PSE-16/PSE-17

```
PSE-16  MAIEITGEDVVLDETPGLQNATATPTPAGDADDNDILVASLPSDFSTRLTALSAGTATGAALSGYTGAVGNTGSDAFTVTNGESITDISFVDSTGAPLDGADSGLFTLDGTSILLYTDTN
PSE-17             MDETAGLQNATATPTPAGDADDNDILVASLPSSFSTRLTALGAGTATGAALSGYTGAAGDTGSNAFTFTGGGSITDIRFVDSAGAPLNGVDSGLDTLDGTSILLYTDTD

PSE-16  NNILLGRAGSATGAIVFAAYIEETGSPVSGGKIWTVEYQPLQHPDGTNPDDSLNLLDKVFVGTSQDLEFSLAGVPSGANLFLMFTKANPTVVDVGGVLRITDPTIIATGKDPADESSGAN
PSE-17  NNILLGRAGGADGAIVFAAYIEETGSPVTGGKIWTVEYQPLKHPDATNPDDSLNLLDKVFIGVSQDLGFSLAGAPSGQNLFLMFTKLNPTTETVDGVVRITDPTIIATGKNPADQSSGAN

PSE-16  INTGDTIITSQAANPTTFGTNSQMITEQDGIRFSFVTGARQDVTIPNLSPTEADVESNIDFTDVVNAKKASFDVVQLQSGKSAVVKISAFSTAAEPDVNFINGYANDTPVAITNVRVFN-
PSE-17  INTGDTINTSQGGGPTTIGTNSQMITEQEGIRFSFVTGARQNVTVPNLSQTEADVESNIDFTSVYDATSASFDVVQLQSGKSAVVKISAFSTAAEPGVNFINGYTGDAPVAITNVSVINI

PSE-16  SAGVVIENSDGSVNDPTIGISFSGGVATVTGVLAGYQIEYTTTEDHNRVLVENGAALDARGNTHADFDIGGFTLLQTAVTTAEIGSKIIFEDDGP-------------------------
PSE-17  STGLVIENSDGSVNDPAIGISFSGGVATITGVLAGYQIEYTTTADHNRVLIENGAALDARGNDHADFDIGGFTLREASTTIAEIGSKMIFEDDGPSISATGTEPTLTVDETVLATDATQN

PSE-16  ------------------------------------------------------------------------------------------------------------------------
PSE-17  FAANFTSAFGADGPGTLTYALGVVAGASGLTDTATGEAVNLSLNGTVVEGRTATTNLLVFTVSVAANGDVTLDQQRAVVHPDATNPDDSTSLTLDDLVTLTATTTDGDGDSVQATLNIGS

PSE-16  ------------------------------------------------------------------------------------------------------------------------
PSE-17  NLVFKDDGPSITATGEEPTLTVDETVLTTDATQNFAANFSSAFGADGAGTLTYALGVVAGASGLTDTATGEAVNLSLNGTVVEGRTATTNLLVFTVSVAANGDITLDQQRAVVHPDATNP

PSE-16  ------------------------------------------------------------------------------------------------------------------------
PSE-17  DDSTTLSADNLVTLTATTTDGDGDSVQATLNIGSNLVFKDDGPSITATGEEPTLTVDETVLTTDATQNFAANFSSAFGADGAGTLTYALGVVAGASGLTDTATGEAVNLSLNGTVVEGRT

PSE-16  ------------------------------------------------------------------------------------------------------------------------
PSE-17  ATTGLLVFTVSVAANGDVTLDQQRAVVHPDATNPDDSTSLTSDDLVTLTATTTDGDGDSVQATLNIGSNLVFKDDGPSITTTGEEPTLTVDETVLATGATQNFAANFTSAFGADGPGTLT

PSE-16  ------------------------------------------------------------------------------------------------------------------------
PSE-17  YALGVVAGASGLTDTATGEAVNLSLNGTVVEGRTATTNLLVFTVSVAANGDVTLDQQRAVVHPDATNPDDSTSLTSDDLVTLTATTTDGDGDSVQATLNIGSNLVFKDDGPSITTTGEEP

PSE-16  ------------------------------------------------------------------------------------------------------------------------
PSE-17  TLTVDETVLTTDATQNFAANFTSAFGADGAGTLTYALGVVAGASGLTDTATGEAVNLSLNGTVVEGRTATTNLLVFTVSVAANGDVTLDQSRAVVHPDATNPDDSTSLSADNLVTLTATT

PSE-16  ------------------------------------------------------------------------------------------------------------------------
PSE-17  TDGDGDSVQATLNIGSNLVFKDDGPSISTTGEEPTLTVDETVLATNATQNFAANFSSTFGADGAGTLTYALGVVAGASGLTDTATGEAVNLSLNGGVVQGRTATTNLLVFTVSVAANGDV

PSE-16  ------------------------------------------------------------ALAFGNLIGTGSVLPQTGFWSNSAGADGLDAAGLDISVNSQFTLVRPNNTTTTGTATLTE
PSE-17  TLDQSRAVVHPDATDPDDSTSLSADNLVTLTATKTDGDGDSAQATLNIGSNLVFKDDGPPALAFGNLIGTGSVLPQTGFWSKSAGADGLSAAGLDISVNSQFTLVRPDNTTTTGTATLTE

PSE-16  VVPSPDGNGAYQFAGTLTGDFDNNADTADTTVDYTLTAFDDGSYALDLVQGFSSTVVLSTADGSLAAGGPDPVRTLLIPDTNDPTIPSASEEVVFFSAKALASTTDILAGIGLGAPDPTE
PSE-17  LVPSPDANGAYQFAGTLTGDFDNNAATADTSVDYTLTAYADGRYALDLVQGFSSEIVLSTADGALGAGGPDPVRTLLIPEQDPPTIPSPSEEVVFFSAKALASTSDILTGIGLGEPDPTE

PSE-16  AELQTTPLPSYIDPRAMNVSTSGIGVANNVLQGDNLVAIGATDESFVVNPESLVTAMKVFIDNSVAGYNTATEDLYYRIYYEDGTTSNLIEVNTLTPEPRGQVSFLIEQEGSLLIDAVQL
PSE-17  AQIQTNPLPSYIDPSAMNVSTAGIGVANNLFQGDNLAAIGVDDESFVVNPESLLTGMRVFIDNSVGGYNTATEDLYYRAYYEDGTFSNLIEVNTLTPEAGGQVSFLIESDGTNLIDAVQL

PSE-16  TMGRGDIKIPVIQFIQETESLASDIQLTFNATVTDRDGDSASSTFDANLFANDLAGA-FDYTLVGTDDELDAFNVDLSFDENLYQITGFDTDANLRDTLVLNGDQSAVVQ-IDTSGADSI
PSE-17  TMARGEIKIPTIQFIQESESLASDVQLTFNATLTDKDGDSATSTFDANLFANDAVDALFDFSLVGTGGERDAFNIDLSVDENLYQVTGFDANANLRDALVLNGDQSAVVQSIDISGADSI

PSE-16  VTVTETGGDVTTITLVGVDLLSSDIVLGSA
PSE-17  VTVAETGGQVTTITLVGVDLLSSDIVYGSV
```

Rhi-8/ Rhi-9

```
RHI-8  MAIVITSTGVFAVLDETDGLQNATATPAPPSPSGDADDNDILSSSLPGPFSTRLTSYSL-TVDEAALSGYTGAAGNTGANIINITGATATTDLALTGANGAAFPDYETGATSAFSSGLFA
RHI-9  MAIAITSTGAFVVLDETEDLQNATATP---SPAGDADDNDT-STPLPAAFSTALTSHGVVTIAEAALSGHNGAAGNTGANIITVTGATATTDFAFRGENGAAFTAYEAGATSTLNSGLSA

RHI-8  VADDGTISEIFLFTDPTNNNIVYGVAGDSGDDPIVFAVYLEEVKT-SGITTGAKMWSVLADGYTLAHTTDGSSVAAHDDFLDLTNKLFVSAIAENDFSFANAPSGQNLFMMFGNTTLAIL
RHI-9  VAPDGTITEIYLFADPDDNNIVYGVAGDSGDDPIVFAIYLEEVKNASNITIGAKMWTVLAEGYTLAHTTDD-----HDESLDLADKLFVAAVAENDFSFANAPSGQNLFMMFGNTSLAIL

RHI-8  VTGEDPANQSAGQNVSNDGDTVNTGQGGGATTLGTEGQQIKAQKALVVTFVTGANSNFLVPGLTPTEANLESNIAFSGYAQDVTGAAITISQMTPGSASTTASVELQAFLTADTSGTGYI
RHI-9  VTGEDPADQSAGQNVSNDGDTVNTGQGGGATTLGTEGQQIKAQKALVVTFVTGANSNFLVPDLAPTEANLESNIAFTGYAQDVTGAAFTISQMTPGNANTTATVELQAFLTADASGTGYI

RHI-8  DNSPLTTGDAAVAIASVQVIRGGVDVTGTLGVNVTISGGIATIMGVKDDDIIQYTTTGDHNRVLIRN-QPATGQGSNVAFDLGGFTITQVSSDVEEVGSKVRFEDDGPDAVAKNVAGPTA
RHI-9  DNNPLTTGDAAVAIASVQVIRGGVDVTGTLGVNVTISGGIATIMGVKDDDIIQYTTTGDHNRVLIRNDQPATGQGSNVAFDLGGFTITQVSSDVEEVGSKVQFEDDGP------------

RHI-8  TLDESPLPTSGDGVNSVTIAATTIAALFETPDFGEDGQGSVSYTLSGTAGAQTGLWLTGESGAANEILLVKVSDTQWEGRKAGAGGTLAFTVSINGTTGAVTVTRSSATLEHTVDGSSAA
RHI-9  ------------------------------------------------------------------------------------------------------------------------

RHI-8  AHDDALTMAATANLFVVQHVTDGDGDTDSATATNPLTIKFEDDGPDAVAKNVTGPTATLDESPLPTAGDGVNSVTIAATTIAALFETPDFGSDGQGSVSYTLSGTDGAQTGLWLTGESGL
RHI-9  ------------------------------------------------------------------------------------------------------------------------

RHI-8  ANEILLVKVSDTQWEGRKAGAGGTLAFTVSINGTTGAVTVTRSAATLEHTVDGSSAAAHDDALAMAATANLFVVQHVTDGDGDTDSATATNPLTIKFEDDGPDAVAKNVAGPTATLDESP
RHI-9  ------------------------------------------------------------------------------------------------------------------------

RHI-8  LPTAGDGVNSVTIAATTIAALFETPDFGADGQGSVSYTLSGTAGARTGLWLTGESGLANEILLVKVSDTQWEGRKAGVGGTLAFTVSINGTTGAVTVTRSAATLEHTTDGNSAAAHDDAL
RHI-9  ------------------------------------------------------------------------------------------------------------------------

RHI-8  TMAATANLFVVQHVTDGDGDTDSATATNPLTIKFEDDGPAVTVDNSSGTYDAGAQGTWNDNDPGSDGFESLSVNFDSYEIDGDGLVTVDAALTKT-----GEFTFTGSITDDFNSDGADE
RHI-9  ---------------------------------------AITVDNSSGTYDLGAQGIWSD-DPGSDRFDSLEVTFDGYSIGGSAVTVDTSLGTKNETDVNGNYVFTGSITDDFNGDGADE
```

```
    RHI-8  TVGFTLTFDPVN------DTYDLDVTTPPPTTRTFDTSQGSLKAGGPDAVQTLLFGGSEAGADDIVFFGVVATAPIEGVSPPPPSANDIEDLVVVDPINGTDLTEAQIEGLFPIASLINP
    RHI-9  TVGFTLTLDPNDPDDPDDNTYDLDVTTPPPTTRTFDTSQGSLKAGGPDAVQTLLFGGSEAGADDIVFFGVVATAPIQGVSSPP--TNDIEDLVVE---GAGDLTEAQIEGLFPIPSLINP

    RHI-8  STQMNVSTSGIGINNNNLNGASEGAGTGAFAGTTITSGDESFVVNPETVVDKVTVFIDNSVGGYNPATEDLYFTVYYTDGTVQAATKVAAGMLTPVTSGVAAGGFSFEIDGGAKQIDAVQ
    RHI-9  STQMNVSTSGIGINNNNLNGASEGNGTGAFAGTTITSGDESFVVNPETVVDKVTVFIDNSVGGYNPATEDLYFTVYYTDGTVQAATKVAAGMLTPVTSGVAAGGFSFEIDGGAKQIDAVQ

    RHI-8  LTMGQGTIKIPVIAFSVEQVFDPEALQLDFTATLFDGDGDSSSDTFSIDLVEAVV
    RHI-9  LTMGQGTIKIPVIAFSVEQVFDPEALQLDFTATLFDGDGDSSSDSFSIDLVEAVV
Consensus  LTMGQGTIKIPVIAFSVEQVFDPEALQLDFTATLFDGDGDSSSDsFSIDLVEAVV
```

Figure S5. Alignments of Mus (panel A) and group II (panel B) DDxXP proteins. In all proteins, repeats when present, were removed. Homologies in the C region of Var proteins to Pse-36 are highlighted.

A) Mus proteins

N regions

```
Mus-3   MALDIVAQDLIIDETSGLQDDDTSAT------NATVTYLLSLDSAGGLASPQVASQADFVVASASAGETISSIILTQNSGGTPFSTSVGVNSGIKTVDGNYVWLFQDATHSNVVIGVIGTSDPAAAPAATGPLAFSFALVSTSATHADLYTVQYVPLLHPDTNDPDDRIDLTDKVFASVS
Mus-15  MALDITTQELVIDETTGLQDDDSSVS------NSTVTYLLSLDSAGGLTSPEVAVQADFVVASASAGETITSVVLTQDSNGTPFSTTVGVNSGIKTVDGNYVWLFQDATHANVVIGVIGTSDANAAPDPTGPLAFSFALVSTSATHADLYTVQYVPLFHPDDTNPDDRIDLTDKVFASVE
Mus-11  MALDILAQDIIVDESTDLQDDDINPS---GNTNPTLLYLLSLDDPGGLTSPEVAHQANFVQASASAGETISGIVLAQDASGTPFSKTVGVNSGIQTVDGNYVWLFQDPTNPNVVIGVIGTSDPLAEPDETGPLAFAFGLDPTSATKADLYLVQYVPLLHPDETNPDDRIDLTDHVFASVS
Mus-14  MALDIQTQDIIIDETTLLQDDDVNPSLAPHVTNPTLLYLLTLDDPGGLTSPEVAHQADFVQASASAGETISGIVLAQDASGTPFSTTVGVNSDLRTVDGNYIWLFQDPNNANVVIGVIGTSDPLAEPDETGPLAFAFGLDPTSATKADLYLVQYVPLLHPDATNADDRIDLTDHVFASVT
Mus-2   MALDLVAQDIIIDETTGLQDNDVNPSLPPHSTNTTLSYLLGLDGPGGLASPEVAFKANFVQATASAGETITSVTLSQNLAGNAFSTTVGVNSGIRTVDGNYVWLFQDATHANVVIGVIGTSDPAAAPAATGPLAFSFALLNTSNTNADLYTVQYVPLLHPDATNPDDRIDLTDKVFASVA
Mus-1   MALDIQTQDIIIDESTGLQDDDVNPSLPPFDTNTTVQYLLGLDDSGGLTSPEVAFQSDFVQVTASAGETITSVVLSQNLNGTPFSTTVGVNSGIQTVDGNFVWLFQDTTHANVVIGVIGTSDAATMPAPTDPLAYSFALINTSATHADLYTVQYVPLLHPDATNPDDRIDLANKVFASAA
Mus-18  MALDILAQDIIVDETTGLQDDDVNSSLSPHN-NATVQYLLSLDSAGGLSSPQVAFQADFVQATASAGETITTVVLAQSVSGTPFSTTVGVNSGIQTVDGNYVWLFQDATHANVVIGVIGTSDAAAMPAATGPLAYSFALMSTSATTADLYTVQYVPLLHPVATDPDDRIDLTDHVFAAVS

Mus-3   ASTVVNFSQLGDAPPGHNDWYILDADVASTQKILVTAHDTGVQSEVNVSTQGLGVASQDVRFGRELQIDLISGGTQSAGKDFTNSPTAPNYTAHLDNISSAGFSISQSTPT--NTKANIEIHAYNNDDNAKGAAFPGDDNDTEINITGATFK-LNGVATTAAALGITVDLSGTGMILLNV
Mus-15  GTSTVSFLG-SNAAPGNRDFYLIDSSSDASKQVLVIGLNG---LTANVSEQGFGVKNQSINPGETLQVDFVTGGTQPAGTD----ASEIQYGSHIETITQAGFTINQITPSQPDKRVDITISAFNNTGNDQGSNFYDGTATTAVDITSIKLTGASGFASIITADG-TY-ATGSGNVTITG
Mus-11  GTTVLNFSG-QNAAPGNHDFYAINSSNDASKQLLVTGFVGAANATANVSTQGFGVNNQSINPTEVLQVDFVTGSTLAAGS-----GSQIQYGSHLDNVTQAGFTINQITPSAETRRVDITIKAFDNTGNEQGSDFFNGTTTTAVDITSIKLTGTSGFATTITADG-TY-ATGSGNVTISG
Mus-14  GTSVLSFLG-QNAASGNHDFYVINSSTDASKQLLVTGFVAAANATANVSTQGFGIGNQSINPTETLQCDFVTGGNLPAGS-----GSQIQYGSHLDNVTQAGFTINQITPSAETRRVDITIRAFDNTGNEQGSDFFNGTTTTAVDITSIKLTGTSGFATTITADG-TY-ATASGDVTISG
Mus-2   GTSVANFSG-QNAPPGINQFYLLDSSNDASKQILVTGFLGTANATPNVSTQGFGVDNQSINPTETLQVDFVTGGTLHAGS-----ASQIQYSSHLETITQAGFTINQITPSNPNLRVDIKISAFNVQGNEQGLNFYDGSPTTTAPITSLKLTGQSGFASPITANG-TY-ATGSGNVTVSG
Mus-1   GTTTVSFSG-QNAAPGNHDFYVINSTTDASKQVLVTGFLGANNATANVSTQGFGINNQSINPTETLQVDFVTGGTLPAGS-----GSQIQYGSHLDNVMQAGFTINQITPSNPNLRVDITIRALDVTGNEQGSDFFNGSPTTAAPITSLKLTGQSGLASPIIADG-TYDVAGNVDITVSG
Mus-18  GTTTVSFSG-QNAAPGNHEFYVINSPSDAAKQILVTGFLGAANAQANVSTQGFGVNNQSINPTEKLQVDFVTGGTLAAGS-----ASQIQYGSHIETITKAGFTVNQITPSNPNLRVDITISAFNVQGNEQGLNFWDGSPTTAAPITSIKLTGQSGFASPITADG-TYDVAGNVDIVVSG

Mus-3   --GEG-----------VTVDFTTAGGASGSFDRFTIKNIDPDKDYFDVKEVHFTSDTPSAYTEQVGSFINFDDDGP   Repeat region
Mus-15  LSGTGNAVTITGLDNVTTVDITTA----SPMDRLQVAGVDANEGC-DITEFHYSVTSTNAHTEEVGSFINFDDDGP
Mus-11  LTGTGNAVTITGLDNITTVDIATA----TQMDRLTVTGVDATEGC-DITEFHFTSQSTNAHTEEVGSFINFDDSGP
Mus-14  LTGTGNAVTITGLDNVTTVDFTTA----TQMDRLTVTGVDATEGC-DITEFHFTSQSSNAHTEEVGSFINFDDSGP
Mus-2   L-GTN-VVTITGLDNITTVDVTTG----SPMDRLTVTGVDANEGC-DITEFHFTTQTSNAYTEQVGSFINFDDDGP
Mus-1   M-GTG-VVTITGLDNITTVDVTTS----SQMDRLTVTGVDSNEGL-DITEVHFTSQTPNALTEEVGSFINFDDDGP
Mus-18  L-GTN-AVTIRGLDNITTVDITTS----SPLDRLTITGVDSNEGL-DVTEFHFSSQTPNAYVEQVGSFINFDDDGP
```

C regions

```
Mus-3   TITAPFDADPGTAGIQTPETLGNSAGATASGTFGYDIGHDSRPTSFYDATHSDFVDSNSTLAGVQIDLTGTVDNSQNPNITNAVATLTAESATSATFGFTFHYDKDPITAGVQDSTAGGTLVFDKVADTYTITLTDPIDGFSFDVLHTADLVAKQPTGNTGHPLIVAEQLTPNADP----
Mus-15  IITAPFDADPAAPGIQTPEQISNTAGATASGTFGYDM-TDKHTAAEYLAGVSDFVDADTILGGVQITLTGTVANAQNPNITNAVVTLASESLTSASFNFSFHYDADPITAGVQDATAGGTLVFDKVADTYTFTLNDVIDGFSFDVLHTAELLSKEPTSNTGHPEIVVERLAAD-DPTTAV
Mus-11  TITAAFDADPGTPGIQTPEHLANTAGATASGTFGYDM-TDRHTAAEYAAGISDFVDSNGALAGVQIGLTGTVDNAQNPNITQAVATLTAETETSATFNFSFHYDKDPITAGVQDATAGGTLVFDKVADTYTFTLTDVIDGFSFDVLHTSELVAKAPTGNTGHPLIVAEQLTPNGDP----
Mus-14  TITAPFDADPVAPGIQTPEHLANTLNATASGTFGYDM-TDKHTAAEYAAGASDFVDTDAVTLGIQIGLTGTVDNAQNPAITQAVATRTAETETSATFNFSFHYDKDPITAGVQDATAGGTLVFNKEDDTYTFTLSDVIEGFSFDVLHTSELVAKAPTGNTGHPLIVAEQLTQDDDP----
Mus-2   TITAQFDADPATPGIQTPEHLGNAVGQTATGTFGYDIGHDSHPATFYDATHSDFVDTNPAVPGIQIGLTGTVDNTQNPNITQAVATLTSESATSATFNFSFHYDKDPLTAGVQDSTAGGTLVFDKVAGTYTFTLTDVIDGFSFDVLHTSELVSKQPTGNTGHPLIVAEQLTPNGDP----
Mus-1   TITAPFDADPSLAGVQSPEQLGNAVGQTASGIFGYDIGSDAHLAAFYAGGGSDFVDTNGALAGVQINLTGTVDNAQNPNITNAVATLASESLASASFDFSFHYDKDPITAGVQDATAGGTLVFDKAADTYTFTLNDVIDGFSFNVLHTNELIAKAPAGNTGHPEIVAEQLTPNSDP----
Mus-18  TLTVPFDADPVAPGNQTPEELGNAVGATASGAFGYDM-TDRRTAAEYAAGGSDFVDTNASLNGVQIDLTGTVDNAQNPGITNAVATLTAETTTSASFDFSFHYDKDPITSGVQDATAGGTLVFDKAADTYTITLNDVIDGFSFNVLHTSELVAKQPTGNTGHPLIVAEQLTPNADP----

Mus-3   -NPFFVQFTANSVTQQIGFGFNATGDGAPVG---DTTFNNAGAIHDMVTNVNEDWVSATQATNGVAGDTIQKGEVLTLRFFKENILSDVDPNAPGGGTERLDPTTSASGVVIKFDGIGNSEDLVVILDLKNANGNEITRAVNVENADLIKGNANIPFPYNTEFTLDNNDALLIIEQNDYT
Mus-15  DEDFYVQFTANSITQKIGFGFNSTGDGAPVG---DTTFNNGGATHDMVTNTHEDWVSATQATNGVAGDTIQQGELLTLRFFNSNV---------GIANEATDPTAKAAGVAIKFDGIGSSEDLILILDLKDANGNEITRAITVDNADIFK-TGQVPFPYNTEFSLDNNDGLVVIEANDYN
Mus-11  -DPFFVQFTANSTTQRIGFGFNSTGDGAPSGTPADTAFTQGA--HEMVTNVNEDWVSATQATNGVAGDTIQKGELLSLRFFAENILPDVNPGASGGGTERLDPTTKAGGIVIKFDGIGNGEDLVVILDLKDANGNEITRAVNVQNSDLIKGNANVPAPYNTEFTLDNNDALLIIEQNDYT
Mus-14  -NPFFVQFTANTTTRQIGFGFNSTGDGAPAGPPTDTSFTQGA--HDMVTNVNEDWVSATQATNGVAGDTIQKGELLTLRFFQEDILPDVNPGAPGGGTENVDPTAKASGIVIKFDGIGNSEDLVLILDLKDANGNSITRAVNVQNSDLIKGNANVPPPYNTEFTLDNNDALLIIEQNDYT
Mus-2   -DPFFVQFTGNSVTQQIGFGFNSTGDGAPSGPPTDVSFTQGA-HELVTNVNEDWVSATQSTNGVAGDTIQKGELLTMRFFKENILSDVDPSAPGGGTERLDPTTSASGVVIKFDGIGSSEDLILILDLKNAAGNEITRAVNVQNSDLIKGNANIPAPYNTEFTLDNNDALLIIEQNDYTV
Mus-1   -NPFFVQFTANSTTNSIGFGFNSTGDGAPNGPPTDTAFTQGA--HDMVTNVNEDWVSATQATNGVAGDTIQKGEVLTLRFFSENILGDVAPNAPGGGTERLDPTTSASGVVIKFDGVGNSEDLVLILDLKDANGNEVTRAVNVQNSDLIKGNANVPFPYNTEFTLDNNDALLILEQNDYT
Mus-18  -NPFFVQFTANSTTQQIGFGFNATGDGAPNGPPTDTAFTQGA-HDLVTNVNEDWVSATQSTNGVAGDTIQKGELLTLRFFQENILTDVNPGAPGGGNERLDPTAKASGVVVKFDGIGNSEDLIVILDLKDANGNEITRAVNVQNADLIKGNANIPLPYNSEFTLDNNDALLIIEQNDYTA

Mus-3   VAGETYQIQGIQIMQSSNGLTGTAINLNGAIGANGFSNATSNLTAWDPAD------QDVLKIVDIGFVQQTSGTIDANLDFAFKVADADGDATLLQHIPVGVLNA---
Mus-15  LSGETYQIQGIQIMQSANGLTGSGINLNGAVGANGGSVQNGAFTLQSFTDASFGNEQDVLKIVDIGFVQNTSGTIDANLDFSFQIVDGDGDTTPIQDLLLHV------
Mus-11  VAGETYQIQGVQIMQSANGLTGDAINLIGATGTNGGSNATSNLTAFDTTD------NDVLKIVDIGFVQSTSGTIPADLDFAFQVADADADQTATQHILVDVS-----
Mus-14  VAGETYQIQGIQIMQSANGLTGNGINLNGATGAANGSNATSNLTAFDTTD------NDVLKIVDIGFVQNTSGTISADLDFAFQVADADGDQTDTQHILVDVA-----
Mus-2   AGETYQIQGIQIMQSANGLTGNAIDLNGATGANGGSSATSNLTAWDPTD------NDVLKITDIGFVQQTSGTINANLDFSLALVDADGDATATQHINVNISNAFIV
Mus-1   VAGETFQIQGVQIMQSANGLTGDAINLNGATGANGGSSATSNLTAWDPTD------NDVLKIVDIGFVQQTSGTIDANLDFSFALVDADGDTTATQHLLVNVSNGFIV
Mus-18  AGETYQIQGIQIMQSANGLTGNAINLNGAVGTNGGSNATGSLTAWDPTD------NDVLKIVDIGFVQQTSGTIDANLDFAFNIADADLDLLGVQHINVQISEMFI-
```

1

B) group II-proteins

N regions

```
Nit-1   --------------------MQNLTATPAP---AGDADDND--------ILVSSLPAAFSTRLTALGAGTPTVGAALSGYTGAVGNTGSNIVSITPAPGGTITDLGLVGPGGAPLNGLD-
Nit-4   -MNEIITTTGVTITLDESAGLQNLTATPAP---GGDANDND--------IANSALPSVFSARLTALGLGSPTIGTALSGYTGAAGNTGSNIISVTPEPGGTITDLGFVGPGGAPLNGLD-
Nit-6   -MALNIATTGETITLDETAGLQNATATPAS---AGDANDND--------IANTALPTAFSARLTALGLGAPTIGTALSGYTGAAGNTGSNIISVTPAPGGDVTDLGLVGPGGAPLNGLD-
Pse-28  ---MAIEITGEDVVLDETPGLQNATATPTP---AGDADDND--------ILVASLPSDFSTRLTALSAGTATG-AALSGYTGDVGDTGSDAFTVTD--GATITDISFVDSAGAPLNGVD-
Pse-16  ---MAIEITGEDVVLDETPGLQNATATPTP---AGDADDND--------ILVASLPSDFSTRLTALSAGTATG-AALSGYTGAVGNTGSDAFTVTN--GESITDISFVDSTGAPLDGAD-

Hal-2   -----------------------------------------------------------------------------------------------------------------MDGVDS-
Hal-1   MSESLVNITGTDVTIDETAGLQNSGSTPPG------DADDDDVLFSTIPELFSLRLTALGATDPALGAAESDGDVVTFSP-------------------TGELGNVALTDADGALLDGDD-
Rhi-1   -MAIAITSAGAFVVLDETEDLQNATATPSP---AGDADDNDTSNPLPAAFSTALSSHGVVTFAEAALSGHNGAAGNTGANIITVTGATATTDFAFRGENGAAFTAYEAGATSTLNSGLS-
```

```
Rhi-9  -MAIAITSTGAFVVLDETEDLQNATATPSP---AGDADDNDTSTPLPAAFSTALTSHGVVTIAEAALSGHNGAAGNTGANIITVTGATATTDFAFRGENGAAFTAYEAGATSTLNSGLS-
Rhi-8  -MAIVITSTGVFAVLDETDGLQNATATPAPPSPSGDADDNDILSSSLPGPFSTRLTSYSLTVDEAALSGYTGAAGNTGANIINITGATATTDLALTGANGAAFPDYETGATSAFSSGLF-
Rhi-4  -MAITITPTGAFVVLDETDGLQNEAATPTP---AGDADDNDISSGSLPTAFSGRLSFYTLAVDEAALSGYN---GTTSSNIITVTGATASSDFALTGEDGVAFTAYVSGATSTFNSGLS-
Rhi-5  -MAIIITPTGESVVLDETDGLQNLTATPSP---AGDADDNDILSSSLPTALSSRLTFYGLTLDESALSGYSGAAADTGDNILNVTGATATSDFSLTGSGGAAFPDYEAGATSTFSSGLS-
Rhi-11 -MAITITPTGELVVLDETDGLQNATATASP---AGDADDNDFGG-VVPTALSSRLTFYGVAPDEAALSGYSGAAGDTGDNILNVTGATLDSDFALTGEGGAAFPDYGAGTSSFSSGLFA-
Rhi-10 -MAITITATDELVILDETDGLQNATATPSP---AGDADDNDILSSSLPVAFSGRLTFYGLTLDEAALSGYSGAVADTGDNILNVTGATASTDFALTASGGAGFPDYETGATSTFSSGLS-
Rhi-39 --MAVTINVVGTTSIDETPDLQKDDISLAS--------------------FQTNHASALAAINAALTADNLTVADDAIEIAGDNSVDITLTNATSPVQSLGFVTLDSNGQNPAPVDGLD-
Rhi-37 -------------------------------------------------------------------------MADAIEIAGDDTVDITLTNATSPVQGLGFVALDSNGQNAAPVDGLD-
Rhi-38 --MAVTINVLGTTSIDETPGLQQDDITLLS--------------------FQTNHASALAAINAALASDSLTVDADAIQIAGDEDVDITLTNATSPVQGLGFVTLDSNGQNPAPVDGLD-
Rhi-31 ---MAIDITAHDIIIDESAGLTDNDIDPS-----------------------------DPLYSTNTTLQYLLNLGTALEVAFKEDFVVASADPGETITSV-VLAQDSSGTAFSPTDGAV-
Rhi-40 ---MSLVIVTQDIIIDETASLQDDDINPT-----------------------------IAPYNANTTLQYLETLGSPIETAFQANFVQATASAGETIDSI-TLTQDAAGHPFSTTVGVN-

Var-1  -MAALNLTAGSTVTLDESANLQNLAATPAA---AGDSNDND-------------ISASSLPTAFSTRLTAQGVGTAINAALSGYNGSNTGTNAFTFSVTGTVTDMSFTDANGAPLNGLD-
Var-3  -MSVLITTAISTVTLDESAGLQNPVATPAP---AEDANDND-------------ILASSLPTVFSSRLTTLGAGAPTKAALSGYTGSNTGANAFTFTASGTVTDVSFTGATGAPLNGLD-
Var-4  -MPALNMTAGSTVTLDESATLQNLAATPAV---AGDSNDND-------------ISASSLPTAFSSRLTAQGVGTAINAALSGYDGTNTGTNAFTFSVSGTVTDVSFTDANGALLNGFD-
Pse-36 ----MAIILNSSITVDETSGLQNAATSTDIED-------INDNDITVAAINATDDTGDLPFPFESRLFALLGATPVKNAALSGYTGAAGNTGADLITITGTYVDLAFTDAQGKALGDPGN


Nit-1  -------SGLDTLDGVNILLFTDTNN-NIVLGRAGAADGPIVFALYLEEIGTPVS----------GAKMWSVQYAPLKNTDP--------TNPDDSLNLLDKVFVGASQDLTFSLANA--
Nit-4  -------SDLNTLTGTNILLYTDTNN-NIVLGRAGAADGPIVFALYLEETGTPVS----------GAKIWSVQYAPLENIDP--------TNPDDSLNLLDKVFVAASQDLEFSLANA--
Nit-6  -------SGLDTLSGANILLYTDTNN-NIVLGREEGTN-AIVFALYLEETGTPVT----------GAKMWTVQYAPLSNTDP--------ANHDDSLNLLDKVFVAASQDLEFSLANA--
Pse-28 -------SGLDTLDGTSILLYTDTNN-NILLGRAGSATGAIVFAAYIEETGSPVT----------GGKIWTVEYQPLKHPDG--------TNPDDSLNLLDKVFVGTSQDLEFSLANA--
Pse-16 -------SGLFTLDGTSILLYTDTNN-NILLGRAGSATGAIVFAAYIEETGSPVS----------GGKIWTVEYQPLQHPDG--------TNPDDSLNLLDKVFVGTSQDLEFSLAGV--

Hal-2  -------GFDRTSTGNDILLYTDTDNDNIVLGRDEMTD--EIVFAIYLTEPAG---------DPLESSIWVVEYDAIEHDGADQLNDGDGNDHDASYDLANLVYVTAFESQEFSFAGA--
Hal-1  -------SGLKTTDGESILLYTDTDNDNIVLG-KTAGG--TLVFAIYLEETGGTAT-----TGPTGGKLWTVQYEAIYHDGGDTNLDGDGNNHDSAVDLTGLVHVTAFEAQTFSFENA--
Rhi-1  -------AVAPDGTITEIYLFADPDDNNIVYGVAGDSGDDPIVFAIYLEEVKNASN------ITIGAKMWTVLADGYTLAHT--TDD-----HDESLDLADKLFVAAVAENDFSFANA--
Rhi-9  -------AVAPDGTITEIYLFADPDDNNIVYGVAGDSGDDPIVFAIYLEEVKNASN------ITIGAKMWTVLAEGYTLAHT--TDD-----HDESLDLADKLFVAAVAENDFSFANA--
Rhi-8  -------AVADDGTISEIFLFTDPTNNNIVYGVAGDSGDDPIVFAVYLEEVKTSG-------ITTGAKMWSVLADGYTLAHT--TDGSSVAAHDDFLDLTNKLFVSAIAENDFSFANA--
Rhi-4  -------AVADDGTITEIFLFTDPTNNNIVYGVAGDSGDDPIAFAIYLEEVKTSG-------ITTGAKMWTVIADGYTIAHD--VDGSTVADHDDFLDLTDKLFVSAIGENNFSFANA--
Rhi-5  -------ALDDAGNITEIFLFTDPTNNNIVYGVAGDSGDDPIVFAIYLDEVKTSG-------ITTGTKLWTALADGYTLAHN--TDGTTVADHDDSLDLTNKLFVAAVAENDFSFANA--
Rhi-11 -------LDDATGTTTEIFLFTDPTNNNIVYGVAGDGGTDPIVFAVYLDEVKTSG-------ITTGTKLWTALADGYTLAHN--TDGTTVTDHDDSLDLTNKLFVAAVGEDSFSFEKA--
Rhi-10 -------AVAGDGTITEIFLFTDPTNNNIVYGVAGDSGDDPIVFAVYLDEIKTSG-------ITTGAKLWTVLADGYTLAHN--TDGSTVADHDDSLDLTNKLFVAAVAENDFPFAGA--
Rhi-39 -------SGQQTLDGDGIFLYTDPDNDNVLLGRAGSGTDADPDGQIVFAVYLEEVTDQ--NSVITGGQLWTVLFEPLAHPDD--------QDPDDLVALPDTLGVAATGEQNFSFAGA--
Rhi-37 -------SGQQTLDGNEIFLYTDPNNDNVLLGREGSGTTADSGGQIVFAVYLEEVTNQ--NNVITGGQLWTVLFEPLAHPDD--------TNSDDVVTLPDALGVAATGEQSFSFAGA--
Rhi-38 -------SGQRTLDGNEIFLYTDPDNDNVLLGREGSGTTADAGGQIVFAVYLEEVTDQ--NNVITGGQLWTVLFEPLAHPTN--------PNPDDIVTLPNTLGAAATGEQNFSFAGA--
Rhi-31 -------TGIRTVDGNYVWLFQDPNNADVVIGVIGTSDPTSVGEPDPLLDPLAFSFAL--DG---DADLYLVQYVALMNPDPD-------NADVDLIDLDQLVYASVTGTSVLNFSSLGD
Rhi-40 -------SGIQTVDGNYVWLFRDPTHANVVIGVIGTSNPN--AAPSDASGPLAFSFGL--SGSGASADLYLVQYVPLFNPDP--------NNPDDRIDLTGKVFASVSGSTVVNFSQLGD

Var-1  -------SGLNTADGEDIFLYTDATNNNVVYG---KTALNVVVFAAYLEETGSPVN---------GAKIWTVQYEAMSNPDA--------ANADDPIDLTDKAFVSVSQDTEFNLADA--
Var-3  -------SGLNTADGEDIFLFTDTTNNNIVLG---KTSAGVIVFAAYLEETGTPVS---------GAKIWMVQYEAISNPDP--------TNPDDAVNLLNKVFVSVSQDTEFNLEGA--
Var-4  -------SGLDTADGEDIFLYTDTTNNNIVYG---KTALNVVVFAAYLEETGSPVT---------GAKLWTVQYEAISNPDA--------SNPDDAVNLADKLFVSVASASEFSFANV--
Pse-36 ANGGSDWSGLYTLDGTKIFLYSDTNN-NIVLGRAGSEGATPAPDDDLANPNGTIIFAAYLEQTATGAKVWMVQQEQLMHPDT--------ANPDDVVDMTNHLWVTASQDLAFDFAGL—


Nit-1  --PSGQNLFLMFTTANPTTVNDGGVIRITDPAIIATGKDPANQSTGVN--------INTGDTINSSQGGGTTTFGTNSQMITEQ------EGIRFSFVTGARQNVTIPN-----------
Nit-4  --PSGQNLFLMFTTANPTTVDDNGVTRITDPAIIATGKDPANQSTGAN--------ITTGDTVNSSQGGGTTTFGTNSQMIVEG------DGIHFSFVTGARQNVTIPN-----------
Nit-6  --PSGQNLFLMFTTANPTVVNG----RITDPAIIVTGRDPLNQSQGGN--------ITSGDTINSSQGGGTTTFGTNNQMIVEG------EGLRFSFVSGAQQDVTIPN-----------
Pse-28 --PSGQNLFLMFTTADPTVVDVGGVLRITDPTIIATGKDPANQSTGVN--------ITTGDTINTSQAAGPTTFGTNSQMITEQ------EGIRYSFVTGARQDLTIPN-----------
Pse-16 --PSGANLFLMFTKANPTVVDVGGVLRITDPTIIATGKDPADESSGAN--------INTGDTIITSQAANPTTFGTNSQMITEQ------DGIRFSFVTGARQDVTIPN-----------

Hal-2  --PPGSNLFMWFGDL--------------DQSIIVTGRDPANQSEGENI-------SS-GDTVNTS-GNTDTSLGTNGQQVKAG------EGLYFTFASGSTSSTPDDYIVPN-----LS
Hal-1  --PSGSNLFMAFGDA--------------SQALVITGENPADESSGENV-------SS-GSTVNSSQGGGTTTLGIDGQQIKAQ------QAIVVTFVTGAEADYLAGPAAGNDPGQPLS
Rhi-1  --PSGQNLFMMFGNT--------------SLAILVTGEDPADQSAGQNV-------SNDGDTVNTGQGGGATTLGTEGQQIKAQ------KALVVTFVTGANSNFLVPG---------LT
Rhi-9  --PSGQNLFMMFGNT--------------SLAILVTGEDPADQSAGQNV-------SNDGDTVNTGQGGGATTLGTEGQQIKAQ------KALVVTFVTGANSNFLVPD---------LA
Rhi-8  --PSGQNLFMMFGNT--------------TLAILVTGEDPANQSAGQNV-------SNDGDTVNTGQGGGATTLGTEGQQIKAQ------KALVVTFVTGANSNFLVPG---------LT
Rhi-4  --PSGQNLFMMFGDT--------------SLAILITGEDPANQSAGQHV-------SNDGDTVSSSQGGGPTTLGTEGQHIKAQ------KALVVTFVTGANADYIAGPNAGNNPGQPLS
Rhi-5  --PSGQNLFMMFGTT--------------SLAILVTGEDPANESAGQQV-------SNDGDTVNTGQGGGATTLGTEGQQIKAQ------KALVVTFVTGANSQFLVPD---------LS
Rhi-11 --PSGQNLFMMFGNT--------------SLAILVTGEDPANESAGQQV-------SNDGDTVNTGQGGGATTLGTEGQQIKAQ------KAMVVTFVTGADSQFLVPD---------LT
Rhi-10 --PSGQNLFMMFGTT--------------SLAILVTGEDPANESAGQQV-------SNDGDTVNTGQGGGATTLGTEGQQIKAQ------KALVVTFVAGANSEFLVPG---------LV
Rhi-39 --PAGNNLFMAFGNQ--------------SNALLVTG-------------------TSSSHTVNSSKGGGATTLGTDAQDVRAG------KGMYFTYVTGMDPSFLAPN-----------
Rhi-37 --PAGNNLFMAFGSS--------------ADALLVTG-------------------TSINHTVNSSKGGGATTLGTDAQDVRAG------KGMYFTYVTGMDPNFLAPN-----------
Rhi-38 --PSGNNLFMAFGNA--------------SNALLVSG-------------------TSISHTVNSSKGGGATTLGTDAQDVRAG------TGLYFTYVTGMNADFLAPN-----------
Rhi-31 APPGHNKWYILDADT--------------ASTQKILV-------------------TAHDNGVQAEVNVSTQGLGVSSQDVRFG------RELQIDLINGGTQSAGKNF-----------
Rhi-40 APPGHNQWYILDADT--------------ASSEKILV-------------------TAHDNGVQANVNVSTQGLGVSSQDVRFG------RELQIDLINGGTQSAGKNF-----------

Var-1  --PSGQNLFLMFTTGSPTIVDG----RISNVSIIATGKDPANQSGANPNSTADDINISTGDTINTSKAGGPTTFGTNSQMITEQ------EGIRFTFVTGARADVTIPN-----------
Var-3  --PSGQNLFLMFTKDNPTIVDG----RISEVTIIATGKDPANQSGPNPNITSDDININTGDTINTSQAGGPTTFGTNSQMITEQ------EGIRFTFVTGARADVTIPN-----------
Var-4  --PSGQNLFAMFGN--------------ATAAIVVTGKNPANESTG--------VNINTGDTVNTSLGGGLTTIGTNNQMIDPPSAKNPGEGMYFTFVTGANADDTVPN-----------
Pse-36 --PSGQNGFLMFKAGG------------STTGLIVTGMDPDQVP------------ANQADTVNTSQGAGPTTIGTNSQGIKAG------EGMIFSFITNPDARYTVPD-----------


Nit-1  --LDQNEADVEANIDFTNVFNARAATFDIVQLQSGKSAVVKVSAFSTAAEPGVNFIDGYGNDSSVAITHVKVTLN-GTVLIDTGVNATAN-GVTVAFNAG---VAT
Nit-4  --LDQNEADVEANIDFTDVFDARAATFDVVQLQSGKSAVVKVSALSTDSEPGVDFIDGYGDDNSVPITHVKVALQNGTVLIDTSTGGTAN-GVTVAFNTG---VAT
Nit-6  --LDQNEADVEANIDFTSMFNARAATFDVVQLQSGKSAVVKVTALSTAAEPGVNFIDGYGNDSTIPITHVKVSQG-GTVLIDTSTGGTAN-GVTVAFNAG---VAT
```

```
Pse-28 --LDQNEADVEANIDFTDVVNANKASFDVVQLQSGKTAVVKISAFSTAVESGTTFIDGYANDSPVEITSVSVINISTGLVIESSDGPEDDPAIAITFDADG--VAT
Pse-16 --LSPTEADVESNIDFTDVVNAKKASFDVVQLQSGKSAVVKISAFSTAAEPDVNFINGYANDTPVAITNVRVFNSAG-VVIENSDGSVNDPTIGISFSGG---VAT

Hal-2  QTEADVEANITPLALTDARG-ASVTVSQVNPG-GNTTVSMRITAYTTDLETGVNFLDG-------------LGDADDVPVLITGVTINGTS-VSFTTDGD---GVI
Hal-1  PTEALDEANIQFSGYVLAQA-AQFTISQMTPGNTNTTTSLEINAYLTADGSGDHYIDGNPL---VQGDSAVNITTDSVEVLRGDVNVVGTLGVTVNYTED---GVV
Rhi-1  PTEANLESNIAFSGYAQDVTGAAFTISQMTPGNANTTATVELQAFLTADASGTGYIDNN-----PLTTGDAAVAIASVQVIRGGVDVTGTLGVNVTISGG---IAT
Rhi-9  PTEANLESNIAFTGYAQDVTGAAFTISQMTPGNANTTATVELQAFLTADASGTGYIDNN-----PLTTGDAAVAIASVQVIRGGVDVTGTLGVNVTISGG---IAT
Rhi-8  PTEANLESNIAFSGYAQDVTGAAITISQMTPGSASTTASVELQAFLTADTSGTGYIDNS-----PLTTGDAAVAIASVQVIRGGVDVTGTLGVNVTISGG---IAT
Rhi-4  PTEANVEANIAFSGYAQDVLGAGLTISQMNPGSSNTTATVELEAYLTADGTGTNYINNND----PLVTGDTQINIASVQVIRGGIDVTNTLTITLSGLG-----AT
Rhi-5  PGEATDETSIAFTGYAQDVLGGEITISQMTPGNTNTTATVEIEAYLTADGTGDNYIDNN-----PLVTGDAEVNIASVSVIRAGIDVTGTLGVTVTISGN---TAT
Rhi-11 PGEATDETSIAFTGYAEHVRGASITISQMTPGGSTTTATVELAAYFTTDAAGDSYINDD-----PLVTGDTEINITDVNVIRAGVDVTGTLSVNYDGN-----TAV
Rhi-10 PSEATDEASIDFTGYAQGVRGAEITISQMTPGSASTTATVEIQAYLTADGTGNNYIDDD-----PLVTGDTKVNIESVHVIRGGIDVTDTLTVTISAN-----TAT
Rhi-39 --LSHQEATTINDIDFTGVQDSDAASLTIVKLTGGTSVSVKLTAFTTDAEPKGDYFG---------GLTDDTAVDITHVYINGTE-----VSFTTSGD-----GVI
Rhi-37 --LSHQEATTVGDIAFTGLQDSDAASLTIVKLTGGTSVSVKLTAFTTDVESGANYFPGL-------GDADDDPVTITHVYINGTE-----VTFTTSGD-----GVI
Rhi-38 --LTHKEAINTNDIQFTGLQESDAASLTIVKLTGGTSVSVKLTAYTTDVEARANYFPGL-------GDSDDVNVTITHVYINGSE-----VPFTLDGDGT---GVI
Rhi-31 --TNTPTAPNYTDHIETVSTAGFSISQSTPTHT---VADIEVHAYNNDDNAKGATLPGD-------DNDAEVNIASVTFKLNGVVTDAATLGITVDYTNL---GVI
Rhi-40 --TNSPLAPDYDSHIETVTSAGFSVSQSTPTNS---KADIEIHAYNTADNAEGASFPTD-------NTETESNISGVTFKLNGVTRTAAALGITMDFGGN---GLI

Var-1  --QDQNEADLESNIDFTGMFGARTADFDVVQLQSGKSAQVKISAFSTAAESGNSFIDGYAGDATVAITSVRVLNSATGAVLETFSAGAEGGLSSSLAISITGGVAT
Var-3  --LDQNEADLESNIDFTGMFGARTADFDVVQLQSGKTAQVKVTAYSTAVESGNSFIDGYAGDTVVSITSVRVLDSVTGAVLESFSGGAEGGASSTIAISITGGVAM
Var-4  --LDQNEADEESNIDFTGLLGTTEASFTIAQLQPNKAATLKISAFTTVLASGDAYVDNLQNNTAINISSVVVRDSAG-------------QLVTGLSIDLSGDTAV
Pse-36 --LTHGEATNESNILFGGLFAASGATIIIAQATPPKTCAATISAFTTGLEVGSAYYDGLLDGNGHAVNGADNLGYIASVTVIGANGVAIAGLTGVTTNTTLGSISVTFTTSNAGLDRTAT


Nit-1  ISGVKAGEKIEYTTTADHNRVLIENGAALNATGDTHADFDIGGFKLLQVSTATAEVGSHVKFEDDGP
Nit-4  ISGIKAGYNIEYTTTADHNRVLVEN--AGSGRGNASADFDIGGFQLLQVSATTVEVGSKVNFEDDGP
Nit-6  ISGVKAGYNIEYTTTGDHSRVLVEN--AGSGRGNASADFDVGGFQLLQVSTATVEVGSKVSFEDDGP
Pse-28 ITGVKAGYRIEYTTTDEHNRVLIENGAALDARGNTHADFDIGGFKLLQTAVTTAEIGSQIVFEDDGP
Pse-16 VTGVLAGYQIEYTTTEDHNRVLVENGAALDARGNTHADFDIGGFTLLQTAVTTAEIGSKIIFEDDGP

Hal-2  VSGVKAGDVVGYTTVADHQRVLIENAQPESGKG-SNVSFDLGGFALTEVAGDTDEIGSKISFYDDGP
Hal-1  VMGVKNGDVVRYETDADHNRVLIRNDQPEKGSG-SNVAFDLGGFTLTEVAGDTDEIGSKIFFEDDG-
Rhi-1  ITGVKDDDIIQYTTTDDHNRVLIRNDQPASGQG-SNVSFDLGGFTITQVSSDVEEVGSKVRFEDDGP
Rhi-9  IMGVKDDDIIQYTTTGDHNRVLIRNDQPATGQG-SNVAFDLGGFTITQVSSDVEEVGSKVQFEDDGP
Rhi-8  IMGVKDDDIIQYTTTGDHNRVLIRN-QPATGQG-SNVAFDLGGFTITQVSSDVEEVGSKVRFEDDGP
Rhi-4  ITGVKDNDVIQYTTDSAHNRVLIRNDQPSG----SNVSFDLGGFKITQVASDVEEIGSKVRFEDDGP
Rhi-5  ITGVKDDDVIQYTTDDDHNRVLVRNDQPAQGAG-SNVSFDLGGFTINQVSSDVEEVGSKVKFEDDGP
Rhi-11 ITGVKDDDIIEYTTDADHNRVLVRNDQPEKGSG-SNVSFDLGGFTITQVSSDVEEVGSKVKFEDDGP
Rhi-10 ITGVKDDDVIQYTTTDDHNRVLVRNDQPTQGPG-SNVAFDLGGVTITQVSSDVEEVGSKVKFEDDGP
Rhi-39 VSGVHDGDVIKFETDGDHNRVLVQNAEPAG----SNIHFSIGGFSVDNAVTAAVPVGDHLEFYDDGP
Rhi-37 VSGVHDGDVIKFETSGDHNRVLVENAEAAG----SNIHFSIGGFSIDNAVTAAVPVGDHLEFYDDGP
Rhi-38 VAGVHDGDVIKFETDGDHNRVLVENAQPLG----SNIHFSIGGFAVDNAVTAAVPVGAHLEFFDDGP
Rhi-31 LHNVGEGVTVDFTADGTFDRFTIKNIDAQK----DYFDVKEVHFGTDQTNVASAEVGSLINFDDDGP
Rhi-40 LHNVGTGVTVDFTADATFDRFTIKNVDPQK----DYFDVKEVHFGGKSTNAASAQVGSFINFDDDGP

Var-1  ITGVVAGQSIEYTTSADHNRVLIENGAALNASGNTHADFDIGGFRLLRSSTATAQIGSQMIFEDDGP
Var-3  ITGVKAGYSIEYTTSADHNRVLVENGATLDATGNTHADFDIGGFRLLQVSAATAEVGSMMFFEDDGP
Var-4  ISGIKAGYVIEYQTDANHNRVLIENVGSATNN-NLNASFDIGGFSLPKFEQATGEVGSMMVFEDDGP
Pse-36 IVGFEANFSLQYTVDGTHNRVVIENSGTGN------STFDIGGFHLPNVVAVPQEVGSQLNFEDDGP
```

C regions

```
Nit-1  TLDFGN-LVGTGTIAPQYGEWNGSVGAD--QPGNLDISLN-QFQLVRPDGTTIDGSSFTFDELAGSPNAS-GDFLFAGSLTADFDNIAGTADTTVDFTLTALHDGR-------
Nit-4  TLDFGN-LVGTGTIAPQYGEWNGSVGAD--QPGNLDISLS-QFQLVRPDGSTIAGSSFTFNELAGSPNPA-GDFLFAGSLTADFDNNAGTADTTVDFTLTALQNGR-------
Nit-6  TLDFGN-LVGTGTIAPQYGEWSRLVGAD--QPGNLDISLN-QFQLVRPNGTTINGSSFTFNELAGSPDAS-GNFLFAGSLTADFDNIVSTPDTTVDFTLTALNDGR-------
Pse-28 ALAFGN-LIGTGSVLPQTGFWSNSAGADGLDAAGLDISVNSQFTLVRPNNTTTTG-TATLTEVVPSPDGN-GAYQFAGTLTGDFDNNADTADTTVDYTLTAFDDGS-------
Pse-16 ALAFGN-LIGTGSVLPQTGFWSNSAGADGLDAAGLDISVNSQFTLVRPNNTTTTG-TATLTEVVPSPDGN-GAYQFAGTLTGDFDNNADTADTTVDYTLTAFDDGS-------

Hal-2  AVTVND-ASGTYQAG--ADGTWSDLPGADGFDS-----LNVTFDSFEIDANGTQTTS-TT-N--STFART-GNLSWAGSVTGDFTDDGVINEQTVDFSLTFDPVAD------T
Hal-1  PVSNAS-GTYATPAE--ATGDFVDGSGTDGFGD-----LAITFNWYQIDGEGLIQTS-GN-PNVTFVESV-TDTTWAGTIFDDFNGDGVS--ETVAFTLDVDLDTN------E
Rhi-1  AVTVAD-SNGNYDDG-AQGTWDDNNPGSDGFAS-----LSVNFDSYEID-----DDG-LI-TVDTALTKT-GNFTFTGSITDDFNGDGTD--ETVGFTLTFDPVN------DT
Rhi-9  AITVDN-SSGTYDLG-AQGIWSDD-PGSDRFDS-----LEVTFDGYSIGGSAVTVDT-SL-GTKNETDVN-GNYVFTGSITDDFNGDGAD--ETVGFTLTLDPNDPDDPDDNT
Rhi-8  AVTVDN-SSGTYDAG-AQGTWNDNDPGSDGFES-----LSVNFDSYEIDG-----DG-LV-TVDAALTKT-GEFTFTGSITDDFNSDGAD--ETVGFTLTFDPVN------DT
Rhi-4  AVTVND-TSGTYAAG-AQGTWSDDDPGSDGFAS-----LSVTFDGYEIDANG---------AQPGSALTQ-TGLAFDGSITDDFNGDGID--ETVEFTLTFNRAAN------T
Rhi-5  TVTVND-ISGTFTDDPQTG-TWVHAPGEDDFKS-----LNVSFDSYEIDDN-------GP-VAGSALTKT-GDFTFTGSITDDFNGDGID--ETVPFALTFNPDTD------Q
Rhi-11 TVTVED-LDGTFTDAVQTGGIWDHTNSEDDLNS-----LNVNFDGYQIDAHP------AV-VVDAALTKT-GGFTFDGSITDDFNGDNVD--ETVPFTLIFNPDTD------A
Rhi-10 TVTVND-IFGTFTEDPQTG-TWDHAPGEDGFKVDGDGFLNVTFDSYEIDAN-------GP-VAGSALTKT-GDFTFTGSITDDFNGDGDD--ETVGFTLTFDPDTD------Q
Rhi-39 TIAVAD-VTDGDYTGGAHG-TWTNDPGTDGLGS-----LSVSFDSFEIDSHGTVTTT----ATNSSFTDN-LDGSFDGSITADFNGDG--QDDTVGFTLTLNSDD-------T
Rhi-37 AIAVAD-VADGDYTNGAHG-TWTNDPGQDGFGS-----LGVTFDSFQIDSHGVVTAD----ATNSTFTDN-LDGTFDGSITADFNGDG--KNDTVNFTLTLNSDD-------T
Rhi-38 VIAVAD-VAHGDYTNGAQG-TWTHDPGADGLGS-----LSASFDSFQIDSHGIVTTT----ATNSSFTDN-LDGSFDGSITADFNGDG--TNDTVEFTLTMNSDD-------T
Rhi-31 AIAVEN-ATG-DYDSGAHG-TWTHDPDADGFNL-----LGVTLDDYTIDSNPKVTS-----GLSLTPDAA-DPYTFTGSIHDDFNGDG--TAEDVDFTLTFDPVN------GT
Rhi-40 SITVDD-IASGTYAAGGSS-TWTDPPGADGFKS-----LNVTFNNYTLDAHSPVTVNSSL-GTLTQTDAN-GNYVFNGTIIDDFTNDNKVNPQTVAFKLTFDAVDPGS---GT

Var-1  PTIVARTDLIYANTSNPSPGGTGTFDYEIGADAR--SSYSVSNSDFSAITLTGTVGNAAITSPTVTWTSET-ATGASFAVSFSYKPDPGSATTTNATGTLSFDKVNGT
Var-3  -SIVARSDLIYSNTSNPSPGGTGAFDYEIGADAR--LSYSSSSSDFSAITLTGTVGSAAITSPTVTWSSET-STGASFAVSFSYKPDPSSANTTSATGTLSFDKVNGT
Pse-36 VVTAKS-NVVYANSSNPTPGGTGVFAYDIGADDRLGTVYSSANSDLSVSMGDSTVGTTAITNKVVTWASESDTSAVFNLSFTYVSNNPTLGALTNATGTMTFDKVADT
```

```
Var-4   -TVTAI-SDLTGANDGLPIAG---TYNFSVGADDVDNASTDGIVLNSLTGT-TGGGRAITDAVVSHFAED-ATTVTYNFSFNYYPGPTSTTTQAATGTVVFNKTDGT


Nit-1   YVFDLEQGFGSTITFSSAD GSLDAGGPDPVRTLTIPPD---------EQVVFFGVQATTSDS-------------DIVSAIGLGEPDLTEAQIEA G------GFSFLG
Nit-4   YVFDLIQGFGSTITFSSAN GSLDAGGPDAVRTLTIPPD---------EQVVFFGLQATTSDS-------------DIVSAIGLGEPDLTEAQIEA G------GFSFLG
Nit-6   YVFDLVQGFGSTVTFSSED GSLDAGGPDPVRTLTIPPD---------EQVVFFGVQATTSDS-------------DIVSAIGLGEPDLTEAQVEA G------GFAFLG
Pse-28  YALDLVQGFSSTVVLSTAD GSLAAGGPDPVRTLLIPDTNDPTIPSASEEVVFFSAKALASTT-------------DILAGIGLGAPDPTEAELQT TPL-----PSYID
Pse-16  YALDLVQGFSSTVVLSTAD GSLAAGGPDPVRTLLIPDTNDPTIPSASEEVVFFSAKALASTT-------------DILAGIGLGAPDPTEAELQT TPL-----PSYID
Hal-2   YSIVIDDLPTSTSTFDTSQ GTLKAGGPDEVRTLLFGGEP--PTDAGNDDIVFFGAVPTAVRS----DPAEPVDIDSILDLVVRGELDLTEEEIEA LLPL----PDLVN
Hal-1   YTVTLDTPPAQETTTSTAD GSLDAGGPDAVRTLLFGGD-----DAGSDDIVFFGAVATAPRSDVLGGNPYPAVPNDIEDLVVEGEPDLTEAQIEG FLTPTNQIPTLIN
Rhi-1   YDLDVTTPPPTTRTFDTSQ GSLKAGGPDAVQTLLFGGS-----EAGADDIVFFGVVATAPIQ----GVSSPP-TNDIEDLVVEGAGDLTEAQIEG LFPI----PSLIN
Rhi-9   YDLDVTTPPPTTRTFDTSQ GSLKAGGPDAVQTLLFGGS-----EAGADDIVFFGVVATAPIQ---GVSSPP--TNDIEDLVVEGAGDLTEAQIEG LFPI----PSLIN
Rhi-8   YDLDVTTPPPTTRTFDTSQ GSLKAGGPDAVQTLLFGGS-----EAGADDIVFFGVVATAPIE---GVSPPPPSANDIEDLVVVDPINGTDLTEAQ IEGLFPI-ASLIN
Rhi-4   YDLEVTTPPTTIITFDTSQ GSLKAGGPDAVQTLLFGGS-----EAGADDIVFFGVVPTAPFE---NGTPPP-ATNDIEDLVVVGATDLTEGQIEA LLPL----PGLIT
Rhi-5   YTFQMENPPTTIIQRDTTQ GALKAGGPDPVQTLLFGGS-----ETGADDVVFFGAVANAPQS----GVAGDPPPNDLLDLIGTGATDLTEDQIQA FLSPTNQIPTLIN
Rhi-11  YTFQMENPPTTIIQRDSTQ GALKAGGPDPVQTLLFGGS-----ETGADDVVFFGAVATAPQS----GVAGDPPPNDLLDLIVTGATDLNEDQIQA FLSPTNQIPTLIN
Rhi-10  YTFVMDNPPTQVIQRDTTQ GALKAGGPDPVQTLLFGGS-----DANADDVVFFGAVATAPAS----GVTGNTPPNDLLDLIVNGAPDLTEEQIQA FLTPTNQIPTLIN

Rhi-39  YDLTFTTPPTTTTFSTDQ GSLDAGGPDPVRTLTIGSED--VVFSAVKALTATGDIKAF-------------------------LNASEADIQT N--------AGYL
Rhi-37  YDLTVTTPPTTTTFSTDQ GSLDAGGPDPVRTLTIGSED--VVFSAVKATTATADIKAF-------------------------LNASEATIQG S--------ATYL
Rhi-38  YDLTVTTPPTTTTFSTDQ GSLDAGGPDPVRTLTIGSED--VVFSAVKATTSPDAIKAF-------------------------LDHTESDIQS H--------ATYL
Rhi-31  YDLDVDTPPGTITTFDTTQ GSLKAGGPDAVQTLQFPPAG--HDGDVIFFSNLVTAPLENGAG------------SFPNDIEDLVNNDPTEATLEG Y-----GATSLIN
Rhi-40  YNIQFITPPTSTTTLSTAN GTLGAGGPDPVQTLTIGNQK--IVFSAVKESALVGSIEAN-------------------------LDKTEAQIQT N-----PLPSYID

Var-1   YSLALNAPIAGFNTVSTGQALGFTGYTVGTSTVDQTQPDVSVAQLSSNFFVQFTGYSEPGGGTGANNIQAIAPAAPLGTANGTAFVNGELFKQA
Var-3   YSLALTAPIAGFSTVSTGQALGFTGYTQGTQTVDQTQPDVSVAQLSSNFFVQFTGYSEPGGGTGANNIQAIGPVDPLGTADGTAYVNGELFKQA
Pse-36  YTLSLADDIDSFSILKTSAAQGFTGYNIGGTTPINQQPPVSVAKLADDFFVQFSGFAEQGSGTGANNIMAGGNN---------AFVPGELF-SA

Var-4   FAFDMDQLIGGQTTFSTSSPLASFNYDT----EGNNSPEIVVQQYSSDFFGVLS----------ASSARPPSDSGDLMSGNDHAFTTGEIFTSE



Nit-1   SAN-MNVSTSGIGIGNNNLDGN---------STAGINAGDESFVINPE-TLLTGMKVFIDNSVQGYDPTT--EELYYKIFFDDGT  NSGNVKVLSGDLTAEA--
Nit-4   SAN-MNVSTSGIGIGNNNLDGN---------GTAGINAGDESFVINPE-TLLTGVKVFIDNSVGGYDPAT--EELYYKIFFDDGT  NSGSIKVLSGDLTSEA--
Nit-6   AAN-MNVSTSGIGIGNNNLDGD---------TTAGITAADESFIINPE-TLLTGMKVFIDNSVGGYDPAT--EELYYKIFFDDGS  NSGNIKVLSGDLTAEA--
Pse-28  PRS-MNVSTSGIGVANNVLQGD---------NLVAIGATDESFVVNPE-SLVTAMKVFIDNSVAGYNTAT--EDLYYRIYYEDGT  TSDLIEVN--TLTPEP--
Pse-16  PRA-MNVSTSGIGVANNVLQGD---------NLVAIGATDESFVVNPE-SLVTAMKVFIDNSVAGYNTAT--EDLYYRIYYEDGT  TSNLIEVN--TLTPEP--

Hal-2   PGTQMNVSTAGIGINNNNLDGADEGDGTGAFAGTTITSGDESFVVNPE-TLVDSVTVYISSTVQGYDTAT--EDLYYTVYYADGS  VSGPILVEELDLTHYANN
Hal-1   ASTQMNVSTSGIGINNNNLDGDGNDGGSGAFFGTSITSGDESFVVNPE-PLVDSVTVYISSTVQGYDPET--EDLYYTVYYADGT  IDSPQKVTEDDLTRYANN
Rhi-1   PSTQMNVSTSGIGINNNNLNGASEGAGTGAFAGTSITSGDESFVVNPE-TVVDKVTVFIDNSVGGYNPAT--EELYFTVYYTDGT  VQAATKVTAGMLT-----
Rhi-9   PSTQMNVSTSGIGINNNNLNGASEGNGTGAFAGTTITSGDESFVVNPE-TVVDKVTVFIDNSVGGYNPAT--EDLYFTVYYTDGT  VQAATKVAAGMLT-----
Rhi-8   PSTQMNVSTSGIGINNNNLNGASEGAGTGAFAGTTITSGDESFVVNPE-TVVDKVTVFIDNSVGGYNPAT--EDLYFTVYYTDGT  VQAATKVAAGMLT-----
Rhi-4   PGTQMNVSTSGIGVNNNNLNGADQGAGTGAFAGTTITSGDESFVVNPE-KVVDKVTVFIDNSVGSYNPAT--EDLYFTVYFTDGS  VQAPVKVTAGMLT-----
Rhi-5   SATKMNVSTSGIGINNNNLDGN---------GVLGIQAGDESFVFNPE-EVVDKVTVYIDNSVGGYSPPT--EQLYYTIYFTDGT  ISSNNLVSAADLHNAPRK
Rhi-11  ADTKMNVSTAGIGINNNNLDGN---------GTLGIQAGDESFVFNPE-EVVDKVRVYIDNSVGGYSPPA--EQLYYTIYYTDGT  ISSNNLVSAADLQNAPRK
Rhi-10  GGTQMNVSTSGIGVNNNNLDGT---------ATG-IQAGDESFVFNPE-EVVDSVTVYIDNSVGGYTPPT--EELYYTVYFTDGT  VSSNNLVTAAILQNAPRK
Rhi-39  SPNQMNVSTAGIGLADNLFEGNSIAGIDG--ATTSGGKVDESFVVDPEGTVSSMTVIINNQTNGGYTPINNTEQLFYRIYFSDGS  VSSPVKVEQGDLTVTS--
Rhi-37  SPNQMNVSTAGIGLGDNLFEGNSIAGIDG--ATTSGGKIDESFVVDPEGTVSSMTVIINNKTNGGYTPSN--EDLFYRIYYSDGT  VSADHKVVTGDLTVTS--
Rhi-38  SPAQMNVSTAGIGLGDNLFEGNSIAGIDG--ATTSGGKFDESFVIDPNGDVSSMTVIINNKTNGGYTPSN--EDLFYRIFYANGD  VSEPVKVLAGDLTVTS--
Rhi-31  SSSKMNVSTSGIGVNNNNLDGKSGGTPLF--AGTSITSGDESFVVNPQ-QDVDKVTVYIDNSVGGYNPAT--EDLYYIVYYTDGT  VSGPTDVTAGMLSNAPRH
Rhi-40  PAS-MNVSTSGIGVGNNNFDGNAVAGVDG--QTTSGGAYDESFVIDPTGFLVSSMKIYIDNSVGGYDPST--EGIFYRTYTRN--  --ADNSITAGSITKVLGG

Var-1   GT-WVSVSNLANGVAGDTIQQGEVLDLDFFAANPFGFTTATPTTQAAG-MFLKFDGIGSEDLVLVLKLVDPD----------DGSRTTKAIIIDNADIIKSGGTIPAPYSISL
Var-3   GT-WVSVSNLANGVAGDTIQKGEVLDLDFFAANPFGITTTPTTQAAG-MFLKFDGIGSEDLVVVLKLVDPD----------DNSRITKAIIIDNADIIKFGGTIPAPYSITL
Pse-36  ATSWVSVSGTAAGVAGDTIQQGEVMDLDFFTASPFGDTTAAPTATSKG-IYVKFDGIGSEDLILVLKLIDPDDNSLITRAIIVDSEDIIIKNASTSPNNFHPIANPAAYGITL

Var-4   STAFVNVATNTLGVNSDTVQAGELLNFDFYRSNPVSNPTSTSPPQQPGAAIVGTDKAYADAIDITIDQITDG----------EDVAILLKLFDASTNTTTTRLLIANSATDYQ

Nit-1   S--------GGQTSFAIDT--QGSKLIDSVQLTMGKGVIKIPVIEFIQATENLASDIKLDLTASLTDADSDTATSNFSVDLFANDLSGTFD
Nit-4   S--------GGQKSFAIDT--QGAKLIDSVQLTMGKGVIKVPVIEFIKSTENLASDVKLDFSASLTDADSDTATSNFSVDLFANDLGQTFD
Nit-6   A--------GGQVSFGIET--QGSKLIDSVQLTMGKGVVKIPVIEFIQATENLASDVLLDFTASLTDADGDSATSDFSVDLFANDLEGTFD
Pse-28  P--------RGQVSFLIEQ--EGSLLIDAVQLTMGRGDIKIPVIQFIQETESLASDIQLTFNATVTDRDGDSVSSTFDANLFANDLAGAFD
Pse-16  P--------RGQVSFLIEQ--EGSLLIDAVQLTMGRGDIKIPVIQFIQETESLASDIQLTFNATVTDRDGDSASSTFDANLFANDLAGAFD

Hal-2   PDTSVPKEAKGGSSFTIESGENQVNQIDAVQFTMGLGTVKIPIISFDVETEFEPEPLAMDFTATLEDGDNDTATDDFTVNLL
Hal-1   ADVNVPKEAKGGSSFTIED-EDGDRQIDAVQLTMGLGTIKIPVIEFNTLTQFDPESLELNFTATLEDGDEDTATDDFTVDFSPEPPAELSYVSLDDPDVLL
Rhi-1   P-PVTSGVAAGGFSFEIDG---GAKQIDAVQLTMGEGTIKIPVIAFSVEQVFDPEPLQLDFTATLFDGDGDSSSDSFSIDLIEAVV
Rhi-9   P-PVTSGVAAGGFSFEIDG---GAKQIDAVQLTMGQGTIKIPVIAFSVEQVFDPEALQLDFTATLFDGDGDSSSDSFSIDLVEAVV
Rhi-8   P-PVTSGVAAGGFSFEIDG---GAKQIDAVQLTMGQGTIKIPVIAFSVEQVFDPEALQLDFTATLFDGDGDSSSDTFSIDLVEAVV
Rhi-4   P-PVTSGVASGGFSFEIDG---GDKQIDAVQLTMGKGTIKVPVIAFSVEQAFEPEPLSMDFTATLFDGDGDSSSDAFTIDLAETVV
Rhi-5   SDPTVPDVAEGGKFFEIDG---GTKQIDAVQLTMGAGTVKIPVIQWTIEQEFNPQPLHLDFTASLFDGDDDSSQDPFAIDLVDATV
Rhi-11  ADPTVPDVAEGGKFFEIDG---GTKQIDAIQLTMGAGTVKIPVIQWTIEQEFNPEPLHLDFTASLFDGDGDSDEDAFAIDLADATV
```

```
Rhi-10 GDPNIPDVAEGGKFFVIDG---GTKQIDAVQLTMGAGTVKIPVIQWTIEEEFNPQPLSLDFTATLFDGDDDFDSDTFSIDLVDATV
Rhi-39 S-------KTASFTLGDPD---GPNDIDAVQLIMAKGTIKVPTITFTVSTEFSPQDLDLNFTAELFDGDQDSSPDPFTVHVDAA
Rhi-37 S-------STASFTVGDPN---GPNDIDAVQLIMGRGTIKVPTITFSVSTEFSPQPLDVTFTAELFDGDHDSQTDPFTVHLDAA
Rhi-38 S-------STASFTVGNPL---GSNDIDAVQLVMGRGTIKVPTVTFSVSTEFSPQPLDLHFTADLFDGDHDSITDPFAIHLDAATV
Rhi-31 SDPLIPDVAEGGKYFEIDG---GAKQIEAVQLIMGVGTVKIPVIQFSLEQESNPAPLDLDFTANLVDGDNDSFTDTFSVHVDAPDGII
Rhi-40 PDLTSEAGGQKSFVIPSVD---GKNDLDAVQLFMGSGTVKIPVIEFNISNTFNADPISMNFTATIADGDSDTKTDTFKIALA

Var-1  GDNNDGAVIIESNDFNTGTENYVIEGAQLLVSTEGVTGTAINLNALTGAGGNST--------TLQEFSGTPGTANPEAATSDNDVIKISDIGFVTLNSGQLDANLQFSLAVKDADGDATATQVLDVGIVGGTAFTGG
Var-3  GDNNDGAVIIESNDFNTGSENYVIEGAQLLVSTEGVTGTAINLNPVTGAGGGST--------TFQEFSGTPGTANPEAATTDNDVIKISDIGFVTLNSGQLDASLQFSLAVKDADGDATATQVLDVSIVGGTTFTGG
Pse-36 ADQNDGAVIVESNDYNFGNENYVIQGLQVLVSTEGVTGSGYNLNGNVGVSGASTGNTLTFSNESGEGSKSNPVEAGEAGTWDGDVVKITDLGFVTSSTPD--AHLTFNVVVTDADADATPAQTLDVTIEGDKTFTGG

Var-4  SSAGGGTKIVSIGEDDYNSAIYQIAGVQVLSSTEDLTGTGISLSTHDTVNLTSAGMNYADTADNDVFKIIKIDVITETTIN-SDVDLNFAGQLIDGDADYANFDFDVHLEIDGIANLIGTTNQPTAIA

Nit-1  FTLLGASSDLDGFNVDLAASEDAYRIEGFDTSA---DTLFLLGDLGAGVSINNTGADSIVSVTETGGAQTTVITVVGVDLALTDIALV
Nit-4  FTLVGASSDLDGFDVDLSASQVSYKIEGFDKPA---DKLFLLGDSGAGVSIDNSGANSIVSVTETTGGQTTVITVVGVDLASSDIVLI
Nit-6  FTLTGASGVLDSFNIDLSALQDSYKIEGFDTPD---DQLALLGDLGATVSIDNSGADSIVSVTETG-GQTTSITVVGVDLVPANIVIA
Pse-28 YTLVGTDDELDAFNVDLSFDENLYQITGFDTDANLRDTLVLNGDQSAVVQIDTSGADSIVTVTETG-GDVTTITLVGVDLLSSDIVLGSA
Pse-16 YTLVGTDDELDAFNVDLSFDENLYQITGFDTDANLRDTLVLNGDQSAVVQIDTSGADSIVTVTETG-GDVTTITLVGVDLLSSDIVLGSA

Var-1  ADNEAIQGSAGNDTLNGGGGNDTLTGGLGKDSLTGGDGNDIYDFNAVAESAAGANRDVVSGFMSGADTLDLSGIDAISGNANADDAFSYLGDAAFTNVAGQLRFDVATQTLQADIDGNGTADFEVQLVGVVAPPPLDDFFM
Var-3  ADDEAIQGSAGNDTLNGGAGNDTLTGGLGKDTLTGGDGNDVYDFNAVAESPAGANKDVISGFTSGADKIDLSGVDAIPGNG--DDAFSYLGDGAFTGVAGQLRFDVATNTLQADVDGNGVADFEVQLVGVVVPPPLGDIIV
Pse-36 VGADTFNFSGLDTDGSLTAATAVISAGFTSGVDTLNFPTAGTGANYTEVAAPAANVAAFITAADTALNGTTNYYFGVVGGDGYLAYDGDGTGITSIIKLVGVTDIAPTDIV
```

Figure S6. Organization of ddxp and associated T1SS genes in different bacteria. Genes within T1SS clusters are similarly highlighted. Treg, transcriptional regulators. Gene orientation is denoted by arrows. Different hlyb-hlyd gene pairs are associated to Mus genes.

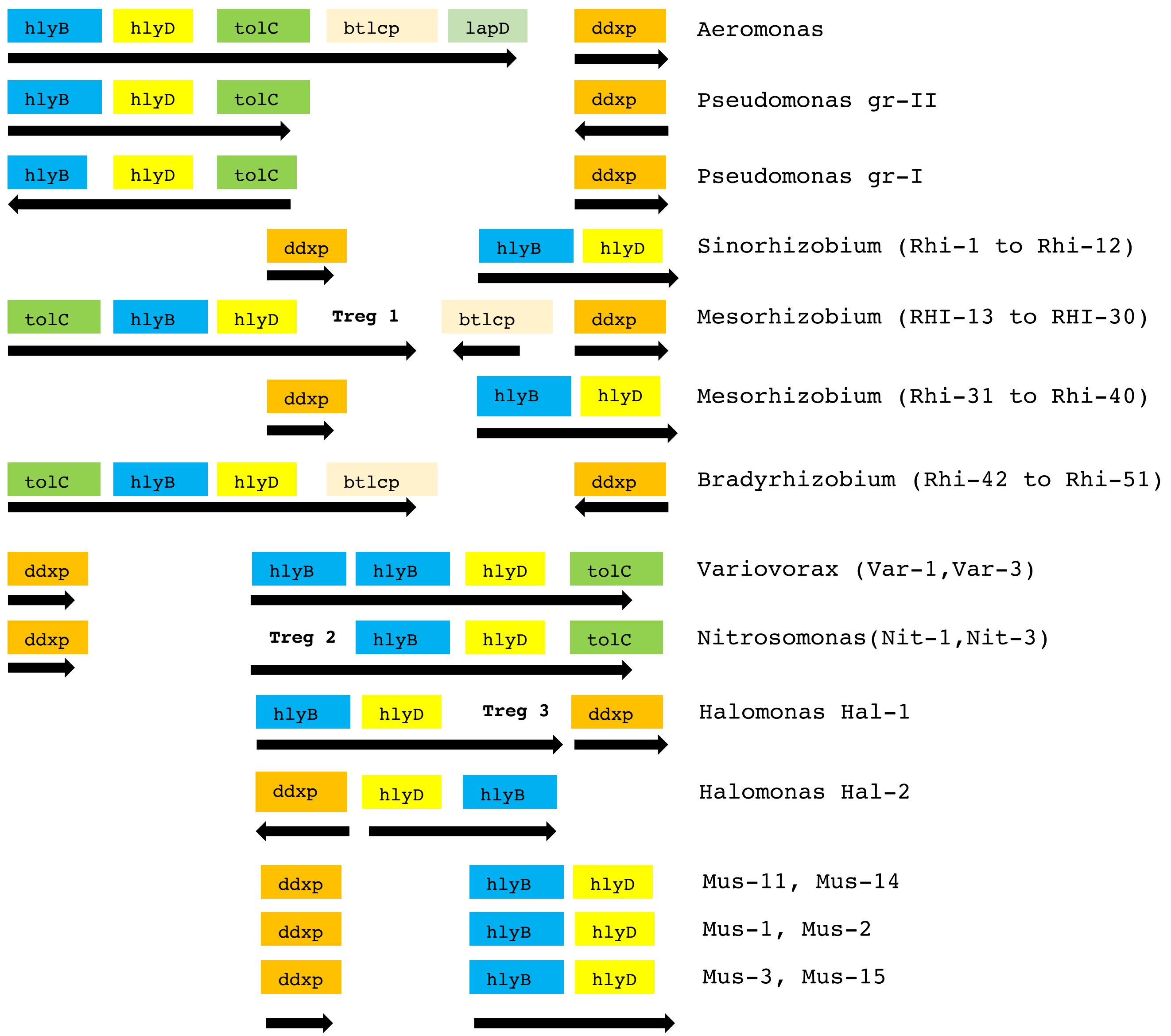

Figure S7. Alignments of DDxP repeats in each branch of the tree shown in Figure 4B. Alignments were generated by the Muscle software [18].

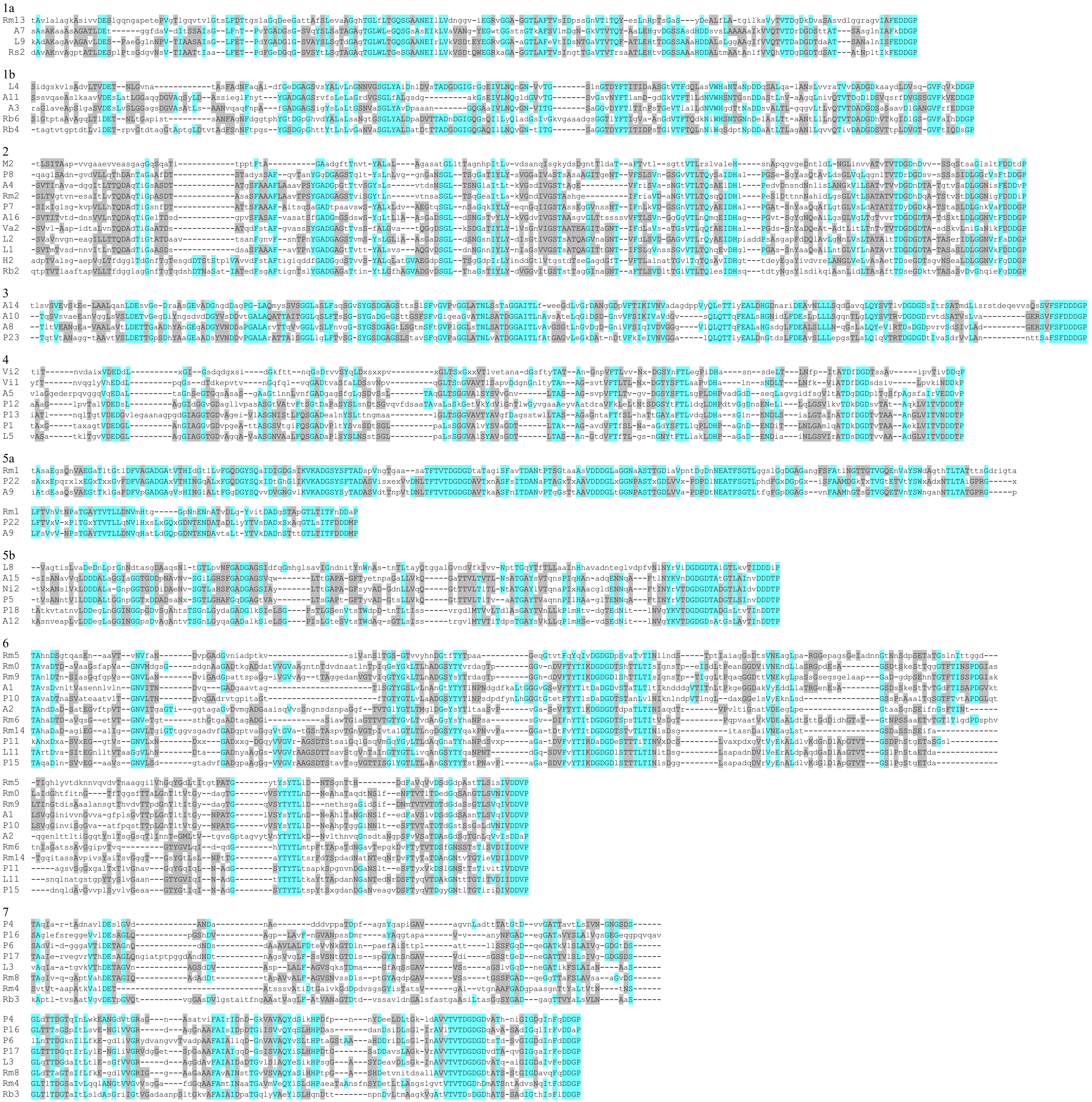

```
8
P21   kitgfelqgGgrVyVDESvGTAGSSrDEnGyvApNDEq---ghgnvlGYAriqgsDLFnlaTngGSDGldASrtqfAlSLVsegvdSGLdaTAgGnillytdsnG--nVlGKvgl---DtiFKISVntvDGsiTleQYkAIaHGntashDelAL------IreGLVkLgvTvyDkDGDSdSaslDLGkvVgFEDDGP
L7    -vVdMavkAGaaltlDEtkGv------kaGdAnANDEa-AsAdAndIGYAklvgsDLFTlTkDaGSDGEqST----lFkLlvsApASGLvDTATnQAIVLSanaGgteVlGKntnG--DVVFKvlltASDGDVevfQYRAIkHenaSdhDeSgaggiierIqAGslkLEvTLTDkDGDSakgelDLGqMmrFEDDGP
H1    -AIaLTslA-pTIaVDESlGTAGSSKDEpGnAApdDEtnAnApAGaIGYAvTtAAtLFTeTaDaGtDGEaSk----vYALVlnAgASGLtDTASGQAVVLSdnsG--vieGrtddaNaDLVFsIAVdATtGDVTTtQYRAldHGAdSNDhDSAVS-----maAGLVELEATLTDGDtDtaSsKIeLGSLIgFEDDGP
Rs1   -AIdLTslA-pTIaVDESvGTgGStKDEpGgAAANDEaaAGApAGaIGYAvTaAAtLFseTTEpGSDGEdgT----AYSLVltAgAtGLtDiATGsAVVLSlvgG--vVeGrsatGN-hLVFtIAVasTtGnVTTtlYRAlsHGAdSNDhDSAVS-----maSGLVELQATLTDGDGDtgSdKIeLGSLIdFEDDGP

9
Rb8   TaHIsLnes------ptLiVdEtd-------GV--gaPgeVdpvgGnLGTnTilGAtlfnnTsvfGsDG-AaSttPtvYSLSlls--TvSGLtDtATGnAitLflNGgvIE----GHaGtlstdpLVFTLSiNSSTGdtTLTQfRAVvhgTeTDPDTsEaatLADnAVgvTvTVTDKDGDSshdtanLngvVkFlDDGP
Rb7   SiHvvLdGEgegqvLtNLvVdESnLTAvTN-GieGSihnginsaiGwfG-----GiF----qansGADGvAdSehPisYALSIgpSGT-SGLVDAqTGeAVVLlmNGtTvE----GHta--aSnlLVFTLSVdSS-GfVTfTvdRsVkEadgTNPDTnEGISLSDGAVTLTATiTDnDGDtAtagiDiGrqVTFlDDGP
Rb1   --sIaLtGn-----VgSLntfEAyLSAaTNaGVnGStPdaVptqghaLdTeSfaGAF----TvvtGADG-Att----aYtLSIagNGTatnLIDSAsGlgVVLdqtGnTIsgyvtGHeG--dAawLVFTLaVNtaTGdVTLTQdRAVhElTassPDTGEGISLtgGlVTLTATVTDKDGDSAaqnlDLsshVTFhDDGP

10
P9    ---------------------------------------------------------------------------------------------------------------------SixP----------------------------------xxxxdIxLtVDETxLxxD-AtSxxxxvaxLFtahfGADGaGSit
A13   TIQVGeLDLNSSVTFlGSNAGYSNSYGYYIKGDDGTPVSGQVIWANVHNQSSGNqvDIsaLDPAhTGFFVIPNGGaNpsLgNntvVTFQLVdGiWQAFMGsTPLsGADGANIVFSDASLNPGGSHLQDTGSAGNQNWEDmTanSDYDYNDVSTSVTWGgaVQLQVDEsnLnhD----AiADFSGvFNVQpGADGLGSqn
P19   TLQVGaLDLNSSVTFhGTDAGYSNSYGYYIKGEDGTPLSGKVIWANVHDQSSGDtfDLgdLDPAsTGFFIIPNGGgNagLlNgadVTFQLVgGkWQAFVGgTPLiGADGANVLFSDAtLNPGGSHLQDTGNAGNQNWEDkTdtSDYDYNDVSTSVTWGstLQLQVDEThLeqgnSaTAtADFSGLFNVQpGADGLGSld

P9    YKvEtxdgtdSGLkDTATGxxIfLFntaSgvEGRVGgxNGxvaFrvTlxxDGxITLDQvRALvHPdTxDxndplsLxxGkixLTaTiTDxDGDxxSasiDlGskltFlDDGP
A13   YsLnIqN-vnSGLiDTATGEqIrLvmndStIEGRta-ttGElVFsLTVdgsGKvTLDQLRAVEHP-TsDPDEAtFLGSGHVsLTLTVTDkDGDhaSGvLDIGKvISFkDDGP
P19   YELQVtN-paSGLvDTeTGEdvvLFlegdqIvGRVGdaNGEaVFtLTVsaDGKITLeQLRAIEHP-TaDPDEAaFLGSGHVeLkLTVTDgDGDsvSGgLDIGKvISFrDDaP

11
A6    SIS-AgSAAnDSLQVDEsNLslNAStsFAslF--TSSfGADGAGTLv--YSLNVsaAGaDSGLnDTATGQdilLYLesGsVV-GRVGg-----sSGsVaFTlSVnssGeVTLDQeRAIaHtPNsGPDQesSLlsAnLvkL--vgTvTDKDGD--SqSASvdLGsAiSFKDDGP
P20   tIS-AgqAgvaSLQVDETtLAiNAttNFsgaF--TSnYGADGAGsIs--YvLdVKSqGVDSGLkDsAsGsdikLYLegGeVV-GRVGd-----qnGaIaFkVtVDAsGnVsLDQvRAIVHsPNTGPDQatgLsAADLvkL--vaTITDKDGD--SnSASLNLGnAiSFRDDaP
L10   SIN--ASAAaDSLtVDETtLgndASANFADNF--TSSfGeDGAGqId--YSLSVh-eGV-tGLvDTATneaVvLsiNndGVVEGRtan------SndlvFTVtVDsAGtVTLDQlRAVVHsPNTGaDQptSLnAADLIqL--TaTITDKDGD--SaSAhLdLGQALnFKDDGP
Rb5   aITMtAtApaDaLvVDETNLAiNASANFADNFTvTSSYGADGAGTtaSAYTLgiKSAGVESGLiDvATGQhVyLYvN-GnVVEGRVGaggaadpaGvIdFTVSVsAAGvVTLDQvhAlqHPnaanPDdvvTLsAtDLItLTRTdTITDRDGDttSgSASiNiGQALSFhDDGP
Va1   SIxVAdtAppDaLtVDETaLAtNASAsFADNFSsTpxYGADGAGTVxSAYALSVKSAGVaSGLvDTlTnQsVvLtLNaGGVVEGRtai------SGxlvFTVSVDAAGlVTLDQqRAIVHPdxTnPDdskTLaAADLIvLTRTdTITDxDGDqntgSdfLNiGQALnFEDDGP

12
P3    SITpSeasvPTLTtDdThipDsDgptSFAdlFnPdFGkDGFKDADDDNxqdADAxTYALGVsAdng-VdSGLVDTlsGdkiyLfLenGsVVGRvGTa-agqadpTGdialTiaVDAntGaVTLtQnnsVVHdDPqDPaEts-TSAagLaAaNLVTLTATITDGDGDTAStsrdIGdafkFEDDGP
Ni3   tIAAnteptPTLTtDdTeivDTagpvSFAglFTsaFGnDxFKDADDDDvqdtDAITYtLGVsganA--vSGLtDTlTGdgVvL--------nkvGnDivgtagAaGpeVlritVDA-SGNVaLsQsRAVVHdDPlDPs--eSsSpatLltadLVTLaATITDGDGDTATATrNIGdafkFEDDGP
Rm10  SIvpSGnDvPTLvVgdsdfv-TDDstSFAAlFTPnFGvDG--------paAtDdvTYtLdVlgGGSnVaSGvfDTlsGleVlLNvvGGdiVGtaGT----------deVFriSVDA-dGNVTLDQsRAVVHgDPtDPvEaD---tpvilAaNLVsLTATaTDGDGDSAhtsVdIGgaftFEDtGP
L6    SIvvSGat-qTLTVDEsvLt-TnDTqSFAglFTPsFGADG--------AaAAnALsYsLGVsAnGA--aSGvlDTAsGnqVfLfLenGiVVGReGSD--AldaATGdvVFniSVDg-SGNVTLDQvRAVVHdnPlDPD--eSTgptqLSAaNLVTLTAiaTDkDGDSASATaNIGlSfnFEDDGP
Ni1   SINttGxE-PxLTVDETvLa-TnDTknFAAnFTsaFGxDG--------AGt---LxfALGIsAsGx--dSxLVDTAxxnhVfLfLxxGvVqGRxGTD--AxxaAgGdiVFTVxxx--xGNVTLDQQRAVVHPDatnPD--D---AkxLlADxLVTLTgTIxDxDGDxxSATLNIGxNLvFEDDGP
P2    SIxttGtE-PTLTVDETvLt-TDaTqSFAAnFssaFGADG--------AGt---LTYALGVvAG----xSGLtDTATGEaVnLSLnGtvVeGRTaT--------TnlpVFTVSVaA-nGdVTLDQQRAVVHPDatnPD--D---StsLTsDNLVTLTATITDkDGDSAxATLNIGqNLvFKDDGP
M1    SISttGtE-PTLTVDETvLa-TDDskSFAAnFssaFGADG--------AGt---LTYALsVvAG----pSGLVDTATnQaVnLSLnGsvVeGRTav--------SndlVFTVSVDA-SGNVTLDQxRAVVHaDxxDxD--D---AatLaADNLVkLTATVTDkDGDhqAATLNIGqNLxFxDDGP
Rm3   tISASGas-asLTVDETvLl-TnDTkaFAsaFTssyGADG--------AGA---ITYALGfnAG----stGLVDTlsGQaVvLSLeaGqVVGRaGa--------gGaiVFTVStDA-SGNVTLDQQRAVVHPt-sDPN--e---pvsLTADNLVTLTATITDkDGDSsAATLNIGqNLtFlDDGP
Rm7   SvTASatQ-PpLTVDEsnLg-qnaTaSFAsvFTPvFGADG--------AaAAnSvTYALGfnAG----atGLVDTATGQaVvLSLnGtvVeGRTav--------SnelVFTVatDA-SGNVTLDQQRAVVHPnaaDPN--D---AksLSADNLiTLTATVTDkDGDTATATaNIGqNLtFlDDGP
Rm11  tISASttnePTLTVsEatLg-TpssgaFggqFTvdykADG--------paAsnptTYALstpgG----dSGLIDTlTGQhVqLSkvGsqiVGtinn--------gtttVFTVSVDg-tGqVTLtQsRAVVqatgsnPDtge---gigLTgsNLVvLTATaTDGDGDSAStpLdltpqLqFtDvGP
Rm12  SITASainaPpLTVDETvLa-TDaTgtFAAqFTPtFGADG--------qGAt-pvsYALstpgG----aSGLtDTATGEsVvLSLvnGqiLGkTaT--------TGltVFvVSVDA-SGqVTLDQQRAiVHanPnDPN--e---SrgLTgsNLVvLTATaTDGDGDhASApLdltplLaFKDDGP
```

Figure S8

Homologies of DDxP repeats to the T1SS-143 model sequence. Homologous sequences are in red
Percent identities range from 75.44% (Vi2 repeat) to 30.77% (L11 repeat)

```
T1SS-143 query              GQFTVTQGADGVVSYQLDDSTNPVAGLTSGGQAVTLSETSNADGNFTYTATAGGNPVFTLTLNADGSYEFTLEGPLDHAAGSDELTLNFPIIATDFDGDTSSITLPVTIVDDVP
                            TITDVDALTVDEDDLPGGSDGSK

                            G+FT  QG+D VVSYQLD S  PV GLTS G  VTL ET+NADG+FTYTATA GNPVFTL +N DGSY FTLEGP+DHA+ SDELTLNFPI ATDFDGDTSS  +PVTIVDD P
Vi2                         GKFTTNQGSDRVVSYQLDXSXXPVXGLTSXGXXVTLVETANADGSFTYTATANGNPVFTLVVNXDGSYNFTLEGPIDHASNSDELTLNFPITATDFDGDTSSAVIPVTIVDDQP

                           GQF + QGAD V S+ LD S NPV GLTS G AVTLS    + +GN TYTA AG   VFTLTLN DG+Y FTL  P+DHA  S++LTLNF +IATDFDGD+ SI LPV I DD P
Vi1                        GQFQLVQGADTVASFALDSSVNPVQGLTSNGVAVTLSAPVDDGNGNLTYTAMAGSVTVFTLTLNXDGTYSFTLAAPVDHALNSNDLTLNFKVIATDFDGDSDSIVLPVKINDDKP

                            GAD  +SY L+ ST+ +  L+SGG A++ + + +     T TA+A G  VFT TL ++G+Y FTL   LDH AG+DE    + L   I ATD DGDT   ++  L +T+ DD P
L5                         SGADAPLSYSLNSSTSGLPALSSGGVALSYAVSGD-----TLTASANGTDVFTFTLGSNGNYTFTLLAKLDHPAGADENDIAINLGSVIRATDSDGDTVVAAADGLVITVDDDTP

                            GAD  ++Y +   T+ +  L+SGG A+  S   +     T TA AG   VFT +LNA G Y FTL  PLDH AG+DE  +TLN   +  ATD DGDT   ++  L +T+ DD P
P1                         SGADVPLTYSVSSDTSGLPALSSGGVALVYSVAGD-----TLTAKAGAVDVFTFSLNAAGDYSFTLLQPLDHPAGNDENDITLNLGAMLQATDKDGDTVTAAAEKLVITVDDDTP

                V  GADG  S+ L  D S+    GLTSGG A++ S   N       TA+AG  PVFTLT+  DGSY FTL GPLDH       S++L    ++F   + ATD DGD  + + P     + + DDVP
A5              VNFGADGAGSFGLGSDVSSLTAQGLTSGGVALSYSVVGN-----VLTASAGSVPVFTLTVGVDGSYSFTLSGPLDHPVADGDDSEQLAGVGIDFSGVLTATDGDGDPLTGSFPAGSFAIRVEDDVP

                         GAD  ++Y L  +     GLTSGG AVT +   +A  + +  TA+AG GN  FT +L+A  G+Y FTL   LDHA+G +E    + L   I ATDFDGDT +     L +T+ DD P
P13                     SGADEALNYSLTTNGMAALGLTSGGVAVTYAVGFDAGSSTWLLTASAGAGNTAFTFSLHATTGAYAFTLVDQLDHASGLNENDLSIALGTAINATDFDGDTVTAAANGLVITVNDDTP

                                                                           G   V T+TLN A G+Y  T   P+ HA    E     TLN+ +   D DGDT++ TL + + DD P
A15                                                                        GATTVLTVTLNSATGAYSVTQNSPIQHANADQENNQAFTLNYKV--NDGDGDTATGTLAINVDDDTP

                                              +AG  SGG  A+T +   NA G +             + LNADGSY +TL    + P +   G++ LT +F     D  G++++ T+ +TIVDDVP
P11                                           LAGSVSGGXGALTXTLVGNAVGQYG-----------QIQLNADGSYTYTLTSAPKSPGNVNDGANSLTESFTYXVKDSLGNSTTSTIVITIVDDVP

                                                                       G N VF + +N   G Y FTL   ++H A +D         + LN+ +I  D DGDT+  TL VTI DD+P
L8                                                                     GVNDVFKIVVNPTTGQYTFTLLAAINHHAVADNTEGLVDPFVNLNYRVI--DGDGDTAIGTLKVTIDDDIP

                          G      GAD + S +L   + P+   + G ++VT S      G  + ++T G     T+T  + G+Y   L  PL H+     D +TLN     TD DGD+++ +L VTI DD P
A12                       GNLGYQAGADALKSLEL---SGPI---SLGTESVT-STWDAQSGTLSISSTRGVLMTVTITDPSTGAYSVKLLQPLMHSEVDSEDNITLNVGYKVTDGDGDSATGSLAVTINDDTP

                         G      GADG+ S +L   +      T G + VT   T + D N  T ++  G      LT  A G+Y   L  PL H      D +TLN     TD DGDT+  +L VTI DD P
P18                      GNLGYDAGADGLKSIELSGPS------TLGSENVT--STWDPDTNTLTISSVRGDLMTVVLTDLASGAYTVNLLKPLMHTVDGTEDNITLNVGYKVTDGDGDTADGSLTVTINDDTP

                                                                G +TY+     N  +  + +NADG+Y +TL          ++ A +++   +F    TD  G+T++ T+ V I+DDVP
L11                                                             GPYTYSLVGAANGTYGVIQINADGTYTYTLTKAYTTAPDANNGANTEDNRDSFTYQVTDAKGNTTTGTITVDIIDDVP
```
